# Second-generation CK2α inhibitors targeting the αD pocket†
†Electronic supplementary information (ESI) available: All experimental details, crystallographic data collection and refinement statistics, details of chemical synthesis, additional figures and tables. See DOI: 10.1039/c7sc05122k


**DOI:** 10.1039/c7sc05122k

**Published:** 2018-02-20

**Authors:** Jessica Iegre, Paul Brear, Claudia De Fusco, Masao Yoshida, Sophie L. Mitchell, Maxim Rossmann, Laura Carro, Hannah F. Sore, Marko Hyvönen, David R. Spring

**Affiliations:** a Department of Chemistry , University of Cambridge , CB2 1EW , Cambridge , UK . Email: spring@ch.cam.ac.uk; b Department of Biochemistry , University of Cambridge , CB2 1GA , Cambridge , UK . Email: mh256@cam.ac.uk; c Structure Biophysics & FBLG , Discovery Sciences , IMED Biotech Unit , AstraZeneca , Cambridge , UK; d R&D Division , Daiichi Sankyo Co., Ltd. , 1-2-58, Hiromachi, Shinagawa-ku , Tokyo 140-8710 , Japan

## Abstract

CK2 is a critical cell cycle regulator that also promotes various anti-apoptotic mechanisms. Development of ATP-non-competitive inhibitors of CK2 is a very attractive strategy considering that the ATP binding site is highly conserved among other kinases. We have previously utilised a pocket outside the active site to develop a novel CK2 inhibitor, **CAM4066**. Whilst **CAM4066** bound to this new pocket it was also interacting with the ATP site: herein, we describe an example of a CK2α inhibitor that binds completely outside the active site. This second generation αD-site binding inhibitor, compound **CAM4712** (IC_50_ = 7 μM, GI_50_ = 10.0 ± 3.6 μM), has numerous advantages over the previously reported **CAM4066**, including a reduction in the number of rotatable bonds, the absence of amide groups susceptible to the action of proteases and improved cellular permeability. Unlike with **CAM4066**, there was no need to facilitate cellular uptake by making a prodrug. Moreover, **CAM4712** displayed no drop off between its ability to inhibit the kinase *in vitro* (IC_50_) and the ability to inhibit cell proliferation (GI_50_).

## Abstract 2

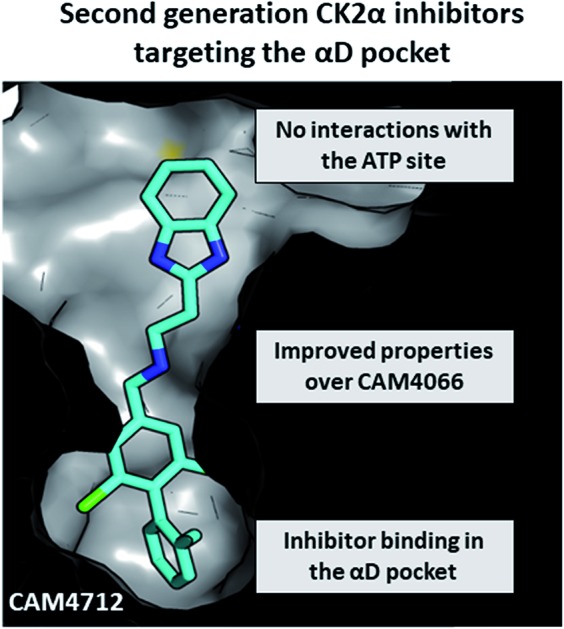
We describe the development of a **CAM4712**, a novel CK2α inhibitor which does not interact with the ATP binding site and shows improved properties over the first-generation inhibitor **CAM4066**.

## Introduction

CK2 is a serine/threonine kinase that is a key regulator of many cellular processes and is involved in cellular proliferation and anti-apoptotic mechanisms.^1^*In vivo* it exists mainly as a holoenzyme composed of two catalytic (α and/or α′) and a dimer of regulatory (β) subunits, but it can also be found as the isolated subunits.^2^ Unlike most other kinases it is constitutively active and more than 300 proteins have been identified as CK2 substrates, making it probably one of the most pleiotropic proteins in eukaryotic systems.^3^ Elevated levels of CK2 have been found in a variety of cancers, including leukaemia, breast, lung, prostate, colorectal, renal and glioblastoma brain tumours.^4^,^5^ It has been shown that cancer cells are particularly susceptible to CK2 inhibition because they rely on high levels of the kinase to survive.^6^ CK2 overexpression has been associated with multi-drug resistance phenotypes and it has been demonstrated that CK2α inhibition leads to an increased uptake of known drugs in multidrug resistant cells.^7^,^8^ It has been shown that CK2 inhibitors are able to limit the growth of a range of cancer cell lines.^9^,^10^ Hence, CK2 has been recognised as a highly promising target for anti-cancer therapies.

Like the majority of kinase inhibitors, most of the known CK2 inhibitors target the ATP binding site, presenting the issue of poor selectivity over other kinases.^11^–^13^ This is the case for **CX4945** (known as silmitasertib), the first in class CK2 inhibitor currently in phase II clinical trials.^14^,^15^ The IC_50_ of **CX4945** against CK2 is 1 nM but it also inhibits 12 other kinases with nanomolar affinity and it is more potent against Clk2 than against CK2.^16^,^17^

**Fig. 1:**
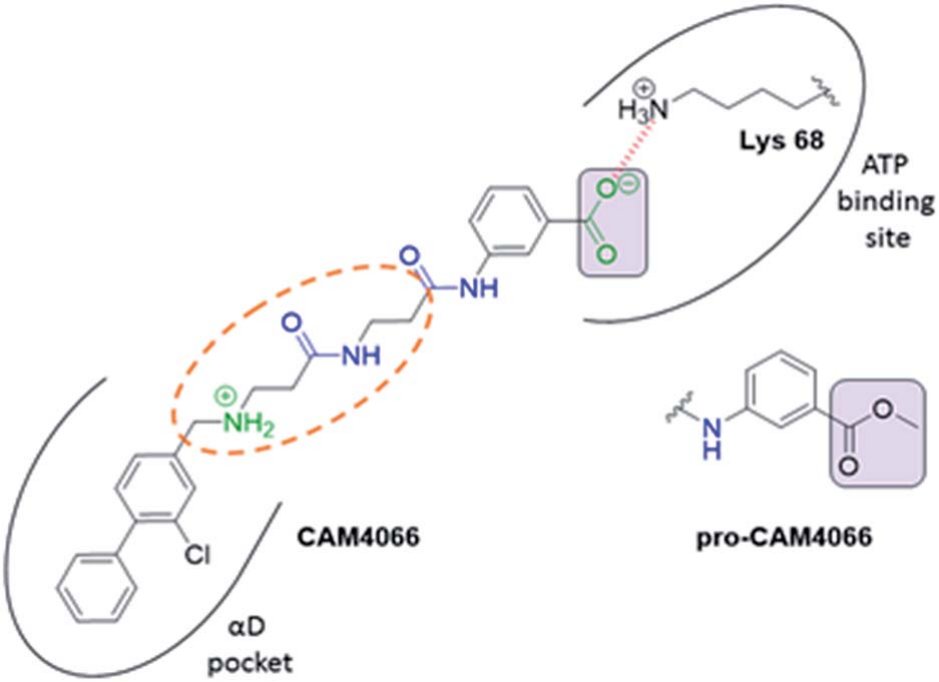
Structure of **CAM4066** and **pro-CAM4066**. Zwitterionic elements are coloured in dark green, amide bonds in blue and the difference between **CAM4066** and its prodrug highlighted in purple. The interaction between **CAM4066** and the highly conserved Lys68 is shown as red dashed line. The flexible linker is circled in orange. The αD pocket and ATP binding site are reported as black curves.

Previous work from our groups led to the discovery of a new binding pocket on CK2α, which is located adjacent to the ATP binding site. This pocket was revealed in a X-ray crystallographic screen, in which several weakly binding fragments where found to occupy this novel site formed through a movement of the αD helix, hence the name of αD pocket.^18^,^19^ Through fragment growing and linking, we generated a novel selective CK2 inhibitor: **CAM4066** (Fig. 1).

**CAM4066** was a valuable tool for validating the concept of using the αD site to develop selective inhibitors of CK2α; however, it has several structural features that are undesirable in a lead molecule or chemical tool. These features, shown in Fig. 1, include a long flexible linker (circled in orange), a zwitterionic nature (the amine and the carboxylate are highlighted in green), amide bonds (coloured blue) and a high MW, which is often associated with poor oral bioavailability (Fig. 1). Moreover, the carboxylate forms a salt bridge with the conserved Lys68 in the ATP binding site. As expected due to its physicochemical properties, **CAM4066** suffers from poor cellular permeability and therefore the methyl ester derivative, **pro-CAM4066**, was used as a pro-drug to improve cellular activity and target engagement.^18^ The aim of this work was to develop enhanced CK2α inhibitors that have improved physico-chemical properties and bind to the αD pocket without reaching deep into the ATP pocket. Our ideal lead-like candidate would have a smaller number of rotatable bonds (<10), not be susceptible to protease action (absence of amide groups), and be cell permeable without resorting to the use of a pro-drug. In addition, we aimed to develop inhibitors that do not rely on any of the conserved interactions within the ATP binding site.

**Fig. 2:**
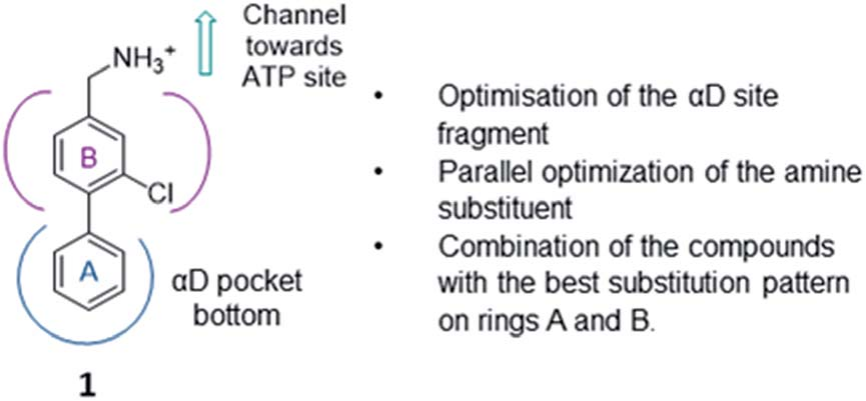
Optimisation strategy adopted in this project using **1** as the fragment starting point.

The strategy (shown in Fig. 2) involved a fragment optimisation and a fragment-growing stage, followed by merging of the best compounds. Firstly, we would optimise the αD site fragment further to gain higher affinity and secondly, we would grow the fragment into the upper part of the αD pocket in order to gain inhibition.

Finally, the compounds with the most promising substitution patterns would be combined to provide the final inhibitor that would show inhibition of the kinase activity, good cell permeability and efficacy in cellular assays.

## Results and discussion

Previously we have reported our preliminary studies on the exploration of the αD pocket, based on the development of primary hits from the crystallographic screen, which led to the identification of compound **1** (*K*_d_ = 267 μM), shown in Fig. 2. Our strategy to optimise the αD site fragment was to concentrate on ring A of the biaryl structure; however, a brief investigation of ring B was also performed. In parallel, optimisation of the amine substituent was carried out growing in the channel that connects the αD site and the ATP binding pocket.

### Optimization of the αD site fragment

#### Ring A

**Fig. 3:**
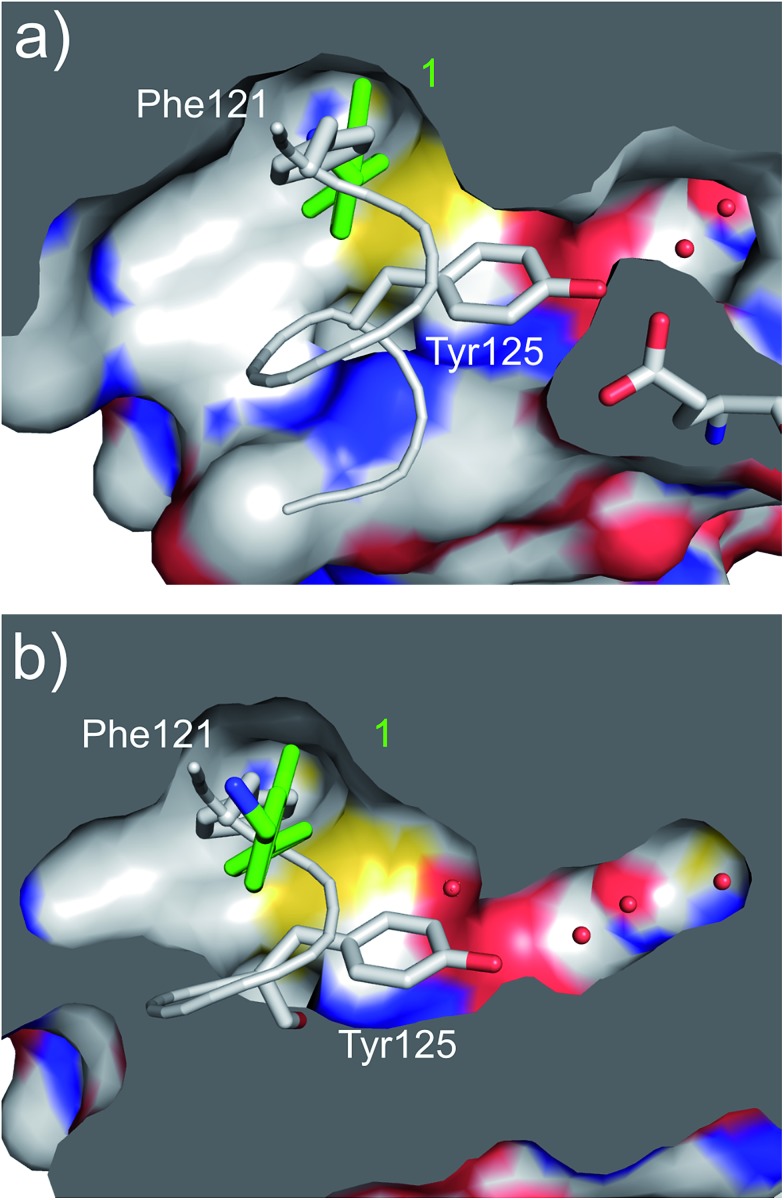
(a) A cross section of the αD pocket of **1** (green) bound to CK2α. The first two water molecules in the water channel are shown (PDB: ; 5CSH). (b) The structure of the αD loop (in grey) in the closed conformation of the apo protein (PDB: ; 5CSP).^19^ Tyr125 fills the mouth of the water channel. The binding mode of **1** (in green) superimposed on the apo structure is shown, with **1** occupying the same position as Phe121.

Ring A effectively fills the bottom of the hydrophobic pocket of the αD site (Fig. 3). However, on closer examination we uncovered a side channel off the main pocket filled by several well-defined water molecules that could be targeted to improve the affinity. Comparison of the co-crystal structure of CK2α and **1** (PDB: ; 5CSH, Fig. 3a) with the closed apo structure (PDB: ; 5CSP, Fig. 3b) shows that the side channel in the closed structure was occupied by Tyr125. This indicates that this channel can be targeted as it has sufficient volume to accommodate sizable groups, *i.e.* the phenolic ring of the Tyr125, and that the waters can be displaced as this happens in the closed form of the αD loop. Modelling studies indicated that **1** contains the right vectors to grow into the Tyr channel by substitutions on the 2-position of ring A.

**Fig. 4:**
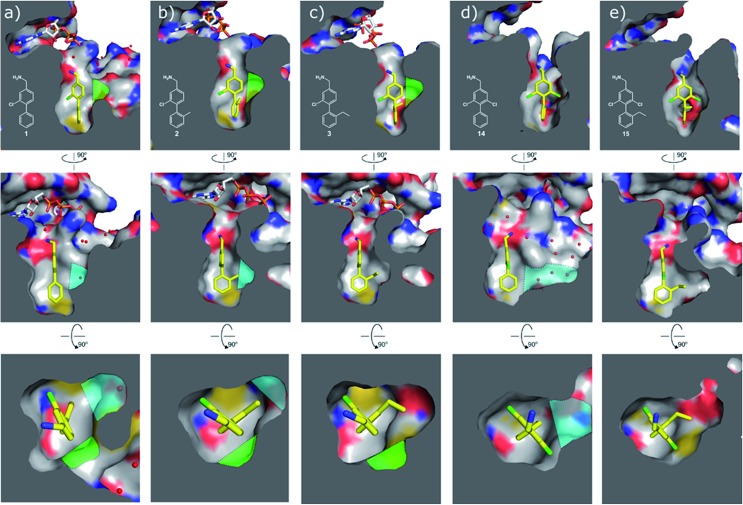
Cross sections of the αD and ATP pockets: first and second rows are lateral views and last row is a view from the top. The water channel is highlighted in blue and the hydrophobic pocket in green for complexes that are not making the most of the space available. (a) Co-crystal structure of **1** with CK2α (PDB: ; 5CSH); (b) co-crystal structure of **2** with CK2α (PDB: ; 5ORH); (c) co-crystal structure of **3** with CK2α (PDB: ; 5ORJ); (d) co-crystal structure of **14** with CK2α (PDB: ; 5OTR); (e) co-crystal structure of **15** with CK2α (PDB: ; 5OTZ).

A robust crystallographic system for CK2α enabled us to use X-ray crystallography as our primary screening technique. Therefore, co-crystal structures of all the compounds were attempted. The majority of the structures showed the ligand bound to CK2 and for all the compounds showing a clear electron density *K*_d_ was determined *via* ITC (overview of the results of the ITC experiments can be found in Table S1†). Several structures that did not show clear electron density for the ligand were also investigated using ITC to provide SAR data and controls. A number of mono- and di-substituted rings and bicyclic systems were investigated (Table 1 and 2, Fig. 4).

**Table 1:** SAR on the bottom ring of **1** (ring A)

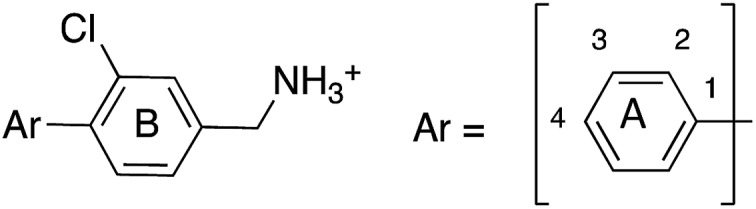									
Compound	Ring A	*K*_d_ ^*a*^ (μM)	LE^*b*^	PDB	Compound	Ring A	*K*_d_ ^*a*^ (μ M)	LE^*b*^	PDB
**1**	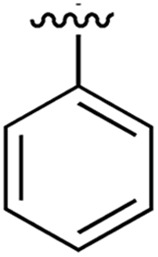	267	0.33	5CSH	**7**	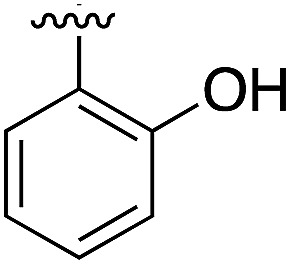	375	0.30	5OSL
**2**	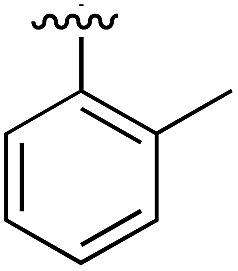	41	0.38	5ORH	**8**	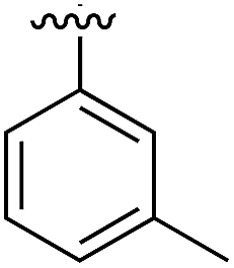	>500	—	n.a
**3**	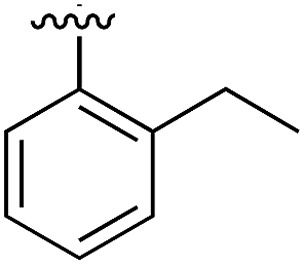	17	0.39	5ORJ	**9**	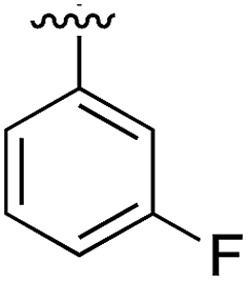	>500	—	5OUL
**4**	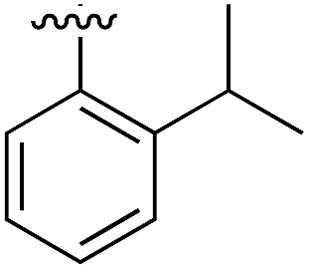	205	0.29	5OS7	**10**	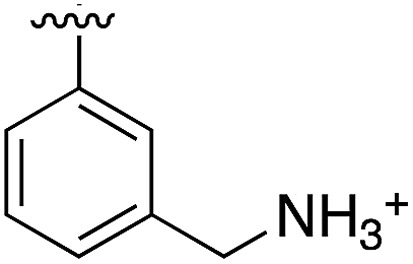	>500	—	n.a
**5**	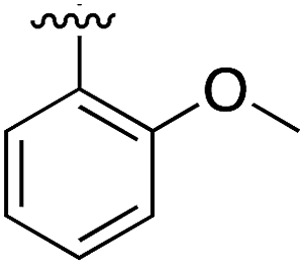	244	0.30	5OQU	**11**	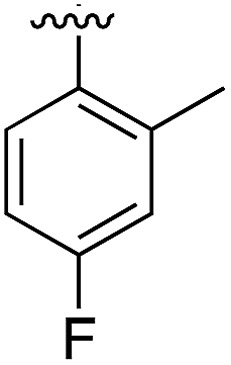	105	0.33	5OS8
**6**	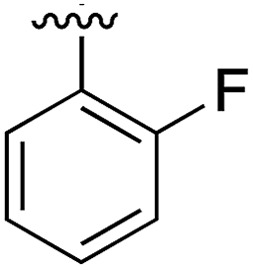	234	0.32	5ORK	**12**	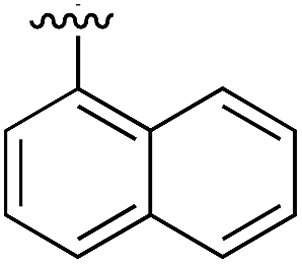	250	0.27	n.a
					**13**	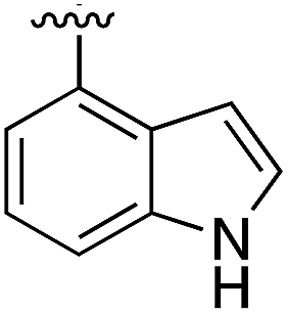	>500	—	n.a

^*a*^Measured by ITC.

^*b*^LE = ligand efficiency*. *Ligand efficiency is defined as the ratio of the Gibbs free energy of binding of a ligand divided by the number of heavy (non-hydrogen) atoms in the molecule (LE = Δ*G*/[number of heavy atoms]).^20^

**Table 2:** SAR studies on the bottom ring (ring A) of dichloro derivatives **14** and **15**

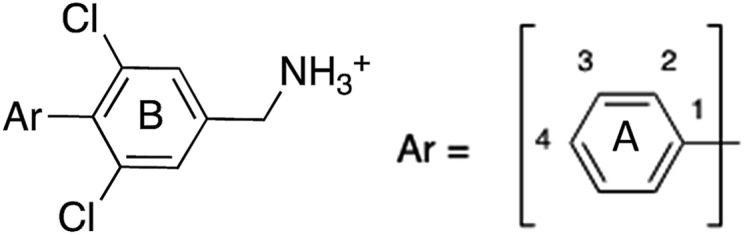				
Compound	Ring A	*K*_d_ ^*a*^ (μM)	LE^*b*^	PDB
**14**	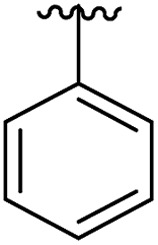	12	0.43	5OTR
**15**	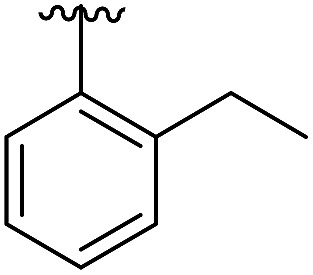	6.5	0.41	5OTZ

^*a*^Measured by ITC.

^*b*^LE = ligand efficiency*. *Ligand efficiency is defined as the ratio of the Gibbs free energy of binding of a ligand divided by the number of heavy (non-hydrogen) atoms in the molecule (LE = Δ*G*/[number of heavy atoms]).^20^

Substitution in the 2 position of ring A was beneficial when compared to the reference compound **1** (*K*_d_ = 267 μM); the 2-methyl derivative **2** was promising with a *K*_d_ of 41 μM. The introduction of an ethyl group in the same position (**3**) resulted in the highest affinity (*K*_d_ = 17 μM) and highest ligand efficiency (LE = 0.39) of the series and the crystal structure (PDB: ; 5ORJ) showed how the lateral chain grows towards the water channel, displacing the first water molecule on the left (Fig. 4b and c). The presence of the chlorine on ring B and the ethyl group on ring A enforced an almost orthogonal conformation of the biaryl system (Fig. 4c). Expanding the size of the lateral chain did not improve the binding (*K*_d_ = 205 μM) nor the LE of the isopropyl derivative **4**. The 2-MeO and 2-F derivatives, **5** and **6** respectively, had a comparable *K*_d_ to **1** while the 2-OH group (compound **7**) was detrimental to binding even though it formed a hydrogen bond to the conserved water (Fig. S1a,† PDB: ; 5OSL). Substitution in the 3 position of ring A (compounds **8** to **10**) appeared not to be tolerated as only the fluoro-derivative **9** yielded a co-crystal structures. The disubstituted 2-Me-4-F derivative **11** was found to have a *K*_d_ of 105 μM, marginally higher than the 2-methyl compound **2**, but with a lower ligand efficiency (LE = 0.33). Expanding ring A to a naphthyl group **12** improved the affinity only slightly, while the indolyl derivative **13** did not show any significant binding and we were unable obtain structures of these in complex with CK2α. Therefore, compound **3** represented the fragment with the best LE and it was chosen as the best optimized fragment as far as the ring A is concerned.

#### Ring B

In many of the crystal structures of the compounds with different ring A substituents two alternative binding modes were observed for ring B, in which the chlorine atom could be on either side of the pocket. Therefore, the dichloro derivative of **1** (**14**) was synthesized to fulfil the interactions that both of these chlorine positions made. **14** showed an improved *K*_d_ of 12 μM with the highest LE of 0.43 (Table 2). The co-crystal structure is shown in Fig. S1b† (PDB: ; 5OTR) where **14** (in blue) is overlapped with the two binding poses of **1** (green). The final compound **15**, merging both the dichloro functionality on ring B and the 2-ethyl group on ring A, was synthesized and showed an improved *K*_d_ of 7 μM (LE = 0.41) with its binding mode overlapping well with that of the merged fragments (Fig. 4e, PDB: ; 5OTZ). Although compounds **3** and **15** showed a comparable LE (0.39 *vs.* 0.41 respectively), compound **15** was chosen as hit to bring forward due to its ability to occupy a larger part of the αD pocket (Fig. 4e).

#### Growing towards the entrance to the ATP site

**Fig. 5:**
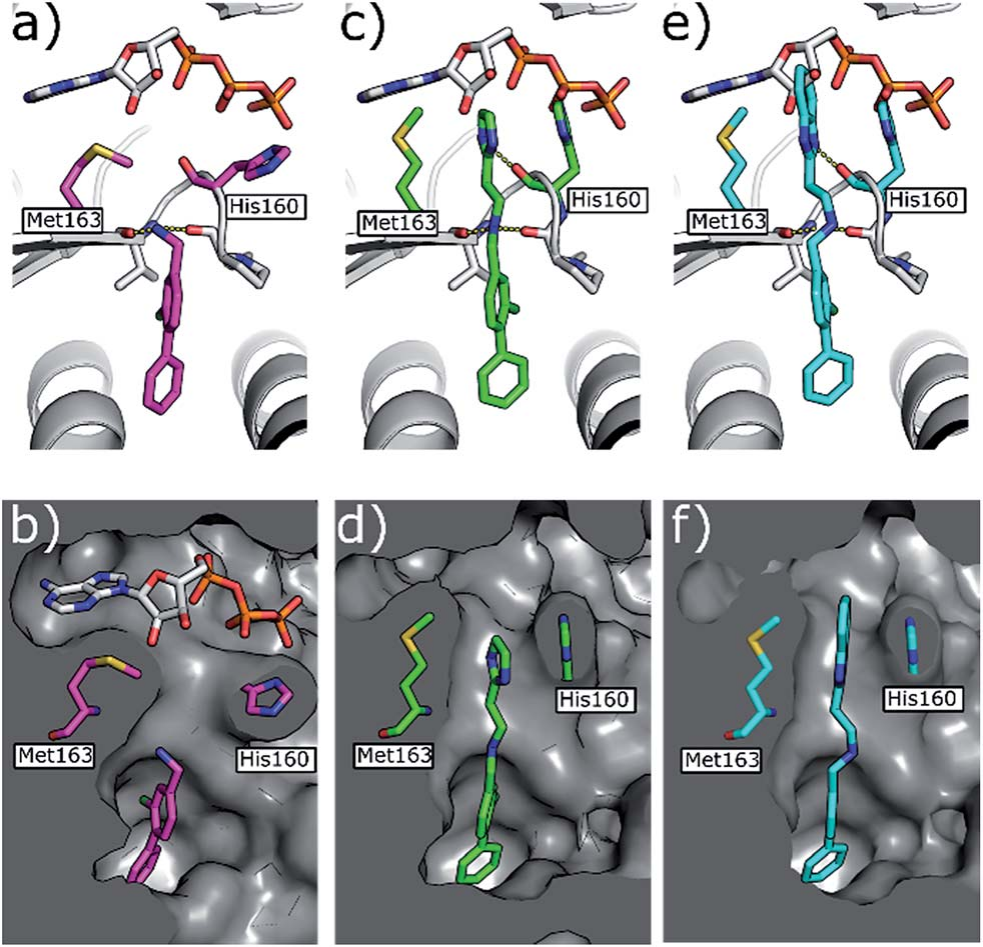
(a) Co-crystal structure of **1** (in pink) and ATP bound to CK2α (PDB: ; 5CSH)^19^ and (b) surface representation of CK2α with **1** bound. (c) Co-crystal structure of **21** (in green) bound to CK2α. The binding mode of ATP (grey) in the ATP site when **1** is bound is superimposed upon the structure. (d) Co-crystal structure of **21** (in green) bound to the surface representation of CK2α. (e) Co-crystal structure of **22** (in blue) bound to CK2α. The binding mode of ATP (grey) in the ATP site when **1** is bound is superimposed upon the structure. (f) Co-crystal structure of **22** (in blue) bound to the surface representation of CK2α.

The compounds tested so far were not able to effectively inhibit the protein activity as ATP was not displaced. Indeed, most of the co-crystal structures featured a molecule of ATP/ADP in the ATP binding site as well as the ligands bound in the αD pocket (Fig. 4 and 5).

During the development of **CAM4066** a series of flexible linkers were designed and tested to join the αD site fragment to an ATP site fragment. These compounds revealed that it was possible to induce the opening of a small channel between the αD and the ATP sites. Our aim was to induce the opening of the channel with shorter, more rigid compounds than **CAM4066**. The flipping of the side chain of Met163 allows the formation of the channel and results in blocking the ATP site – Met163 is located just underneath the adenine base of ATP. Therefore, these compounds would not need to grow deep into the ATP site to achieve inhibition. The channel from the αD site is lined by Met163 and His160 and we envisioned that compounds with aromatic groups that stacked between these amino acid residues would improve the affinity and cause the conformational change that would lead to inhibition. Toward this end, the effect of several aromatic groups on the amine were investigated using a kinase activity assay (Table 3).

**Table 3:** *N*-substituents on the benzylamine^*a*^

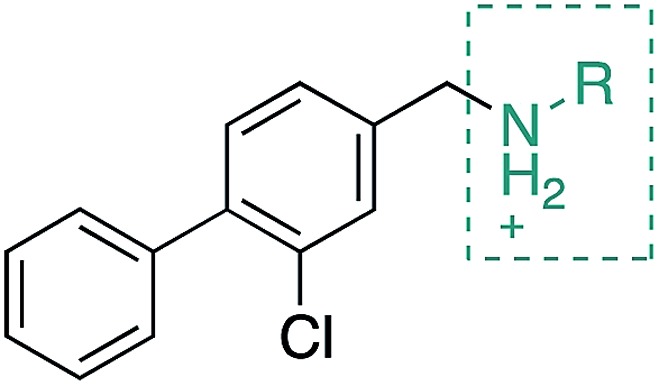			
Compound	R	Inhibition of the kinase activity @ 500 μM (%)	PDB
**1**	H	21	5CSH
**16**	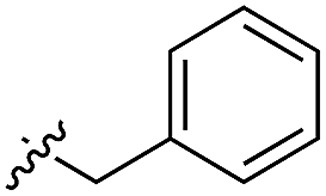	54	n.a.
**17**	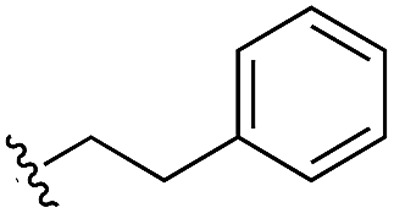	42	n.a.
**18**	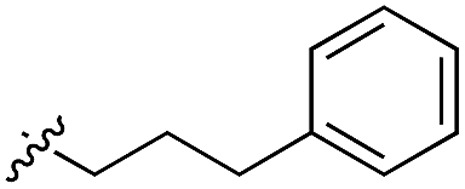	40	6EII
**19**	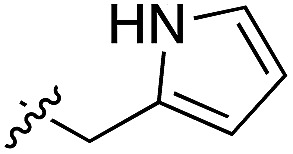	52	5OT6
**20**	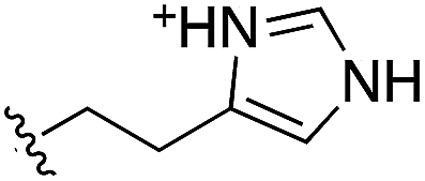	50	5OUE
**21**	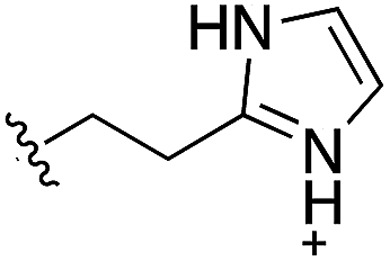	n.d.	5OUM
**22**	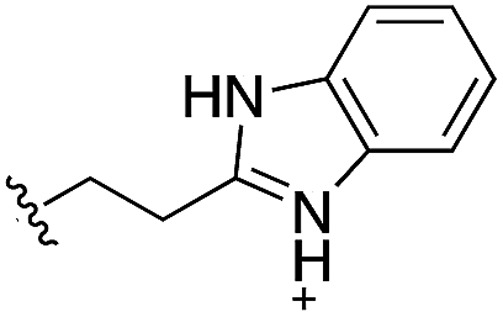	98 (IC_50_ 58)	5OUU

^*a*^n.d. = interference with the assay by the test compound.

Whilst the reference compound **1**, tested at 500 μM, inhibited only 21% of the protein activity, the inhibitory activity of the aromatic derivatives **16–19**, were found to be considerably improved with compounds **16** and **19** being the most promising (54 and 52% respectively). In order to pick up additional H-bond interactions the pyrrole derivative **19** was chosen over **16** and we hypothesized that the heterocycle should go further up into the channel. Whilst the imidazole derivative **20** did not show significant improvement, the benzimidazole derivative **22** was found to be the most potent compound with an IC_50_ of 58 μM. Fig. 5d and f show the co-crystal structure of **21** and **22** with CK2α, respectively. As expected, Met163 flips upon binding of the more extended compounds compared to the co-crystal structure of **1** (Fig. 5b). This explains the displacement of ATP and therefore activity inhibition even without fragments binding directly in the ATP pocket.

As these compounds showed increased activity, the concentration for the inhibition assay was decreased to 10 μM and cellular activity was investigated (Table 4). Although **15** had the highest affinity of the αD binders (Table 2), **3** was chosen for further studies for synthetic reasons, with the idea of retrieving the substitution pattern of **15** in the final compound. Merging **3** with **22** provided compound **23**, which featured higher potency than the original fragments in the inhibition assay and a promising GI_50_ of 10 μM in HCT116 cells. Therefore, SAR studies around the benzimidazole ring were then performed (compounds **24–30**), with both electron withdrawing and donating groups in position α and β of the benzimidazole improving the inhibition activity in respect to the derivative **23**. The α and β methoxy derivatives **26** and **29** were found to be the most promising in the respective α and β substituted series with IC_50_ of 8 and 7 μM, respectively (Table 4, IC_50_ and GI_50_ curves are shown in Fig. S4 and S5†).

**Table 4:** Studies on the benzimidazole substitution

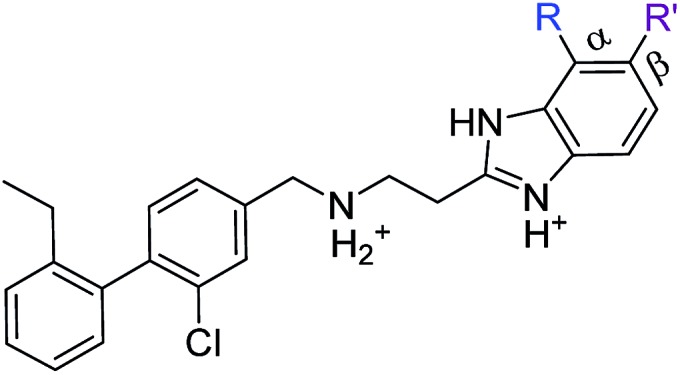					
Compound	R	R′	Inhibition @10 μM (%)	GI_50_^*a*^ (μM)	PDB
**23**	H	H	33 (IC_50_ 18)	10	5OSZ
**24**	Me	H	45	—	5OT5
**25**	NO_2_	H	50	—	50TD
**26**	OMe	H	52 (IC_50_ 8)	11	5OTH
**27**	H	Me	38	—	5OTI
**28**	H	NO_2_	36	—	
**29**	H	OMe	64 (IC_50_ 7)	11	5OTL
**30**	H	Cl	50	—	5OTO

^*a*^Inhibition of proliferation tested in HCT116 cell line.

#### Further modifications of ring B

Alternative substitutions around the middle ring were also investigated and compared to compound **23**. Firstly, the dichloro derivative was synthesized as it gave promising results as a fragment (compound **15**, Table 2). With an IC_50_ of 7 μM, **CAM4712** was the most potent compound compared to the methyl, trifluoromethyl, methoxy and trifluoromethoxy derivatives (compounds **31**, **32**, **33** and **34**, respectively) (Table 5). Substitution of the chlorine atom in compound **23** (compounds **31–34**) was investigated in order to improve the moderate solubility of the related **CAM4712** in water. Unfortunately, compounds **31–34** resulted in loss of activity and were therefore not pursued further.

**Table 5:** Optimization of ring B

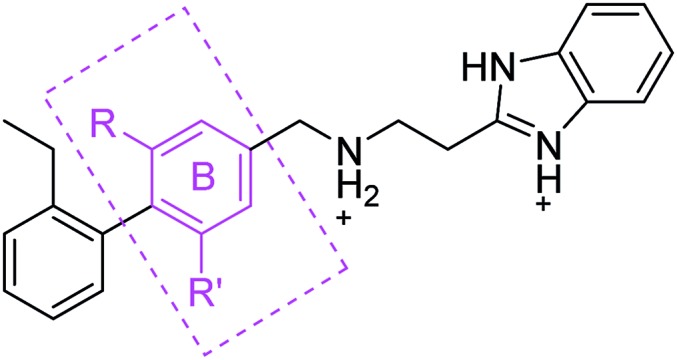				
Compound	R	R′	Inhibition @10 μM (%)	PBD
**23**	Cl	H	33 (IC_50_ 18)	5OSZ
**CAM4712**	Cl	Cl	80 (IC_50_ 7)	5OTY
**31**	Me	H	30	5OYF
**32**	CF_3_	H	23	6EHU
**33**	OMe	H	41	5OTQ
**34**	OCF_3_	H	39	n.a.

**Fig. 6:**
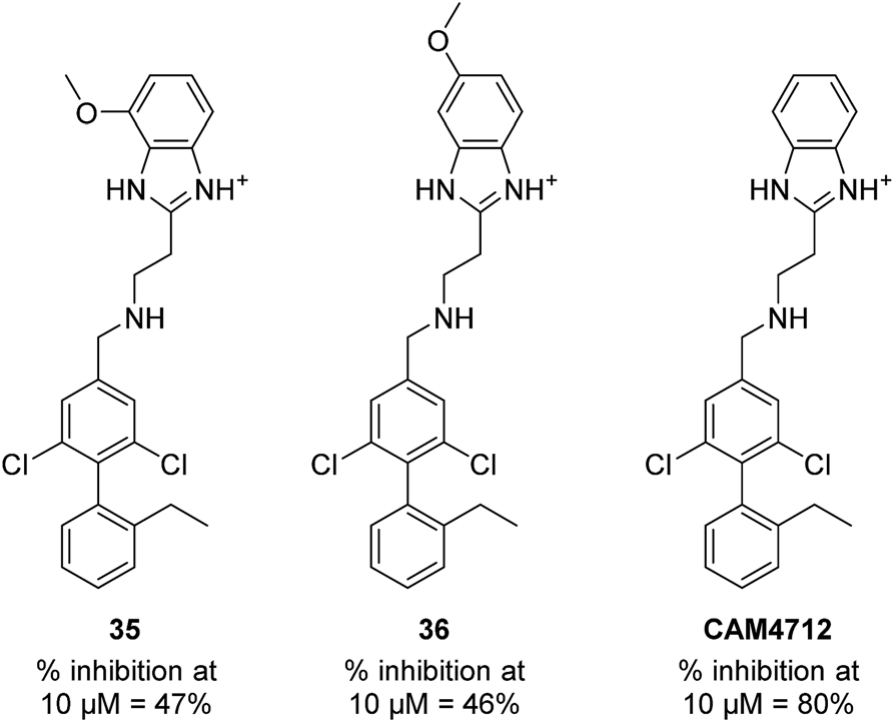
Compounds **35**, **36** and **CAM4712** with respective data.

With the optimisation around each aromatic ring in hand, compounds **35** and **36** were designed *via* a merging strategy so that they contained the most promising substitution patterns. The methoxy derivatives **35** and **36** were synthesized and tested but, disappointingly, gave worse results than **CAM4712** (Fig. 6).

#### Validation of **CAM4712**

As **CAM4712** was the most advanced compound in this series a more detailed investigation was performed. Firstly, **CAM4712** showed an improved IC_50_ of 7 μM compared to the compound **22** (58 μM). This data validated our fragment merging strategy to improve upon both affinity and inhibition. Unfortunately, as with the other compounds in the series, once the benzimidazole group had been added **CAM4712** was not soluble enough for ITC. Therefore, an ITC competition study was performed to confirm the binding mode and to estimate the affinity of **CAM4712** for the αD site (an overview of the results of the ITC experiments can be found in Table S1†). In order to achieve this, several probe molecules that have well characterised binding modes and affinities were titrated into CK2α in the presence of **CAM4712**. From these experiments, it was possible to determine not only the affinity for the αD site but also which part of the ATP site the benzimidazole group blocks.

Four probes were used for this study in four separate competition experiments, each of them titrated into CK2α in the presence of and absence 20 μM **CAM4712** giving the following results:

(1) Inhibition of binding in the αD or ATP site by **CAM4712**:

The binding of **CAM4066** to CK2α was inhibited by 20 μM **CAM4712**. From this the affinity of **CAM4712** was estimated to be 4.0 μM (Fig. S2†). This experiment confirms that **CAM4712** binds to CK2α but the binding could partially occur in the ATP site.

(2) Inhibition of binding in the αD site:

**15** was titrated into CK2α in the presence of 20 μM **CAM4712** (Fig. S3†). This showed that **CAM4712** was also able to inhibit the binding of **15** to CK2α and from this the affinity was estimated to be 5.0 μM. As **15** binds in the αD site this experiment confirms that the binding site of **CAM4712** is the αD pocket as well as confirming the affinity.

(3) Inhibition of binding to Lys68:

2-Hydroxyl, 5-methyl benzoic acid (**37**) binds to the conserved Lys68 in the ATP site and occupies the right-hand side of the pocket (PDB: ; 5CSP). The binding of compound **37** was not inhibited by **CAM4712** (Fig. S6†) confirming that the benzimidazole ring does not interact with the right-hand side of the ATP pocket and validates the binding mode derived from the crystal structure (PDB: ; 5OTZ). This result predicted that it would be possible to generate a crystal structure of **CAM4712** and **37** bound simultaneously to CK2α and this was confirmed by a crystal structure showing both compounds binding simultaneously to CK2α (Fig. S6d,† PDB: ; 6EHK).

(4) Inhibition of binding in the ATP site/hinge region:

**CX4945**, which from the analyses of crystal structures would clash with **CAM4712** in the hinge region, was titrated into CK2α in the presence of **CAM4712** (Fig. S7†). **CAM4712** was shown to inhibit the binding of **CX4945** to CK2α. The affinity of **CAM4712** for CK2α was estimated to be 3.0 μM. This confirms that the benzimidazole ring binds in the Met163 channel and blocks access to the ATP site as this would inhibit the binding of **CX4945**.

In summary, these competition experiments suggest firstly that the *K*_d_ of **CAM4712** towards CK2α is approximately 4 μM. Secondly, they confirm that the binding mode of **CAM4712** in the αD pocket and mouth of the ATP site corresponds to that seen in the crystal structure.

The validation experiments of **CAM4712** and the crystal structures allowed us to rationalise the difference in *in vitro* potency between **CAM4066** and **CAM4712** (IC_50_ 7 μM and 0.37 μM respectively). Whilst the binding of both compounds to CK2 resulted in the flipping of the Met163, **CAM4712** did not H-bond the conserved Lys68 in the ATP binding site. Instead, a low-energy hydrophobic π–π interaction between the His160 and the benzimidazole was introduced (as shown for the related compound **22** in Fig. 5f) resulting in loss of binding affinity and potency compared to **CAM4066**.

**Fig. 7:**
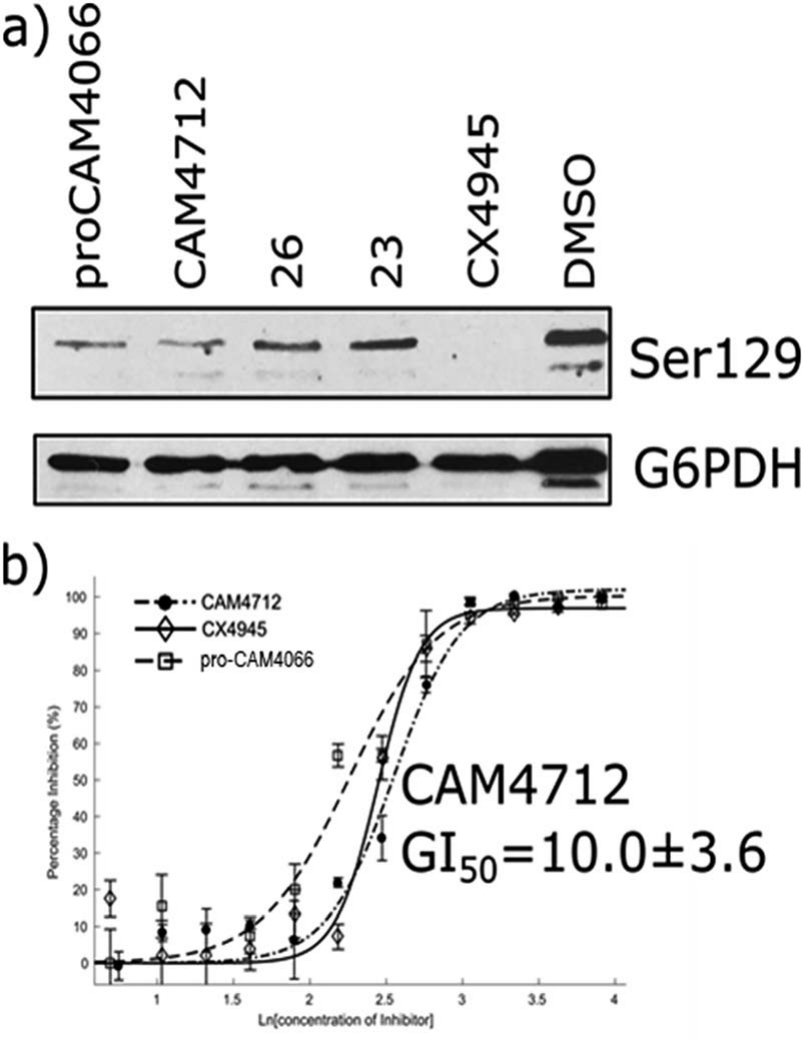
(a) Western blot analysis showing the specific CK2 phosphorylation target: AKT1 serine 129. HCT116 cells were treated with 2 × GI_50_ of **CX4945** (20 μM), **CAM4712** (20 μM) or **pro-CAM4066** (20 μM) for 72 hours (b) dose response curve for the inhibition of the growth of HCT116 cells by **CAM4712**, **pro-CAM4066** and **CX4945**. All graphs show the mean ± SEM of not less than three independent experiments with each in triplicate.

The efficacy of **CAM4712** in cellular assays was tested in HCT116 cell line, which is known to overexpress CK2α. A cell growth inhibition assay yielded a GI_50_ for **CAM4712** of 10.0 ± 3.6 μM, which is similar to that of the clinical trials candidate **CX4945** (11.3 ± 1.2 μM). It is also similar to **pro-CAM4066** (GI_50_ 9.1 ± 1.4 μM, IC_50_ 0.37 μM),^18^ but, importantly, no drop-off in potency was observed from the functional to the cellular assay. This represents a large step forward compared to **CAM4066**, which had to be administered as the prodrug **pro-CAM4066**. The target engagement by **CAM4712** was analysed by following the CK2α dependent phosphorylation of Ser129 of Akt1. This showed good inhibition of the phosphorylation of Ser129 by **CAM4712** as well as by its close analogues **23** and **26** which confirms that these compounds inhibit CK2α in the cellular environment (Fig. 7).

**CAM4712** showed a 10-fold decrease in potency compared to **CAM4066** and therefore the selectivity of **CAM4712** was screened against a panel of 140 kinases at a concentration of 30 μM (4 × IC_50_). **CAM4712** showed good selectivity against the 20 closely related CMGC kinases in the panel (Fig. S6a†). However, 4 kinases (CAMK1, SmMLCK, EF2K and SGK1) were inhibited by **CAM4712** for more than 50% (Fig. S8b†) and hence, **CAM4712** showed a reduced overall selectivity compared to **CAM4066** (which was screened at 2 μM).^19^ This is not surprising considering the high concentration used in the selectivity screen due to **CAM4712** being less potent than **CAM4066**. Nevertheless, **CAM4712** showed a more selective profile than other CK2α inhibitors (Fig. S8b†) and represents, therefore, a good starting point for further development of selective CK2α inhibitors. Our aim in this work has not been to gain specificity but rather to demonstrate mechanistically that an inhibitor that is not making significant contacts with the conserved active site is able to inhibit the kinase effectively. To fully exploit the selectivity that αD binding offers, further optimization of **CAM4712** is needed to increase its affinity towards CK2α.

One of the aims of this work was to generate an improved inhibitor compared to the previous compound **CAM4066**. We managed to design a compound with the physico-chemical properties falling into the range for bioavailable compounds according to Lipinski's rules:^21^ the number of rotatable bonds was reduced, the amide groups were removed and the compound entered the cells and showed activity without the use of a prodrug. Moreover, the number of H-bond donors and acceptors was reduced to 2 and the molecular weight was kept below 500 Da (Table 6). This was all achieved without interacting with the deep and conserved ATP binding site.

**Table 6:** Properties and structural features pf compound **CAM4712** compared to **CAM4066**^*a*^

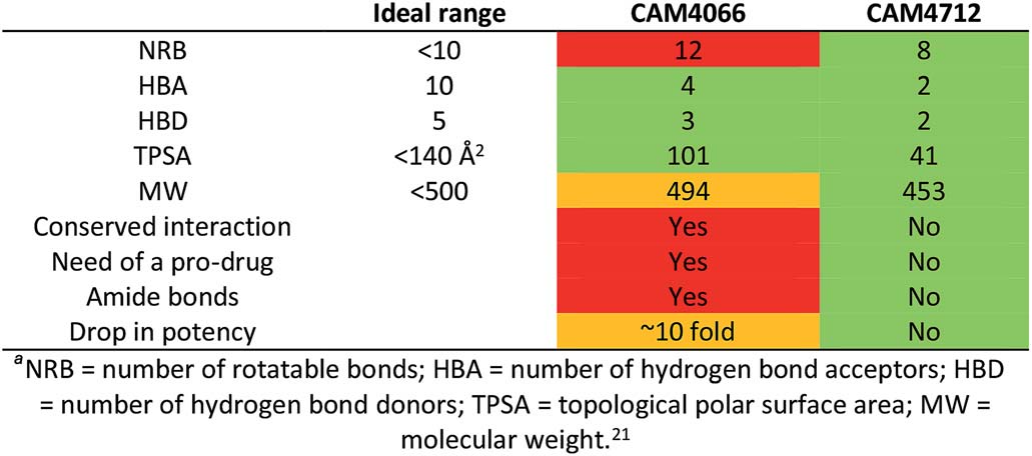			

## Conclusions

In conclusion, we have developed a series of second-generation CK2α inhibitors that target the αD site. This was achieved by first optimising the fragments that bound in the αD site, followed by identification of groups that grow towards the mouth of the ATP site to provide potent inhibitors of CK2α. In our previous work we demonstrated that selectivity could be achieved anchoring the inhibitors in the αD pocket and with this work we achieve inhibition with ligands that do not target the active site. **CAM4712** showed high cellular activity (10.0 ± 3.6 μM) and target engagement was demonstrated. This second generation of αD pocket inhibitor overcomes the limitations of our first inhibitor, including the fact that it does not need to be administered as a pro-drug to exert anti-proliferative activity. We have also shown that it is not necessary to interact with the ATP site directly, but effective inhibition of the kinase and displacement of ATP can be achieved by blocking the mouth of the ATP site with no need to interact with conserved features of the ATP binding site. These results demonstrate an entirely new approach to CK2α inhibition and will allow the future development of drug-like molecules, lead compounds and chemical tools that utilise the novel properties of the αD site.

## Conflicts of interest

There are no conflicts to declare.

## Supplementary Material

Supplementary information

## References

[cit1] Battistutta R., Lolli G. (2011). Mol. Cell. Biochem..

[cit2] Niefind K., Guerra B., Ermakowa I., Issinger O. G. (2001). EMBO J..

[cit3] Meggio F., Pinna L. A. (2016). FASEB J..

[cit4] Zhang J., Yang P. L., Gray N. S. (2009). Nat. Rev. Cancer.

[cit5] Nitta R. T., Gholamin S., Feroze A. H., Agarwal M., Cheshier S. H., Mitra S. S., Li G. (2015). Oncogene.

[cit6] Ruzzene M., Pinna L. A. (2010). Biochim. Biophys. Acta.

[cit7] Zhou B., Ritt D. A., Morrison D. K., Der C. J., Cox A. D. (2016). J. Biol. Chem..

[cit8] Di Maira G., Brustolon F., Bertacchini J., Tosoni K., Marmiroli S., Pinna L. A., Ruzzene M. (2007). Oncogene.

[cit9] Duncan J. S., Litchfield D. W. (2008). Biochim. Biophys. Acta.

[cit10] Sarno S., Pinna L. A. (2008). Mol. BioSyst..

[cit11] Battistutta R., Cozza G., Pierre F., Papinutto E., Lolli G., Sarno S., O'Brien S. E., Siddiqui-Jain A., Haddach M., Anderes K., Ryckman D. M., Meggio F., Pinna L. A. (2011). Biochemistry.

[cit12] Guerra B., Hochscherf J., Jensen N. B., Issinger O.-G. (2015). Mol. Cell. Biochem..

[cit13] Götz C., Gratz A., Kucklaender U., Jose J. (2012). Biochim. Biophys. Acta.

[cit14] Siddiqui-Jain A., Drygin D., Streiner N., Chua P., Pierre F., O'Brien S. E., Bliesath J., Omori M., Huser N., Ho C., Proffitt C., Schwaebe M. K., Ryckman D. M., Rice W. G., Anderes K. (2010). Cancer Res..

[cit15] Chon H. J., Bae K. J., Lee Y., Kim J. (2015). Front. Pharmacol..

[cit16] Pierre F., Chua P. C., O'Brien S. E., Siddiqui-Jain A., Bourbon P., Haddach M., Michaux J., Nagasawa J., Schwaebe M. K., Stefan E., Vialettes A., Whitten J. P., Chen T. K., Darjania L., Stansfield R., Anderes K., Bliesath J., Drygin D., Ho C., Omori M., Proffitt C., Streiner N., Trent K., Rice W. G., Ryckman D. M. (2011). J. Med. Chem..

[cit17] Kim H., Choi K., Kang H., Lee S. Y., Chi S. W., Lee M. S., Song J., Im D., Choi Y., Cho S. (2014). PLoS One.

[cit18] Brear P., De Fusco C., Georgiou K. H., Francis-Newton N. J., Stubbs C. J., Sore H. F., Venkitaraman A. R., Abell C., Spring D. R., Hyvönen M. (2016). Chem. Sci..

[cit19] De Fusco C., Brear P., Iegre J., Georgiou K. H., Sore H. F., Hyvönen M., Spring D. R. (2017). Bioorg. Med. Chem..

[cit20] Hopkins A. L., Keserü G. M., Leeson P. D., Rees D. C., Reynolds C. H. (2014). Nat. Rev. Drug Discovery.

[cit21] Lipinski C. A., Lombardo F., Dominy B. W., Feeney P. J. (1997). Adv. Drug Delivery Rev..

