# Supplementary material for: Second-generation CK2α inhibitors targeting the αD pocket
Source: Chem Sci. 2018 Feb 20;9(11):3041–9. doi: 10.1039/c7sc05122k (PMC5916021; doi:10.1039/c7sc05122k)
Supplement: Supplementary file 1 [file SC-009-C7SC05122K-s001.pdf]

Electronic Supplementary Material (ESI) for Chemical Science.
This journal is © The Royal Society of Chemistry 2018

Supplementary Information

Second generation CK2α inhibitors targeting the αD pocket

Jessica Iegre,‡ Paul Brear,‡ Claudia De Fusco,‡ Masao Yoshida, Sophie L. Mitchell, Maxim Rossmann, Laura Carro Santos, Hannah F. Sore, Marko Hyvönen* and David R. Spring*

Table of contents

Figure S1	3
Figure S2	4
Figure S3	5
Figure S4	6
Figure S5	6
Figure S6	7
Figure S7	9
Figure S8	10
Material and methods	11
Protein expression and purification	11
X-ray crystallography	11
ITC	12
Kinase assay	12
Cell culture	12

Growth inhibition assay	12
Western blotting	13
General synthetic experimental details	14
General synthetic methods	16
Compound overview scheme	19
Compounds and characterisation	21
Compound spectra	57
Table S1: Enthalpic and entropic contribution to Kd	122
Table S2: Crystallisation and soaking conditions	124
Table S3	125

FIGURE S1

Figure S1a

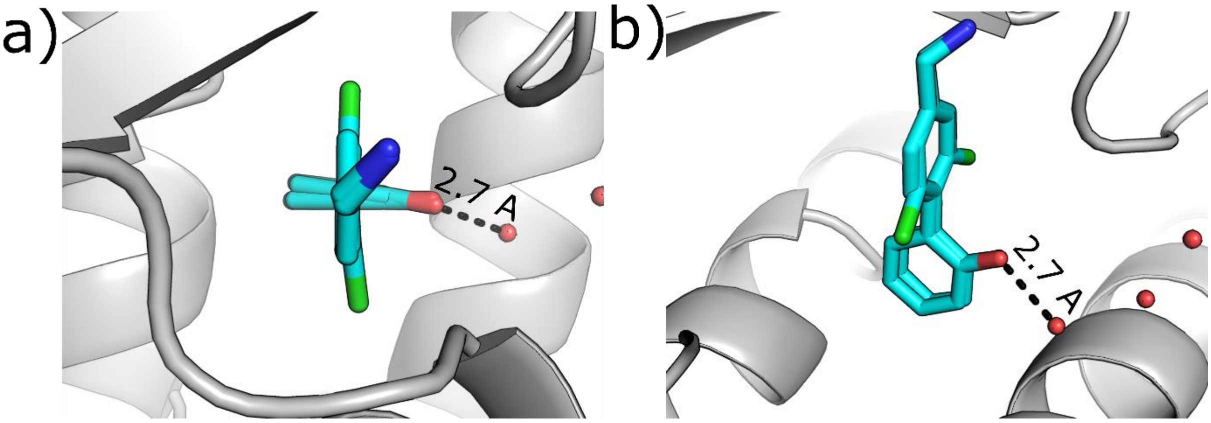

The crystal structure showing the two binding modes of **7** in the αD site of CK2α. The hydrogen bonding between the OH group and a conserved water in the Tyr125 binding pocket of CK2α are highlighted.

Figure S1b

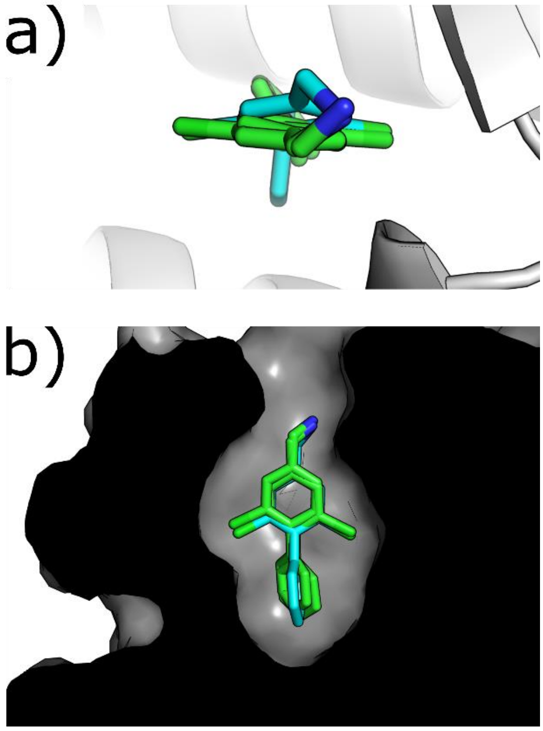

a) The top view of the crystal structure showing the two binding modes of **1** in the αD site with the one binding mode of **14** superimposed upon the structure. b) The surface view of the two binding modes of **1** from the side with the one binding mode of **14** superimposed upon it.

<u>FIGURE S2</u>

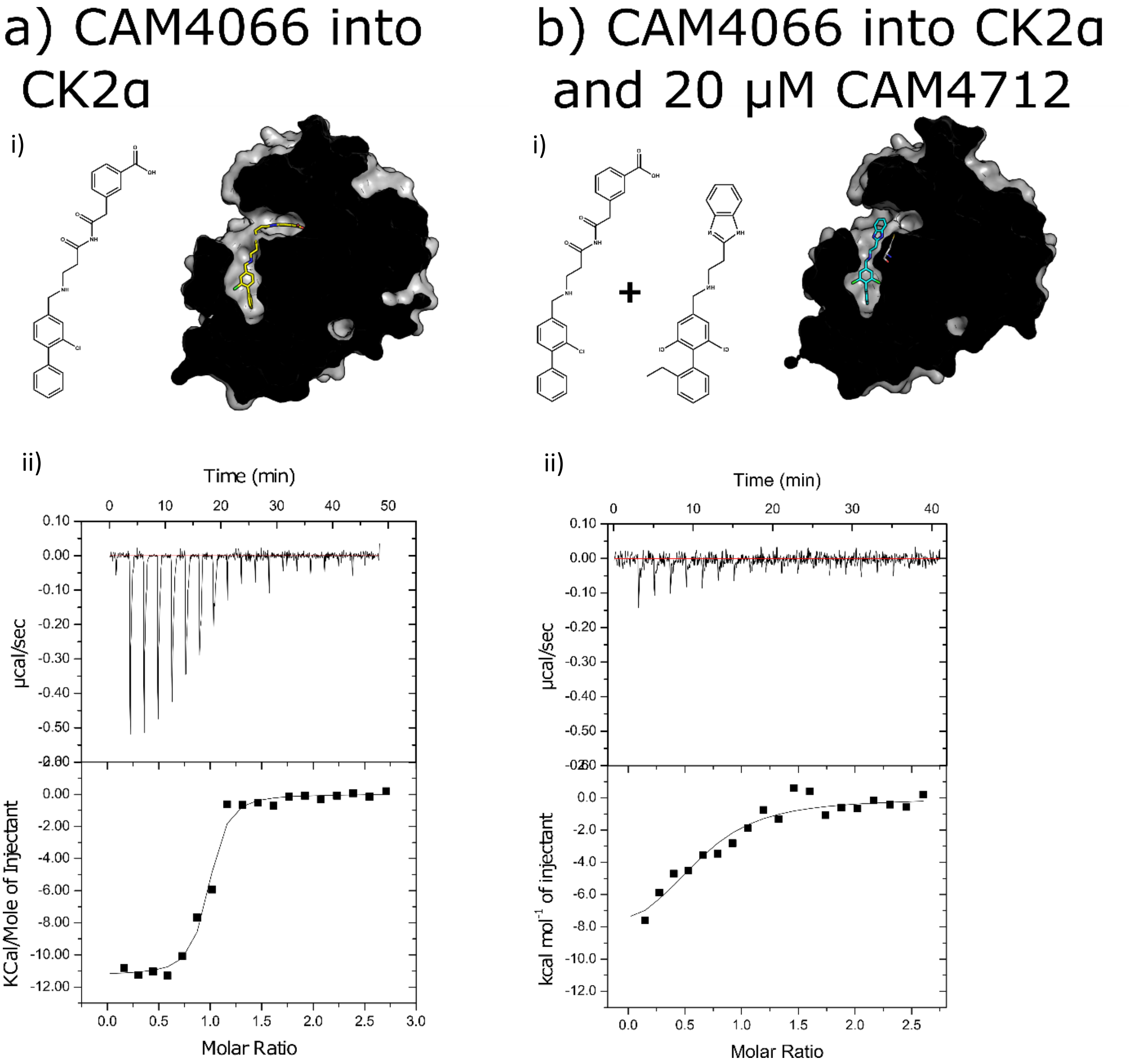

a) **CAM4066** into CK2α. i) The crystal structure of **CAM4066** bound in the αD pocket of CK2α. ii) The binding isotherm of **CAM4066** titrated into CK2α binding in the αD site. b) **CAM4066** into CK2α and 20 µM **CAM4712**. i) The crystal structure of **CAM4712** showing how it blocks the binding of **CAM4712**. ii) The binding isotherm of **CAM4066** titrated into CK2α and 20 µM **CAM4712** binding in the αD site. The presence of **CAM4712** reduces the apparent affinity of **CAM4066** for the αD site.

FIGURE S3

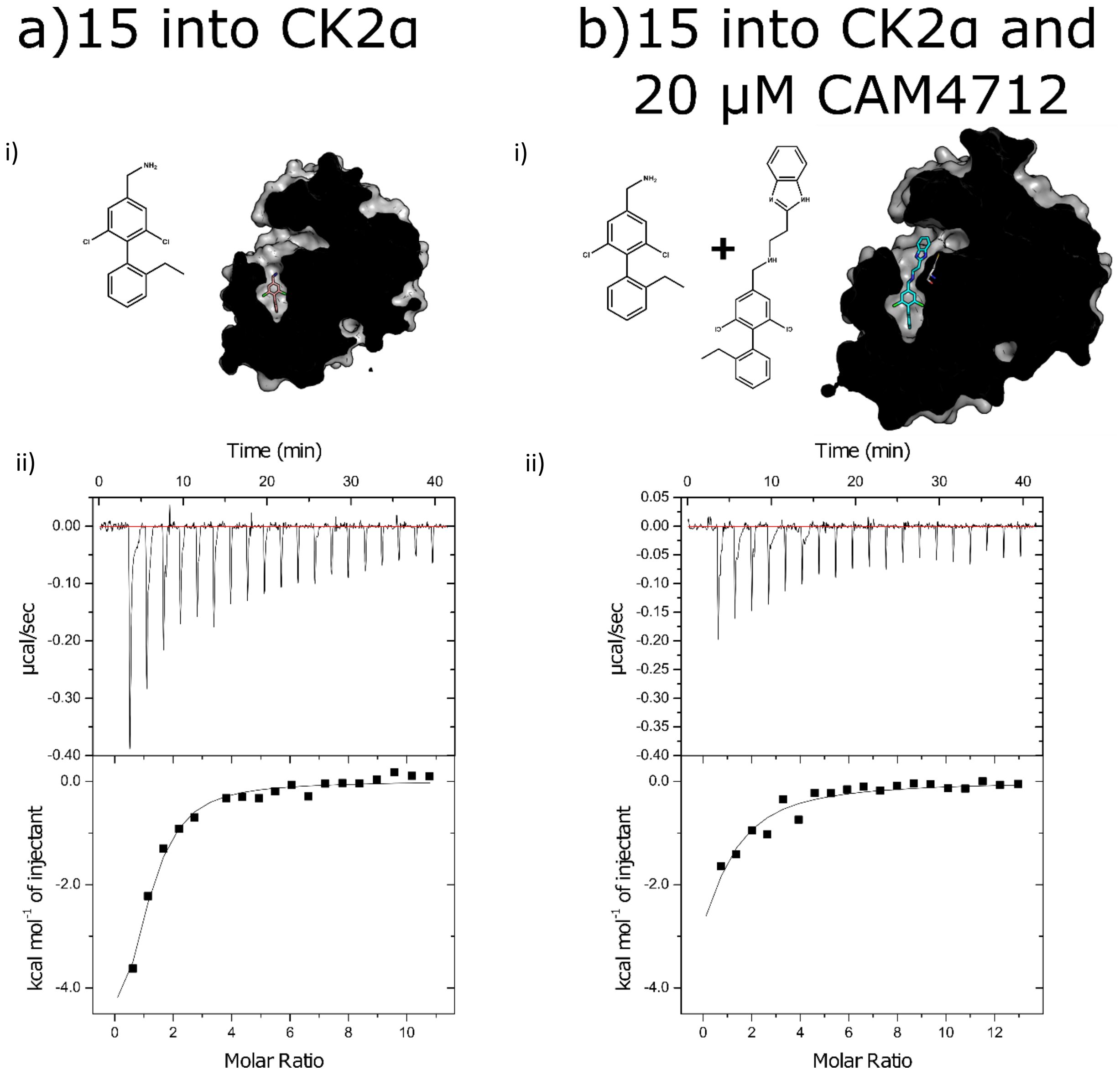

a) **15** into CK2α. i) The crystal structure of **15** bound in the αD pocket of CK2α. ii) The binding isotherm of **15** titrated into CK2α binding in the αD site. b) **15** into CK2α and 20 µM **CAM4712**. i) The crystal structure of **CAM4712** showing how it blocks the binding of **15**. ii) The binding isotherm of **15** titrated into CK2α and 20 µM **CAM4712** binding in the αD site. The presence of **CAM4712** reduces the apparent affinity of **15** for the αD site.

FIGURE S4

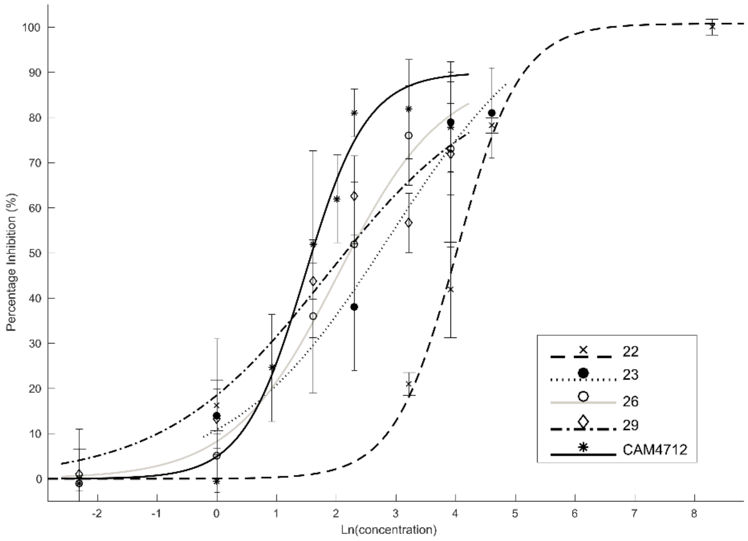

Dose-response curves of compounds **22**, **23**, **26**, **29** and **CAM4712** for CK2 inhibition. It should be noted that we the exception of compound **22**, the compounds were not soluble under the assay conditions, at concentration greater than 50 µM and therefore 100% inhibition was not achieved. All graphs show the mean ± SEM of not less than three independent experiments with each in triplicate.

FIGURE S5

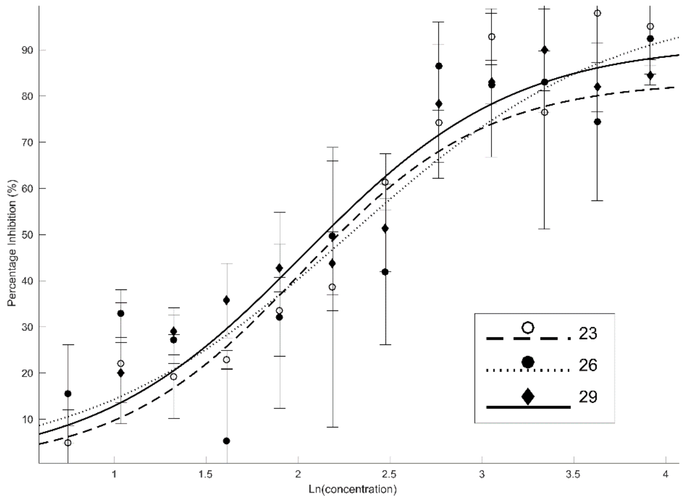

Dose-response curves of compounds **23**, **26**, **29** for anti-proliferative inhibition of HCT116 cells. All graphs show the mean ± SEM of not less than three independent experiments with each in triplicate.

FIGURE S6

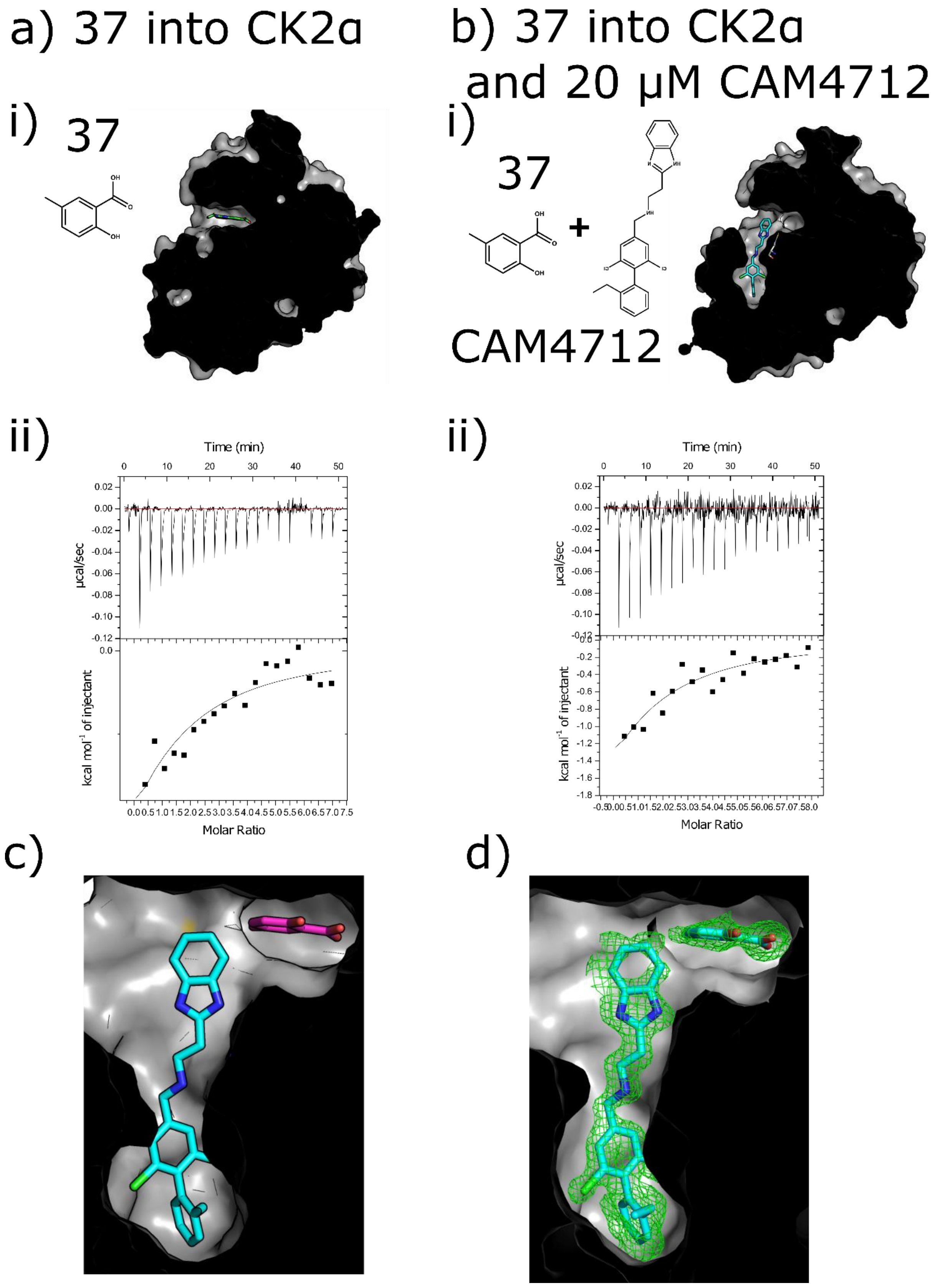

a) 37 into CK2α
b) 37 into CK2α and 20 µM CAM4712
i)
37
i)
37
+
CAM4712
ii)
ii)
Time (min)
µcal/sec
kcal mol-1 of injectant
Molar Ratio
c)
d)

a) **37** into CK2α. i) The crystal structure of **37** bound in the αD pocket of CK2α. ii) The binding isotherm of **37** titrated into CK2α. b) **37** into CK2α and 20 μM **CAM4712**. i) The crystal structure of **CAM4712** showing how it binds to CK2α. ii) The binding isotherm of **37** titrated into CK2α and 20 μM **CAM4712** binding in the αD site. The presence of **CAM4712** does not significantly change the affinity of **37** for the αD site. c) The structure of **CAM4712** (light blue) bound in the αD site with the structure of **37** (purple) bound in the ATP site superimposed. d) The cocrystal structure of **CAM4712** and **37** binding simultaneously to CK2α. The Fo-Fc map is shown contoured at 1.6σ.

FIGURE S7

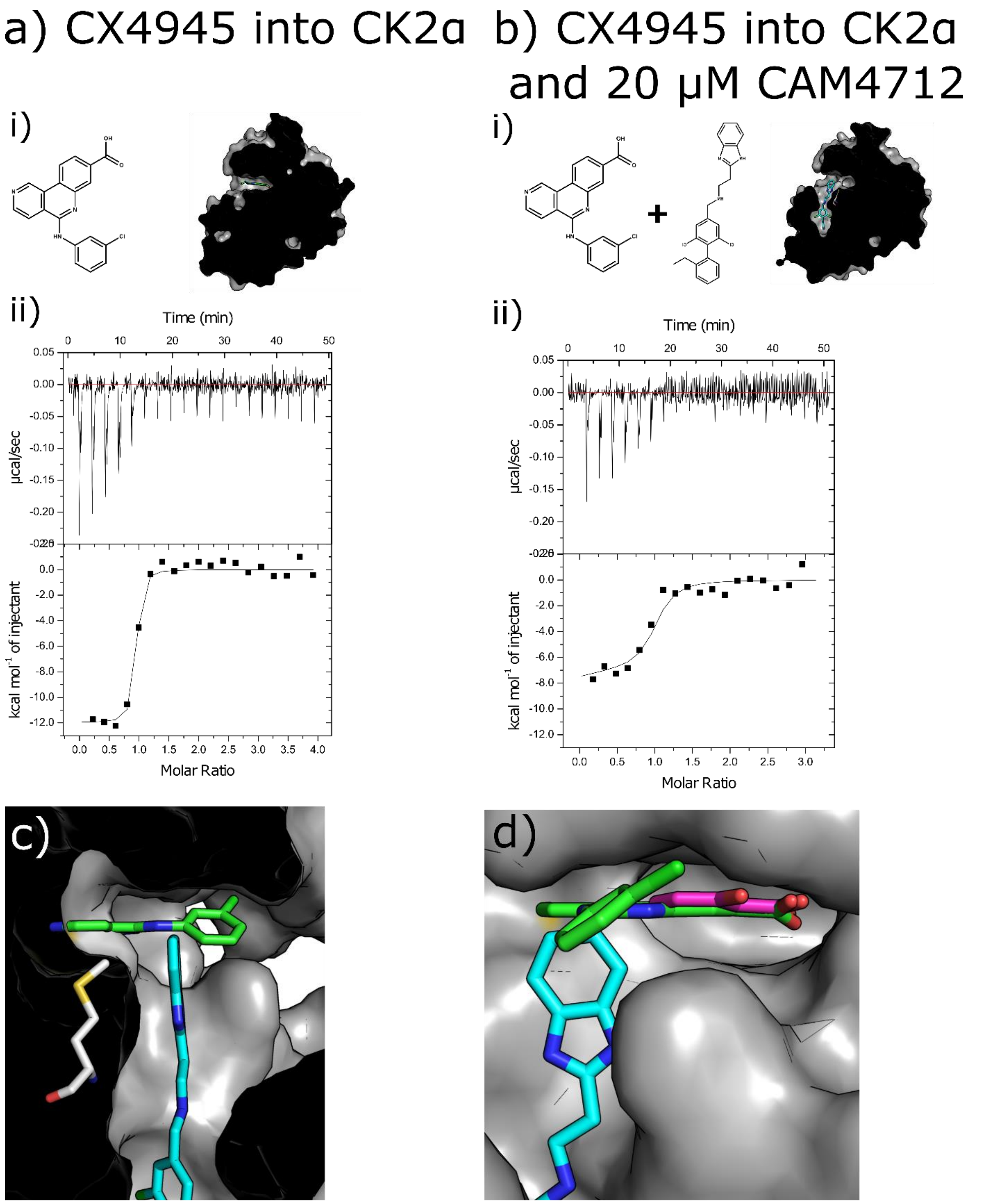

a) **CX4945** into CK2α. i) The crystal structure of CX4945 (PDB :3nga)[1] bound in the αD pocket of CK2α. ii) The binding isotherm of **CX4945** titrated into CK2α binding in the ATP site. b) **CX4945** into CK2α and

---

[1]A. D. Ferguson, P.R. Sheth, A. D. Basso, S. Paliwal, K. Gray, T. O. Fischmann, H. V. Le, *Febs Lett.*, **2011**, 585, 104-110
[2] H. Kim, K. Choi, H. Kang, S. Y. Lee, S. W. Chi, M. S. Lee, J. Song, D. Im, Y. Choi and S. Cho, PLoS One, **2014**, 9, 1–8.
[3] B. Guerra, J. Hochscherf, N. B. Jensen and O.-G. Issinger, Mol. Cell. Biochem., **2015**, 406, 151–161.
[4] C. Gotz, A. Gratz, U. Kucklaender and J. Jose, Biochem.Biophys. Acta, **2012**, 1820, 970–977

20 µM **CAM4712**. i) The crystal structure of **CAM4712** showing how it binds to CK2α. ii) The binding isotherm of **CX4945** titrated into CK2α and 20 µM **CAM4712**. The presence of **CAM4712** reduces the apparent affinity of **CX4945** for the αD site. c) The structure of **CAM4712** (light blue) bound in the αD site with the structure of **CX4945** (green) bound in the ATP site superimposed. d) The structure of **CAM4712** (light blue) bound in the αD site with the structure of **CX4945** (green) and **37** bound in the ATP site superimposed.

FIGURE S8

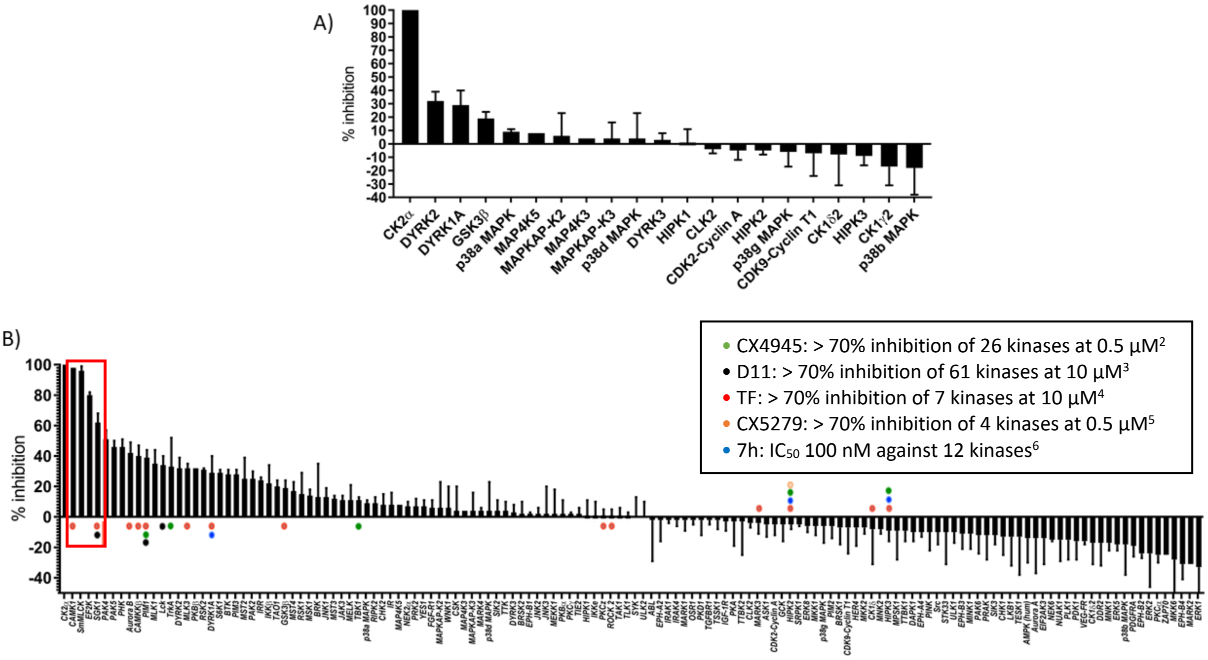

A) Selectivity profile of **CAM4712** at 30 µM concentration against closely related CMGC family members; B) Selectivity profile of **CAM4712** at 30 µM against a panel of 140 kinases. Kinases inhibited by **CAM4712** for more than 50% are highlighted in red. Kinases inhibited by other CK2 kinase inhibitors (CX4945, D11, TF, CX5279, 7h) are shown as coloured dots.

[5] R. Battistutta, G. Cozza, F. Pierre, E. Papinutto, G. Lolli, S. Sarno, S. E. O'Brien, A. Siddiqui-Jain, M. Haddach, K. Anderes, D. M. Ryckman, F. Meggio and L. A. Pinna, Biochemistry, **2011**, 50, 8478–8488

[6] J. E. Dowling, M. Alimzhanov, L. Bao, C. Chuaqui, C. R. Denz, E. Jenkins, N. A. Larsen, P. D. Lyne, T. Pontz, Q. Ye, G. A. Holdgate, L. Snow, N. O'Connell and A. D. Ferguson, ACS Med. Chem. Lett., **2016**,7, 300–305

MATERIAL AND METHODS

Protein expression and purification

Three constructs of CK2α were used in this study. For ITC and kinase activity assays CK2α_WT was used (residues 2-329). For crystallization purposes two different constructs were used: CK2α_KA and CK2α_FP10. CK2α_KA (residues 2-329) contained four mutations designed to aid crystallization by reducing the overall charge of the protein; R21S, K74A, K75A and K76A. CK2α_FP 10 contained one mutation (R21S) and an N-terminal extension GSMDIEFDDDADDDGSGSGSGSGS aimed at mimicking a substrate peptide for CK2α. CK2α_FP10 was cloned into pHAT4 vector and CK2α_KA was cloned into pHAT2 vector to give constructs with cleavable His6-tags. Recombinant plasmids containing one of the three constructs (CK2α_WT/ CK2α_KA/ CK2α_FP10) were introduced into Escherichia coli BL21(DE3) for protein production. Single colonies of the cells were grown in 6x1L of 2xTY with 100 µg/mL ampicillin at 37°C. Isopropylthio-β-D-galactopyranoside (IPTG) was added to a final concentration of 0.4 mM to induce expression when the optical density at 600 nm reached 0.6. The cells were incubated overnight at 25°C then harvested by centrifugation at 4,000 g for 20 minutes. The same extraction and purification procedure was used for all four constructs, with the exception that CK2α_KA used 350 mM NaCl in the buffer, whereas, CK2α_WT and CK2α_FP10 required 500 mM NaCl. The cell pellets were suspended in 20 mM Tris, 350/50 mM NaCl, pH 8.0) and lysed using a high pressure homogenizer. Protease inhibitor cocktail tablets (one tablet per 50 mL extract; Roche Diagnostics) and DNase I were then added. The crude cell extract was then centrifuged at 10,000 g for 45 minutes, the supernatant was filtered with a 0.22 µm filter. The soluble supernatant was applied on a Ni Sepharose Fast Flow6 column at pH 8.0, washed and eluted in 20 mM Tris pH 8.0, 350/500 mM NaCl, 200 mM imidazole. After overnight dialysis into 20 mM Tris, pH 8.0, 350/500 mM NaCl the N-terminal His6-Tag was cleaved overnight by TEV protease and passed through a second metal affinity column to remove uncleaved protein and the protease. The cleaved protein was further purified on a Sepharose Q HP anion-exchange column and the main peak fraction from this column was further purified by gel filtration on a Superdex 75 16/60 HiPrep column equilibrated with Tris 20 mM, pH 8.0, 350/500 mM NaCl. Pure protein was concentrated to 15 mg/mL and flash frozen in liquid nitrogen.

X-ray crystallography

CK2α_KA at 5 mg/mL in 20 mM Tris, pH 8.0, 350 mM NaCl, 1 mM DTT, and 25 mM ATP was crystallised with 112.5 mM MES pH 6.5, 35% glycerol ethoxylate and 180 mM ammonium acetate in a 1:1 ratio with a total volume of 2 µL by the hanging drop vapour-diffusion method. The fragments were soaked as singletons at 2-100 mM into these crystals for 15–20 h in 107 mM MES pH 6.5, 35% glycerol ethoxylate and 1.04 M ammonium acetate after which the crystals were cryo-cooled in liquid nitrogen for data collection.CK2α_FP10 at 10 mg/mL in 20 mM Tris, pH 8.0, 500 mM NaCl, 4 mM DTT, 13 mM ATP, 2 mM phytic acid was crystallised with 107 mM MES, pH 6.5, 29% glycerol ethoxylate, 1.04 M ammonium acetate in a 1:1 ratio with a total volume of 2 µL by the hanging drop vapour-diffusion method. The fragments were soaked into the crystals of CK2α_FP10 for 15–20 h at 100 mM in 107 mM MES pH 6.5, 29% glycerol ethoxylate and 1.04 M ammonium acetate. The crystals were cryo-cooled in liquid nitrogen in the same solution for data collection. The crystals were cryo-cooled in liquid nitrogen in the same solution for data collection.

X-ray diffraction data was collected at the Diamond synchrotron radiation source, then processed using the pipedream package by Global Phasing Ltd; structures were solved by using programs from the CCP4 package. Models were iteratively refined and rebuilt by using AutoBuster and Coot programs. Ligand coordinates and restraints were generated from their SMILES strings using the Grade software package. All coordinates have been deposited to Protein Data Bank and accession numbers, data collection and refinement statistics are shown in Table S3, with crystallisation and soaking conditions being listed in Table S2.

ITC

All ITC experiments were performed at 25 °C using a MicroCal itc200 instrument (GE Healthcare). CK2α_WT (20 mg/mL, 20 mM Tris pH 8.0, 500 mM NaCl) was diluted in Tris buffer (200 mM Tris, 300 mM NaCl, 10% DMSO) and concentrated to 20-50 µM. Compounds in 100x stock solutions were diluted into the buffer ensuring that the DMSO concentrations were carefully matched. In a typical experiment CK2α_WT (40 µM) was loaded into the sample cell and 0.4-2.0 mM of the ligand was titrated in nineteen 2 µL injections of 2 s duration at 150 s intervals, with injector speed of 750 rpm. Heats of dilution were determined in identical experiments, but without protein in the cell. The data fitting was performed with a single site binding model using the Origin software package.

Kinase assays

The kinase assays were performed using the ADP-Glo™ kinase assay kit (Promega). 50 nM CK2α_WT was incubated in the kinase reaction buffer (40 mM Tris pH7.5, 200 mM NaCl, 20 mM $MgCl_2$, 0.1 mg/mL BSA, 25 µM ATP, 50 µM substrate peptide (RRRADDSDDDD, Enzo Life Sciences Inc.), 5% (v/v) DMSO) in the presence of different concentrations of the inhibitor at 25 °C for 40 min. 5 µL aliquots of the kinase reaction were quenched with 5 µL of ADP-glo™ solution. After another 40 min the kinase detection reagent was added and maintained at 25 °C for 30 minutes. The luminescence was recorded using a PHERAstar FS plate reader (BMG LABTECH) with an integration time of 1 s. Percentage inhibition was calculated relative to a DMSO control and a baseline measurement without ATP. All measurements were performed in triplicate. The IC_{50} curves were fitted using Sigma plot 11.0.

Cell culture

All cell lines used were obtained from ATCC and were supplied as mycoplasma free. HCT116 colon carcinoma cells were maintained in McCoy's 5A (1x) + Glutamax-I growth medium (Gibco, 36600-021) supplemented with fetal bovine serum (FBS, Gibco Life Technologies, 10270-106) at a final concentration of 10%. All cells were grown at 37°C / 5% CO_2 in a humidified environment and all the assays were performed using these culturing conditions.

Growth Inhibition assays

Adherent cell lines (HCT116) were seeded into flat-bottomed tissue culture 96-well plates in a volume of 150 µL of growth medium. HCT116 cells were seeded at 750 cells per well. After 24 hours, compounds dissolved in DMSO were diluted in growth medium and were added to cells such that the final DMSO concentration was 1% (v/v) and the final volume in the well was 200 µL. Cells were then incubated in the presence compound for 72 hours before fixation. Without removing supernatant 100

µL of cold 10% (v/v) trichloroacetic acid was added to each well and the plates were incubated for 30 minutes at 4 °C. After that the plates were washed three times in tap water and left to dry at room temperature. The fixed cells were stained in a 0.057% sulforhodamine B/1% acetic acid solution (w/v) and incubated at room temperature with agitation for 30 minutes after which the dye was removed and the plates washed in 1% (v/v) acetic acid and left to dry. The dye was then solubilised in 200 µL 10 mM Tris solution (pH 10.5) and incubated for 30 minutes under agitation. The 510 nm absorbance was then measured using a PHERAstar plus plate reader (BMG Labtech). Percentage of growth inhibition was calculated relative to DMSO controls and GI_{50} values were calculated using Graphpad Prism.

Western Blotting

HCT116 cells (2 mL) were seeded into 6-well tissue culture plates at a seeding density of $3x10^5$ cells/ml and cultured for 24 hours prior to the addition of compound. Compound was diluted in culture medium to the desired concentration and a final DMSO concentration of 1% (v/v). Cells were harvested, washed in PBS and the pellet collected. Cells were lysed using a NP-40 lysis buffer (50 mM Tris pH 8, 150 mM NaCl, 1% NP-40) with the addition of Proteoblock protease inhibitor (Fermentas), and the phosphatase inhibitors #2 and #3 (Sigma Aldrich) at the recommended concentrations. The cell pellet was incubated in lysis buffer on ice for 2 hours and then centrifuged for 10 minutes at 4 °C at 13000 rpm on a bench top centrifuge for 10 minutes and the supernatant collected and stored at -80 °C. Protein levels were quantified using the Pierce BCA protein assay kit (Pierce, Thermo Fisher Scientific). A total of 30 µg of protein was loaded onto a 4-12% Bis-Tris gel (Invitrogen) and run for 1 hour at a constant 200 V. The gel was transferred onto NC membrane at 4°C overnight at a constant 12 V. Transfer efficiency was confirmed by staining the membrane with Ponceau S (Sigma-Aldrich) after which it was incubated in blocking buffer (either 5% Milk-TBS-0.1% Tween20) for 1 hour. Membranes were then incubated with anti-AKT1 (phosphoS129) (Abcam, ab133458) antibodies diluted 1:3.000 in 5% BSA-TBST 0.1% Tween20 for 24 hours at 4 °C. Anti-GAPDH antibody was used as a loading control (Sigma-Aldrich, G8795) diluted in Milk-TBS-0.1% Tween20. After washing, membranes were then incubated with HRP-labelled anti-rabbit or anti-mouse antibody for 1 hour at room temperature, washed, and then visualised using ECL Clarity™ (Bio-Rad).

GENERAL SYNTHETIC EXPERIMENTAL DETAILS

Solvents: Except as otherwise indicated, reactions were carried out using oven-dried glassware under nitrogen with dry, freshly distilled solvents. THF was distilled from CaH_2 and $LiAlH_4$ in the presence of triphenylmethane. Diethyl ether was distilled from CaH_2 and $LiAlH_4$. CH_2Cl_2 and MeOH were distilled from CaH_2. All other solvents were used as obtained from commercial sources.

Materials: All reagents were used as obtained from commercial sources.

(2-Chloro-[1,1'-biphenyl]-4-yl)methanaminium chloride (**1**)[1], 2-Chloro-[1,1'-biphenyl]-4-carbaldehyde[2], 2-Chloro-4-formylphenyl trifluoromethanesulfonate[3], 4-formyl-2-methoxyphenyl trifluoromethanesulfonate[4] and 4-formyl-2-(trifluoromethoxy)phenyl trifluoromethanesulfonate[5] were synthesized according literature procedures.

TLC: All reactions were monitored by thin layer chromatography (TLC) using glass plates precoated with Merck silica gel 60 F254. Visualization was by the quenching of UV fluorescence (λ_{max} = 254 nm) or by staining with ninhydrin. Retention factors (R_f) are quoted to 0.01.

Chromatography: Flash column chromatography was carried out using slurry-packed Merck 9385 Kieselgel 60 silica gel under a positive pressure of nitrogen.

Semi-Preparative HPLC: HPLC purification was performed on an Agilent 1260 Infinity system fitted with a Supelcosil ABZ+Plus column (250 mm x 21.2 mm, 5 µm) using linear gradient systems (solvent A: 0.1% (v/v) TFA in water, solvent B: 0.05% (v/v) TFA in acetonitrile) at a flow rate of 20 mL·min^{-1}.

Infrared (IR): IR spectra were recorded on a Perkin-Elmer Spectrum One FT-IR spectrometer using a Diamant/KRS5 ATR. Selected absorption maxima (ν_{max}) are reported in wavenumbers (cm^{-1}).

NMR: Magnetic resonance spectra were processed using iNMR v. 5.5.7 (Mestrelab Research)

or TopSpin v. 3.5 (Bruker). An aryl, quaternary, or two or more possible assignments were given when signals could not be distinguished by any means. Measured coupling constants are reported for mutually coupled signals; coupling constants are labelled apparent in the absence of an observed mutual coupling, or multiplet when none can be determined.

Proton magnetic resonance spectra were recorded using an internal deuterium lock (at 298 K unless stated otherwise) on Bruker DPX (400 MHz; 1H-13C DUL probe), Bruker Avance III HD (400 MHz; Smart probe), Bruker Avance III HD (500 MHz; Smart probe) and Bruker Avance III HD (500 MHz; DCH Cryoprobe) spectrometers. Proton assignments are supported by ^{1}H-^{1}H COSY, ^{1}H-^{13}C HSQC or ^{1}H-^{13}C HMBC spectra, or by analogy. Chemical shifts (δ_H) are quoted in ppm to the nearest 0.01 ppm and are referenced to the residual non-deuterated solvent peak. Discernible coupling constants for mutually coupled protons are reported as measured values in Hertz, rounded to the nearest 0.1 Hz. Data are reported as: chemical shift, multiplicity (br, broad; s, singlet; d, doublet; t, triplet; q, quartet; m,

---

[2] Brear *et al.*, Chem. Sci., **2016**, Advance article, **DOI**: 10.1039/C6SC02335E
[3] M. I. Dawson *et.al.,* J. Med. Chem., **2007**, 50, 2622-2639, **DOI:** 10.1021/jm0613323
[4] K. Ishibashi *et. al.*, Chemical & Pharmaceutical Bulletin, **1999**, 47, 226-240, **DOI:** http://doi.org/10.1248/cpb.47.226
[5] Patent: WO 2007071638 A1

multiplet; or a combination thereof), coupling constants and number of nuclei. Diastereotopic protons are assigned as X and X’, where X’ designates the lower-field proton.

Carbon magnetic resonance spectra were recorded using an internal deuterium lock (at 298 K unless stated otherwise) on Bruker DPX (101 MHz), Bruker Avance III HD (101 MHz) and Bruker Avance III HD (126 MHz) spectrometers with broadband proton decoupling. Carbon spectra assignments are supported by DEPT editing, ^{1}H-^{13}C HSQC or ^{1}H-^{13}C HMBC spectra, or by analogy. Chemical shifts (δ_C) are quoted in ppm to the nearest 0.1 ppm and are referenced to the deuterated solvent peak. Data are reported as: chemical shift, multiplicity (if not a singlet), coupling constants and number of nuclei (if not one).

Fluorine magnetic resonance spectra were recorded on Bruker Avance III (376 MHz; QNP Cryoprobe) or Bruker Avance III HD (376 MHz; Smart probe) spectrometers. Chemical shifts (δ_F) are quoted in ppm to the nearest 0.1 ppm. Data are reported as: chemical shift, number of nuclei (if not one), multiplicity (if not a singlet), coupling constants and assignment.

HRMS: High resolution mass spectrometry was carried out with a Micromass Q-TOF or a Waters LCT Premier Mass Spectrometer using electrospray ionisation [ESI].

Melting points: These data were collected on a BÜCHI B-545 and are uncorrected.

GENERAL SYNTHETIC METHODS

General method **A**: Phenol triflation

To a solution of phenol (1.0 equiv) in anhydrous CH_2Cl_2 (~0.3 M) was added pyridine (1.6 equiv). The solution was cooled to 0 °C and trifluoromethanesulfonic anhydride (1.4 equiv) was added dropwise over 30 minutes. The reaction was allowed to warm to room temperature and stirred overnight. The volatiles were removed under reduced pressure and the residue was diluted with H_2O and extracted with EtOAc. The organic layer was washed with 10% aqueous HCl, 5% aqueous Na_2CO_3, a saturated aqueous solution of NaCl and H_2O, then dried over $MgSO_4$, filtered and concentrated *in vacuo*. The crude product was then purified by flash column chromatography to yield the desired product.

General method **B**: Suzuki-Miyaura coupling 1

A mixture of the aryl bromide (1.0 equiv.) or aryl triflate (1.0 – 1.6 equiv), appropriate boronic acid (1.0 - 1.3 equiv), $Pd(PPh_3)_4$ (0.16 - 0.05 equiv) and 2M aqueous Na_2CO_3 (1.6 - 3.0 equiv) or K_2CO_3 (2.0 equiv) were solvated with DME (0.16 – 0.7 M) and H_2O (1.4 - 2.3 M). The reaction was degassed by bubbling nitrogen through the solution for 15 minutes and then heated to reflux or heated to 100 ^oC under microwave irradiation until consumption of the starting material (1-7 hours) by TLC monitoring. The reaction was allowed to cool to room temperature, filtered through celite washing with Et_2O and the solvent removed under reduced pressure. The residue was dissolved in CH_2Cl_2/H_2O and extracted three times with CH_2Cl_2. The combined organic extracts were washed with a saturated aqueous solution of NaCl, dried over $MgSO_4$, filtered and concentrated *in vacuo*. The crude product was then purified by flash column chromatography to yield the desired product.

General method **C**: Suzuki-Miyaura coupling 2

A mixture of the aryl bromide (1.0 - 1.2 equiv) or aryl triflate (1.0 equiv.), appropriate boronic acid (1.0 – 1.2 equiv), $PdCl_2(dppf){\cdot}CH_2Cl_2$ (0.05 equiv) and K_3PO_4 (1.20 – 2.0 equiv) were solvated with DME (0.2 – 0.3 M), EtOH (1.3 – 1.7 M) and H_2O (2.0 – 2.5 M). The reaction mixture was degassed by bubbling nitrogen through the solution for 15 minutes and then heated to reflux or heated to 110 ^oC under microwave irradiation until consumption of the starting material (1-6 hours) by TLC monitoring. The reaction was allowed to cool to room temperature, filtered through celite washing with Et_2O and the solvent removed under reduced pressure. The residue was dissolved in Et_2O/H_2O and extracted three times with Et_2O. The combined organic extracts were washed with a saturated aqueous solution of NaCl, dried over $MgSO_4$, filtered and concentrated *in vacuo*. The crude product was then purified by flash column chromatography to yield the desired product.

General method **D**: Suzuki-Miyaura coupling 3

A mixture of the benzaldehyde (1.0 equiv), appropriate boronic acid (2.0 equiv) and K_3PO_4 (4.0 equiv) was dissolved in 1,4-dioxane (0.25 M). The reaction mixture was degassed by bubbling nitrogen through the solution for 15 minutes before the addition of $PdCl_2(dppf)$ (0.10 equiv). The reaction mixture was then heated to 90^oC until consumption of the starting material (2.5-6 hours) by TLC monitoring. The mixture was then diluted with EtOAc and washed twice with H_2O. The organic layer was washed with a saturated aqueous solution of NaCl, dried over $MgSO_4$, filtered and concentrated *in vacuo*. The crude product was then purified by flash column chromatography to yield the desired

product.

General method **E**: Benzonitrile reduction

To a stirred suspension of $LiAlH_4$ (2.0 – 4.0 equiv) in Et_2O (~0.25 M) was added $AlCl_3$ (0.0 – 4.0 equiv) and the reaction mixture cooled to 0 °C for 10 minutes. The reaction was allowed to warm to room temperature and the nitrile (1.0 equiv) was added portionwise. The reaction was stirred at room temperature for 30 minutes and then heated at 50 °C overnight. After cooling to room temperature, a saturated aqueous solution of potassium sodium tartrate tetrahydrate and Et_2O were added and the mixture stirred for 1 hour. The reaction mixture was diluted with 2M aqueous $NaCO_3$ and extracted three times with Et_2O. The combined organic extracts were washed with a saturated aqueous solution of NaCl, dried over $MgSO_4$, filtered and concentrated *in vacuo*. The crude product was then purified by flash column chromatography or semi-preparative HPLC to yield the desired product.

General method **F**: Reductive amination of free amines

The benzaldehyde (1.0 equiv) and the amine (1.3 - 1.5 equiv) were combined in anhydrous 1,2-dichloroethane (0.28 M) under an atmosphere of nitrogen and stirred for 2 hours. Sodium triacetoxyborohydride (1.4 equiv) was added in two portions with a 30 minute interval and the reaction was stirred at room temperature for 18 hours. The reaction mixture was poured into 2M aqueous Na_2CO_3 and extracted three times with CH_2Cl_2. The combined organic extracts were washed with a saturated aqueous solution of NaCl, dried over $MgSO_4$, filtered and concentrated *in vacuo*. The crude product was then purified by flash column chromatography to yield the desired product.

General method **G**: Reductive amination of ammonium salts

A solution of the ammonium salt (1.5 equiv) in MeOH (0.28 M) was treated with NEt_3 (2.0 equiv) and aldehyde (1.0 equiv) and the mixture stirred at room temperature for 2 hours. Sodium triacetoxyborohydride (1.4 equiv) was added in two portions with a 30 minute interval and the reaction was stirred at room temperature for 18 hours. The reaction mixture was poured into 2 M aqueous Na_2CO_3 and extracted three times with CH_2Cl_2. The combined organic extracts were washed with a saturated aqueous solution of NaCl, dried over $MgSO_4$, filtered and concentrated *in vacuo*. The crude product was then purified by flash column chromatography to yield the desired product.

General method **H**: Benzimidazole condensation

The appropriate carboxylic acid (1.0 equiv), diamine (2.0 equiv), HBTU (2.0 equiv) and NEt_3 (3.0 equiv) were combined in anhydrous DMF (0.09 M) under at atmosphere of nitrogen and stirred for 2.5 hours. The reaction mixture was diluted with EtOAc and washed with H_2O, a saturated aqueous solution of $NaHCO_3$ and a saturated aqueous solution of NaCl before drying over Na_2SO_4, filtration and concentrated *in vacuo*. The crude product was purified by flash column chromatography and the resulting residue dissolved in acetic acid (0.09 M) and heated to 70 °C overnight. Following cooling to 0 °C, the reaction mixture was poured into a saturated aqueous solution of $NaHCO_3$ and extracted three times with EtOAc. The combined organic extracts were washed with a saturated aqueous solution of NaCl, dried over Na_2SO_4, filtered and concentrated *in vacuo*. The crude product was then purified by flash column chromatography to yield the *N*-boc-protected product.

General method **J**: Boc-deprotection and salt formation

A solution of protected amine (1.0 equiv) in HCl (4M in 1,4-dioxane) (~100 equiv) was stirred at room temperature for 3 hours. The volatiles were removed *in vacuo* and the residue triturated with Et_2O to yield the desired product.

COMPOUND OVERVIEW SCHEME

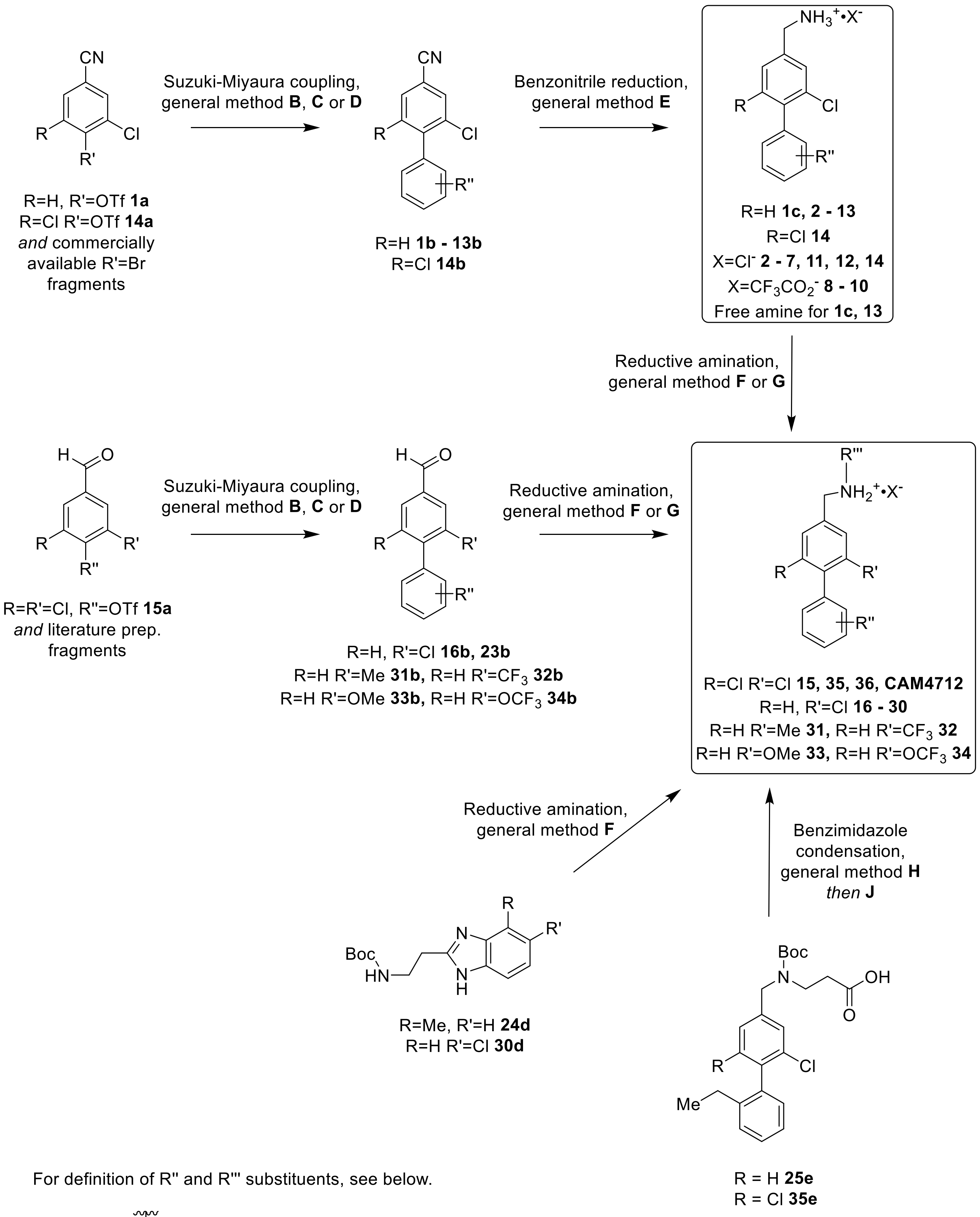

For definition of R'' and R''' substituents, see below.

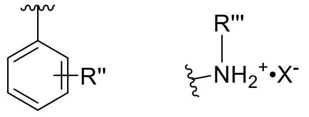

Definition for R'' substituent as follows:

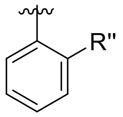

R''=H **1b, 14b, 16b, 1c, 14, 16 - 22**
R''=Me **2b, 2**
R''=Et **3b, 23b, 31b - 34b, 3, 15, 23 - 36, CAM4712**
R''=^{i}Pr **4b, 4**
R''=OMe **5b, 5**
R''=F **6b, 6**
R''=OH **7b, 7**

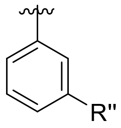

R''=Me **8b, 8**
R''=F **9b, 9**
R''=CN **10b**
R''=$CH_2NH_3^+$•$CF_3CO_2^-$ **10**

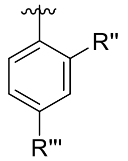

R''=Me, R'''=F **11b, 11**

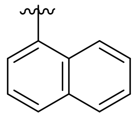

12b *and* **12**

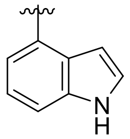

13b *and* **13**

Definition for R''' substituent as follows:

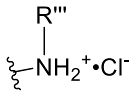

R'''=Bn **16**
R'''=CH_2Bn **17**
R'''=CH_2CH_2Bn **18**

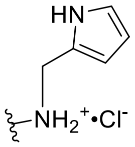

19

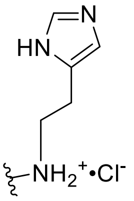

20

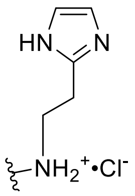

21

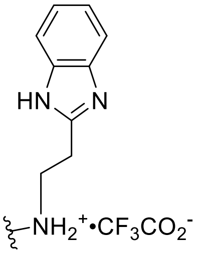

CAM4712

Definition for substitution when R''' = benzimidazole:

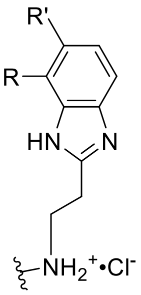

R=R'=H **22, 23, 31 - 34**
R=Me, R'=H **24**
R=NO_2, R'=H **25**
R=OMe, R'=H **26, 35**
R=H, R'=Me **27**
R=H, R'=NO_2 **28**
R=H, R'=OMe **29, 36**
R=H, R'=Cl **30**

COMPOUNDS AND CHARACTERISATION

2-Chloro-4-cyanophenyl trifluoromethanesulfonate (1a)

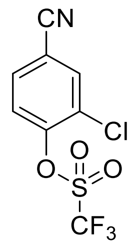

Prepared by general method **A** using 3-chloro-4-hydroxybenzonitrile (2.00 g, 13.0 mmol), anhydrous CH_2Cl_2 (40.0 mL), anhydrous pyridine (3.20 mL, 39.6 mmol) and trifluoromethanesulfonic anhydride (2.84 mL, 16.9 mmol). The crude residue was purified by flash column chromatography (silica gel, 5:95 EtOAc:Hexane) to provide the title compound **1a** as a white crystalline solid (3.37 g, 11.8 mmol, 91%):

R_f 0.68 (8:2 EtOAc:Hexane); **m.p.** = 61-62 °C; **IR ν_{max}**: 2245, 1575, 1478; **^{1}H NMR** (400 MHz, $CDCl_3$): δ 7.86 (1H, d, *J* = 2.0 Hz), 7.68 (1H, dd, *J* = 8.6, 2.0 Hz), 7.51 (1H, d, *J* = 8.6 Hz); **^{13}C NMR** (101 MHz, $CDCl_3$): δ 148.5, 134.9, 132.2, 128.9, 124.2, 120.1, 116.9, 114.9 (d, *J* = 227.3 Hz, 1C); **^{19}F NMR** (376 MHz, $CDCl_3$): δ -74.0; **LCMS** R_t 1.56 min, calcd for $[C_8H_3ClF_3NO_3S - H]^-$ 283.9 $[M-H]^-$ 284.0.

2,6-Dichloro-4-cyanophenyl trifluoromethanesulfonate (14a)

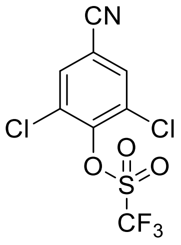

Prepared by general method **A** using 3,5-dichloro-4-hydroxybenzonitrile (500 mg, 2.66 mmol), anhydrous CH_2Cl_2 (2.40 mL), anhydrous pyridine (0.30 mL, 4.26 mmol) and trifluoromethanesulfonic anhydride (0.60 mL, 3.46 mmol). The crude residue was purified by flash column chromatography (silica gel, 10:90 EtOAc:Hexane) to provide the title compound **14a** as a white solid (630 mg, 1.97 mmol, 74%):

R_f 0.40 (1:9 EtOAc:Hexane); **m.p.** = 93-96 °C; **IR ν_{max}**: 2238, 1204, 860, 829; **^{1}H NMR** (400 MHz, DMSO-d_6): δ 8.48 (2H, s); **^{13}C NMR** (101 MHz, DMSO-d_6): δ 144.7[6], 134.4, 129.2, 119.5, 115.9, 114.2; **^{19}F NMR** (376 MHz, $CDCl_3$): δ -71.4; **LCMS** R_t = 4.07 min, calcd for $[C_8H_2Cl_2F_3NO_3S + K]^+$: 359.16, found: 358.21.

[6] Quartets were expected but doublets were observed. The two missing peaks of the quartets are probably lost in the baseline.

2,6-Dichloro-4-formylphenyl trifluoromethansulfonate (15a)

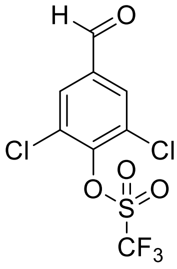

Prepared by general method **A** using 3,5-dichloro-4-hydroxybenzaldehyde (200 mg, 1.05 mmol), anhydrous CH_2Cl_2 (1.50 mL), anhydrous pyridine (0.15 mL, 1.88 mmol) and trifluoromethanesulfonic anhydride (0.26 mL, 1.52 mmol). The crude residue was purified by flash column chromatography (silica gel, 10:90 EtOAc:Hexane) to provide the title compound **15a** as a colourless oil (191 mg, 0.60 mmol, 57%):

R*f* 0.19 (1:9 EtOAc:Hexane); **IR** ν_{max}**:** 1706, 1429, 1210, 1129, 855, 744, 709; **^{1}H NMR** (500 MHz, $CDCl_3$): δ 9.97 (1H, s), 7.97 (2H, s); **^{13}C NMR** (126 MHz, DMSO-d_6): δ190.1, 141.1, 137.2, 131.0, 117.0 (q, *J* = 320 Hz, 1C); **^{19}F NMR** (376 MHz, $CDCl_3$): δ -71.4; **HRMS** (ESI) calcd for $[C_8H_4O_4{}^{35}Cl_2F_3S^{+}+H]^{+}$: 322.9159, found: 322.9153.

2-Chloro-[1,1'-biphenyl]-4-carbonitrile (1b)

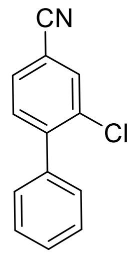

Prepared by general method **B** using **1a** (2.74 g, 9.58 mmol), phenylboronic acid (1.52 g, 12.4 mmol), DME (60.0 mL), 2M aqueous Na_2CO_3 (14.4 mL, 28.7 mmol) and $Pd(PPh_3)_4$ (554 mg, 0.479 mmol). The crude product was purified by flash column chromatography (silica gel, 5:95 EtOAc:Hexane) to provide the title compound **1b** as a white solid (1.09 g, 5.10 mmol, 73%):

R*f* 0.63 (2:8 EtOAc:Hexane); **m.p.** 78-79 °C; **IR** υ_{max} 3065, 2230, 1595, 1501, 1474; **^{1}H-NMR** (400 MHz, $CDCl_3$) δ 8.18 (d, *J* = 1.6 Hz, 1H), 7.91 (dd, *J* = 8.0, 1.6 Hz, 1H), 7.62 (d, *J* = 8.0 Hz, 1H), 7.54 - 7.44 (m, 5H); **^{13}C-NMR** (126 MHz, $CDCl_3$): 145.5, 137.8, 133.7, 133.5, 132.2, 130.5, 129.2, 128.8, 128.5, 117.6, 112.6; **HRMS** (ESI) calcd for $[C_{13}H_8N^{35}Cl + H]^{+}$: 214.0418, found 214.0412.

2-Chloro-2'-methyl-[1,1'-biphenyl]-4-carbonitrile (2b)

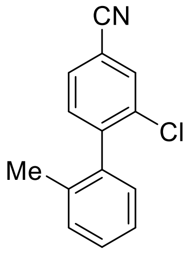

Prepared by general method **B** using 3-chloro-4-bromobenzonitrile (300 mg, 1.39 mmol), *o*-tolylboronic acid (189 mg, 1.39 mmol), $Pd(PPh_3)_4$ (80 mg, 0.07 mmol), K_2CO_3 (383 mg, 2.77 mmol), DME (3.0 mL) and H_2O (1.0 mL). The crude product was purified by flash column chromatography (silica gel, 1:9 EtOAc:pet ether) to provide the title compound **2b** as a clear oil (300 mg, 1.32 mmol, 95%):

R_f 0.56 (2:3 CH_2Cl_2:pet ether); **IR** υ_{max} 1473, 1384, 1073, 836, 761, 726, 609; **^{1}H NMR** (400 MHz, $CDCl_3$) δ 7.77 (d, *J* = 1.5 Hz, 1H), 7.63 (dd, *J* = 7.9, 1.6 Hz, 1H), 7.41 – 7.27 (m, 4H), 7.14 (d, *J* = 7.4 Hz, 1H), 2.16 (s, 3H); **^{13}C NMR** (101 MHz, $CDCl_3$) δ 145.6, 137.5, 135.5, 134.4, 132.6, 131.8, 130.2, 130.0, 128.7, 128.6, 125.7, 117.3, 112.5, 19.6; **HRMS** (ESI) calcd for $[C_{14}H_{10}{}^{35}ClN + H]^+$: 228.0580, found: 228.0575.

2-Chloro-2'-ethyl-[1,1'-biphenyl]-4-carbonitrile (3b)

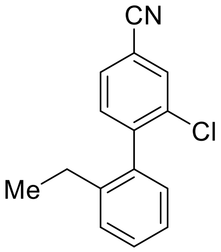

Prepared by general method **B** using 3-chloro-4-bromobenzonitrile (300 mg, 1.39 mmol), 2-ethylphenylboronic acid (208 mg, 1.39 mmol), $Pd(PPh_3)_4$ (80 mg, 0.07 mmol), K_2CO_3 (383 mg, 2.77 mmol), DME (3.0 mL) and H_2O (1.0 mL). The crude product was purified by flash column chromatography (silica gel, 1:9 EtOAc:pet ether) to provide the title compound **3b** as a clear oil (206 mg, 0.85 mmol, 61%):

R_f 0.40 (1:9 EtOAc:pet ether); **IR** υ_{max} 3067, 2967, 2933, 2873, 2232, 1596, 1539, 1472, 1445, 1382, 1255, 1196, 1140, 1074, 1005, 883, 863, 837, 760; **^{1}H NMR** (400 MHz, $CDCl_3$) δ 7.79 (d, *J* = 1.5 Hz, 1H), 7.62 (dd, *J* = 7.9, 1.6 Hz, 1H), 7.45 – 7.36 (m, 3H), 7.30 (td, *J* = 7.3, 1.7 Hz, 1H), 7.10 (dd, *J* = 7.6, 0.9 Hz, 1H), 2.56 – 2.33 (m, 2H), 1.10 (t, *J* = 7.6 Hz, 3H); **^{13}C NMR** (101 MHz, $CDCl_3$) δ 145.8, 141.7, 137.1, 134.9, 132.9, 132.2, 130.2, 129.1, 129.1, 128.6, 125.9, 117.6, 112.7, 26.2, 15.1; **HRMS** (ESI) calcd for $[C_{15}H_{12}N^{35}Cl + H]^+$: 242.0737, found: 242.0735.

2-Chloro-2'-isopropyl-[1,1'-biphenyl]-4-carbonitrile (4b)

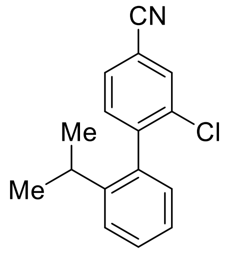

Prepared by general method **B** using **1a** (105 mg, 0.37 mmol), 2-isopropylphenylboronic acid (50 mg, 0.23 mmol), $Pd(PPh_3)_4$ (11 mg, 0.01 mmol), 2 M aqueous Na_2CO_3 solution (0.30 mL, 0.53 mmol) and DME (5.0 mL) and refluxed for 3 hours. The crude product was purified by flash column chromatography (silica gel, 20:80 EtOAc:Hexane) to provide the title compound **4b** as a colourless oil (40 mg, 0.15 mmol, 85%):

R_f 0.25 (2:8 EtOAc:Hexane); **IR ν_{max}**: 2962, 2326, 1473, 138, 832, 758; **^{1}H NMR** (400 MHz, CD_3OD): δ 7.96 (1H, dd, *J* = 1.6, 0.3 Hz), 7.76 (1H, dd, *J* = 7.9, 1.6 Hz), 7.47-7.41 (3H, m), 7.28-7.25 (1H, m), 7.05 (1H, ddd, *J* = 7.2, 1.8, 0.7 Hz), 2.60 (1H, sept, *J* = 6.8 Hz), 1.22 (3H, d, *J* = 6.7 Hz), 1.09 (3H, d, *J* = 6.7 Hz); **^{13}C NMR** (101 MHz, CD_3OD): δ 147.5, 147.3, 137.9, 135.9, 133.9, 133.7, 131.6, 130.2, 129.9, 126.8, 126.6, 118.4, 113.9, 31.6, 24.7, 23.4; **HRMS** (ESI) calcd for $[C_{15}H_{14}N^{35}Cl + H]^+$: 256.0893, found: 256.0893.

2-Chloro-2'-methoxy-[1,1'-biphenyl]-4-carbonitrile (5b)

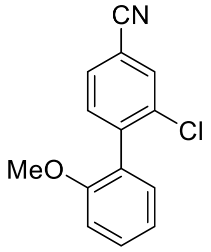

Prepared by general method **B** using 3-chloro-4-bromobenzonitrile (300 mg, 1.39 mmol), 2-methoxyphenylboronic acid (211 mg, 1.39 mmol), $Pd(PPh_3)_4$ (80 mg, 0.07 mmol), K_2CO_3 (383 mg, 2.77 mmol), DME (2.0 mL) and H_2O (0.6 mL). The crude product was purified by flash column chromatography (silica gel, 1:9 EtOAc:pet ether) to provide the title compound **5b** as a white solid (228 mg, 0.94 mmol, 67%):

R_f 0.55 (1:4 EtOAc:pet ether); **m.p.** 71.8–72.2 °C; **IR** υ_{max} 2938, 2837, 2359, 2232, 1604, 1503, 1475, 1435, 1384, 1275, 1255, 1234, 1195, 1181, 1163, 1122, 1075, 1050, 1025, 1004, 884, 865, 835, 793, 754; **^{1}H NMR** (400 MHz, $CDCl_3$) δ 7.75 (d, *J* = 1.6 Hz, 1H), 7.59 (dd, *J* = 7.9, 1.6 Hz, 1H), 7.45 – 7.39 (m, 2H), 7.16 (dd, *J* = 7.5, 1.8 Hz, 1H), 7.05 (td, *J* = 7.5, 1.0 Hz, 1H), 7.00 (d, *J* = 8.3 Hz, 1H), 3.79 (s, 3H); **^{13}C NMR** (101 MHz, $CDCl_3$) δ 156.5, 143.2, 135.2, 132.8, 132.7, 130.6, 130.5, 130.1, 126.9, 120.7, 117.9, 112.5, 111.2, 55.7; **HRMS** (ESI) calcd for $[C_{14}H_{10}NO^{35}Cl + H]^+$: 244.0529, found: 244.0525.

2-Chloro-2'-fluoro-[1,1'-biphenyl]-4-carbonitrile (6b)

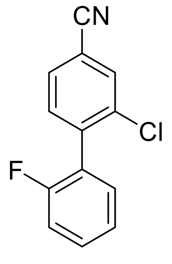

Prepared by general method **B** using 3-chloro-4-bromobenzonitrile (300 mg, 1.39 mmol), 2-fluorophenylboronic acid (194 mg, 1.39 mmol), $Pd(PPh_3)_4$ (80 mg, 0.07 mmol), K_2CO_3 (383 mg, 2.77 mmol), DME (3.0 mL) and H_2O (1.0 mL). The crude product was purified by flash column chromatography (silica gel, 1:9 EtOAc:pet ether) to provide the title compound **6b** as a white solid (58 mg, 0.25 mmol, 18%):

R_f 0.50 (1:9 EtOAc:pet ether); **m.p.** 46-48 °C; **IR** υ_{max} 1474, 1206, 1073, 768, 604; **^{1}H NMR** (400 MHz, $CDCl_3$) δ 7.73 (d, *J* = 1.5 Hz, 1H), 7.57 (dd, *J* = 7.9, 1.6 Hz, 1H), 7.44 – 7.35 (m, 2H), 7.28 – 7.17 (m, 2H), 7.17 – 7.09 (m, 1H); **^{13}C NMR** (101 MHz, $CDCl_3$) δ 159.3 (d, *J* = 248.8 Hz, 1C), 140.1, 134.9, 133.1, 132.6 (d, *J* = 1.1 Hz, 1C), 131.1, 131.0 (d, *J* = 12.7 Hz, 1C), 130.3, 125.4 (d, *J* = 15.5 Hz, 1C), 124.3 (d, *J* = 3.7 Hz, 1C), 117.5, 116.1 (d, *J* = 21.7 Hz, 1C), 113.4; **HRMS** (ESI) calcd for $[C_{13}H_7{}^{35}Cl^{19}FN + H]^+$: 232.0329, found: 232.0322.

2-Chloro-2'-hydroxy-[1,1'-biphenyl]-4-carbonitrile (7b)

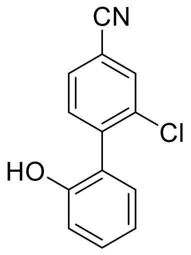

Prepared by general method **C** using 3-chloro-4-bromobenzonitrile (150 mg, 0.69 mmol), 2-hydroxyphenylboronic acid (87 mg, 0.63 mmol), $PdCl_2(dppf).CH_2Cl_2$ (26 mg, 0.032 mmol), K_3PO_4 (161 mg, 0.76 mmol), DME (2.0 mL), EtOH (0.5 mL) and H_2O (0.3 mL). The crude product was purified by flash column chromatography (silica gel, gradient elution: 5:95 Et_2O:Hexane to 20:80 Et_2O:Hexane) to provide the title compound **7b** as a white solid (100 mg, 0.44 mmol, 69%):

R_f 0.33 (1:1 Et_2O:Hexane); **m.p.** 141.6–141.9 °C; **IR** υ_{max} 3340, 2245, 1614, 1592, 1479, 1446, 1386, 1358, 1291, 1269, 1255, 1206, 1196, 1108, 1073, 1007, 878, 866, 846, 821, 749; **^{1}H NMR** (500 MHz, DMSO-d_6) δ 9.72 (s, 1H), 8.10 (d, *J* = 1.6 Hz, 1H), 7.83 (dd, *J* = 7.9, 1.7 Hz, 1H), 7.52 (d, *J* = 7.9 Hz, 1H), 7.26 (td, *J* = 8.2, 1.7 Hz, 1H), 7.10 (dd, *J* = 7.6, 1.6 Hz, 1H), 6.95 (dd, *J* = 8.2, 0.7 Hz, 1H), 6.89 (td, *J* = 7.5, 1.0 Hz, 1H); **^{13}C NMR** (126 MHz, DMSO-d_6) δ 154.3, 143.2, 133.9, 133.0, 132.6, 130.6, 130.2, 130.0, 124.7, 118.9, 117.7, 115.7, 111.5; **HRMS** (ESI) calcd for $[C_{13}H_8NO^{35}Cl + H]^+$: 230.0373, found: 230.0373.

2-Chloro-3'-methyl-[1,1'-biphenyl]-4-carbonitrile (8b)

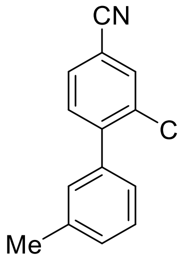

Prepared by general method **C** using 3-chloro-4-bromobenzonitrile (150 mg, 0.69 mmol), 3-methylphenylboronic acid (86 mg, 0.63 mmol), $PdCl_2$(dppf).CH_2Cl_2 (26 mg, 0.032 mmol), K_3PO_4 (161 mg, 0.76 mmol), DME (2.0 mL), EtOH (0.5 mL) and H_2O (0.3 mL). The crude product was purified by flash column chromatography (silica gel, elution: 5:95 Et_2O:Hexane) to provide the title compound **8b** as a white solid (120 mg, 0.53 mmol, 84%):

R_f 0.25 (1:19 Et_2O:Hexane); **m.p.** 72.3-72.4 °C; **IR** υ_{max} 3073, 2971, 2230, 1589, 1539, 1475, 1451, 1385, 1259, 1199, 1169, 1097, 1076, 1034, 968, 883, 836, 821, 777; **^{1}H NMR** (500 MHz, $CDCl_3$) δ 7.77 (d, *J* = 1.5 Hz, 1H), 7.60 (dd, *J* = 7.9, 1.6 Hz, 1H), 7.44 (d, *J* = 7.9 Hz, 1H), 7.36 (t, *J* = 7.9 Hz, 1H), 7.28 – 7.20 (m, 3H), 2.42 (s, 3H); **^{13}C NMR** (126 MHz, $CDCl_3$) δ 145.7, 138.3, 137.7, 133.7, 133.5, 132.2, 130.4, 129.8, 129.6, 128.4, 126.3, 117.7, 112.5, 21.6; **HRMS** (ESI) calcd for $[C_{14}H_{10}N^{35}Cl + H]^+$: 228.0580, found: 228.0577.

2-Chloro-3'-fluoro-[1,1'-biphenyl]-4-carbonitrile (9b)

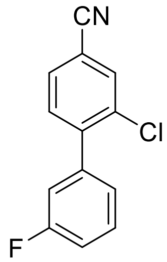

Prepared by general method **C** using 3-chloro-4-bromobenzonitrile (150 mg, 0.69 mmol), 3-fluorophenylboronic acid (88 mg, 0.63 mmol), $PdCl_2$(dppf).CH_2Cl_2 (26 mg, 0.032 mmol), K_3PO_4 (161 mg, 0.76 mmol), DME (2.0 mL), EtOH (0.5 mL) and H_2O (0.3 mL). The crude product was purified by flash column chromatography (silica gel, elution: 1:99 Et_2O:Hexane) to provide the title compound **9b** as a white solid (130 mg, 0.56 mmol, 89%):

R_f 0.37 (1:4 Et_2O:Hexane); **m.p.** 73.4-73.7 °C; **IR** υ_{max} 3072, 2971, 2232, 1616, 1583, 1541, 1500, 1470, 1429, 1380, 1293, 1279, 1254, 1182, 1158, 1083, 1068, 1025, 1005, 893, 873, 828, 778; **^{1}H NMR** (400 MHz, $CDCl_3$) δ 7.79 (d, *J* = 1.6 Hz, 2H), 7.62 (dd, *J* = 8.0, 1.6 Hz, 1H), 7.48 – 7.40 (m, 2H), 7.22 – 7.18 (m, 1H), 7.18 – 7.11 (m, 2H); **^{13}C NMR** (101 MHz, $CDCl_3$) δ 162.6 (d, *J* = 247.1 Hz, 1C), 144.2 (d, *J* = 2.1 Hz, 1C), 139.6 (d, *J* = 8.0 Hz, 1C), 133.7, 133.6, 132.1, 130.6, 130.2 (d, *J* = 8.4 Hz, 1C), 117.5, 116.5 (d, *J* = 22.6 Hz, 1C), 115.8 (d, *J* = 21.0 Hz, 1C), 113.1; **HRMS** (ESI) calcd for $[C_{13}H_7N^{35}Cl^{19}F + H]^+$: 232.0329, found: 232.0340.

2'-Chloro-[1,1'-biphenyl]-3,4'-dicarbonitrile (10b)

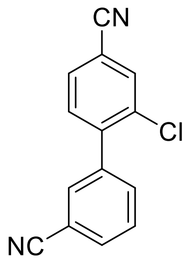

Prepared by general method **C** using 3-chloro-4-bromobenzonitrile (150 mg, 0.69 mmol), 3-cyanophenylboronic acid (93 mg, 0.63 mmol), $PdCl_2$(dppf).CH_2Cl_2 (26 mg, 0.032 mmol), K_3PO_4 (161 mg, 0.76 mmol), DME (2.0 mL), EtOH (0.5 mL) and H_2O (0.3 mL). The crude product was purified by flash column chromatography (silica gel, gradient elution: 20:80 Et_2O:Hexane to 30:70 Et_2O:Hexane) to provide the title compound **10b** as a white solid (113 mg, 0.47 mmol, 75%):

R*f* 0.19 (2:3 Et_2O:Hexane); **m.p.** 153.5–153.9 °C; **IR** υ_{max} 3064, 2971, 2232, 1579, 1539, 1493, 1474, 1420, 1384, 1258, 1200, 1174, 1073, 1030, 891, 883, 836, 802; **^{1}H NMR** (500 MHz, $CDCl_3$) δ 7.82 (d, *J* = 1.5 Hz, 1H), 7.76 – 7.72 (m, 2H), 7.69 – 7.66 (m, 2H), 7.61 (ddd, *J* = 8.2, 6.0, 0.6 Hz, 1H), 7.45 (d, *J* = 7.9 Hz, 1H); **^{13}C NMR** (126 MHz, $CDCl_3$) δ 143.0, 138.9, 133.7, 133.7, 133.7, 132.8, 132.4, 131.9, 130.9, 129.5, 118.3, 117.2, 113.8, 113.1; **HRMS** (ESI) calcd for $[C_{14}H_7N_2{}^{35}Cl + H]^+$: 239.0376, found: 239.0381.

2-Chloro-4'-fluoro-2'-methyl-[1,1'-biphenyl]-4-carbonitrile (11b)

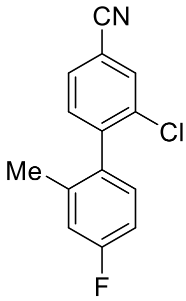

Prepared by general method **B** using **1a** (66 mg, 0.23 mmol), 4-fluoro-2-methylphenylboronic acid (50 mg, 0.32 mmol), $Pd(PPh_3)_4$ (13 mg, 0.01 mmol), 2 M aqueous Na_2CO_3 solution (0.30 mL) and DME (5.0 mL) and refluxed for 4 hours. The crude product was purified by flash column chromatography (silica gel, gradient elution: 0:100 EtOAc:Hexane to 5:95 EtOAc:Hexane) to provide the title compound **11b** as a colourless oil (55 mg, 0.22 mmol, 97%):

R*f* 0.37 (2:8 EtOAc:Hexane); **IR ν_{max}:** 2233, 1383, 831, 763; **^{1}H NMR** (500 MHz, $CDCl_3$): δ 7.78 (1H, d, *J* = 1.4 Hz), 7.60 (1H, dd, *J* =7.8, 1.5 Hz), 7.34 (1H, d, *J* = 7.8 Hz), 7.10 - 6.92 (3H, m), 2.09 (3H, s); **^{13}C NMR** (126 MHz, $CDCl_3$): δ 163.1 (d, *J* = 247.0 Hz, 1C), 144,9, 138.5 (d, *J* = 8.1 Hz, 1C), 134.9, 133.8, 132.9, 132,1, 130.5 (d, *J* = 8.6 Hz, 1C), 130.3, 117.4, 116.9 (d, *J* = 21.3 Hz, 1C), 112.9, 112.8 (d, *J* = 21.4, 1C), 19.8 (d, *J* = 1.5, 1C);[7] **^{19}F NMR** (375 MHz, $CDCl_3$); δ -114.3; **HRMS** (ESI) calcd for $[C_{14}H_{10}N^{35}ClF + H]^+$: 246.0493, found: 246.0486.

[7] Quartets were expected but doublets were observed. The two missing peaks of the quartets are probably lost in the baseline.

3-Chloro-4-(naphthalen-1-yl)benzonitrile (12b)

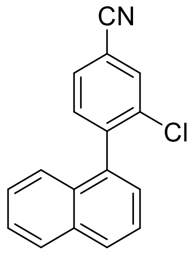

Prepared by general method **C** using 3-chloro-4-bromobenzonitrile (116 mg, 0.54 mmol), naphthalene-1-boronic acid (84 mg, 0.49 mmol), $PdCl_2$(dppf).CH_2Cl_2 (20 mg, 0.024 mmol), K_3PO_4 (124 mg, 0.59 mmol), DME (1.5 mL), EtOH (0.4 mL) and H_2O (0.2 mL). The crude product was purified by flash column chromatography (silica gel, 1:39 EtOAc:Hexane) to provide the title compound **12b** as a clear oil (105 mg, 0.40 mmol, 82%):

R_f 0.45 (1:4 EtOAc:Hexane); **IR** υ_{max} 3059, 2231, 1591, 1538, 1509, 1485, 1459, 1396, 1383, 1337, 1259, 1247, 1195, 1141, 1122, 1065, 1051, 1018, 965, 907, 884, 867, 837, 800, 775, 730; **^{1}H NMR** (400 MHz, $CDCl_3$) δ 8.01 – 7.93 (m, 2H), 7.87 (d, *J* = 1.6 Hz, 1H), 7.66 (dd, *J* = 7.9, 1.6 Hz, 1H), 7.61 – 7.52 (m, 2H), 7.51 – 7.45 (m, 2H), 7.45 – 7.41 (m, 1H), 7.38 (dd, *J* = 7.0, 1.1 Hz, 1H); **^{13}C NMR** (101 MHz, $CDCl_3$) δ 144.5, 135.5, 135.2, 133.4, 132.9, 132.9, 130.9, 130.2, 129.2, 128.5, 126.9, 126.7, 126.3, 125.2 (br s, 2C), 117.5, 113.0; **HRMS** (ESI) calcd for $[C_{17}H_{10}N^{35}Cl + H]^+$: 264.0580, found: 264.0576.

3-Chloro-4-(1H-indole-4-yl)benzonitrile (13b)

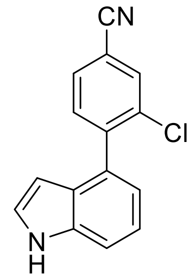

Prepared by general method **B** using **1a** (125 mg, 0.44 mmol), 4-(1H-indole)boronic acid pinacol ester (150 mg, 0.62 mmol), $Pd(PPh_3)_4$ (25 mg, 0.02 mmol), 2 M aqueous Na_2CO_3 solution (0.70 mL, 1.32 mmol) and DME (15.0 mL) and refluxed for 3 hours. The crude product was purified by flash column chromatography (silica gel, 20:80 EtOAc:Hexane) to provide the title compound **13b** as a brown oil (104 mg, 0.41 mmol , 67%):

R_f 0.44 (20:80 EtOAc:Hexane); **IR ν_{max}**: 3402, 2231, 1383, 1336, 754; **^{1}H NMR** (400 MHz, CD_3OD): δ 7.96 (1H, d, *J* = 1.6 Hz), 7.74 (1H, dd, *J* = 8.1, 1.6 Hz), 7.63 (1H, d, *J* = 8.0 Hz), 7.48 (1H, dd, *J* = 7.4, 0.9 Hz), 7.29 (1H, d, *J* = 3.2 Hz), 7.21 (1H, t, *J* = 7.3 Hz), 7.01 (1H, dd, *J* = 7.3, 0.8 Hz), 6.17 (1H, dd, *J* = 3.2, 0.8 Hz); **^{13}C NMR** (101 MHz, CD_3OD): δ 145.4, 136.3, 133.9, 132.9, 132.6, 130.0, 129.4, 126.5, 125.1, 120.5, 119.6, 117.2, 111.8, 111.4, 100.1; **HRMS** (ESI) calcd for $[C_{15}H_{10}N_2{}^{35}Cl +H]^+$: 253.0533, found: 253.0545.

2,6-Dichloro-[1,1'-biphenyl]-4-carbonitrile (14b)

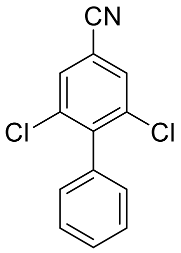

Prepared by general method **C** using **14a** (300 mg, 0.94 mmol), phenylboronic acid (160 mg, 1.31 mmol), $PdCl_2(dppf).CH_2Cl_2$ (48 mg, 0.06 mmol), K_3PO_4 (239 mg, 1.13 mmol), DME (0.8 mL), EtOH (0.6 mL) and H_2O (0.15 mL) and heated to 110°C under microwave irradiation for 2 hours. The crude product was purified by flash column chromatography (silica gel, gradient elution: 0:100 EtOAc:Hexane to 20:80 EtOAc:Hexane) followed by a second purification by preparative TLC plate (100% Hexane) to provide the title compound **14b** as a white film (55 mg, 0.22 mmol, 24%):

R_f 0.37 (100% hexane); **m.p.** = 100-103 °C; **IR ν_{max}**: 2236, 1532, 1444, 1207, 809, 767; **^{1}H NMR** (500 MHz, $CDCl_3$): δ 7.70 (2H, s), 7.58-7.48 (3H, m), 7.26-7.24 (2H, m); **^{13}C NMR** (126 MHz, $CDCl_3$): δ 144.6, 136.3, 135.3, 131.1, 128.9, 128.8, 128.4, 116.2, 113.2; **HRMS** (ESI) calcd for $[C_{13}H_8N^{35}Cl2+H]^+$: 248.0028, found: 248.0022.

2-Chloro-[1,1'-biphenyl]-4-carbaldehyde (16b)

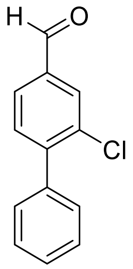

Prepared by general method **B** using 2-chloro-4-formylphenyl trifluoromethanesulfonate (341 mg, 1.18 mmol), phenylboronic acid (158 mg, 1.30 mmol), $Pd(PPh_3)_4$ (227 mg, 0.196 mmol), 2 M aqueous Na_2CO_3 solution (0.67 mL, 1.25 mmol) and DME (6.3 mL) and refluxed for 3 hours. The crude product was purified by flash column chromatography (silica gel, gradient elution: 2:98 EtOAc:hexane to 20:80 EtOAc:hexane) to provide the title compound **16b** as a white solid (139 mg, 0.64 mmol , 54%):

R_f 0.73 (1:4 EtOAc:hexane); **m.p.** = 96-98 °C; **IR** υ_{max} 2835, 1697, 1597; **^{1}H NMR** (400 MHz, $CDCl_3$) δ 10.02 (s, 1H), 7.99 (d, *J* = 1.6 Hz, 1H), 7.83 (dd, *J* = 7.8, 1.6 Hz, 1H), 7.53 (d, *J* = 7.8 Hz, 1H), 7.50-7.40 (m, 5H). **^{13}C NMR** (101 MHz, $CDCl_3$) δ 190.8, 146.5, 138.3, 136.6, 133.9, 132.2, 131.2, 129.3, 128.6, 128.4, 128.0; **HRMS** (ESI) calcd for $[C_{13}H_9O^{35}Cl + H]^+$: 217.0420, found: 217.0414.

2-Chloro-2'-ethyl-[1,1'-biphenyl]-4-carbaldehyde (23b)

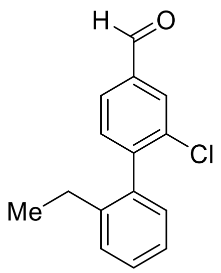

Prepared by general method **D** using 2-chloro-4-formylphenyl trifluoromethanesulfonate (500 mg, 1.73 mmol), 2-ethylphenylboronic acid (390 mg, 2.60 mmol), $PdCl_2$(dppf) (127 mg, 0.17 mmol), K_2CO_3 (717 mg, 5.19 mmol) and 1,4-dioxane (7.0 mL) and heating for 2.5 hours. The crude product was purified by flash column chromatography (silica gel, gradient elution: 0:100 EtOAc:pet ether to 10:90 EtOAc:pet ether) to provide the title compound **23b** as a pale yellow oil (310 mg, 1.27 mmol, 73%):

R_f 0.56 (1:5 EtOAc:pet ether); **IR** $υ_{max}$ 2967, 1698, 1595, 1370, 1186, 1067, 1005; **^{1}H NMR** (400 MHz, $CDCl_3$) δ 10.03 (s, 1H), 7.99 (d, *J* = 1.6 Hz, 1H), 7.82 (dd, *J* = 1.6, 7.7 Hz, 1H), 7.44 (d, *J* = 7.7 Hz, 1H), 7.30 – 7.42 (m, 2H), 7.28 (dd, *J* = 1.6, 7.4 Hz, 1H), 7.10 (dd, *J* = 1.1, 7.7 Hz, 1H), 2.32 – 2.54 (m, 2H), 1.06 (t, *J* = 7.5 Hz, 3H); **^{13}C NMR** (126 MHz, $CDCl_3$) δ 190.6, 146.8, 141.7, 137.7, 136.7, 134.9, 132.1, 130.4, 129.0, 128.4, 127.5, 125.7, 26.2, 15.0; **HRMS** (ESI) calcd for $[C_{15}H_{13}O^{35}Cl + H]^+$: 245.0733, found: 245.0741.

2'-ethyl-2-methyl-[1,1'-biphenyl]-4-carbaldehyde (31b)

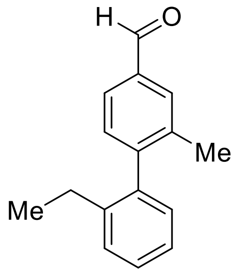

Prepared by general method **D** using 4-bromo-3-methylbenzaldehyde (199 mg, 1.00 mmol), 2-ethylphenylboronic acid (300 mg, 2.00 mmol), $PdCl_2$(dppf) (82 mg, 0.10 mmol), K_2CO_3 (553 mg, 4.00 mmol) and 1,4-dioxane (4.0 mL) and heating for 4 hours. The crude product was purified by flash column chromatography (silica gel, gradient elution: 0:100 EtOAc:pet ether to 5:95 EtOAc:pet ether) to provide the title compound **31b** as a pale yellow oil (202 mg, 0.90 mmol, 90%):

R_f 0.63 (1:10 EtOAc:pet ether); **IR** $υ_{max}$ 2966, 1692, 1606, 1565, 1478, 1445, 1385, 1284, 1221, 1152, 1006; **^{1}H NMR** (400 MHz, $CDCl_3$) δ 10.04 (s, 1H), 7.79 (s br, 1H), 7.74 (dd, *J* = 7.7, 1.3 Hz, 1H), 7.33 – 7.36 (m, 2H), 7.31 (d, *J* = 7.7 Hz, 1H), 7.23 – 7.27 (m, 1H), 7.05 (dd, *J* = 7.6, 0.8 Hz, 1H), 2.25 – 2.47 (m, 2H), 2.13 (3H, s), 1.03 (t, *J* = 7.5 Hz, 3H); **^{13}C NMR** (126 MHz, $CDCl_3$) δ 192.3, 148.1, 141.3, 139.6, 137.2, 135.4, 131.0, 130.4, 128.8, 128.5, 128.0, 127.1, 125.7, 26.1, 20.0, 15.1; **HRMS** (ESI) calcd for $[C_{16}H_{16}O + H]^+$: 225.1279, found: 225.1278.

2'-ethyl-2-(trifluoromethyl)-[1,1'-biphenyl]-4-carbaldehyde (32b)

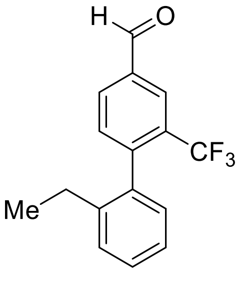

Prepared by general method **D** using 4-bromo-3-trifluoromethylbenzaldehyde (253 mg, 1.00 mmol), 2-ethylphenylboronic acid (300 mg, 2.00 mmol), $PdCl_2$(dppf) (82 mg, 0.10 mmol), K_2CO_3 (553 mg, 4.00 mmol) and 1,4-dioxane (4.0 mL) and heating for 6 hours. The crude product was purified by flash column chromatography (silica gel, gradient elution: 0:100 Et_2O:pet ether to 10:90 Et_2O:pet ether) to provide the title compound **32b** as a colourless oil (180 mg, 0.65 mmol, 65%):

R*f* 0.46 (1:5 Et_2O:pet ether); **IR** υ_{max} 2968, 1704, 1611, 1313, 1182, 1167, 1124, 1063, 1006; **^{1}H NMR** (400 MHz, $CDCl_3$) δ 10.12 (s, 1H), 8.28 (d, *J* = 1.2 Hz, 1H), 8.07 (dd, *J* = 7.8, 1.2 Hz, 1H), 7.47 (d, *J* = 7.8 Hz, 1H), 7.39 (ddd, *J* = 7.6, 7.3, 1.3 Hz, 1H), 7.33 (dd, *J* = 7.6, 1.3 Hz, 1H), 7.23 (ddd, *J* = 7.6, 7,3, 1.3 Hz, 1H), 7.10 (d, *J* = 7.6 Hz, 1H), 2.22 – 2.44 (m, 2H), 1.07 (t, *J* = 7.6 Hz, 3H); **^{13}C NMR** (126 MHz, $CDCl_3$) δ 192.0, 146.8, 141.4, 137.0, 135.3, 133.0, 131.6, 130.3 (q, *J* = 30.7 Hz, 1C), 128.9, 128.7, 128.0, 127.8 (q, *J* = 5.0 Hz, 1C), 124.9, 123.5 (q, *J* = 274.4 Hz, 1C), 26.1, 14.8; **HRMS** (ESI) calcd for $[C_{16}H_{13}OF_3 + H]^+$: 279.0991, found: 279.0988.

2'-ethyl-2-methoxy-[1,1'-biphenyl]-4-carbaldehyde (33b)

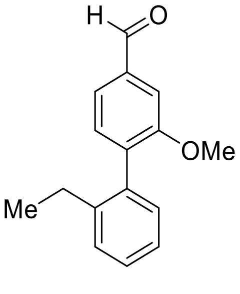

Prepared by general method **D** using 4-formyl-2-methoxyphenyl trifluoromethanesulfonate (222 mg, 0.78 mmol), 2-ethylphenylboronic acid (175 mg, 1.17 mmol), $PdCl_2$(dppf) (64 mg, 0.08 mmol), K_2CO_3 (324 mg, 2.34 mmol) and 1,4-dioxane (2.0 mL) and heating for 6 hours. The crude product was purified by flash column chromatography (silica gel, gradient elution: 0:100 EtOAc:pet ether to 20:80 EtOAc:pet ether) to provide the title compound **33b** as a pale yellow oil (172 mg, 0.72 mmol, 91%):

R*f* 0.45 (1:5 EtOAc:pet ether); **IR** υ_{max} 2967, 1687, 1597, 1573, 1461, 1412, 1385, 1263, 1240, 1152, 1034, 1004; **^{1}H NMR** (400 MHz, $CDCl_3$) δ 10.03 (s, 1H), 7.52 (dd, *J* = 7.5, 1.2 Hz, 1H), 7.48 (d, *J* = 1.3 Hz, 1H), 7.30 – 7.38 (m, 3H), 7.22 – 7.28 (m, 1H), 7.13 (dd, *J* = 7.5, 1.3 Hz, 1H), 3.83 (s, 3H), 2.33 – 2.54 (m, 2H), 1.06 (t, *J* = 7.6 Hz, 3H); **^{13}C NMR** (100 MHz, $CDCl_3$) δ 192.0, 157.4, 142.3, 137.8, 137.0, 136.8, 131.7, 129.6, 128.2, 128.1, 125.5, 124.2, 108.9, 55.7, 26.2, 15.0; **HRMS** (ESI) calcd for $[C_{16}H_{16}O_2 + H]^+$: 241.1237, found: 241.1228.

2'-ethyl-2-(trifluoromethoxy)-[1,1'-biphenyl]-4-carbaldehyde (34b)

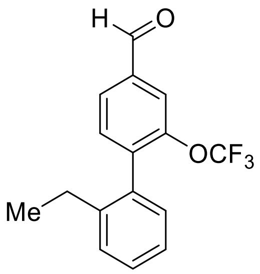

Prepared by general method **D** using 4-formyl-2-(trifluoromethoxy)phenyl trifluoromethanesulfonate (292 mg, 0.86 mmol), 2-ethylphenylboronic acid (259 mg, 1.73 mmol), $PdCl_2$(dppf) (70 mg, 0.09 mmol), K_2CO_3 (478 mg, 3.46 mmol) and 1,4-dioxane (4.0 mL) and heating for 5 hours. The crude product was purified by flash column chromatography (silica gel, gradient elution: 0:100 EtOAc:pet ether to 8:92 EtOAc:pet ether) to provide the title compound **34b** as a pale yellow oil (224 mg, 0.76 mmol, 88%):

R_f 0.47 (1:10 EtOAc:pet ether); **IR** υ_{max} 2971, 1702, 1250, 1205, 1007; **^{1}H NMR** (400 MHz, $CDCl_3$) δ 10.06 (s, 1H), 7.85 – 7.88 (m, 2H), 7.49 (d, *J* = 8.1 Hz, 1H), 7.33 – 7.41 (m, 2H), 7.23 – 7.29 (m, 1H), 7.12 (dd, *J* = 7.6, 1.1 Hz, 1H), 2.33 – 2.53 (m, 2H), 1.08 (t, *J* = 7.6 Hz, 3H); **^{13}C NMR** (100 MHz, $CDCl_3$) δ 190.4, 147.3, 141.9, 141.4, 137.0, 134.7, 132.9, 129.5, 128.8, 128.3, 127.8, 125.5, 120.8, 120.3 (q, *J* = 259.0 Hz, 1C), 26.0, 14.9; **HRMS** (ESI) calcd for $[C_{16}H_{13}O_2F_3 + H]^+$: 295.0946, found: 295.0950.

(2-Chloro-[1,1'-biphenyl]-4-yl)methanamine (1c)

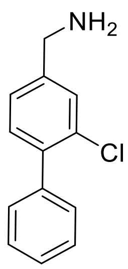

Prepared by general method **E** using $LiAlH_4$ (284 mg, 7.49 mmol), Et_2O (12.5 mL), $AlCl_3$ (998 mg, 7.49 mmol) and **1b** (800 mg, 3.74 mmol). The title amine **1c**, provided as a pale yellow oil (757 mg, 3.48 mmol, 93%), was of sufficient purity for the following step:

IR υ_{max} 3420, 2913, 1607, 1514; **^{1}H NMR** (400 MHz, $CDCl_3$) δ 7.46 – 7.34 (m, 6H), 7.30 (d, *J* = 7.8 Hz, 1H), 7.26 (dd, *J* = 7.8, 1.7 Hz, 1H, under the solvent peak), 3.89 (s, 2H); **^{13}C NMR** (101 MHz, $CDCl_3$) δ 144.1, 139.3, 139.0, 132.6, 131.6, 129.6, 128.6, 128.2, 127.7, 125.7, 45.8; **HRMS** (ESI) calcd for $[C_{13}H_{12}N^{35}Cl + H]^+$: 218.0737, found: 218.0729.

(2-Chloro-2'-methyl-[1,1'-biphenyl]-4-yl)methanamine HCl salt (2)

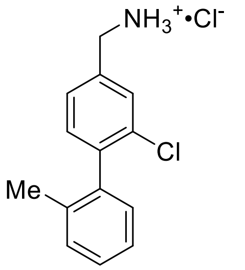

Prepared by general method **E** using $LiAlH_4$ (77 mg, 2.02 mmol), Et_2O (3.4 mL), $AlCl_3$ (269 mg, 2.02 mmol) and **2b** (230 mg, 1.01 mmol). The crude product was purified by flash column chromatography (silica gel, 1:5:14 NH_3:MeOH:CH_2Cl_2) to provide free amine **2**. The free amine was then dissolved in CH_2Cl_2 (0.5 mL) and HCl (2M in Et_2O) (5.05 mL, 10.1 mmol) added dropwise. The reaction was stirred for 1 hour before the precipitate was filtered, washed with cold Et_2O and dried to provide the title compound **2** as a yellow oil (53 mg, 0.20 mmol, 20%):

HPLC t_r = 9.24 mins (5-100% B); **IR** υ_{max} 2945, 2832, 1021; **^{1}H NMR** (500 MHz, DMSO-d_6) δ 8.61 (s, 3H), 7.77 (d, *J* = 1.6 Hz, 1H), 7.55 (dd, *J* = 7.8, 1.7 Hz, 1H), 7.37 – 7.30 (m, 3H), 7.29 – 7.24 (m, 1H), 7.08 (d, *J* = 7.3 Hz, 1H), 4.09 (s, 2H), 2.05 (s, 3H); **^{13}C NMR** (126 MHz, DMSO-d_6) δ 139.9, 138.3, 135.4, 135.4, 132.2, 131.3, 129.9, 129.7, 129.1, 128.1, 127.9, 125.8, 41.3, 19.4; **HRMS** (ESI) calcd for $[C_{14}H_{15}N^{35}Cl + H]^+$: 232.0893, found: 232.0846.

(2-Chloro-2'-ethyl-[1,1'-biphenyl]-4-yl)methanamine HCl salt (3)

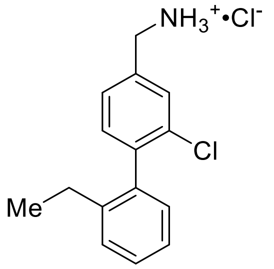

Prepared by general method **E** using $LiAlH_4$ (42 mg, 1.1 mmol), Et_2O (5.0 mL), $AlCl_3$ (147 mg, 1.1 mmol) and **3b** (132 mg, 0.55 mmol). The crude product was purified by flash column chromatography (silica gel, 1:5:14 NH_3:MeOH:CH_2Cl_2) to provide free amine **3**. The free amine was then dissolved in CH_2Cl_2 (0.5 mL) and HCl (2M in Et_2O) (2.75 mL, 5.5 mmol) added dropwise. The reaction was stirred for 1 hour before the precipitate was filtered, washed with cold Et_2O and dried to provide the title compound **3** as a white solid (65 mg, 0.23 mmol, 43%):

R_f 0.18 (1:10 MeOH:CH_2Cl_2); **m.p.** 181-183 °C; **IR** υ_{max} 3300 – 2300, 2963, 2871, 2612, 1601,1504, 1476, 1446, 1401, 1378, 1214, 1076, 1006; **^{1}H NMR** (500 MHz, DMSO-d_6) δ 8.63 (s, 3H), 7.78 (d, *J* = 1.6 Hz, 1H), 7.55 (dd, *J* = 7.8, 1.7 Hz, 1H), 7.39 – 7.33 (m, 3H), 7.28 – 7.23 (m, 1H), 7.04 (d, *J* = 7.3 Hz, 1H), 4.09 (s, 2H), 2.46 – 2.24 (m, 2H), 0.98 (t, *J* = 7.6 Hz, 3H); **^{13}C NMR** (126 MHz, DMSO-d_6) δ 141.4, 139.7, 137.7, 135.4, 132.3, 131.5, 129.7, 129.3, 128.3 (br s, 2C), 127.8, 125.7, 41.3, 25.7, 15.1; **HRMS** (ESI) calcd for $[C_{15}H_{16}N^{35}Cl + H]^+$: 246.1049, found: 458.0961.

(2-Chloro-2’-isopropyl-[1,1’-biphenyl]-4-yl)methanamine HCl salt (4)

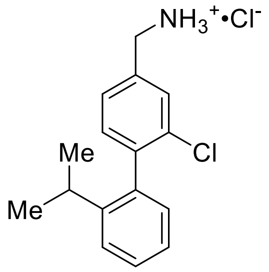

Prepared by general method **E** using $LiAlH_4$ (11 mg, 0.30 mmol), Et_2O (3.00 mL), $AlCl_3$ (20 mg, 0.15 mmol) and **4b** (40 mg, 0.15 mmol). The crude amine was dissolved in CH_2Cl_2 (0.5 mL) and HCl (2M in Et_2O) (0.75 mL, 1.5 mmol) added dropwise. The reaction was stirred for 1 hour before the precipitate was filtered, washed with cold Et_2O and dried to provide the title compound **4** as a white solid (35 mg, 0.12 mmol, 80%):

m.p. 218-220°C; **IR ν_{max}**: 2960, 1478, 1403, 834, 754; **^{1}H NMR** (500 MHz, CD_3OD): δ 7.68 (1H, d, *J* = 1.5 Hz), 7.49 (1H, dd, *J* = 7.8, 1.5 Hz), 7.44 - 7.38 (2H, m), 7.35 (1H, d, *J* = 7.8 Hz), 7.24 (1H, td, *J* = 6.9, 1.7 Hz), 7.03 (1H, dd, *J* = 7.0, 1.6 Hz), 4.30 (2H, s), 2.65 (1H, sept, *J* = 6.7 Hz), 1.19 (3H, d, *J* = 7.4 Hz), 1.06 (3H, d, *J* = 7.4 Hz); **^{13}C NMR** (126 MHz, CD_3OD): δ 146.3, 141.5, 137.4, 134.1, 133.9, 131.9, 129.6, 128.9, 128.4, 127.1, 125.2, 125.0, 42.1, 30.1, 22.0, 23.4; **HRMS** (ESI) calcd for $[C_{16}H_{19}N_2{}^{35}Cl + H]^+$: 260.1206, found: 260.1196.

(2-Chloro-2'-methoxy-[1,1'-biphenyl]-4-yl)methanamine HCl salt (5)

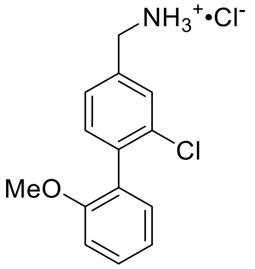

Prepared by general method **E** using $LiAlH_4$ (68 mg, 1.8 mmol), Et_2O (3.0 mL), $AlCl_3$ (240 mg, 1.8 mmol) and **5b** (220 mg, 0.9 mmol). The crude product was purified by flash column chromatography (silica gel, 1:5:14 NH_3:MeOH:CH_2Cl_2) to provide free amine **5**. The free amine was then dissolved in CH_2Cl_2 (0.5 mL) and HCl (2M in Et_2O) (4.5 mL, 9.0 mmol) added dropwise. The reaction was stirred for 1 hour before the precipitate was filtered, washed with cold Et_2O and dried to provide the title compound **5** as a white solid (65 mg, 0.23 mmol, 26%):

R_f 0.15 (1:10 MeOH:CH_2Cl_2); **m.p.** 194-196 °C; **IR** υ_{max} 3300 – 2300, 2890, 2591, 1599, 1582, 1505, 1479, 1463, 1434, 1397, 1378, 1275, 1254, 1232, 1214, 1121, 1075, 1049, 1026, 1003; **^{1}H NMR** (500 MHz, DMSO-d_6) δ 8.37 (s, 3H), 7.68 (d, *J* = 1.6 Hz, 1H), 7.47 (dd, *J* = 7.8, 1.7 Hz, 1H), 7.41 (ddd, *J* = 8.3, 7.4, 1.8 Hz, 1H), 7.35 (d, *J* = 7.8 Hz, 1H), 7.12 (dd, *J* = 7.5, 1.8 Hz, 2H), 7.03 (td, *J* = 7.4, 1.0 Hz, 1H), 4.08 (s, 2H), 3.72 (s, 3H); **^{13}C NMR** (126 MHz, DMSO-d_6) δ 156.3, 137.5, 135.1, 132.9, 131.9, 130.4, 129.8, 129.5, 127.6, 127.2, 120.3, 111.4, 55.4, 41.5; **HRMS** (ESI) calcd for $[C_{14}H_{14}NO^{35}Cl + H]^+$: 248.0842, found: 248.0834.

(2-Chloro-2'-fluoro-[1,1'-biphenyl]-4-yl)methanamine HCl salt (6)

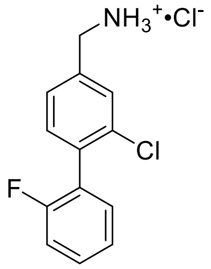

Prepared by general method **E** using $LiAlH_4$ (17 mg, 0.44 mmol), Et_2O (3.0 mL), $AlCl_3$ (59 mg, 0.44 mmol) and **6b** (50 mg, 0.22 mmol). The crude product was purified by flash column chromatography (silica gel, 1:5:14 NH_3:MeOH:CH_2Cl_2) to provide free amine **6**. The free amine was then dissolved in CH_2Cl_2 (0.5 mL) and HCl (2M in Et_2O) (1.1 mL, 2.2 mmol) added dropwise. The reaction was stirred for 1 hour before the precipitate was filtered, washed with cold Et_2O and dried to provide the title compound **6** as a white solid (55 mg, 0.20 mmol, 93%):

HPLC t_r = 8.73 mins (5-100% B); **m.p.** 198.3-201.1 °C; **IR** υ_{max} 2977, 2916, 1641, 1044; **^{1}H NMR** (500 MHz, DMSO-d_6) δ 8.34 (s, 3H), 7.76 (d, *J* = 1.6 Hz, 1H), 7.57 – 7.43 (m, 3H), 7.40 – 7.26 (m, 3H), 4.11 (s, 2H); **^{13}C NMR** (126 MHz, DMSO-d_6) δ 158.9 (d, *J* = 245.4 Hz, 1C), 136.2, 134.3, 132.5, 131.9, 131.4 (d, *J* = 1.8 Hz, 1C), 130.7 (d, *J* = 8.1 Hz, 1C), 129.8, 127.9, 125.8 (d, *J* = 15.8 Hz, 1C), 124.6 (d, *J* = 2.9 Hz, 1C), 115.6 (d, *J* = 21.8 Hz, 1C), 41.4; **HRMS** (ESI) calcd for $[C_{13}H_{12}N^{35}ClF + H]^+$: 236.0642, found: 236.0564.

4'-(aminomethyl)-2'-chloro-[1,1'-biphenyl]-2-ol HCl salt (7)

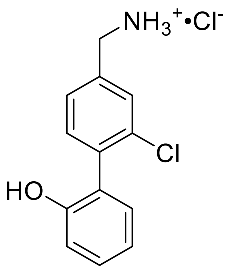

Prepared by general method **E** using $LiAlH_4$ (33 mg, 0.88 mmol), Et_2O (1.1 mL), $AlCl_3$ (117 mg, 0.88 mmol) and **7b** (50 mg, 0.22 mmol). The crude amine was dissolved in CH_2Cl_2 (0.5 mL) and HCl (2M in Et_2O) (1.1 mL, 2.2 mmol) added dropwise. The reaction was stirred for 1 hour before the precipitate was filtered, washed with cold Et_2O and dried to provide the title compound **7** as a brown solid (35 mg, 0.13 mmol, 59%):

HPLC t_r = 7.63 mins (5-95% B); **m.p.** 314.1-314.5 °C; **IR** υ_{max} 3204, 2919, 2590, 2354, 1980, 1606, 1485, 1447, 1401, 1374, 1290, 1259, 1205, 1108, 1074, 1006, 862, 819, 750; **^{1}H NMR** (400 MHz, DMSO-d_6) δ 9.61 (s, 1H), 8.52 (s, 3H), 7.68 (d, *J* = 1.5 Hz, 1H), 7.47 (dd, *J* = 7.9, 1.6 Hz, 1H), 7.34 (d, *J* = 7.8 Hz, 1H), 7.21 (ddd, *J* = 8.1, 7.5, 1.7 Hz, 1H), 7.05 (dd, *J* = 7.5, 1.7 Hz, 1H), 6.95 (dd, *J* = 8.1, 0.8 Hz, 1H), 6.85 (td, *J* = 7.4, 1.0 Hz, 1H), 4.06 (s, 2H); **^{13}C NMR** (101 MHz, DMSO-d_6) δ 154.6, 137.9, 134.9, 132.9, 132.1,

130.6, 129.6, 129.3, 127.4, 125.6, 118.7, 115.6, 41.4; **HRMS** (ESI) calcd for $[C_{13}H_{12}NO^{35}Cl + H]^+$: 234.0680, found: 234.0671.

(2-Chloro-3'-methyl-[1,1'-biphenyl]-4-yl)methanamine TFA salt (8)

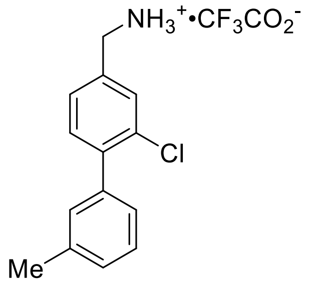

Prepared by general method **E** using $LiAlH_4$ (33 mg, 0.88 mmol), Et_2O (2.0 mL), $AlCl_3$ (116 mg, 0.88 mmol) and **8b** (100 mg, 0.44 mmol). The crude amine was purified by semi-preparative HPLC (20-70% B) to provide title compound **8** as a white solid (70 mg, 0.20 mmol, 46%):

HPLC t_r = 7.73 mins (20-70% B); **m.p.** 143.4-143.6 °C; **IR** $υ_{max}$ 2988, 1673, 1596, 1530, 1482, 1459, 1427, 1401, 1378, 1328, 1207, 1182, 1129, 1078, 971, 914, 881, 837, 798, 790; **^{1}H NMR** (500 MHz, DMSO-d_6) δ 8.33 (s, 3H), 7.70 (d, *J* = 1.6 Hz, 1H), 7.49 (dd, *J* = 7.9, 1.7 Hz, 1H), 7.45 (d, *J* = 7.9 Hz, 1H), 7.36 (t, *J* = 7.5 Hz, 1H), 7.26 – 7.17 (m, 3H), 4.11 (s, 2H), 2.37 (s, 3H); **^{13}C NMR** (126 MHz, DMSO-d_6) δ 140.0, 138.1, 137.5, 135.2, 131.6, 131.3, 130.2, 129.6, 128.6, 128.2, 128.0, 126.3, 41.4, 21.0; **HRMS** (ESI) calcd for $[C_{14}H_{14}N^{35}Cl + H]^+$: 232.0893, found: 232.0897.

(2-Chloro-3'-fluoro-[1,1'-biphenyl]-4-yl)methanamine TFA salt (9)

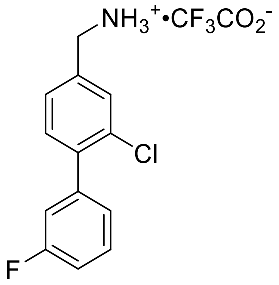

Prepared by general method **E** using $LiAlH_4$ (33 mg, 0.87 mmol), Et_2O (2.0 mL), $AlCl_3$ (116 mg, 0.87 mmol) and **9b** (100 mg, 0.43 mmol). The crude amine was purified by semi-preparative HPLC (5-55% B) to provide title compound **9** as a white solid (116 mg, 0.33 mmol, 77%):

HPLC t_r = 11.61 mins (5-55% B); **m.p.** 152.4-152.7 °C; **IR** $υ_{max}$ 2988, 1675, 1589, 1527, 1474, 1432, 1405, 1378, 1296, 1257, 1207, 1184, 1134, 1073, 977, 913, 879, 832, 789; **^{1}H NMR** (500 MHz, DMSO-d_6) δ 8.26 (s, 3H), 7.73 (s, 1H), 7.61 – 7.43 (m, 3H), 7.36 – 7.15 (m, 3H), 4.12 (s, 2H); **^{13}C NMR** (126 MHz, DMSO-d_6) δ 161.8 (d, *J* = 244.2 Hz, 1C), 140.3 (d, *J* = 8.0 Hz, 1C), 138.5, 135.9, 131.7, 131.2, 130.4 (d, *J* = 8.6 Hz, 1C), 130.3, 128.1, 125.5, 116.1 (d, *J* = 22.0 Hz, 1C), 114.9 (d, *J* = 20.8 Hz, 1C), 41.4; **HRMS** (ESI) calcd for $[C_{13}H_{11}N^{35}Cl^{19}F + H]^+$: 236.0642, found: 236.0645.

(2'-Chloro-[1,1'-biphenyl]-3,4'-diyl)dimethanamine TFA salt (10)

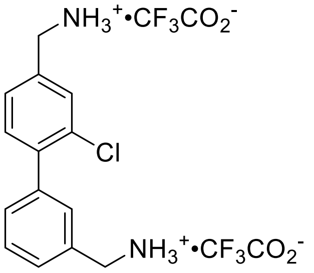

Prepared by general method **E** using $LiAlH_4$ (55 mg, 1.44 mmol), Et_2O (4.0 mL), $AlCl_3$ (59 mg, 1.44 mmol) and **10b** (85 mg, 0.36 mmol). The crude amine was purified by semi-preparative HPLC (5-35% B) to provide title compound **10** as a yellow oil (78 mg, 0.16 mmol, 46%):

HPLC t_r = 6.65 mins (5-35% B); **IR** υ_{max} 2988, 2344, 1667, 1526, 1435, 1380, 1191, 1131, 1080, 906, 839, 797; **^{1}H NMR** (500 MHz, DMSO-d$_6$) δ 8.51 – 8.14 (m, 6H), 7.73 (d, *J* = 1.5 Hz, 1H), 7.56 – 7.50 (m, 4H), 7.48 – 7.44 (m, 2H), 4.20 – 3.99 (m, *J* = 5.5 Hz, 4H); **^{13}C NMR** (126 MHz, DMSO-d$_6$) δ 139.2, 138.4, 135.6, 134.3, 131.6, 131.2, 130.3, 129.7, 129.3, 128.6, 128.4, 128.1, 42.1, 41.3; **HRMS** (ESI) calcd for $[C_{14}H_{15}N_2{}^{35}Cl + H]^+$: 247.1002, found: 247.1000.

(2-Chloro-4'-fluoro-2'methyl-[1,1'-biphenyl]-4-yl)methanamine HCl salt (11)

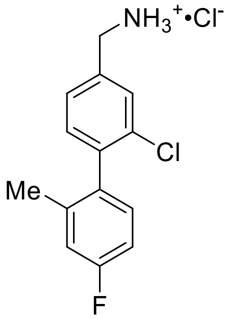

Prepared by general method **E** using $LiAlH_4$ (32 mg, 0.84 mmol), Et_2O (7.20 mL), $AlCl_3$ (56 mg, 0.48 mmol) and **11b** (70 mg, 0.28 mmol). The crude product was purified by flash column chromatography (silica gel, 1:4:95 NH_3:MeOH:CH_2Cl_2) to provide free amine **11** (28 mg, 0.11 mmol, 39%) as colourless oil. The free amine (23 mg, 0.09 mmol) was then dissolved in CH_2Cl_2 (0.1 mL) and HCl (2M in Et_2O) (0.45 mL, 0.9 mmol) added dropwise. The reaction was stirred for 1 hour before the precipitate was filtered, washed with cold Et_2O and dried to provide the title compound **11** as a white solid (8 mg, 0.30 mmol, 32%):

R_f (amine) 0.70 (2:8:90 NH_3:MeOH:CH_2Cl_2); **m.p.** 189-193 °C; **IR ν_{max}**: 2981, 1481, 860, 813; **^{1}H NMR** (500 MHz, CD_3OD): δ 7.52 (1H, d, *J* = 1.5 Hz), 7.35 (1H, dd, *J* = 7.8, 1.5 Hz), 7.19 (1H, d, *J* = 7.8 Hz), 7.08-7.04 (1H, m), 7.03-7.01 (1H, m), 6.96 (1H, td, *J* = 8.5, 2.6 Hz), 4.20 (2H, s), 2.83 (3H, s); **^{13}C NMR** (126 MHz, CD_3OD): δ 163.2 (d, *J* = 244.5 Hz), 142,7, 138.7 (d, *J* = 8.0 Hz, C1), 138.3, 135.2, 133.3, 131.1, 130.7 (d, *J* = 8.4 Hz, 1C), 128.2, 125.9, 115.8 (d, *J* = 21.5 Hz, 1C), 111.8 (d, *J* = 21.5 Hz, 1C), 44.2, 18.6 (d, *J* = 1.6 Hz, 1C); **^{19}F NMR** (376 MHz, d$_6$-DMSO): δ -114.7 ; **HRMS** (ESI) cald for $[C_{14}H_{14}{}^{35}ClFN + H]^+$:

250.0793, found: 250.0797.

(3-Chloro-4-(naphthalen-1-yl)phenyl)methanamine HCl salt (12)

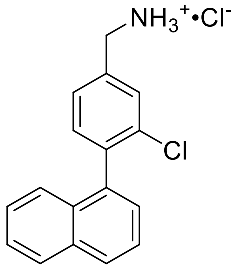

Prepared by general method **E** using $LiAlH_4$ (9 mg, 0.24 mmol), Et_2O (0.5 mL), $AlCl_3$ (32 mg, 0.24 mmol) and **12b** (31 mg, 0.12 mmol). The crude amine was dissolved in CH_2Cl_2 (0.5 mL) and HCl (2M in Et_2O) (0.6 mL, 1.2 mmol) added dropwise. The reaction was stirred for 1 hour before the precipitate was filtered, washed with cold Et_2O and dried to provide the title compound **12** as a yellow solid (14 mg, 0.05 mmol, 38%):

HPLC t_r = 9.40 mins (5-95% B); **m.p.** 246.3–246.7 °C; **IR** $υ_{max}$ 3377, 2901, 1592, 1493, 1395, 1377, 1248, 1215, 1185, 1123, 1065, 1051, 1017, 964, 887, 833, 802, 774, 688, 650; **^{1}H NMR** (400 MHz, DMSO-d$_6$) δ 8.56 (s, 3H), 8.06 – 7.99 (m, 2H), 7.84 (d, *J* = 1.5 Hz, 1H), 7.65 – 7.59 (m, 2H), 7.58 – 7.53 (m, 1H), 7.52 – 7.44 (m, 2H), 7.38 (dd, *J* = 7.0, 1.0 Hz, 1H), 7.30 (d, *J* = 8.3 Hz, 1H), 4.16 (d, *J* = 3.9 Hz, 2H); **^{13}C NMR** (101 MHz, DMSO-d$_6$) δ 138.6, 136.2, 135.9, 133.1, 132.9, 132.3, 130.9, 129.9, 128.4 (br s, 2C), 128.0, 127.1, 126.6, 126.1, 125.5, 124.9, 41.4; **HRMS** (ESI) calcd for [$C_{17}H_{14}N^{35}Cl$]: 267.0815, found: 267.0811.

(3-Chloro-4-(1H-indol-4-yl)phenyl)methanamine (13)

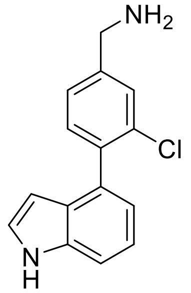

Prepared by general method **E** using $LiAlH_4$ (29 mg, 0.75 mmol), Et_2O (7.00 mL), $AlCl_3$ (50 mg, 0.37 mmol) and **13b** (95 mg, 0.38 mmol). The crude product was purified by flash column chromatography (silica gel, gradient elution: 0:2:98 to 1:4:95 NH_3 (7M solution in MeOH):MeOH:CH_2Cl_2 to provide the title compound **13** as a white solid (55 mg, 0.21 mmol, 55%):

R$_f$ 0.20 (0.2:1.8:98 NH_3:MeOH:CH_2Cl_2); **m.p.** 150-152°C; **IR ν$_{max}$**: 2983, 1373, 938, 846; **^{1}H NMR** (400 MHz, CD_3OD): δ 7.55 (1H, d, *J* = 1.4 Hz), 7.43-7.40 (2H, m), 7.34 (1H, dd, *J* = 7.8, 1.6 Hz), 7.23 (1H, d, *J* = 3.2 Hz), 7.17 (1H, t, *J* = 7.4 Hz), 6.96 (1H, dd, *J* = 7.2, 0.7 Hz), 6.12 (1H, dd, *J* = 3.2, 0.7 Hz), 3.88 (2H, s); **^{13}C NMR** (101 MHz, CD_3OD): δ 142.2, 138.8, 136.3, 132.9, 131.7, 131.1, 128.4, 127.0, 125.4, 124.4,

120.5, 119.8, 110.4, 100.5, 44.4; **HRMS** (ESI) calcd for $[C_{15}H_{14}N^{35}Cl_2 + H]^+$: 257.0840, found: 257.0834.

(2,6-Dichloro-[1,1'-biphenyl]-4-yl)methanamine HCl salt (14)

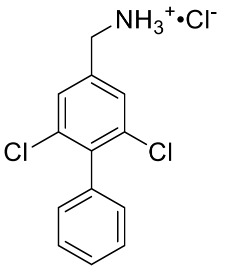

Prepared by general method **E** using $LiAlH_4$ (7 mg, 0.18 mmol), Et_2O (1.70 mL), $AlCl_3$ (12 mg, 0.09 mmol) and **14b** (22 mg, 0.09 mmol). The crude product was purified by flash column chromatography (silica gel, 1:9:90 NH_3:MeOH:CH_2Cl_2) to provide free amine **14** (20 mg, 0.08 mmol, 88%). The free amine (20 mg, 0.08 mmol) was then dissolved in CH_2Cl_2 (1.0 mL) and HCl (2M in Et_2O) (0.40 mL, 0.80 mmol) added dropwise. The reaction was stirred for 1 hour before the precipitate was filtered, washed with cold Et_2O and dried to provide the title compound **14** as a white solid (10 mg, 0.04 mmol, 50%):

R_f 0.44 (amine) (1:9:90 NH_3:MeOH:CH_2Cl_2); **m.p.** = 246-250 °C; **IR ν_{max}**: 3332, 892, 879; **^{1}H NMR** (500 MHz, CD_3OD): δ 7.62 (2H, s), 7.50-7.41 (3H, m), 7.22-7.18 (2H, m), 4.19 (2H, s); **^{13}C NMR** (126 MHz, CD_3OD): δ 141.7, 137.7, 136.6, 136.4, 131.7, 130.4, 129.5, 129.5, 43.0; **HRMS** (ESI) calcd for $[C_{13}H_{12}N^{35}Cl_2 + H]^+$: 252.0341, found: 252.0337.

(2,6-dichloro-2'-ethyl-[1,1'-biphenyl]-4-yl)methanamine TFA salt (15)

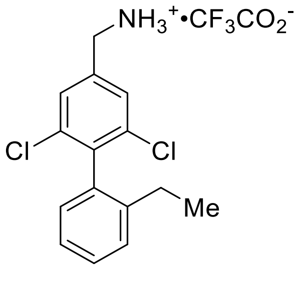

Prepared by general method **C** using **15a** (771 mg, 2.40 mmol), 2-ethyl-phenylboronic acid (430 mg, 2.87 mmol), $PdCl_2$(dppf).CH_2Cl_2 (98 mg, 0.12 mmol), K_3PO_4 (1.02 g, 4.80 mmol), DME (5.60 mL), EtOH (0.35 mL) and H_2O (0.06 mL) and refluxed for 3 hours. A solution of crude 2,6-dichloro-2'-ethyl-[1,1'-biphenyl]-4-carbaldehyde (200 mg), *t*-butylcarbamate (170 mg, 4.30 mmol), EtSiH (0.66 mL, 4.30 mmol) and TFA (0.21 mL, 2.72 mmol) in MeCN (3.20 mL) was stirred at room temperature for 14 h. The mixture was diluted with Et_2O and washed with an aqueous solution of $NaHCO_3$ and a saturated aqueous solution of NaCl. The combined organic layers were dried over $MgSO_4$, filtered and concentrated *in vacuo*. To the *N*-Boc derivative was added TFA (4.5 mL) and the mixture stirred at room temperature with monitoring by TLC. After 15 minutes the excess TFA was blown off under nitrogen and the residue stirred in Et_2O to give **15** as a white solid (24 mg, 0.09 mmol):

R_f 0.05 (amine) (1:9 MeOH:CH_2Cl_2); **m.p**. 218-222 °C; **IR ν_{max}:** 3373, 1674, 1118, 970; **^{1}H NMR** (500 MHz, CD_3OD): δ 7.66 (2H, s), 7.41-7.39 (2H, m), 7.39-7.27 (1H, m), 6.99 (1H, d, *J* = 7.4 Hz), 4.19 (2H, s), 2.36 (2H, q, *J* = 7.6 Hz), 1.08 (2H, t, *J* = 7.6 Hz); **^{13}C NMR** (126 MHz, CD_3OD): δ 141.6, 135.6, 135.4, 135.1, 128,9, 128.7, 128.3, 128.2, 125.8, 125.7, 41.7, 25.9, 13.9; **^{19}F NMR** (376 MHz, CD_3OD): δ -76.4; **HRMS** (ESI) m/z calcd for $[C_{15}H_{16}Cl_2N + H]^+$: 280.0654, found 280.0641.

***N*-benzyl-1-(2-chloro-[1,1'-biphenyl]-4-yl)methanamine HCl salt (16)**

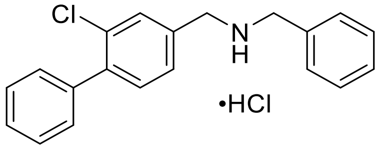

Prepared by general method **F** using aldehyde **16b** (76 mg, 0.35 mmol), benzylamine (57 µL, 0.53 mmol), 1,2-dichloroethane (1.25 mL) and sodium triacetoxyborohydride (104 mg, 0.49 mmol). The crude product was purified by flash column chromatography (silica gel, gradient elution: 2:98 MeOH:CH_2Cl_2 to 10:90 MeOH:CH_2Cl_2) to provide free amine **16** (88 mg, 0.29 mmol, 82%). The free amine (88 mg, 0.29 mmol) was then dissolved in CH_2Cl_2 (0.5 mL) and HCl (2M in Et_2O) (1.45 mL, 2.90 mmol) added dropwise. The reaction was stirred for 1 hour before the precipitate was filtered, washed with cold Et_2O and dried to provide the title compound **16** as a white solid (92 mg, 0.28 mmol, 94%):

R_f 0.59 (amine) (1:9 MeOH:CH_2Cl_2); **m.p.** 222-223 °C; **IR** υ_{max} 2933, 2767, 1440, 830, 762; **^{1}H NMR** (500 MHz, DMSO-d_6) δ 9.70 (br s, 2H), 7.82 (d, *J* = 1.6 Hz, 1H), 7.62 – 7.52 (m, 3H), 7.53 – 7.25 (m, 9H), 4.23 (s, 2H), 4.20 (s, 2H); **^{13}C NMR** (126 MHz, DMSO-d_6) δ 140.2, 138.1, 133.2, 131.9, 131.6, 131.4, 131.2, 130.1, 129.3, 129.1, 129.0, 128.6, 128.3, 128.0, 50.1, 49.0; **HRMS** (ESI) calcd for $[C_{20}H_{18}N^{35}Cl + H]^+$: 308.1206, found: 308.1204.

***N*-((2-chloro-[1,1'-biphenyl]-4-yl)methyl)-2-phenylethan-1-amine HCl salt (17)**

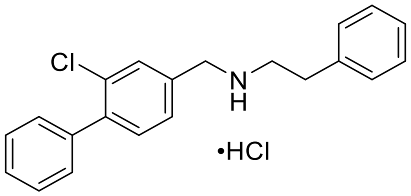

Prepared by general method **F** using aldehyde **16b** (43 mg, 0.20 mmol), phenethylamine (38 µL, 0.30 mmol), 1,2-dichloroethane (0.71 mL) and sodium triacetoxyborohydride (59 mg, 0.29 mmol). The crude product was purified by flash column chromatography (silica gel, gradient elution: 2:98 MeOH:CH_2Cl_2 to 10:90 MeOH:CH_2Cl_2) to provide free amine **17** (53 mg, 0.18 mmol, 82%). The free amine (50 mg, 0.16 mmol) was then dissolved in CH_2Cl_2 (0.5 mL) and HCl (2M in Et_2O) (0.80 mL, 1.60 mmol) added dropwise. The reaction was stirred for 1 hour before the precipitate was filtered, washed with cold Et_2O and dried to provide the title compound **17** as a white solid (52 mg, 0.18 mmol, 92%):

R_f 0.62 (amine) (1:9 MeOH:CH_2Cl_2); **m.p.** 248-250 °C; **IR** υ_{max} 2790, 1446, 767, 699; **^{1}H NMR** (400 MHz, DMSO-d_6) δ 9.64 (br s, 2H), 7.85 (d, *J* = 1.6 Hz, 1H), 7.63 (dd, *J* = 7.9, 1.7 Hz, 1H), 7.52 – 7.40 (m, 6H), 7.37 – 7.31 (m, 2H), 7.29 – 7.22 (m, 3H), 4.23 (s, 2H), 3.20 – 3.13 (m, 2H), 3.08 – 2.99 (m, 2H); **^{13}C NMR** (101 MHz, DMSO-d_6) δ 140.2, 138.1, 137.3, 133.3, 131.6, 131.4, 131.3, 129.3, 129.2, 128.7, 128.6, 128.3, 128.0, 126.8, 48.9, 47.7, 31.4; **HRMS** (ESI) calcd for $[C_{21}H_{20}N^{35}Cl + H]^+$: 322.1362, found: 322.1373.

***N*-((2-chloro-[1,1'-biphenyl]-4-yl)methyl)-3-phenylpropan-1-amine HCl salt (18)**

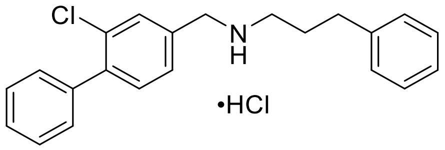

Prepared by general method **F** using hydrocinnamaldehyde (33 µL, 0.25 mmol), benzylamine **1c** (82 mg, 0.38 mmol), 1,2-dichloroethane (0.89 mL) and sodium triacetoxyborohydride (74 mg, 0.35 mmol). The crude product was purified by flash column chromatography (silica gel, gradient elution: 0:100 MeOH:CH_2Cl_2 to 10:90 MeOH:CH_2Cl_2) to provide free amine **18** (50 mg, 0.15 mmol, 60%). The free amine (45 mg, 0.13 mmol) was then dissolved in CH_2Cl_2 (0.5 mL) and HCl (2M in Et_2O) (0.65 mL, 1.30 mmol) added dropwise. The reaction was stirred for 1 hour before the precipitate was filtered, washed with cold Et_2O and dried to provide the title compound **18** as a white solid (35 mg, 0.12 mmol, 48%):

R_f 0.42 (amine) (1:9 MeOH:CH_2Cl_2); **m.p.** 203-205 °C; **IR** υ_{max} 2942, 2740, 1444, 764, 697.; **^{1}H NMR** (500 MHz, DMSO-d_6) δ 9.31 (s, 2H), 7.82 (d, *J* = 1.7 Hz, 1H), 7.59 (dd, *J* = 7.9, 1.8 Hz, 1H), 7.53 – 7.39 (m, 6H), 7.35 – 7.27 (m, 2H), 7.27 – 7.17 (m, 3H), 4.19 (s, 2H), 2.93 (app d, *J* = 3.9 Hz, 2H), 2.67 (t, *J* = 7.6 Hz, 2H), 1.98 (dt, *J* = 15.5, 7.8 Hz. 2H); **^{13}C NMR** (126 MHz, DMSO-d_6) δ 140.7, 140.2, 138.1, 133.3, 131.6, 131.4, 131.3, 129.2, 129.1, 128.4, 128.3, 128.3, 128.0, 126.1, 48.8, 46.2, 31.9, 27.1; **HRMS** (ESI) calcd for $[C_{22}H_{22}N^{35}Cl + H]^+$: 336.1519, found: 336.1495.

***N*-((1H-Pyrrol-2-yl)methyl)-1-(2-chloro-[1,1'-biphenyl]-4-yl)methanamine HCl salt (19)**

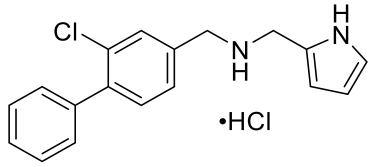

Prepared by general method **F** using pyrrole-2-carboxaldehyde (24 mg, 0.25 mmol), benzylamine **1c** (82 mg, 0.38 mmol), 1,2-dichloroethane (0.89 mL) and sodium triacetoxyborohydride (74 mg, 0.35 mmol). The crude product was purified by flash column chromatography (silica gel, gradient elution: 2:98 MeOH:CH_2Cl_2 to 5:95 MeOH:CH_2Cl_2) to provide free amine **19** (35 mg, 0.12 mmol, 47%). The free amine (35 mg, 0.12 mmol) was then dissolved in CH_2Cl_2 (0.5 mL) and HCl (2M in Et_2O) (0.59 mL, 1.18 mmol) added dropwise. The reaction was stirred for 1 hour before the precipitate was filtered, washed with cold Et_2O and dried to provide the title compound **19** as a brown solid (31 mg, 0.10 mmol, 79%):

R_f 0.60 (amine) (1:9 MeOH:CH_2Cl_2); **m.p.** 246-248 °C; **IR** υ_{max} 3305, 2938, 2784, 1430, 724, 698; **^{1}H NMR** (400 MHz, DMSO-d_6) δ 11.05 (br s, 1H), 9.61 (br s, 2H), 7.77 (d, *J* = 1.6 Hz, 1H), 7.56 (dd, *J* = 7.9, 1.7 Hz, 1H), 7.51 – 7.38 (m, 6H), 6.85 (td, *J* = 2.6, 1.6 Hz, 1H), 6.25 (app t, *J* = 3.6 Hz, 1H), 6.06 (dd, *J* = 5.8, 2.6 Hz, 1H), 4.15 (s, 4H); **^{13}C NMR** (101 MHz, DMSO-d_6) δ 140.3, 138.2, 133.2, 131.7, 131.42, 131.36, 129.3, 129.2, 128.4, 128.1, 121.4, 119.4, 110.3, 108.2, 48.3, 43.0; **HRMS** (ESI) calcd for $[C_{18}H_{17}N_2{}^{35}Cl + H]^+$: 297.1158, found: 297.1153.

***N*-((2-Chloro-[1,1'-biphenyl]-4-yl)methyl)-2-(1*H*-imidazol-5-yl)ethan-1-amine HCl salt (20)**

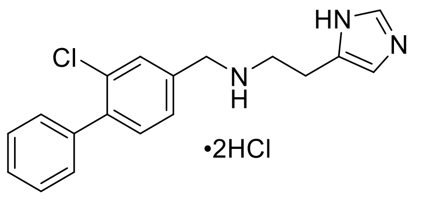

Prepared by general method **F** using **16b** (43 mg, 0.20 mmol), histamine (35 mg, 0.30 mmol), 1,2-dichloroethane (0.71 mL) and sodium triacetoxyborohydride (59 mg, 0.28 mmol). The crude product was purified by flash column chromatography (silica gel, 2:8 MeOH:CH_2Cl_2) to provide free amine **20** (29 mg, 0.09 mmol, 47%). The free amine (13 mg, 0.04 mmol) was then dissolved in CH_2Cl_2 (0.5 mL) and HCl (2M in Et_2O) (0.20 mL, 0.40 mmol) added dropwise. The reaction was stirred for 1 hour before the precipitate was filtered, washed with cold Et_2O and dried to provide the title compound **20** as a white solid (13 mg, 0.03 mmol, 83%):

R_f 0.20 (amine) (1:9 MeOH:CH_2Cl_2); **m.p.** 252-253 °C; **IR** υ_{max} 2901, 2769, 1444, 1081, 826, 764, 697; **^{1}H NMR** (500 MHz, DMSO-d_6) δ 14.36 (s, 2H), 9.58 (br s, 2H), 9.01 (s, 1H), 7.84 (d, *J* = 1.6 Hz, 1H), 7.62 (dd, *J* = 7.9, 1.7 Hz, 1H), 7.55 (s, 1H), 7.52 – 7.46 (m, 4H), 7.46 – 7.41 (m, 4H), 4.24 (s, 2H), 3.29 (t, *J* = 7.5 Hz, 2H, under the solvent peak), 3.15 (t, *J* = 7.4 Hz, 2H); **^{13}C NMR** (126 MHz, DMSO-d_6) δ 140.3, 138.1, 134.2, 133.1, 131.7, 131.4, 131.3, 129.3, 129.1, 128.9, 128.4, 128.0, 116.8, 48.9, 44.9, 21.1; **HRMS** (ESI) calcd for $[C_{18}H_{18}{}^{35}ClN_3 + H]^+$: 312.1267, found: 312.1247.

***N*-((2-Chloro-[1,1'-biphenyl]-4-yl)methyl)-2-(1*H*-imidazol-2-yl)ethan-1-amine HCl salt (21)**

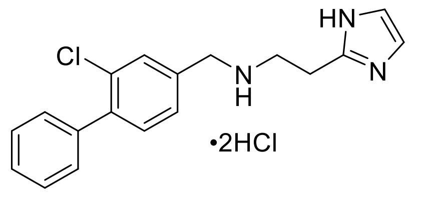

Prepared by general method **G** using **16b** (20 mg, 0.09 mmol), 2-(2-ammonioethyl)-1H-imidazol-3-ium 2,2,2-trifluoroacetate (46 mg, 0.14 mmol), MeOH (0.32 mL), NEt_3 (25 µL, 0.18 mmol) and sodium triacetoxyborohydride (27 mg, 0.13 mmol). The crude product was purified by flash column chromatography (silica gel, gradient elution: 2:98 MeOH:CH_2Cl_2 to 5:95 MeOH:CH_2Cl_2) to provide free amine **21** (20 mg, 0.06 mmol, 70%). The free amine (10 mg, 0.03 mmol) was then dissolved in CH_2Cl_2 (0.5 mL) and HCl (2M in Et_2O) (0.15 mL, 0.30 mmol) added dropwise. The reaction was stirred for 1

hour before the precipitate was filtered, washed with cold Et_2O and dried to provide the title compound **21** as a white solid (9 mg, 0.02 mmol, 75%):

R*f* 0.37 (amine) (1:9 MeOH:CH_2Cl_2); **m.p.** 230-232 °C; **IR** υ_{max} 2743, 1455, 851, 761, 698; **^{1}H NMR** (500 MHz, DMSO-d_6) δ 7.83 (d, *J* = 1.5 Hz, 1H), 7.62 (dd, *J* = 7.9, 1.6 Hz, 1H), 7.61 (s, 2H), 7.52 – 7.47 (m, 3H), 7.46 – 7.41 (m, 3H), 4.27 (s, 2H), 3.45 (s, 4H); **^{13}C NMR** (126 MHz, DMSO-d_6) δ 143.1, 140.4, 138.1, 133.0, 131.7, 131.4, 129.3, 129.1, 128.4, 128.1, 119.3, 49.0, 43.3, 22.5; **HRMS** (ESI) calcd for $[C_{18}H_{18}{}^{35}ClN_3 + H]^+$: 312.1267, found: 312.1284.

2-(1*H*-Benzo[d]imidazol-2-yl)-*N*-((2-chloro-[1,1'-biphenyl]-4-yl)methyl)ethan-1-amine HCl salt (22)

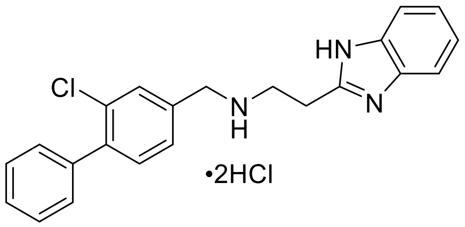

Prepared by general method **F** using **16b** (43 mg, 0.20 mmol), 2-(1H-benzo[d]imidazol-2-yl)ethan-1-amine (48 mg, 0.30 mmol), 1,2-dichloroethane (0.71 mL) and sodium triacetoxyborohydride (59 mg, 0.28 mmol). The crude product was purified by flash column chromatography (silica gel, gradient elution: 0:100 MeOH:CH_2Cl_2 to 2:98 MeOH:CH_2Cl_2) to provide free amine **22** (22 mg, 0.06 mmol, 31% yield). The free amine (13 mg, 0.04 mmol) was then dissolved in CH_2Cl_2 (0.5 mL) and HCl (2M in Et_2O) (0.20 mL, 0.40 mmol) added dropwise. The reaction was stirred for 1 hour before the precipitate was filtered, washed with cold Et_2O and dried to provide the title compound **22** as a white solid (15 mg, 0.03 mmol, 86%):

R*f* 0.21 (amine) (1:19 MeOH:CH_2Cl_2); **m.p.** 261-263 °C; **IR** υ_{max} 2612, 828, 742, 700, 619; **^{1}H NMR** (500 MHz, DMSO-d_6) δ 7.88 (d, *J* = 1.6 Hz, 1H), 7.78 (dd, *J* = 6.1, 3.1 Hz, 2H), 7.66 (dd, *J* = 7.9, 1.7 Hz, 1H), 7.53 – 7.46 (m, 5H), 7.46 – 7.39 (m, 3H), 4.33 (s, 2H), 3.70 (t, *J* = 6.6 Hz, 2H), 3.64 (t, *J* = 6.7 Hz, 2H); **^{13}C NMR** (126 MHz, DMSO-d_6): 150.2, 140.3, 138.1, 133.0, 131.7, 131.4, 131.3, 129.3, 129.1, 128.3, 128.0, 125.2, 113.9, 48.9, 43.2, 23.6; **HRMS** (ESI) calcd for $[C_{22}H_{20}ClN_3 + H]^+$: 362.1424, found: 362.1443.

2-(1*H*-Benzo[*d*]imidazol-2-yl)-*N*-((2-chloro-2'-ethyl-[1,1'-biphenyl]-4-yl)methyl)ethan-1-amine HCl salt (23)

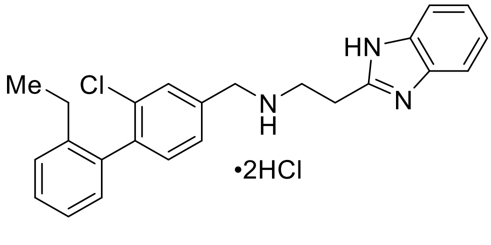

Prepared by general method **F** using **23b** (50 mg, 0.20 mmol), 2-(1*H*-benzimidazol-2-yl)ethylamine (49 mg, 0.30 mmol), 1,2-dichloroethane (0.70 mL), DMF (0.70 mL) and sodium triacetoxyborohydride (65 mg, 0.31 mmol). The crude product was purified by flash column chromatography (silica gel, gradient elution: 0:100 MeOH:CH_2Cl_2 to 9:91 MeOH:CH_2Cl_2) and the resulting residue was then dissolved in CH_2Cl_2 (2.0 mL) and HCl (4M in 1,4-dioxane) (0.16 mL, 0.64 mmol) added dropwise. The reaction was stirred for 30 minutes before the mixture was concentrated *in vacuo* and triturated with Et_2O to provide the title compound **23** as a white solid (40 mg, 0.08 mmol, 42%):

R*f* 0.25 (amine) (1:10 MeOH:$CHCl_3$); **m.p.** 228-230 °C; **IR** $υ_{max}$ 3200, 2000, 2724, 2616, 1628, 1576, 1517, 1461, 1388, 1227, 1077, 1024, 1005; **^{1}H NMR** (400 MHz, DMSO-d_6) δ 7.90 (d, *J* = 1.4 Hz, 1H), 7.79 (dd, *J* = 6.2, 3.2 Hz, 2H), 7.66 (dd, *J* = 7.8, 1.4 Hz, 1H), 7.52 (dd, *J* = 6.2, 3.2 Hz, 2H), 7.42 – 7.32 (m, 3H), 7.31 - 7.24 (m, 1H), 7.05 (d, *J* = 7.4 Hz, 1H), 4.35 (s, 2H), 3.78 – 3.64 (m, 4H), 2.47 – 2.25 (m, 2H), 0.99 (t, *J* = 7.5 Hz, 3H); **^{13}C NMR** (126 MHz, DMSO-d_6) δ 150.3, 141.4, 140.4, 137.7, 133.2, 132.5, 131.7, 130.8, 129.4, 128.9, 128.5, 128.4, 125.8, 125.3, 114.0, 49.1, 43.5, 25.7, 23.6, 15.2; **HRMS** (ESI) calcd for $[C_{24}H_{24}N_3{}^{35}Cl + H]^+$: 390.1737, found: 390.1725.

***tert*-Butyl (2-(7-methyl-1*H*-benzo[*d*]imidazol-2-yl)ethyl)carbamate (24d)**

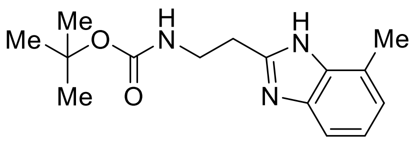

To a stirred solution of Boc-β-Ala-OH (150 mg, 0.79 mmol) in DMF (5.0 mL) was added 3-methyl-1,2-phenylenediamine (145 mg, 1.19 mmol), HBTU (390 mg, 1.03 mmol) and NEt_3 (0.22 mL, 1.59 mmol) at 0 °C under nitrogen. After stirring at room temperature for 2.5 hours, the reaction mixture was diluted with EtOAc and washed with H_2O, saturated aqueous $NaHCO_3$ and saturated aqueous NaCl. The organic phase was dried over Na_2SO_4, filtered and concentrated *in vacuo*. The crude product was purified by flash column chromatography (silica gel, gradient elution: 35:65 EtOAc:pet ether to 80:20 EtOAc:pet ether) and the resulting residue was dissolved with acetic acid (3.0 mL) and stirred at 65 °C for 4 hours. The reaction mixture was cooled to 0 °C, poured into a saturated aqueous solution of $NaHCO_3$ and extracted three times with EtOAc. The combined organic layers were washed with a saturated aqueous solution of NaCl, dried over Na_2SO_4, filtered and concentrated *in vacuo*. The crude product was purified by flash column chromatography (silica gel, gradient elution: 50:50 EtOAc:pet ether to 90:10 EtOAc:pet ether) to provide the title compound **24d** as a white solid (151 mg, 0.55 mmol, 69%):

R*f* 0.18 (4:1 EtOAc:pet ether); **m.p.** 195-197 °C; **IR** $υ_{max}$ 3400, 2300, 3358, 2979, 1684, 1518, 1440, 1367, 1276, 1253, 1172; **^{1}H NMR** (400 MHz, $CDCl_3$) δ 7.38 (br s, 1H), 7.12 (dd, *J* = 7.8, 7.4 Hz, 1H), 7.02 (d, *J* = 7.4 Hz, 1H), 5.20 (br s, 1H), 3.69 (dd, *J* = 6.5, 6.0 Hz, 2H), 3.17 (t, *J* = 6.0 Hz, 2H), 2.57 (s, 3H), 1.42 (s, 9H); **^{13}C NMR** (126 MHz, $CDCl_3$) δ 157.0, 151.8, 122.7, 122.2, 80.2, 37.9, 3.6, 28.4, 16.9; **HRMS** (ESI) calcd for $[C_{15}H_{21}N_3O_2 + H]^+$: 276.1712, found: 276.1705.

N-((2-Chloro-2'-ethyl-[1,1'-biphenyl]-4-yl)methyl)-2-(7-methyl-1H-benzo[d]imidazol-2-yl)ethan-1-amine HCl salt (24)

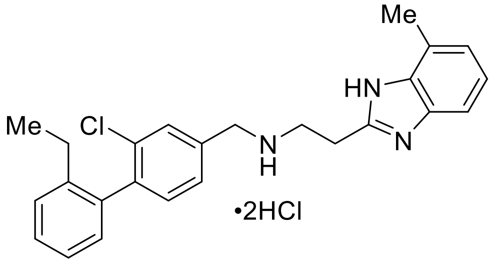

To a stirred solution of **24d** (110 mg, 0.40 mmol) in CH_2Cl_2 (1.0 mL) was added trifluoroacetic acid (1.0 mL) at room temperature. The reaction was stirred for 1.5 hours before the volatiles were removed *in vacuo* and the resulting residue used directly in general method **F** along with **23b** (50 mg, 0.20 mmol), NEt_3 (0.11 mL, 0.80 mmol), 1,2-dichloroethane (1.0 mL), DMF (1.0 mL) and sodium triacetoxyborohydride (130 mg, 0.61 mmol). The crude product was purified by flash column chromatography (silica gel, gradient elution: 0:100 MeOH:CH_2Cl_2 to 10:90 MeOH:CH_2Cl_2) and the resulting residue was then dissolved in CH_2Cl_2 (2.0 mL) and HCl (4M in 1,4-dioxane) (0.20 mL, 0.80 mmol) added dropwise. The reaction was stirred for 30 minutes before the mixture was concentrated *in vacuo* and triturated with Et_2O to provide the title compound **24** as a beige solid (40 mg, 0.08 mmol, 41%):

R_f 0.29 (amine) (1:10 MeOH:$CHCl_3$); **m.p.** 219-222 °C; **IR** υ_{max} 3200, 2000, 2724, 2615, 2421, 1636, 1581, 1447, 1388, 1229, 1078, 1019, 1005; **^{1}H NMR** (400 MHz, DMSO-d_6) δ 7.89 (d, *J* = 1.3 Hz, 1H), 7.66 (dd, *J* = 7.9, 1.3 Hz, 1H), 7.59 (d, *J* = 8.2 Hz, 1H), 7.44 – 7.35 (m, 1H), 7.32 (d, *J* = 7.4 Hz, 1H), 7.30 – 7.25 (m, 5H), 7.05 (d, *J* = 7.4 Hz, 1H), 4.35 (s, 2H), 3.78 – 3.67 (m, 4H), 2.63 (s, 3H), 2.47 – 2.25 (m, 2H), 0.99 (t, *J* = 7.5 Hz, 3H); **^{13}C NMR** (126 MHz, DMSO-d_6) δ 150.0, 141.5, 140.4, 137.7, 133.2, 132.5, 131.7, 130.8, 129.4, 128.9, 128.5, 128.4, 125.8, 125.4, 124.3, 111.3, 49.1, 43.6, 25.7, 23.5, 16.8, 15.2; **HRMS** (ESI) calcd for $[C_{25}H_{26}N_3{}^{35}Cl + H]^+$: 404.1893, found: 404.1907.

3-((tert-Butyloxycarbonyl)((2-chloro-2'-ethyl-[1,1'-biphenyl]-4-yl)methyl)amino)propanoic acid (25e)

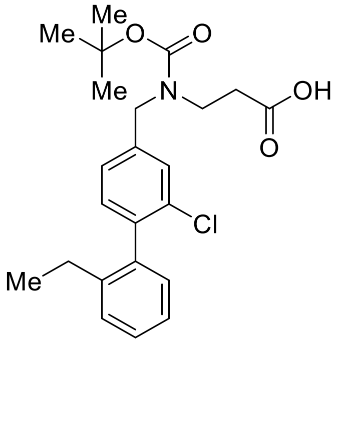

The unprotected amine of **25e** was prepared by general method **F** using **23b** (450 mg, 1.84 mmol), β-alanine methyl ester hydrochloride (385 mg, 2.76 mmol), NEt_3 (0.39 mL, 2.76 mmol), 1,2-dichloroethane (7.0 mL), and sodium triacetoxyborohydride (585 mg, 2.76 mmol). The crude amine product was then dissolved in CH_2Cl_2 (8.0 mL) and Boc_2O (1.20 g, 5.52 mmol), NEt_3 (0.77 mL, 5.52 mmol) and DMAP (22 mg, 0.18 mmol) were added. The reaction mixture was stirred at room temperature overnight before diluting with CH_2Cl_2 and washing with H_2O, a saturated aqueous solution of $NaHCO_3$ and a saturated aqueous solution of NaCl. The organic layer was dried over Na_2SO_4, filtered and concentrated *in vacuo*. The crude product was purified by flash column chromatography (silica gel, gradient elution: 1:99 EtOAc:pet ether to 20:80 EtOAc:pet ether) and the resulting residue was dissolved in THF (7.0 mL) and H_2O (7.0 mL). To the solution was added lithium hydroxide monohydrate (126 mg, 3.00 mmol) and the reaction mixture stirred at room temperature for 1.5 hours. The mixture was cooled to 0 °C, quenched with 1M aqueous HCl and extracted three times with CH_2Cl_2. The combined organic extracts were washed with a saturated aqueous solution of NaCl, dried over Na_2SO_4, filtered and concentrated *in vacuo* to provide the title compound **25e** as a colourless oil (730 mg, 1.75 mmol, 95%):

R*f* 0.50 (1:10 MeOH:$CHCl_3$); **IR** υ_{max} 3385, 2554, 2973, 2929, 1734, 1693, 1476, 1415, 1394, 1366, 1273, 1247, 1158, 1117; **^{1}H NMR** (400 MHz, $CDCl_3$) δ 7.38 – 7.30 (m, 3H), 7.23 (dd, *J* = 7.0, 1.8 Hz, 1H), 7.20 (d *J* = 7.7 Hz, 1H), 7.15 (d, *J* = 7.7 Hz, 1H), 7.10 (dd, *J* = 7.5, 1.0 Hz, 1H), 4.50 (br s, 2H), 3.55 (br s, 2H), 2.65 (br s, 2H), 2.54 – 2.33 (m, 2H), 1.49 (br s, 9H), 1.05 (t, *J* = 7.5 Hz, 3H); **^{13}C NMR** (126 MHz, $CDCl_3$) δ 176.2, 155.6, 142.2, 139.4, 138.5, 133.7, 131.4, 129.7, 128.2, 125.5, 80.4, 53.5, 43.0, 33.2, 28.4, 26.2, 15.0; **HRMS** (ESI) calcd for $[C_{23}H_{28}NO_4{}^{35}Cl + Na]^+$: 440.1605, found: 440.1603.

N-((2-Chloro-2'-ethyl-[1,1'-biphenyl]-4-yl)methyl)-2-(7-nitro-1*H*-benzo[*d*]imidazol-2-yl)ethan-1-amine HCl salt (25)

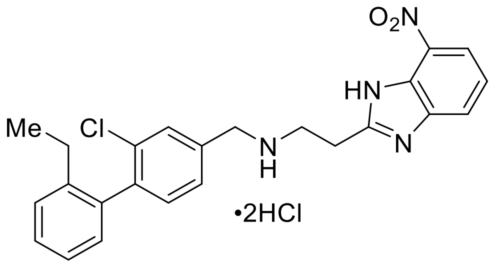

Prepared by general method **H** using **25e** (75 mg, 0.18 mmol), 3-nitro-1,2-phenylenediamine (55 mg, 0.36 mmol), HBTU (136 mg, 0.36 mmol) and NEt_3 (0.08 mL, 0.54 mmol) in DMF (2.0 mL). The crude product was first purified by flash column chromatography (silica gel, gradient elution: 35:65 EtOAc:pet ether to 50:50 EtOAc:pet ether) before dissolving in acetic acid (2.0 mL) and heating to 70 °C overnight. The boc-protected amine of **25** was recovered by flash column chromatography (silica gel, gradient elution: 15:85 EtOAc:pet ether to 50:50 EtOAc:pet ether) as an orange oil (69 mg, 0.13 mmol, 72%) and then deprotected by general method **J** using amine (25 mg, 0.05 mmol) and HCl (4M in 1,4-dioxane) (1.0 mL, 4.0 mmol) to provide the title compound **25** as a yellow solid (20 mg, 0.04 mmol, 84%):

R_f 0.53 (amine) (1:10 MeOH:$CHCl_3$); **m.p.** 178-180 °C; **IR** υ_{max} 3600, 2000, 2715, 2613, 2420, 1615, 1570, 1539, 1502, 1456, 1389, 1345, 1303, 1198, 1076, 1005; **^{1}H NMR** (400 MHz, DMSO-d_6) δ 8.16 (d, *J* = 8.1 Hz, 1H), 8.10 (d, *J* = 7.8 Hz, 1H), 7.87 (d, *J* = 1.5 Hz, 1H), 7.64 (dd, *J* = 7.8, 1.5 Hz, 1H), 7.47 – 7.35 (m, 4H), 7.31 – 7.24 (m, 1H), 7.07 (d, *J* = 7.4 Hz, 1H), 4.34 (s, 2H), 3.62 – 3.47 (m, 4H), 2.48 – 2.26 (m, 2H), 0.99 (t, *J* = 7.5 Hz, 3H); **^{13}C NMR** (126 MHz, DMSO-d_6) δ 155.0, 141.4, 140.4, 137.7, 133.3, 132.5, 131.7, 130.9, 130.7, 129.4, 128.9, 128.8, 128.4, 125.8, 125.5, 121.7, 121.6, 118.8, 49.2, 44.2, 25.7, 24.9, 15.1; **HRMS** (ESI) calcd for $[C_{24}H_{23}N_4O_2{}^{35}Cl + H]^+$: 435.1588, found: 435.1598.

N-((2-Chloro-2'-ethyl-[1,1'-biphenyl]-4-yl)methyl)-2-(7-methoxy-1*H*-benzo[*d*]imidazol-2-yl)ethan-1-amine HCl salt (26)

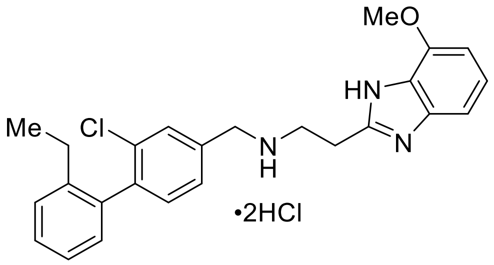

Prepared by general method **H** using **25e** (50 mg, 0.12 mmol), 3-methoxy-1,2-phenylenediamine (25 mg, 0.18 mmol), HBTU (68 mg, 0.18 mmol) and NEt_3 (0.05 mL, 0.36 mmol) in DMF (1.5 mL). The crude product was first purified by flash column chromatography (silica gel, gradient elution: 20:80 EtOAc:pet ether to 75:25 EtOAc:pet ether) before dissolving in acetic acid (2.0 mL) and heating to 70 °C overnight. The boc-protected amine of **26** was recovered by flash column chromatography (silica gel, gradient elution: 20:80 EtOAc:pet ether to 75:25 EtOAc:pet ether) as a brown foam (54 mg, 0.10 mmol, 87%) and then deprotected by general method **J** using amine (48 mg, 0.09 mmol) and HCl (4M in 1,4-dioxane) (1.0 mL, 4.0 mmol) to provide the title compound **26** as a white solid (37 mg, 0.07 mmol, 81%):

R_f 0.33 (amine) (1:10 MeOH:$CHCl_3$); **m.p.** 142-144 °C; **IR** υ_{max} 3600, 2000, 2729, 2611, 1628, 1584, 1453, 1422, 1267, 1108; **^{1}H NMR** (400 MHz, DMSO-d_6) δ 7.89 (d, *J* = 1.6 Hz, 1H), 7.66 (dd, *J* = 7.8, 1.6 Hz, 1H), 7.46 (t, *J* = 8.2 Hz, 1H), 7.41 – 7.33 (m, 4H), 7.30 – 7.25 (m, 1H), 7.12 (d, *J* = 8.0 Hz, 1H), 7.05 (d, *J* = 7.4 Hz, 1H), 4.33 (s, 2H), 4.03 (s, 3H), 3.75 – 3.62 (m, 4H), 2.47 – 2.25 (m, 2H), 0.99 (t, *J* = 7.5 Hz, 3H); **^{13}C NMR** (126 MHz, DMSO-d_6) δ 149.7, 147.0, 141.5, 140.4, 137.7, 133.2, 132.5, 131.7, 130.8, 129.4, 128.9, 128.5, 128.4, 126.6, 125.8, 121.8, 106.3, 106.0, 56.2, 49.1, 43.4, 25.7, 23.4, 15.2; **HRMS** (ESI) calcd for $[C_{25}H_{26}N_3O^{35}Cl + H]^+$: 420.1842, found: 420.1859.

N-((2-Chloro-2'-ethyl-[1,1'-biphenyl]-4-yl)methyl)-2-(6-methyl-1*H*-benzo[*d*]imidazol-2-yl)ethan-1-amine HCl salt (27)

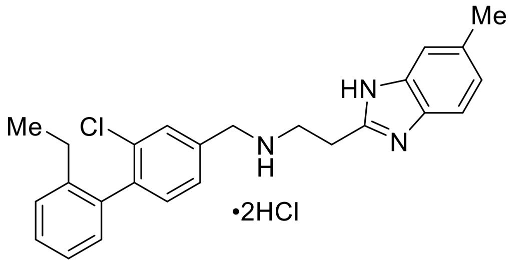

Prepared by general method **H** using **25e** (50 mg, 0.12 mmol), 3,4-diaminotoluene (22 mg, 0.18 mmol), HBTU (68 mg, 0.18 mmol) and NEt_3 (0.05 mL, 0.36 mmol) in DMF (1.5 mL). The crude product was first purified by flash column chromatography (silica gel, gradient elution: 20:80 EtOAc:pet ether to 75:25 EtOAc:pet ether) before dissolving in acetic acid (2.0 mL) and heating to 70 °C overnight. The boc-protected amine of **27** was recovered by flash column chromatography (silica gel, gradient elution: 20:80 EtOAc:pet ether to 75:25 EtOAc:pet ether) as a yellow foam (54 mg, 0.11 mmol, 89%) and then deprotected by general method **J** using amine (48 mg, 0.10 mmol) and HCl (4M in 1,4-dioxane) (1.0 mL, 4.0 mmol) to provide the title compound **27** as a pale pink solid (34 mg, 0.07 mmol, 75%):

R_f 0.32 (amine) (1:10 MeOH:$CHCl_3$); **m.p.** 232-234 °C; **IR** υ_{max} 3600, 2000, 2734, 2613, 1627, 1575, 1456, 1234, 1077; **^{1}H NMR** (400 MHz, DMSO-d_6) δ 7.89 (d, *J* = 1.5 Hz, 1H), 7.69 – 7.64 (m, 2H), 7.59 (s, 1H), 7.41 – 7.33 (m, 3H), 7.31 – 7.24 (m, 1H), 7.05 (d, *J* = 7.3 Hz, 1H), 4.34 (s, 2H), 3.75 – 3.64 (m, 4H), 2.49 (s, 3H), 2.47 – 2.25 (m, 2H), 0.99 (t, *J* = 7.5 Hz, 3H); **^{13}C NMR** (126 MHz, DMSO-d_6) δ 141.5, 140.4, 137.7, 135.4, 133.2, 132.5, 131.7, 130.8, 129.4, 128.9, 128.5, 128.4, 126.9, 125.8, 113.6, 113.4, 49.1, 43.4, 25.7, 23.6, 21.2, 15.2; **HRMS** (ESI) calcd for $[C_{25}H_{26}N_3{}^{35}Cl + H]^+$: 404.1893, found: 404.1898.

***N*-((2-Chloro-2'-ethyl-[1,1'-biphenyl]-4-yl)methyl)-2-(6-nitro-1*H*-benzo[*d*]imidazol-2-yl)ethan-1-amine HCl salt (28)**

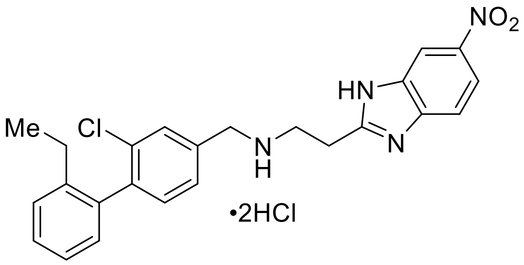

Prepared by general method **H** using **25e** (75 mg, 0.18 mmol), 4-nitro-1,2-phenylenediamine (55 mg, 0.36 mmol), HBTU (136 mg, 0.36 mmol) and NEt_3 (0.08 mL, 0.54 mmol) in DMF (2.0 mL). The crude product was first purified by flash column chromatography (silica gel, gradient elution: 15:85 EtOAc:pet ether to 65:35 EtOAc:pet ether) before dissolving in acetic acid (2.0 mL) and heating to 70 °C overnight. The free amine of **28** was recovered by flash column chromatography (silica gel, gradient elution: 0:100 MeOH:CH_2Cl_2 to 10:90 MeOH:CH_2Cl_2) and the resulting residue was then dissolved in CH_2Cl_2 (1.0 mL) and HCl (4M in 1,4-dioxane) (0.10 mL, 0.40 mmol) added dropwise. The reaction was stirred for 30 minutes before the mixture was concentrated *in vacuo* and triturated with Et_2O to provide the title compound **28** as a yellow solid (17 mg, 0.03 mmol, 19%):

R_f 0.46 (amine) (1:10 MeOH:$CHCl_3$); **m.p.** 203-205 °C; **IR** υ_{max} 3600, 2000, 2714, 2645, 2588, 2425, 1636, 1578, 1542, 1508, 1454, 1345, 1223, 1077, 1018; **^{1}H NMR** (400 MHz, DMSO-d_6) δ 8.16 (dd, *J* = 8.8, 2.2 Hz, 1H), 7.88 (d, *J* = 1.3 Hz, 1H), 7.77 (d, *J* = 8.8 Hz, 1H), 7.64 (dd, *J* = 7.8, 1.3 Hz, 1H), 7.42 – 7.35 (m, 3H), 7.31 – 7.25 (m, 1H), 7.06 (d, *J* = 7.4 Hz, 1H), 4.34 (s, 2H), 3.61 – 3.46 (m, 4H), 2.48 – 2.26 (m, 2H), 0.99 (t, *J* = 7.5 Hz, 3H); **^{13}C NMR** (126 MHz, DMSO-d_6) δ 156.5, 143.3, 141.8, 140.8, 138.1, 133.7, 132.9, 132.1, 131.2, 129.8, 129.3, 128.8, 126.2, 118.5, 114.9, 112.0, 49.6, 44.3, 26.1, 25.5, 15.5; **HRMS** (ESI) calcd for $[C_{24}H_{23}N_4O_2{}^{35}Cl + H]^+$: 435.1588, found: 435.1596.

N-((2-Chloro-2'-ethyl-[1,1'-biphenyl]-4-yl)methyl)-2-(6-methoxy-1*H*-benzo[*d*]imidazol-2-yl)ethan-1-amine HCl salt (29)

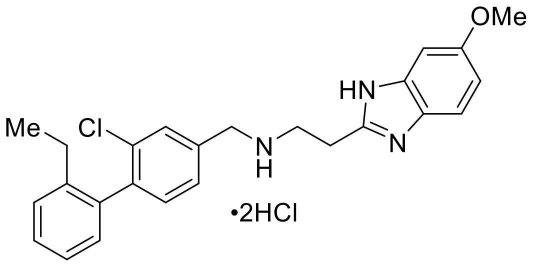

Prepared by general method **H** using **25e** (45 mg, 0.11 mmol), 4-methoxy-1,2-phenylenediamine hydrochloride (34 mg, 0.16 mmol), HBTU (61 mg, 0.16 mmol) and NEt_3 (0.08 mL, 0.54 mmol) in DMF (1.5 mL). The crude product was first purified by flash column chromatography (silica gel, gradient elution: 20:80 EtOAc:pet ether to 75:25 EtOAc:pet ether) before dissolving in acetic acid (2.0 mL) and heating to 70 °C overnight. The free amine of **29** was recovered by flash column chromatography (silica gel, gradient elution: 0:100 MeOH:CH_2Cl_2 to 10:90 MeOH:CH_2Cl_2) and the resulting residue was then dissolved in CH_2Cl_2 (2.0 mL) and HCl (4M in 1,4-dioxane) (0.10 mL, 0.40 mmol) added dropwise. The reaction was stirred for 30 minutes before the mixture was concentrated *in vacuo* and triturated with Et_2O to provide the title compound **29** as a white solid (17 mg, 0.04 mmol, 32%):

R_f 0.35 (amine) (1:10 MeOH:$CHCl_3$); **m.p.** 208-210 °C; **IR** υ_{max} 3600, 2000, 2744, 1629, 1496, 1458, 1269, 1161, 1025; **^{1}H NMR** (400 MHz, DMSO-d_6) δ 7.88 (d, *J* = 1.5 Hz, 1H), 7.68 (d, *J* = 9.0 Hz, 1H), 7.65 (dd, *J* = 7.9, 1.5 Hz, 1H), 7.42 – 7.35 (m, 3H), 7.31 – 7.25 (m, 1H), 7.23 (d, *J* = 2.2 Hz, 1H), 7.13 (dd, *J* = 9.0, 2.2 Hz, 1H), 7.05 (d, *J* = 7.4 Hz, 1H), 4.34 (s, 2H), 3.86 (s, 3H), 3.72 – 3.61 (m, 4H), 2.48 – 2.25 (m, 2H), 0.99 (t, *J* = 7.5 Hz, 3H); **^{13}C NMR** (126 MHz, DMSO-d_6) δ 157.7, 149.5, 141.4, 140.4, 137.7, 133.2, 132.5, 131.7, 130.8, 129.4, 128.9, 128.5, 128.4, 125.8, 115.0, 114.8, 96.4, 56.0, 49.2, 43.5, 25.7, 23.6, 15.3; **HRMS** (ESI) calcd for $[C_{25}H_{26}N_3O^{35}Cl + H]^+$: 420.1842, found: 420.1859.

***tert*-Butyl (2-(5-chloro-1*H*-benzo[*d*]imidazol-2-yl)ethyl)carbamate (30d)**

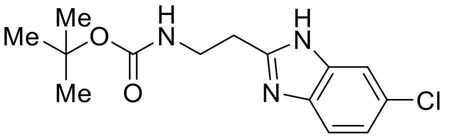

To a stirred solution of Boc-β-Ala-OH (300 mg, 1.59 mmol) in DMF (10 mL) was added 4-chloro-1,2-phenylenediamine (339 mg, 2.38 mmol), HBTU (724 mg, 1.91 mmol) and diisopropylethylamine (0.56 mL, 4.11 mmol) at 0 °C under nitrogen. After stirring at room temperature for 2.5 hours, the reaction mixture was diluted with EtOAc and washed with H_2O, saturated aqueous $NaHCO_3$ and saturated aqueous NaCl. The organic phase was dried over Na_2SO_4, filtered and concentrated *in vacuo*. The crude product was purified by flash column chromatography (silica gel, gradient elution: 20:80 EtOAc:pet ether to 80:20 EtOAc:pet ether) and the resulting residue was dissolved with acetic acid (3.0 mL) and stirred at 65 °C for 4 hours. The reaction mixture was cooled to 0 °C, poured into a saturated aqueous solution of $NaHCO_3$ and extracted three times with EtOAc. The combined organic layers were washed with a saturated aqueous solution of NaCl, dried over Na_2SO_4, filtered and concentrated *in vacuo*. The crude product was purified by flash column chromatography (silica gel, gradient elution: 50:50 EtOAc:pet ether to 85:15 EtOAc:pet ether) to provide the title compound **30d** as a pale red foam (217 mg, 1.29 mmol, 81%):

$\mathbf{R}_f$ 0.30 (4:1 EtOAc:pet ether); **IR** υ_{max} 3600 , 2000, 2977, 1682, 1622, 1513, 1447, 1414, 1392, 1365, 1275, 1249, 1162, 1059; **^{1}H NMR** (400 MHz, $CDCl_3$) δ 7.53 (br s, 1H), 7.44 (d, *J* = 8.5 Hz, 1H), 7.18 (dd, *J* = 8.5, 2.0 Hz, 1H), 5.18 (br s, 1H), 3.67 (dt, *J* = 6.3, 6.0 Hz, 2H), 3.15 (t, *J* = 6.0 Hz, 2H), 1.42 (s, 9H); **^{13}C NMR** (126 MHz, $CDCl_3$) δ 157.1, 153.6, 127.8, 122.8, 80.4, 37.7, 30.7, 28.3; **HRMS** (ESI) calcd for $[C_{14}H_{18}N_3O_2{}^{35}Cl + H]^+$: 296.1166, found: 296.1151.

2-(6-Chloro-1*H*-benzo[*d*]imidazol-2-yl)-*N*-((2-chloro-2'-ethyl-[1,1'-biphenyl]-4-yl)methyl)ethan-1-amine HCl salt (30)

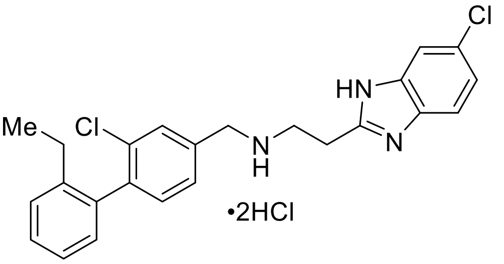

To a stirred solution of **30d** (90 mg, 0.30 mmol) in CH_2Cl_2 (1.0 mL) was added trifluoroacetic acid (1.0 mL) at room temperature. The reaction was stirred for 1.5 hours before the volatiles were removed *in vacuo* and the resulting residue used directly in general method **F** along with **23b** (49 mg, 0.20 mmol), NEt_3 (0.08 mL, 0.61 mmol), 1,2-dichloroethane (1.0 mL), DMF (1.0 mL) and sodium triacetoxyborohydride (64 mg, 0.30 mmol). The crude product was purified by flash column chromatography (silica gel, gradient elution: 0:100 MeOH:CH_2Cl_2 to 9:91 MeOH:CH_2Cl_2) and the resulting residue was then dissolved in CH_2Cl_2 (1.0 mL) and HCl (4M in 1,4-dioxane) (0.10 mL, 0.40 mmol) added dropwise. The reaction was stirred for 1 hour before the mixture was concentrated *in*

vacuo and triturated with Et_2O to provide the title compound **30** as a dark green solid (23 mg, 0.03 mmol, 15%):

R*f* 0.33 (amine) (1:10 MeOH:$CHCl_3$); **m.p.** 238-240 °C; **IR** υ_{max} 3200, 2000, 2723, 2620, 1622, 1571, 1509, 1477, 1454, 1381, 1216, 1077, 1062, 1018, 1006; **^{1}H NMR** (400 MHz, DMSO-d_6) δ 7.87 (d, *J* = 1.2 Hz, 1H), 7.80 (br s, 1H), 7.71 (d, *J* = 8.6 Hz, 1H), 7.64 (dd, *J* = 7.7, 1.3 Hz, 1H), 7.46 – 7.34 (m, 4H), 7.31 – 7.24 (m, 1H), 7.06 (d, *J* = 7.7 Hz, 1H), 4.33 (s, 2H), 3.65 – 3.50 (m, 4H), 2.48 – 2.25 (m, 2H), 0.99 (t, *J* = 7.5 Hz, 3H); **^{13}C NMR** (126 MHz, DMSO-d_6) δ 152.0, 141.4, 140.4, 137.7, 133.2, 132.5, 131.7, 130.8, 129.4, 128.9, 128.5, 128.4, 125.8, 124.6, 115.6, 114.0, 49.2, 43.6, 25.7, 24.1, 15.2; **HRMS** (ESI) calcd for $[C_{24}H_{23}N_3{}^{35}Cl_2 + H]^+$: 424.1347, found: 424.1344.

2-(1*H*-Benzo[*d*]imidazol-2-yl)-*N*-((2,6-dichloro-2'-ethyl-[1,1'-biphenyl]-4-yl)methyl)ethan-1-amine TFA salt (CAM4712)

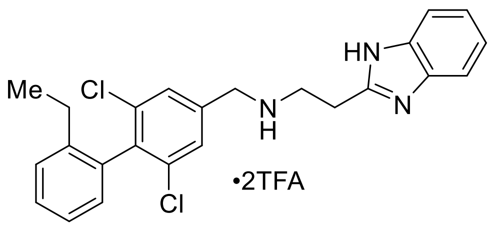

Prepared by general method **C** using **15a** (250 mg, 0.77 mmol), 2-ethylphenylboronic acid (139 mg, 0.93 mmol), $PdCl_2(dppf).CH_2Cl_2$ (32 mg, 0.39 mmol), K_3PO_4 (329 mg, 1.55 mmol), DME (2.00 mL), EtOH (0.30 mL) and H_2O (0.15 mL) and refluxed for 6 hours. The crude material (80 mg, 0.29 mmol) was then subjected to general method **F** using 2-(1*H*-benzimidazol-2-yl)ethylamine (69 mg, 0.43 mmol), 4 Å molecular sieves (50 mg), 1,2-dichloroethane (1.00 mL) and sodium triacetoxyborohydride (86 mg, 0.41 mmol). The crude amine was purified by semi-preparative HPLC (5-100% B) to provide title compound **CAM4712** as a sticky film (26 mg, 0.04 mmol, 13%):

HPLC t_r = 9.43 mins (5-100% B); **IR ν_{max}:** 2975, 1667, 1199, 798, 720; **^{1}H NMR** (500 MHz, CD_3OD): δ 7.75-7.72 (2H, m), 7.70 (2H, s) 7.53-7.49 (2H, m), 7.39-7.35 (2H, m), 7.29-7.24 (1H, m), 6.97 (1H, d, *J* = 7.4 Hz), 3.75-3.62 (4H, m), 3.30 (3H, quint, *J* = 6.1 Hz), 2.34 (2H, q *J* = 7.6), 1.04 (3H, t, *J* = 7.6 Hz); **^{13}C NMR** (126 MHz, CD_3OD): δ 150.8, 143.0, 141.9, 137.1, 136.7, 134.4, 134.2, 130.8, 130.2, 130.1, 129,6, 127.1, 126.7, 115.2, 51.2, 45.6, 27.2, 25.1, 15.1; **HRMS** (ESI) calcd for $[C_{24}H_{24}N_3{}^{35}Cl_2{}^+ + H]^+$: 424.1347, found 424.1346.

2-(1*H*-benzo[*d*]imidazol-2-yl)-*N*-((2'-ethyl-2-methyl-[1,1'-biphenyl]-4-yl)methyl)ethan-1-amine HCl salt (31)

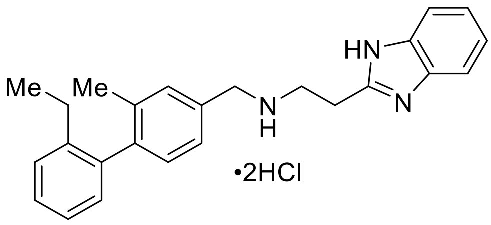

Prepared by general method **F** using **31b** (50 mg, 0.22 mmol), 2-(1*H*-benzimidazol-2-yl)ethylamine (64 mg, 0.40 mmol), DMF (2.0 mL) and sodium triacetoxyborohydride (142 mg, 0.67 mmol). The crude product was purified by flash column chromatography (silica gel, gradient elution: 0:100 MeOH:CH_2Cl_2 to 10:90 MeOH:CH_2Cl_2) and the resulting residue was then dissolved in CH_2Cl_2 (1.0 mL) and HCl (4M in 1,4-dioxane) (0.10 mL, 0.40 mmol) added dropwise. The reaction was stirred for 30 minutes before the mixture was concentrated *in vacuo* and triturated with Et_2O and MeOH to provide the title compound **31** as a beige solid (25 mg, 0.06 mmol, 25%):

R_f 0.27 (amine) (1:10 MeOH:$CHCl_3$); **m.p.** 194-196 °C; **IR** $υ_{max}$ 3600 – 2000, 2784, 2725, 2650, 2615, 1628, 1576, 1516, 1452, 1387, 1227, 1022, 1006; **^{1}H NMR** (400 MHz, DMSO-d$_6$) δ 7.84 – 7.76 (m, 2H), 7.58 – 7.46 (m, 4H), 7.39 – 7.30 (m, 2H), 7.29 – 7.22 (m, 1H), 7.17 (d, *J* = 7.7 Hz, 1H), 7.00 (d, *J* = 7.4 Hz, 1H), 4.27 (s, 2H), 3.77 – 3.63 (m, 4H), 2.44 – 2.18 (m, 2H), 2.01 (s, 3H), 0.96 (t, *J* = 7.5 Hz, 3H); **^{13}C NMR** (126 MHz, DMSO-d$_6$) δ 150.4, 141.6, 141.2, 139.8, 135.7, 131.7, 131.5, 131.0, 130.9, 129.8, 129.7, 129.1, 128.5, 127.8, 127.3, 125.8, 125.6, 125.3, 114.0, 50.0, 43.5, 25.6, 23.6, 19.8, 15.2; **HRMS** (ESI) calcd for $[C_{25}H_{27}N_3 + H]^+$: 370.2283, found: 370.2295.

2-(1*H*-benzo[*d*]imidazol-2-yl)-*N*-((2'-ethyl-2-(trifluoromethyl)-[1,1'-biphenyl]-4-yl)methyl)ethan-1-amine HCl salt (32)

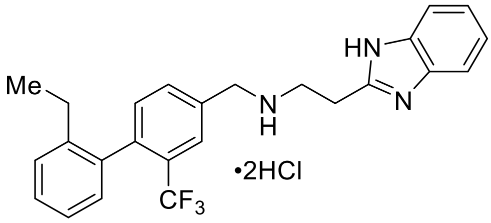

Prepared by general method **F** using **32b** (60 mg, 0.22 mmol), 2-(1*H*-benzimidazol-2-yl)ethylamine (64 mg, 0.40 mmol), DMF (2.0 mL) and sodium triacetoxyborohydride (137 mg, 0.65 mmol). The crude product was purified by flash column chromatography (silica gel, gradient elution: 0:100 MeOH:CH_2Cl_2 to 10:90 MeOH:CH_2Cl_2) and the resulting residue was then dissolved in CH_2Cl_2 (3.0 mL) and HCl (4M in 1,4-dioxane) (0.20 mL, 0.80 mmol) added dropwise. The reaction was stirred for 30 minutes before the mixture was concentrated *in vacuo* and triturated with Et_2O to provide the title compound **32** as an off-white solid (32 mg, 0.06 mmol, 30%):

R_f 0.37 (amine) (1:10 MeOH:$CHCl_3$); **m.p.** 187-189 °C; **IR** $υ_{max}$ 3600 – 2000, 2719, 2608, 1626, 1572, 1481, 1460, 1319, 1209, 1171, 1123, 1069, 1021, 1006; **^{1}H NMR** (400 MHz, DMSO-d$_6$) δ 8.16 (s, 1H), 7.95 (d, *J* = 7.8 Hz, 1H), 7.82 – 7.76 (m, 2H), 7.55 – 7.49 (m, 2H), 7.44 (d, *J* = 7.8 Hz, 1H), 7.41 – 7.34 (m, 2H), 7.26 – 7.21 (m, 1H), 7.05 (d, *J* = 7.6 Hz, 1H), 4.43 (s, 2H), 3.71 (s br, 4H), 2.38 – 2.16 (m, 2H), 1.01 (t, *J* = 7.5 Hz, 3H); **^{13}C NMR** (126 MHz, DMSO-d$_6$) δ 150.3, 141.3, 140.4, 137.3, 133.8, 132.5, 132.1, 131.8, 129.2, 128.5, 128.1, 127.9, 127.5 (q, *J* = 29.1 Hz, 1C), 125.34, 125.0, 123.8 (q, *J* = 274.4 Hz, 1C), 114.0, 49.3, 43.6, 25.7, 23.7, 15.0; **HRMS** (ESI) calcd for $[C_{25}H_{24}N_3F_3 + H]^+$: 424.2000, found: 424.1999.

2-(1*H*-benzo[*d*]imidazol-2-yl)-*N*-((2'-ethyl-2-methoxy-[1,1'-biphenyl]-4-yl)methyl)ethan-1-amine HCl salt (33)

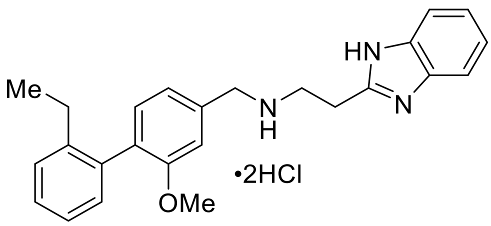

Prepared by general method **F** using **33b** (50 mg, 0.21 mmol), 2-(1*H*-benzimidazol-2-yl)ethylamine (64 mg, 0.40 mmol), DMF (2.0 mL) and sodium triacetoxyborohydride (132 mg, 0.62 mmol). The crude product was purified by flash column chromatography (silica gel, gradient elution: 0:100 MeOH:CH_2Cl_2 to 10:90 MeOH:CH_2Cl_2) and the resulting residue was then dissolved in CH_2Cl_2 (1.5 mL) and HCl (4M in 1,4-dioxane) (0.20 mL, 0.80 mmol) added dropwise. The reaction was stirred for 30 minutes before the mixture was concentrated *in vacuo* and triturated with Et_2O to provide the title compound **33** as a beige solid (33 mg, 0.07 mmol, 35%):

R*f* 0.26 (amine) (1:10 MeOH:$CHCl_3$); **m.p.** 198-200 °C; **IR** υ_{max} 3600 – 2000, 2715, 2611, 1625, 1575, 1514, 1483, 1461, 1419, 1389, 1276, 1224, 1174, 1035, 1005; **^{1}H NMR** (400 MHz, DMSO-d$_6$) δ 7.83 – 7.76 (m, 2H), 7.57 – 7.48 (m, 3H), 7.32 – 7.27 (m, 2H), 7.25 – 7.18 (m, 2H), 7.14 (d, *J* = 7.6 Hz, 1H), 7.02 (d, *J* = 7.4 Hz, 1H), 4.31 (s, 2H), 3.83 – 3.61 (m, 7H), 2.45 – 2.26 (m, 2H), 0.98 (t, *J* = 7.5 Hz, 3H); **^{13}C NMR** (126 MHz, DMSO-d$_6$) δ 156.4, 150.4, 142.0, 137.3, 132.5, 131.6, 131.0, 130.9, 130.7, 130.0, 128.0, 127.7, 127.6, 125.6, 125.5, 125.4, 121.9, 114.0, 113.0, 55.6, 50.1, 43.4, 25.7, 23.6, 15.2; **HRMS** (ESI) calcd for $[C_{25}H_{27}N_3O + H]^+$: 386.2232, found: 386.2235.

2-(1*H*-benzo[*d*]imidazol-2-yl)-*N*-((2'-ethyl-2-(trifluoromethoxy)-[1,1'-biphenyl]-4-yl)methyl)ethan-1-amine HCl salt (34)

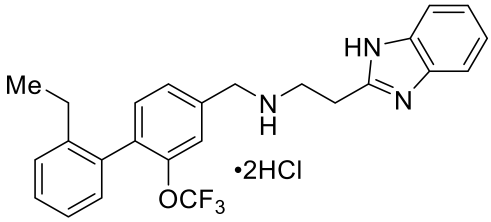

Prepared by general method **F** using **34b** (50 mg, 0.17 mmol), 2-(1*H*-benzimidazol-2-yl)ethylamine (55 mg, 0.34 mmol), DMF (2.0 mL) and sodium triacetoxyborohydride (108 mg, 0.51 mmol). The crude product was purified by flash column chromatography (silica gel, gradient elution: 0:100 MeOH:CH_2Cl_2 to 10:90 MeOH:CH_2Cl_2) and the resulting residue was then dissolved in CH_2Cl_2 (1.5 mL) and HCl (4M in 1,4-dioxane) (0.20 mL, 0.80 mmol) added dropwise. The reaction was stirred for 30 minutes before the mixture was concentrated *in vacuo* and triturated with Et_2O to provide the title compound **34** as a beige solid (37 mg, 0.07 mmol, 42%):

R*f* 0.26 (amine) (1:10 MeOH:$CHCl_3$); **m.p.** 176-178 °C; **IR** υ_{max} 3600 – 2000, 2724, 2616, 1630, 1575, 1519, 1484, 1462, 1387, 1246, 1209, 1169, 1021, 1007; **^{1}H NMR** (400 MHz, DMSO-d$_6$) δ 7.84 – 7.76 (m, 3H), 7.74 (d, *J* = 7.9 Hz, 1H), 7.55 – 7.50 (m, 2H), 7.47 (d, *J* = 7.9 Hz, 1H), 7.41 – 7.36 (m, 2H), 7.32 –

7.24 (m, 1H), 7.09 (d, *J* = 7.5 Hz, 1H), 4.40 (s, 2H), 3.78 – 3.65 (m, 4H), 2.45 – 2.27 (m, 2H), 1.01 (t, *J* = 7.6 Hz, 3H); **^{13}C NMR** (126 MHz, DMSO-d$_6$) δ 150.3, 145.6, 141.6, 135.0, 134.8, 133.6, 132.4, 131.6, 129.8, 129.2, 128.6, 128.3, 125.7, 125.4, 122.5, 120.0 (q, *J* = 257.5 Hz, 1C), 114.0, 49.2, 43.5, 25.5, 23.6, 15.1; **HRMS** (ESI) calcd for $[C_{25}H_{24}N_3OF_3 + H]^+$: 440.1949, found: 440.1951.

3-((*tert*-Butyloxycarbonyl)((2,6-dichloro-2'-ethyl-[1,1'-biphenyl]-4-yl)methyl)amino)propanoic acid (35e)

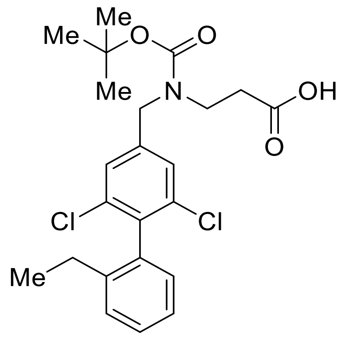

Prepared by general method **D** using **15a** (1.08 g, 3.34 mmol), 2-ethylphenylboronic acid (1.00 g, 6.68 mmol), $PdCl_2$(dppf) (273 mg, 0.33 mmol), K_2CO_3 (1.85 g, 13.4 mmol) and 1,4-dioxane (15.0 mL) and heating for 5 hours. The crude product was purified by flash column chromatography (silica gel, gradient elution: 0:100 EtOAc:pet ether to 5:95 EtOAc:pet ether) and the resulting residue was used in general method **F** with β-alanine methyl ester hydrochloride (700 mg, 5.02 mmol), NEt_3 (0.70 mL, 5.02 mmol), 1,2-dichloroethane (15.0 mL) and sodium triacetoxyborohydride (1.33 g, 6.28 mmol). The crude product was purified by flash column chromatography (silica gel, gradient elution: 0:100 MeOH:CH_2Cl_2 to 5:95 MeOH:CH_2Cl_2) and the resulting residue was then dissolved in CH_2Cl_2 (10 mL) and Boc2O (1.07 g, 4.90 mmol), NEt3 (0.68 mL, 4.90 mmol) and DMAP (30 mg, 0.25 mmol) were added. The reaction mixture was stirred at room temperature overnight before diluting with CH2Cl2 and washing with H2O, a saturated aqueous solution of NaHCO3 and a saturated aqueous solution of NaCl. The organic layer was dried over Na2SO4, filtered and concentrated *in vacuo*. The crude product was purified by flash column chromatography (silica gel, gradient elution: 0:100 EtOAc:pet ether to 15:85 EtOAc:pet ether) and the resulting residue was dissolved in THF (10 mL) and H_2O (10 mL). To the solution was added lithium hydroxide monohydrate (210 mg, 5.00 mmol) and the reaction mixture stirred at room temperature for 18 hours. The mixture was cooled to 0 °C, quenched with 1M aqueous HCl and extracted three times with CH_2Cl_2. The combined organic extracts were washed with a saturated aqueous solution of NaCl, dried over Na_2SO_4, filtered and concentrated *in vacuo* to provide the title compound **35e** as a colourless oil (1.08 g, 2.38 mmol, 71%; containing ~10% of by-products):

R_f 0.43 (1:10 MeOH:CH_2Cl_2); **IR** $υ_{max}$ 3600 – 2400, 2973, 2932, 1695, 1463, 1393, 1366, 1272, 1250, 1157, 1120; **^{1}H NMR** (400 MHz, $CDCl_3$) δ 7.42 – 7.31 (m, 3H), 7.31 – 7.22 (m, 2H), 7.04 (dd, *J* = 7.5, 0.8 Hz, 1H), 4.47 (s br, 2H), 3.55 (s br, 2H), 2.66 (s br, 2H), 2.36 (q, *J* = 7.6 Hz, 2H), 1.47 (s br, 9H), 1.09 (t, *J* = 7.6 Hz, 3H); **^{13}C NMR** (100 MHz, $CDCl_3$) δ 176.2, 155.6, 142.2, 138.0, 136.0, 135.4, 135.2, 129.4, 128.7, 128.2, 125.8, 81.0, 50.0, 43.0, 33.2, 28.4, 26.1, 14.5; **HRMS** (ESI) calcd for $[C_{23}H_{27}NO_4{}^{35}Cl_2 + Na]^+$: 474.1215, found: 474.1223.

***N*-((2,6-dichloro-2'-ethyl-[1,1'-biphenyl]-4-yl)methyl)-2-(7-methoxy-1*H*-benzo[*d*]imidazol-2-yl)ethan-1-amine HCl salt (35)**

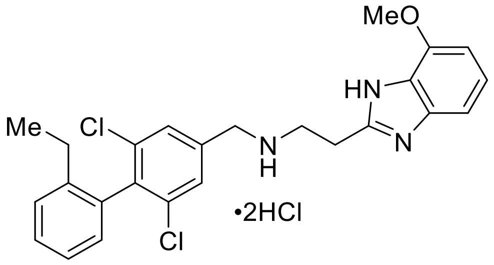

Prepared by general method **H** using **35e** (90 mg, 0.20 mmol), 3-methoxy-1,2-phenylenediamine (41 mg, 0.30 mmol), HBTU (114 mg, 0.30 mmol) and NEt_3 (0.08 mL, 0.60 mmol) in DMF (2.0 mL). The crude product was first purified by flash column chromatography (silica gel, gradient elution: 20:80 EtOAc:pet ether to 75:25 EtOAc:pet ether) before dissolving in acetic acid (3.0 mL) and heating to 65 °C overnight. The boc-protected amine of **35** was recovered by flash column chromatography (silica gel, gradient elution: 20:80 EtOAc:pet ether to 75:25 EtOAc:pet ether) as a colourless oil (66 mg, 0.12 mmol, 60%) and then deprotected by general method **J** using amine (29 mg, 0.05 mmol) and HCl (4M in 1,4-dioxane) (1.0 mL, 4.0 mmol) to provide the title compound **35** as an off-white solid (21 mg, 0.04 mmol, 81%):

R_f 0.47 (amine) (1:10 MeOH:CH_2Cl_2); **m.p.** 149-151 °C; **IR** $υ_{max}$ 3600 – 2000, 2729, 2598, 1628, 1574, 1544, 1493, 1441, 1272, 1221, 1107; **^{1}H NMR** (400 MHz, DMSO-d_6) δ 7.91 (s, 2H), 7.45 – 7.38 (m, 3H), 7.35 – 7.29 (m, 2H), 7.07 (d, *J* = 8.0 Hz, 1H), 7.02 (d, *J* = 7.5 Hz, 1H), 4.33 (s, 2H), 4.01 (s, 3H), 3.68 – 3.58 (m, 4H), 2.29 (q, J = 7.6 Hz, 2H), 1.02 (t, *J* = 7.6 Hz, 3H); **^{13}C NMR** (100 MHz, DMSO-d_6) δ 149.6, 147.1, 141.3, 138.9, 135.2, 134.5, 134.3, 133.4, 129.9, 129.0, 128.6, 126.3, 126.1, 106.1, 56.1, 48.6, 43.6, 25.7, 23.6, 14.7; **HRMS** (ESI) calcd for $[C_{25}H_{25}N_3O^{35}Cl_2 + H]^+$: 454.1453, found: 454.1460.

***N*-((2,6-dichloro-2'-ethyl-[1,1'-biphenyl]-4-yl)methyl)-2-(6-methoxy-1*H*-benzo[*d*]imidazol-2-yl)ethan-1-amine HCl salt (36)**

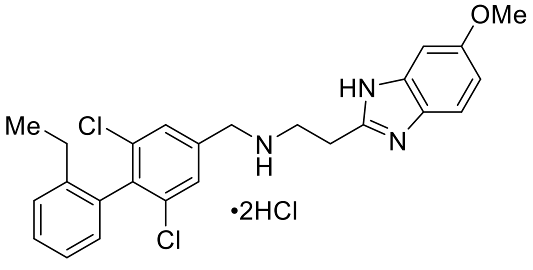

Prepared by general method **H** using **35e** (90 mg, 0.20 mmol), 4-methoxy-1,2-phenylenediamine hydrochloride (63 mg, 0.30 mmol), HBTU (114 mg, 0.30 mmol) and NEt_3 (0.14 mL, 1.00 mmol) in DMF (2.0 mL). The crude product was first purified by flash column chromatography (silica gel, gradient elution: 25:75 EtOAc:pet ether to 75:25 EtOAc:pet ether) before dissolving in acetic acid (3.0 mL) and heating to 65 °C overnight. The boc-protected amine of **36** was recovered by flash column chromatography (silica gel, gradient elution: 25:75 EtOAc:pet ether to 75:25 EtOAc:pet ether) and

then deprotected by general method **J** using HCl (4M in 1,4-dioxane) (1.0 mL, 4.0 mmol) to provide the title compound **36** as an off-white solid (30 mg, 0.06 mmol, 30%):

R*f* 0.47 (amine) (1:10 MeOH:CH_2Cl_2); **m.p.** 194-196 °C; **IR** υ_{max} 3600 – 2000, 2704, 1629, 1574, 1543, 1520, 1496, 1457, 1439, 1270, 1225, 1201, 1160, 1117, 1028; **^{1}H NMR** (400 MHz, DMSO-d_6) δ 7.92 (s, 2H), 7.68 (d, *J* = 8.9 Hz, 1H), 7.43 – 7.39 (m, 2H), 7.35 – 7.29 (m, 1H), 7.23 (d, *J* = 2.2 Hz, 1H), 7.13 (dd, *J* = 8.9, 2.2 Hz, 1H), 7.02 (d, *J* = 7.5 Hz, 1H), 4.35 (s, 2H), 3.86 (s, 3H), 3.72 – 3.61 (m, 4H), 2.30 (q, J = 7.6 Hz, 2H), 1.03 (t, *J* = 7.6 Hz, 3H); **^{13}C NMR** (126 MHz, DMSO-d_6) δ 157.7, 149.4, 141.3, 138.9, 135.2, 134.5, 134.3, 134.2, 129.9, 129.0, 128.6, 126.3, 115.0, 114.8, 96.4, 56.0, 48.6, 43.5, 25.7, 23.6, 14.7; **HRMS** (ESI) calcd for $[C_{25}H_{25}N_3O^{35}Cl_2 + H]^+$: 454.1453, found: 454.1455.

COMPOUND SPECTRA

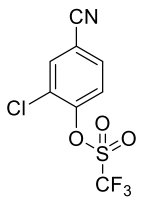

1a

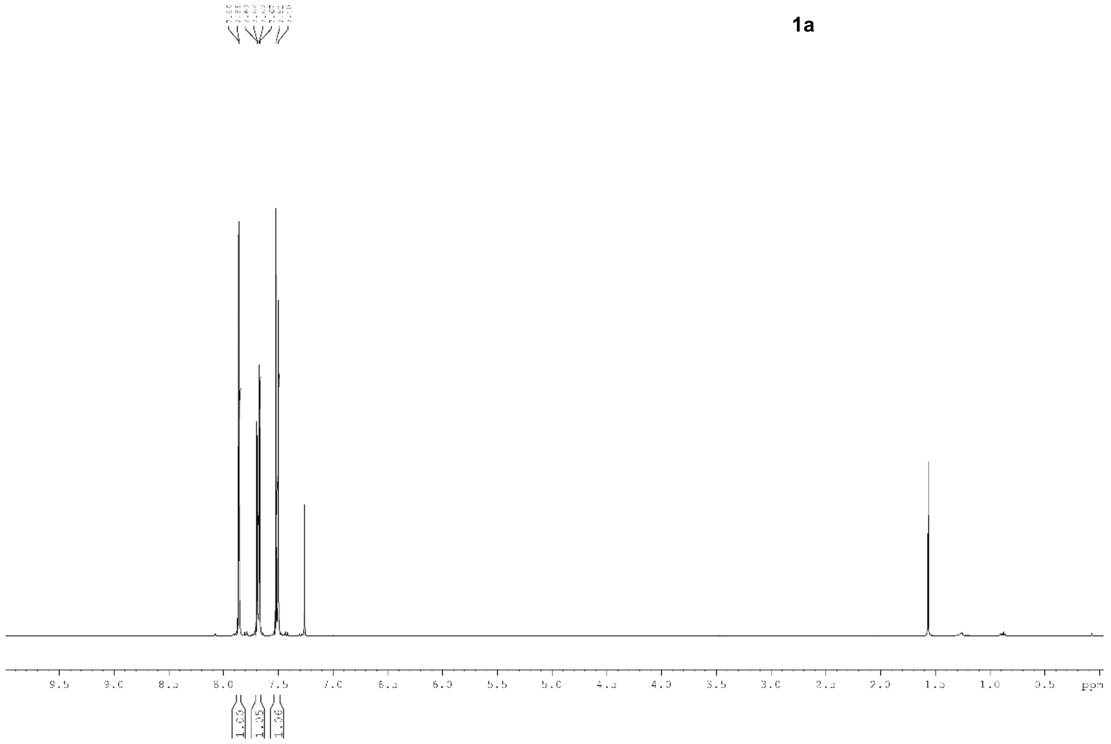

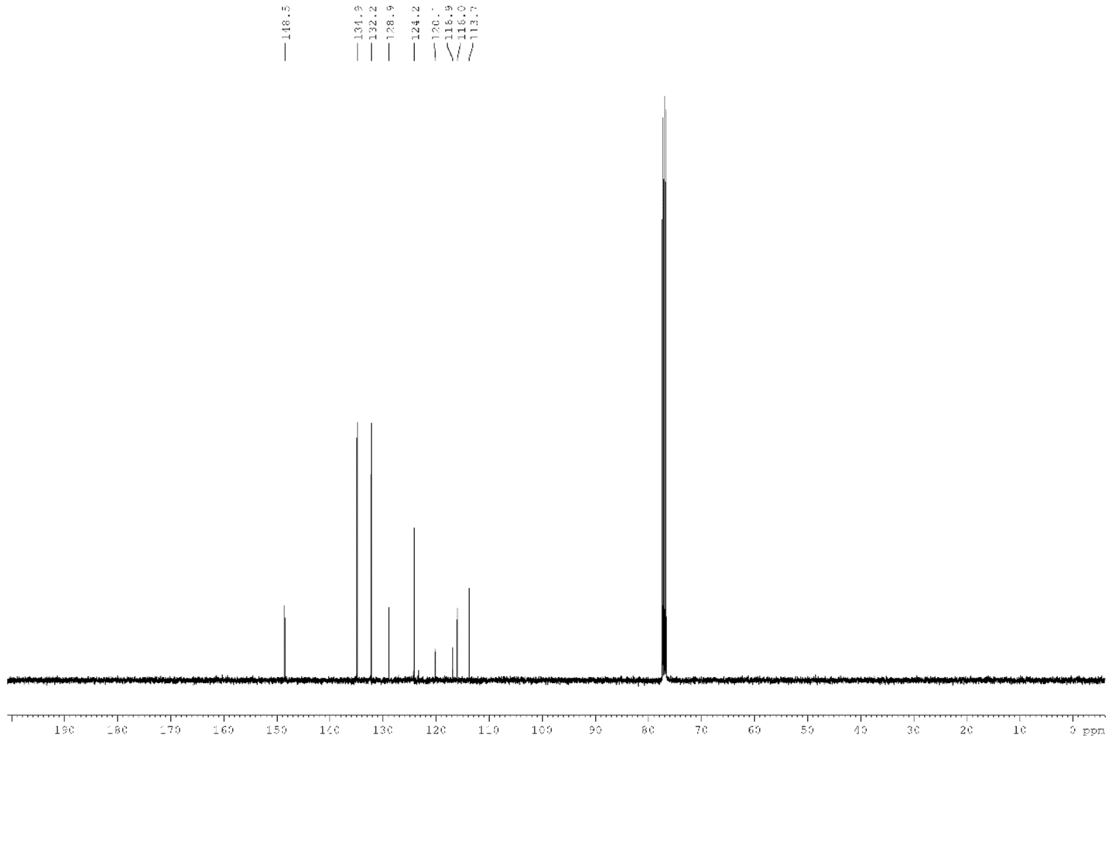

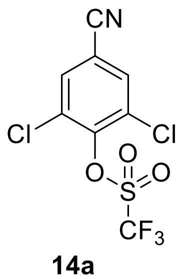

14a

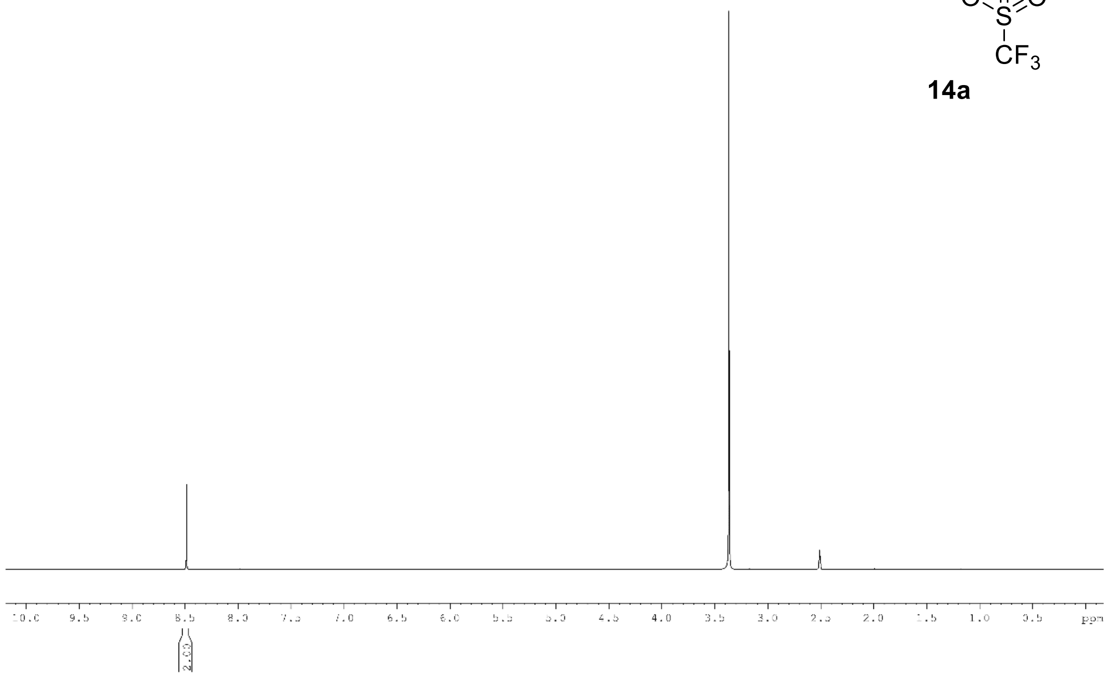

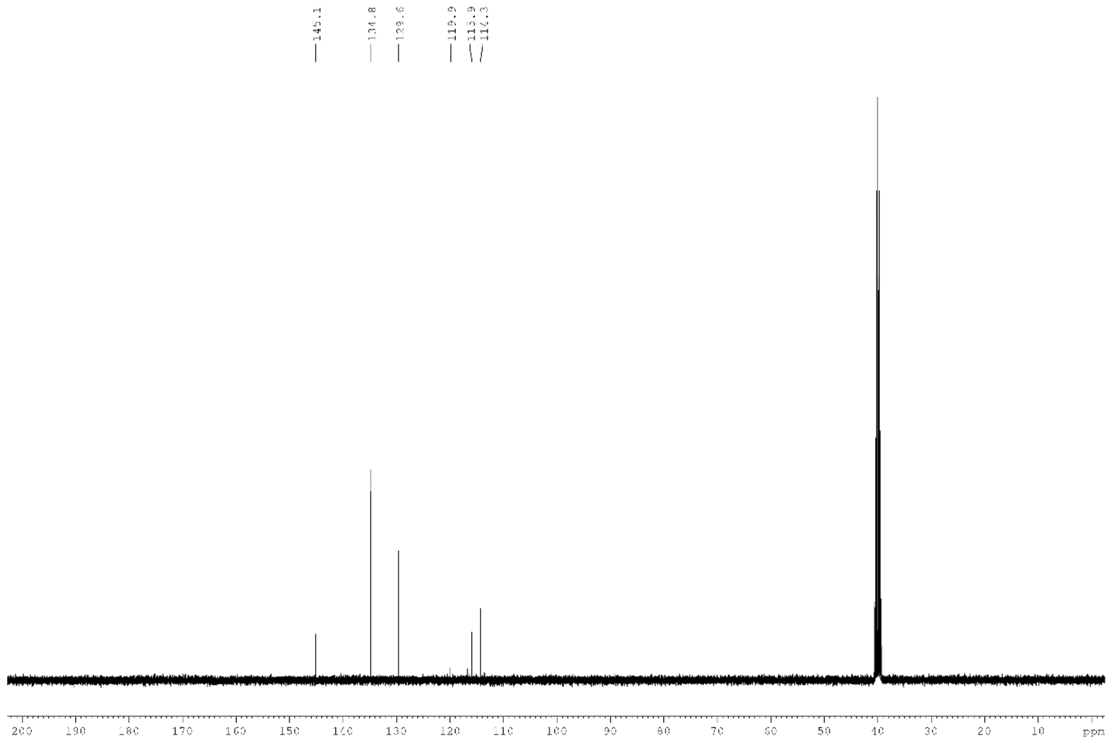

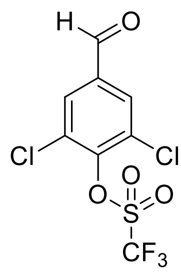

15a

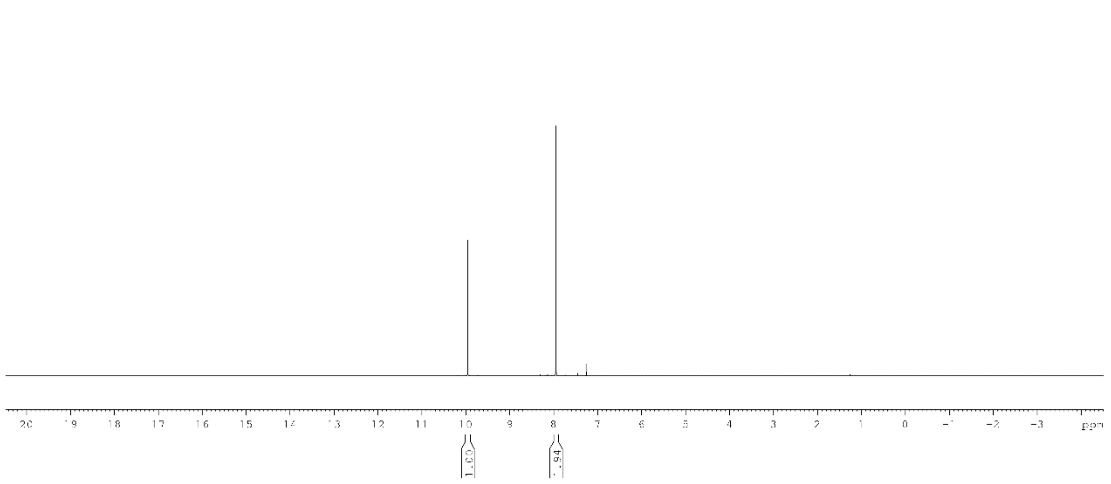

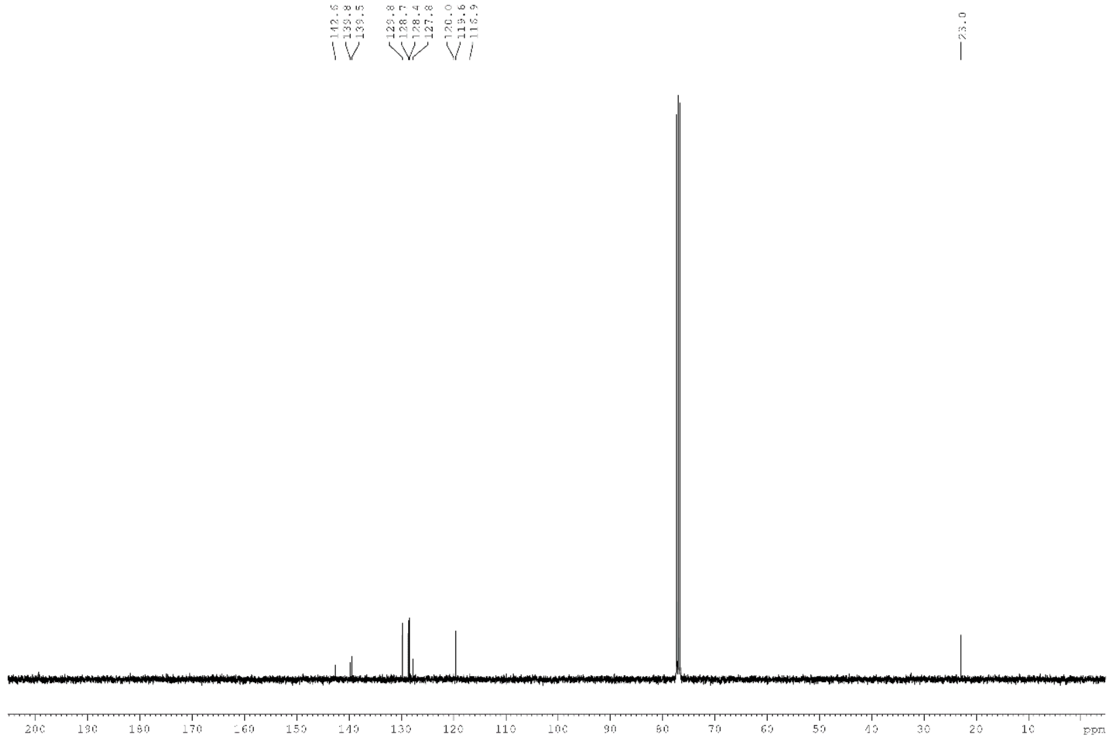

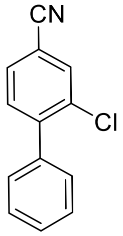
CN
Cl

1b

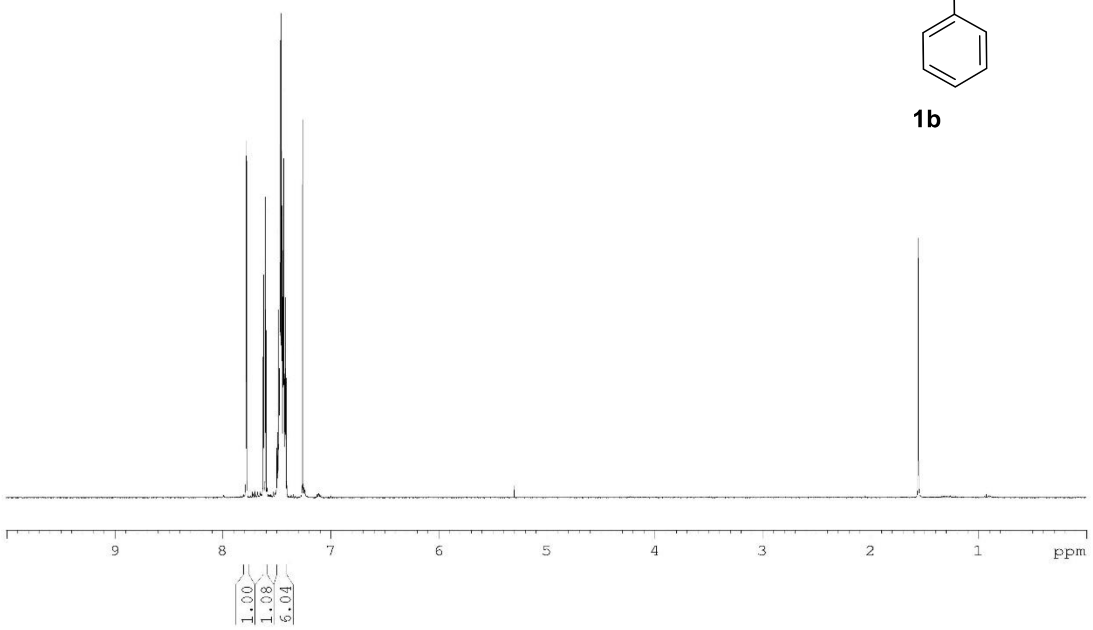
7.78
7.78
7.62
7.62
7.60
7.60
7.48
7.48
7.47
7.47
7.47
7.46
7.46
7.46
7.46
7.45
7.45
7.45
7.45
7.44
7.44
7.44
7.43
7.42
7.42
7.42
7.42
7.41
9 8 7 6 5 4 3 2 1 ppm
1.00
1.08
6.04

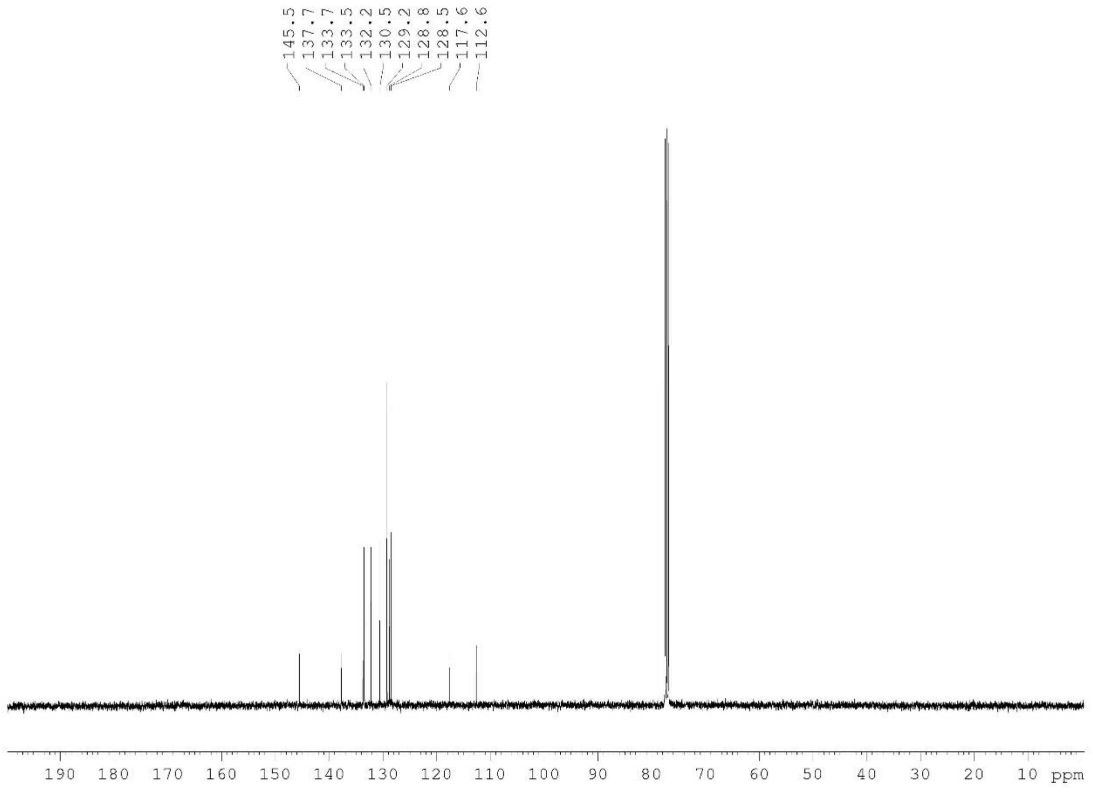
145.5
137.7
133.7
133.5
132.2
130.5
129.2
128.8
128.5
117.6
112.6
190 180 170 160 150 140 130 120 110 100 90 80 70 60 50 40 30 20 10 ppm

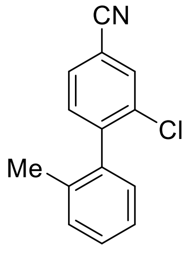
CN
Cl
Me

2b

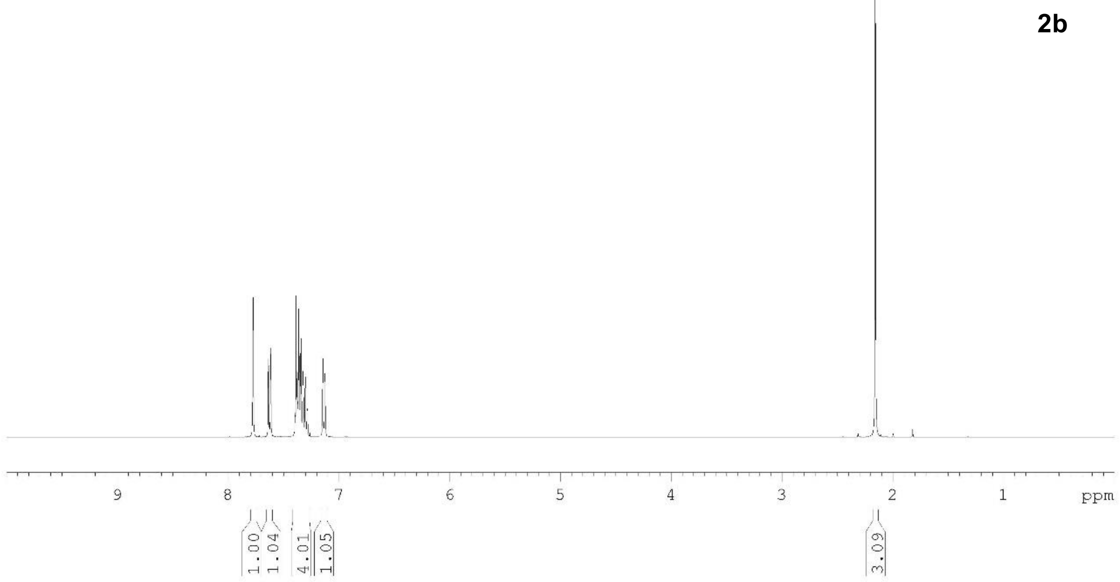
7.78 7.77 7.64 7.63 7.62 7.61 7.40 7.39 7.38 7.38 7.36 7.36 7.36 7.34 7.32 7.30 7.29 7.28 7.14 7.13
2.16
9 8 7 6 5 4 3 2 1 ppm
1.00 1.04 4.01 1.05
3.09

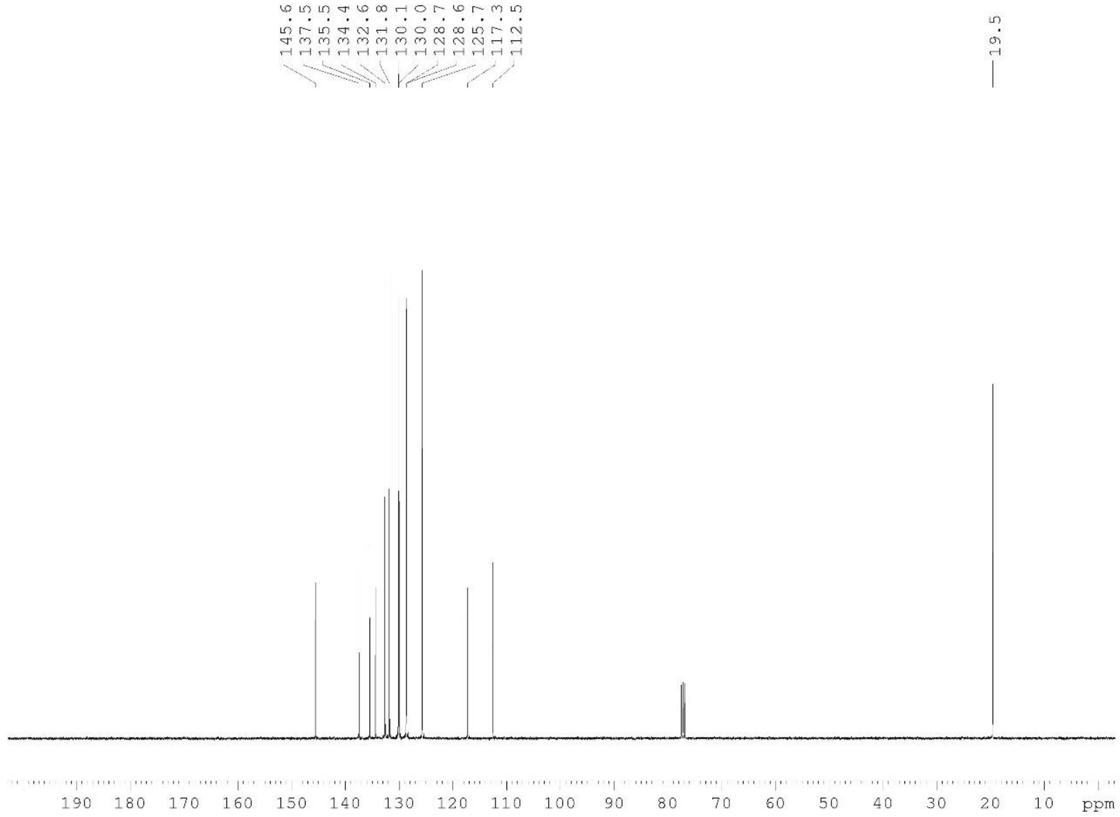
145.6 137.5 135.5 134.4 132.6 131.8 130.1 130.0 128.7 128.6 125.7 117.3 112.5
19.5
190 180 170 160 150 140 130 120 110 100 90 80 70 60 50 40 30 20 10 ppm

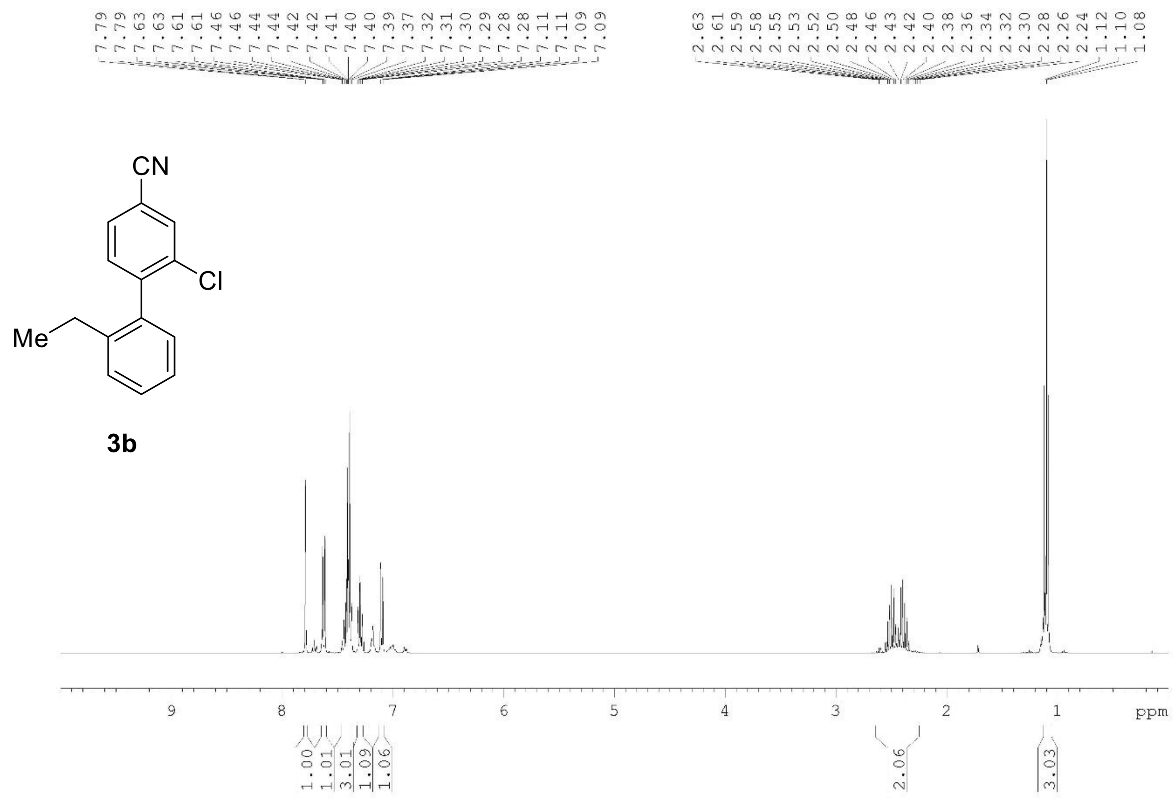
CN
Cl
Me
3b
7.79 7.79 7.63 7.61 7.61 7.46 7.44 7.44 7.42 7.42 7.41 7.40 7.40 7.39 7.37 7.32 7.31 7.30 7.29 7.28 7.28 7.11 7.11 7.09
2.63 2.61 2.59 2.58 2.55 2.53 2.52 2.50 2.48 2.46 2.43 2.42 2.40 2.38 2.36 2.34 2.32 2.30 2.28 2.26 2.24 1.12 1.10 1.08
9 8 7 6 5 4 3 2 1 ppm
1.00 1.01 3.01 1.09 1.06
2.06
3.03

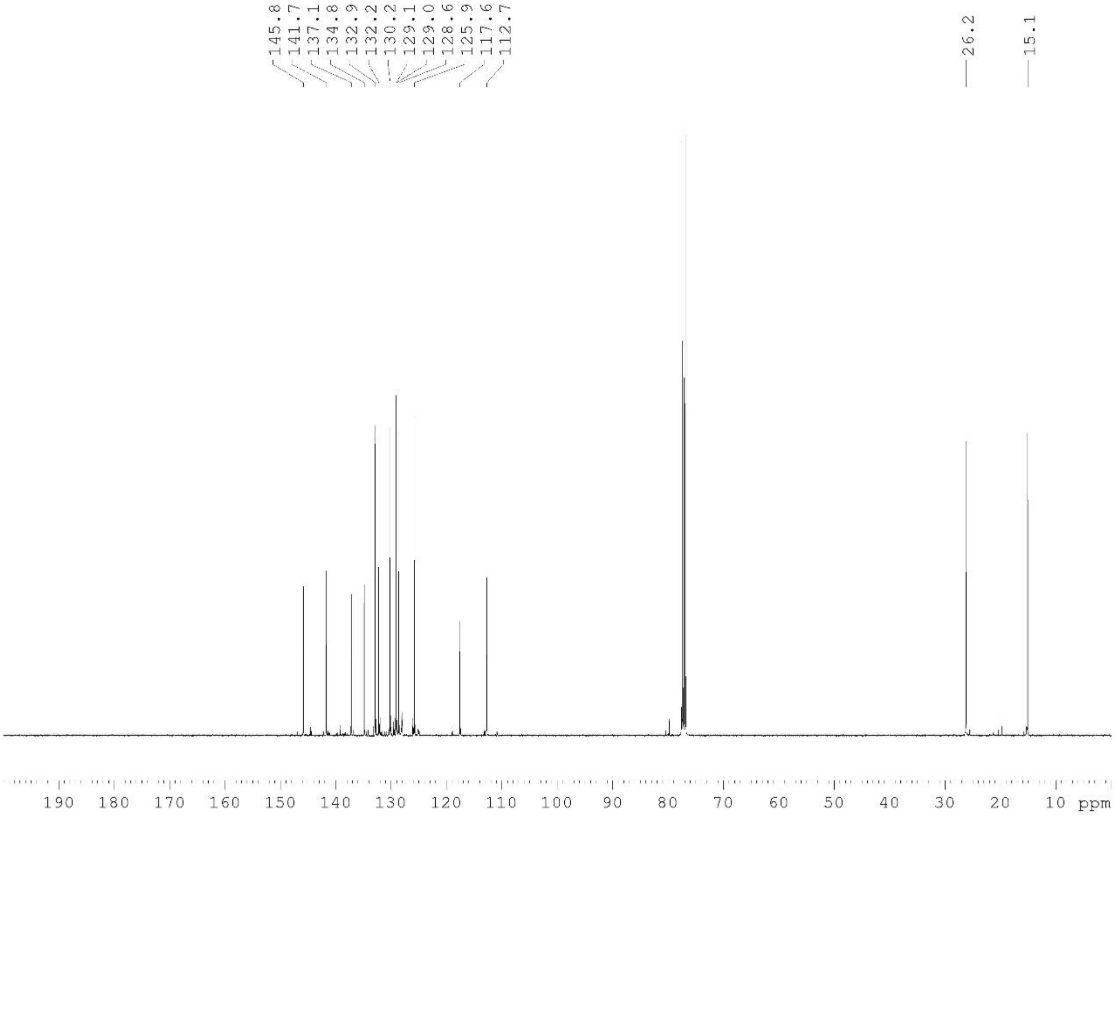
145.8 141.7 137.1 134.8 132.9 132.2 130.2 129.1 129.0 128.6 125.9 117.6 112.7
26.2
15.1
190 180 170 160 150 140 130 120 110 100 90 80 70 60 50 40 30 20 10 ppm

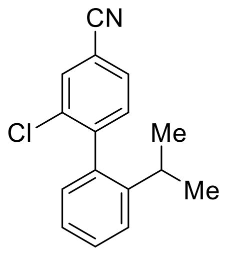
CN
Cl
Me
Me

4b

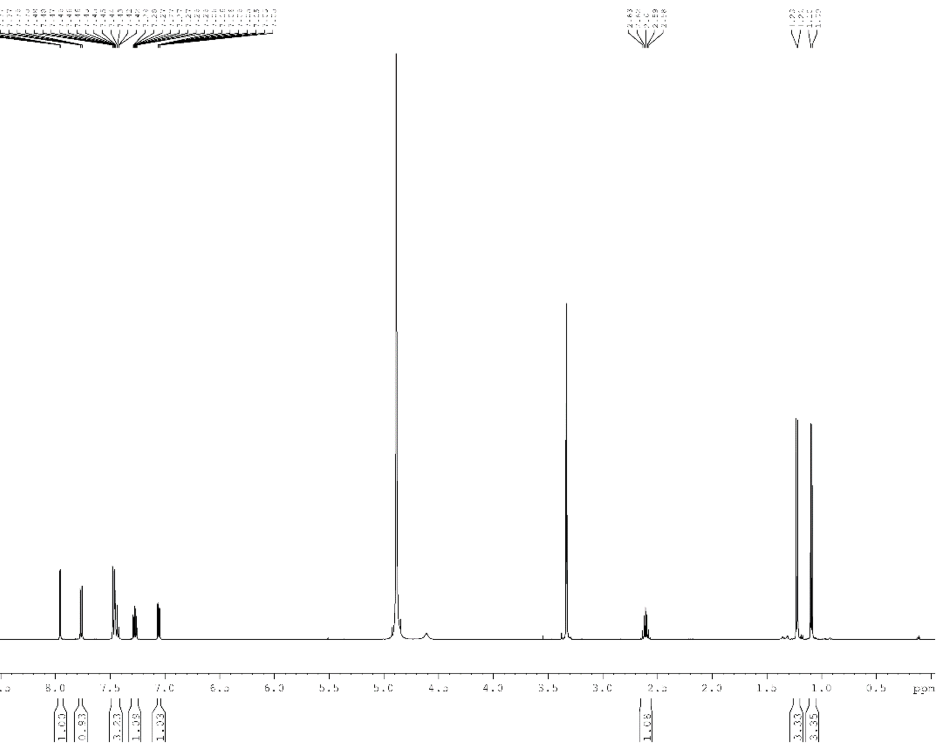

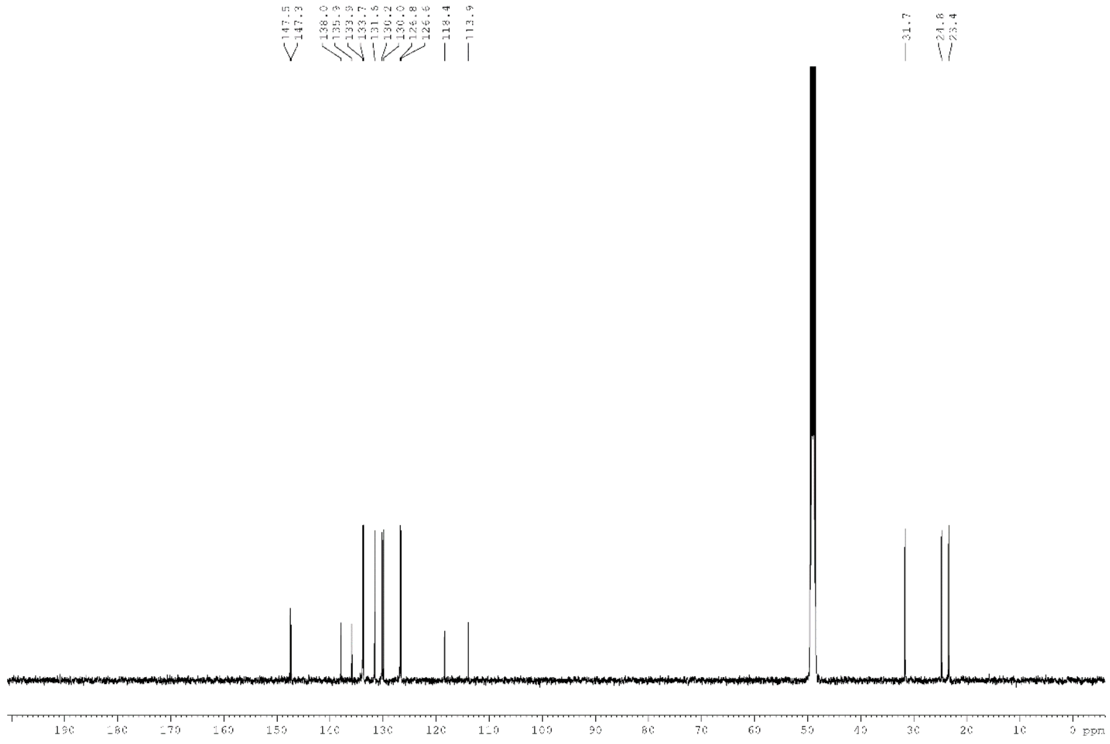
147.5
147.3
138.0
135.9
133.9
133.7
131.6
130.2
130.0
126.8
126.6
118.4
113.9
31.7
24.8
23.4

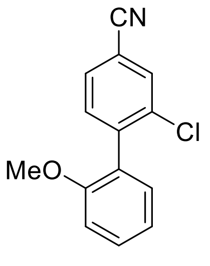
CN
Cl
MeO

5b

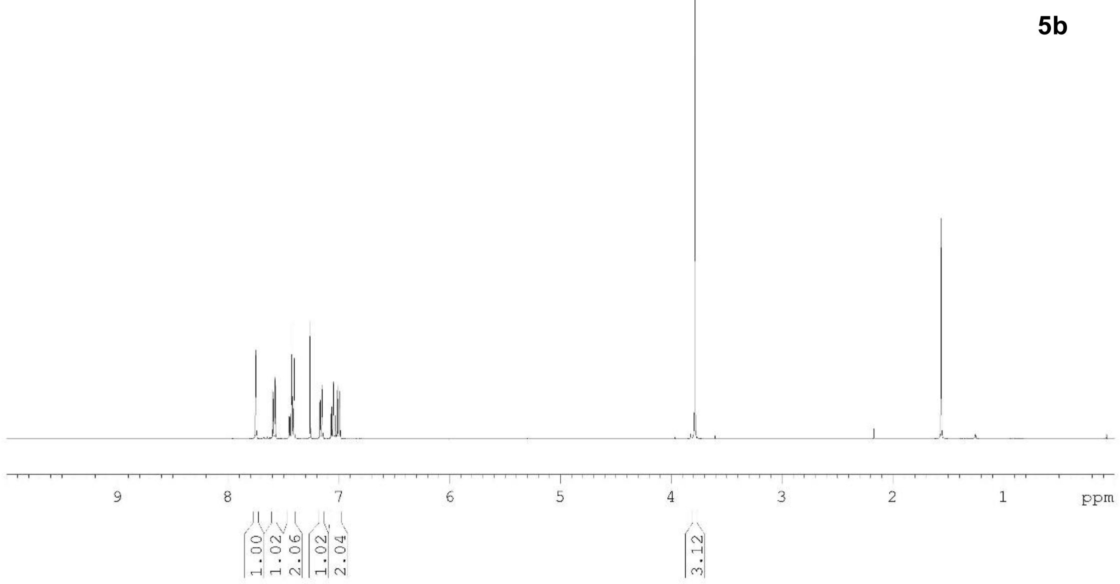
7.75 7.75 7.60 7.59 7.58 7.57 7.45 7.44 7.43 7.43 7.42 7.41 7.40 7.17 7.17 7.15 7.15 7.07 7.07 7.05 7.05 7.03 7.03 7.01 6.99
3.79
9 8 7 6 5 4 3 2 1 ppm
1.00 1.02 2.06 1.02 2.04
3.12

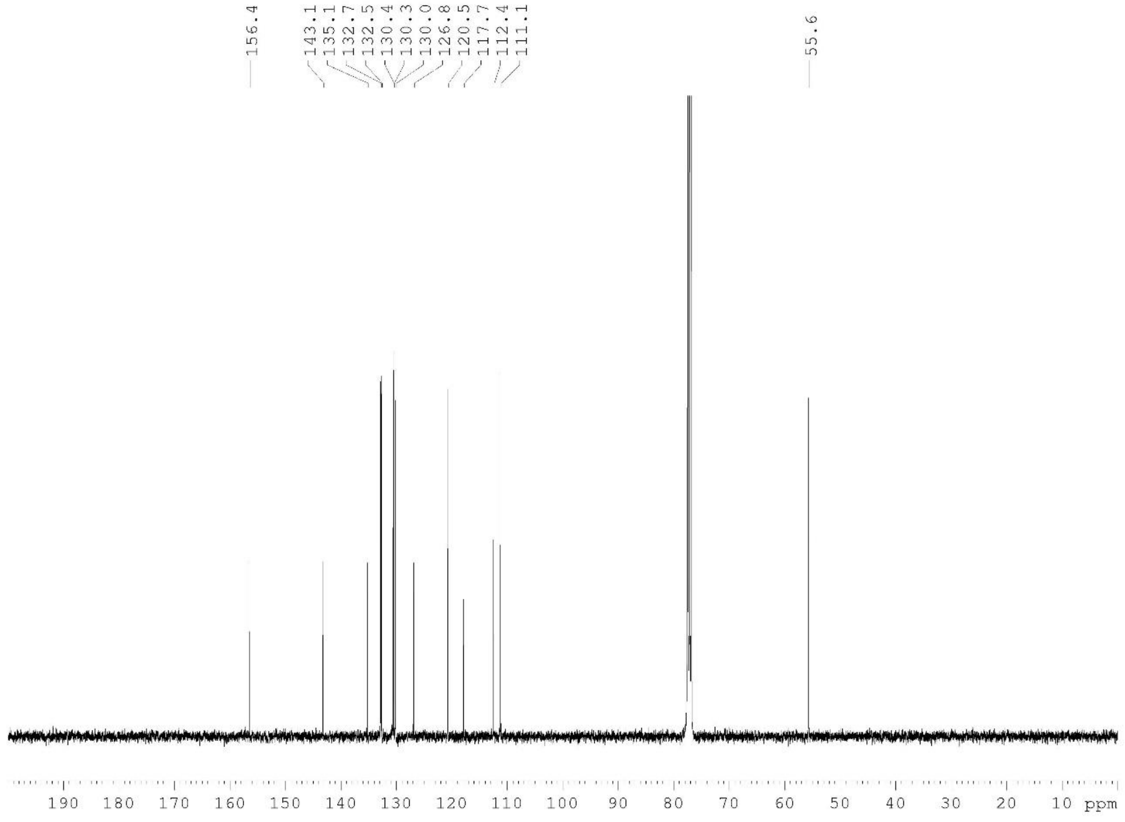
156.4
143.1 135.1 132.7 132.5 130.4 130.3 130.0 126.8 120.5 117.7 112.4 111.1
55.6
190 180 170 160 150 140 130 120 110 100 90 80 70 60 50 40 30 20 10 ppm

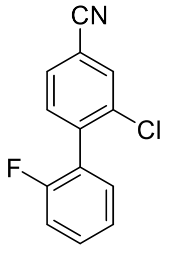
CN
Cl
F

6b

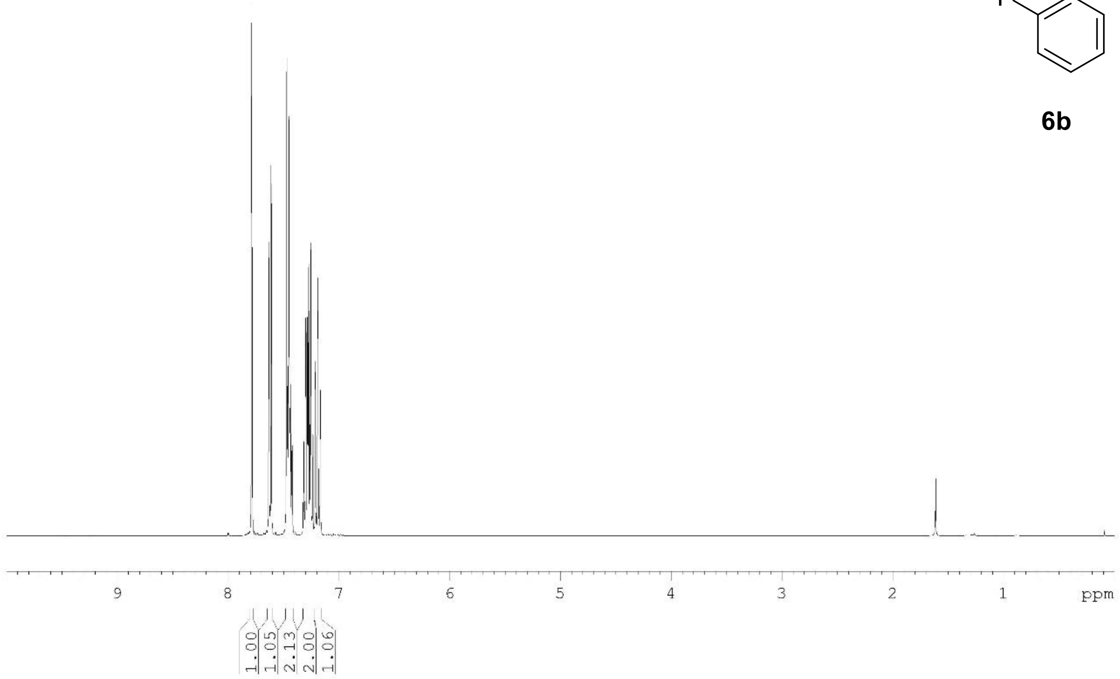
7.61
7.61
7.48
7.47
7.46
7.46
7.45
7.44
7.44
7.44
7.43
7.43
7.42
7.32
7.31
7.30
7.30
7.28
7.28
7.27
7.27
7.25
7.25
7.24
7.23
7.21
7.21
7.19
7.19
7.17
7.17
9
8
7
6
5
4
3
2
1
ppm
1.00
1.05
2.13
2.00
1.06

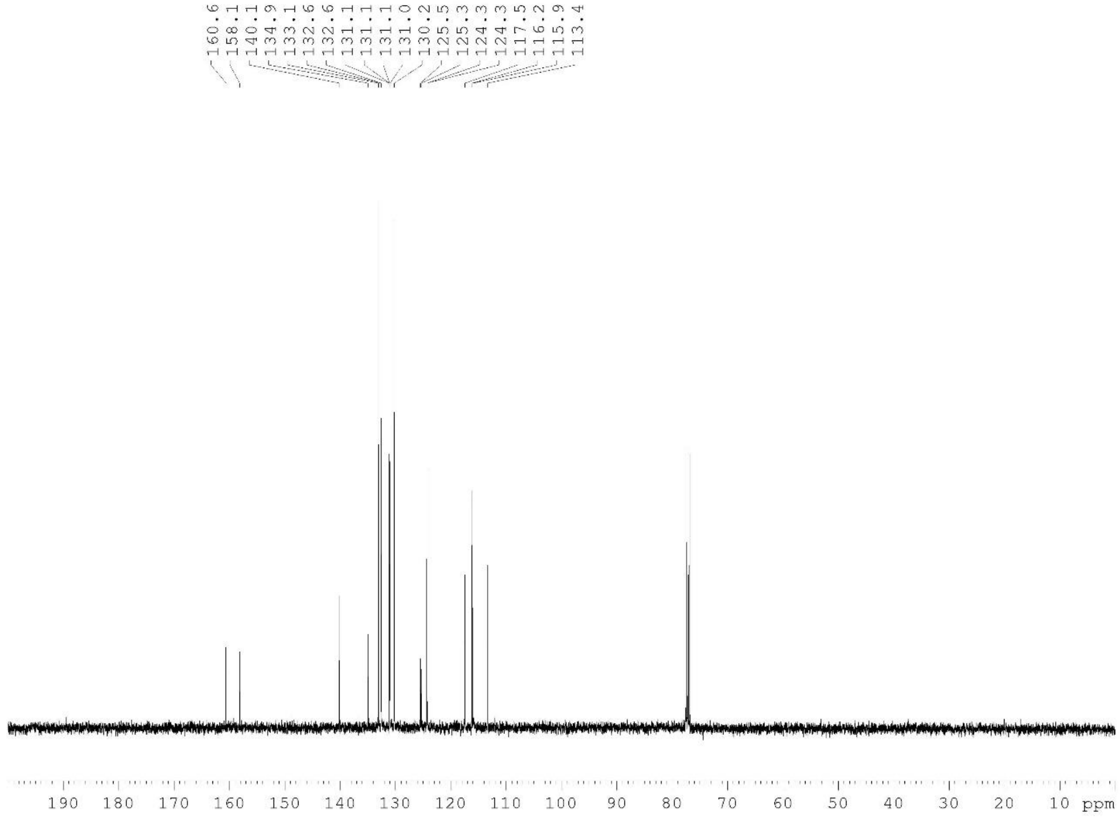
160.6
158.1
140.1
134.9
133.1
132.6
131.1
131.1
131.0
130.2
125.5
125.3
124.3
124.3
117.5
116.2
115.9
113.4
190
180
170
160
150
140
130
120
110
100
90
80
70
60
50
40
30
20
10
ppm

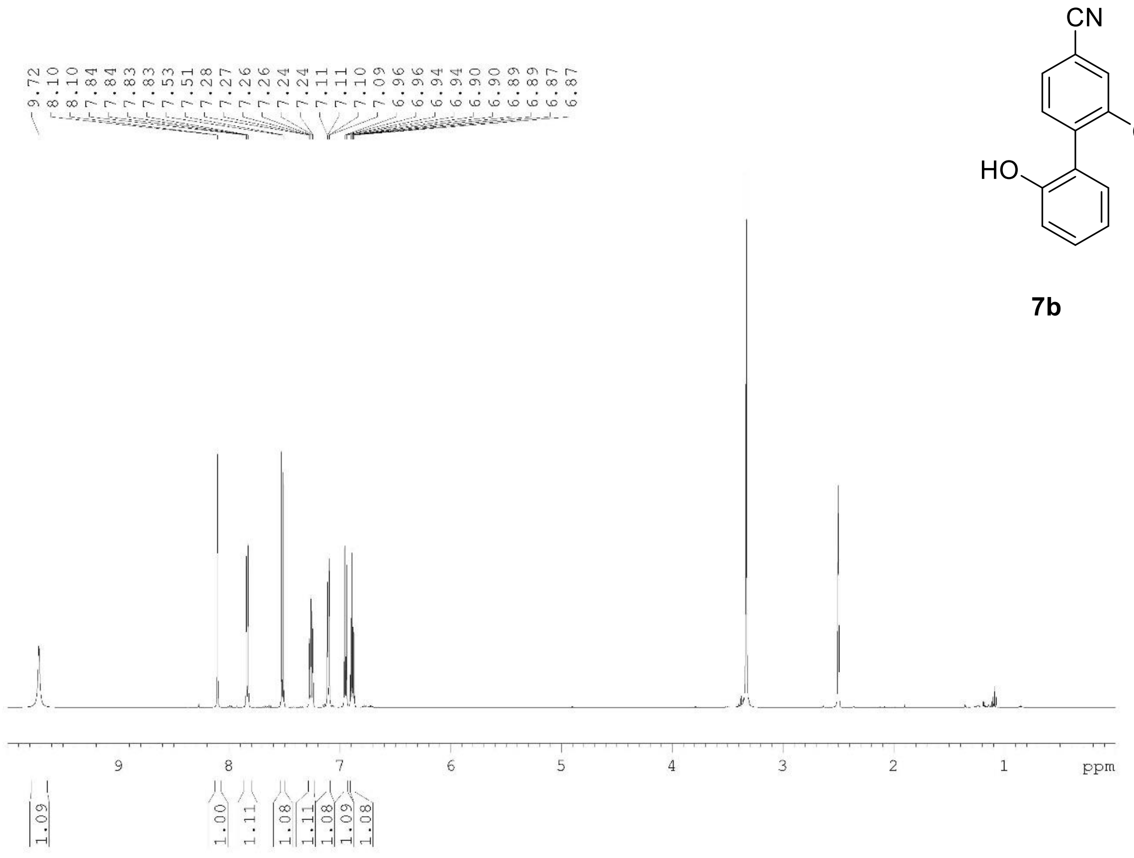

CN
Cl
HO
7b
9.72
8.10
8.10
7.84
7.84
7.83
7.83
7.53
7.51
7.28
7.27
7.26
7.26
7.24
7.24
7.11
7.11
7.10
7.09
6.96
6.96
6.94
6.94
6.90
6.90
6.89
6.89
6.87
6.87
9 8 7 6 5 4 3 2 1 ppm
1.09
1.00
1.11
1.08
1.11
1.08
1.09
1.08

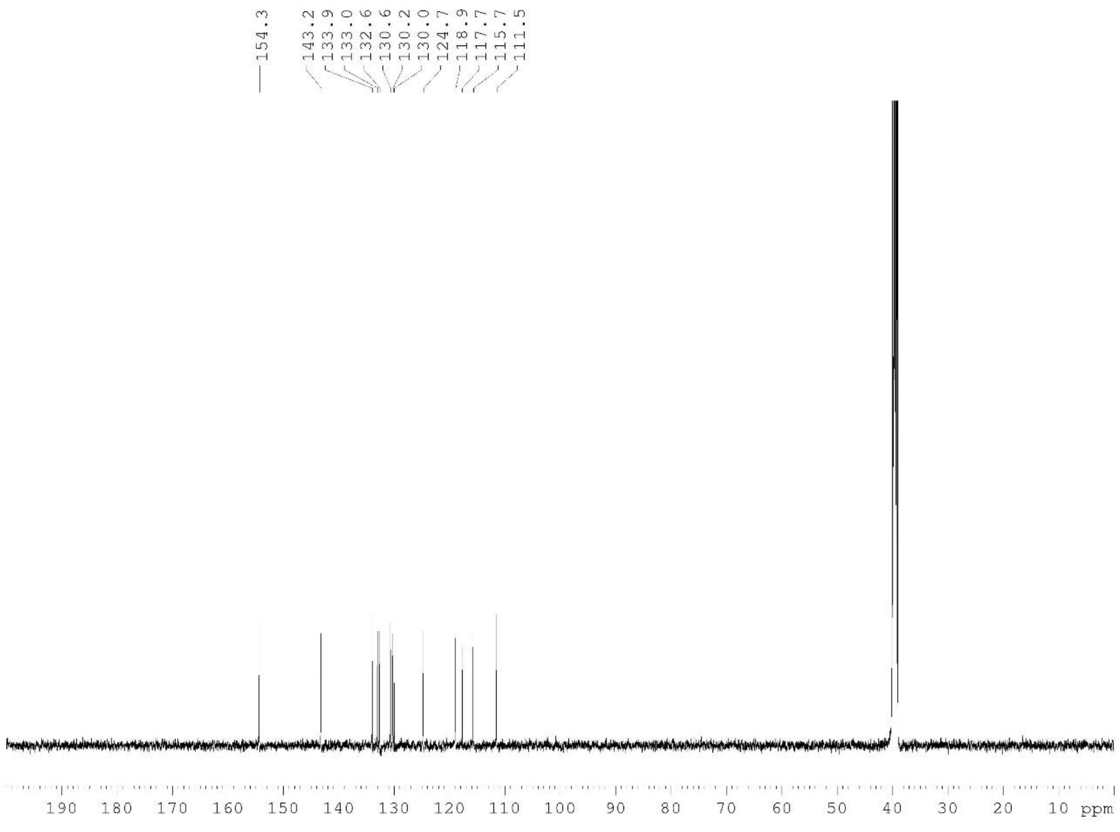

154.3
143.2
133.9
133.0
132.6
130.6
130.2
130.0
124.7
118.9
117.7
115.7
111.5
190 180 170 160 150 140 130 120 110 100 90 80 70 60 50 40 30 20 10 ppm

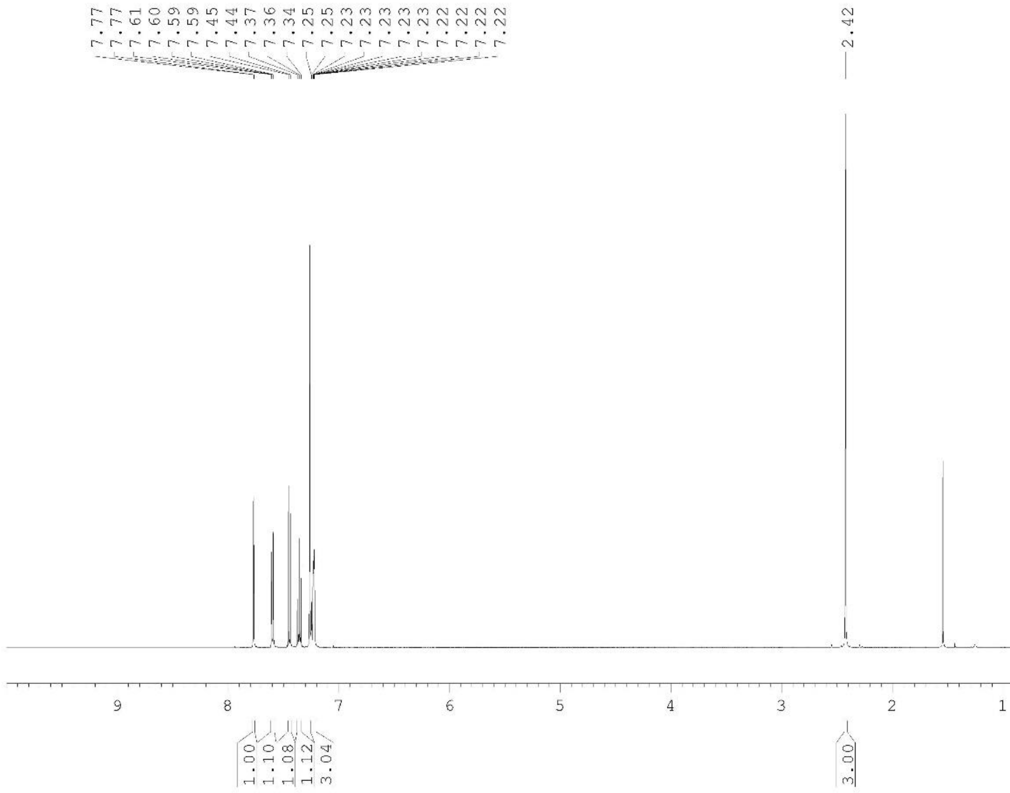
7.77
7.77
7.61
7.60
7.59
7.59
7.45
7.44
7.37
7.36
7.34
7.25
7.25
7.23
7.23
7.23
7.23
7.23
7.22
7.22
7.22
7.22
2.42
9 8 7 6 5 4 3 2 1 ppm
1.00
1.10
1.08
1.12
3.04
3.00

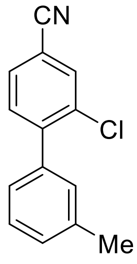
CN
Cl
Me

8b

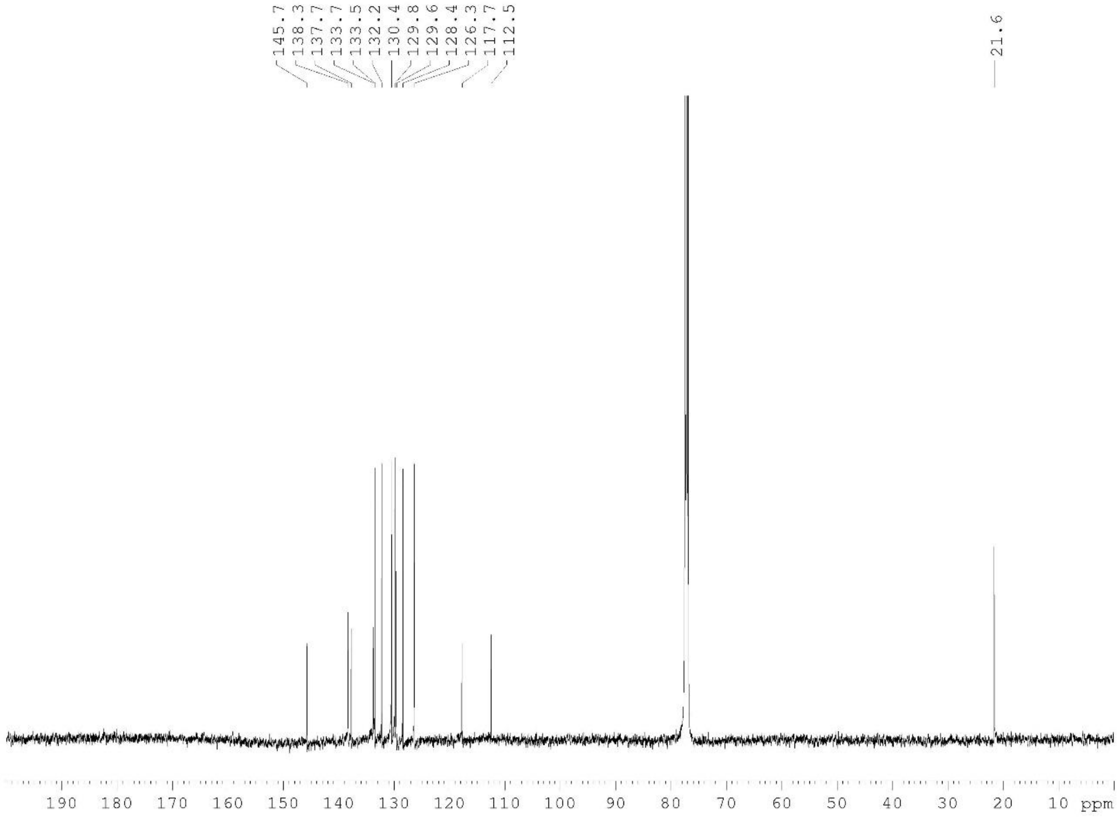
145.7
138.3
137.7
133.7
133.5
132.2
130.4
129.8
129.6
128.4
126.3
117.7
112.5
21.6
190 180 170 160 150 140 130 120 110 100 90 80 70 60 50 40 30 20 10 ppm

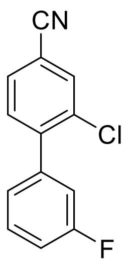

CN
Cl
F

9b

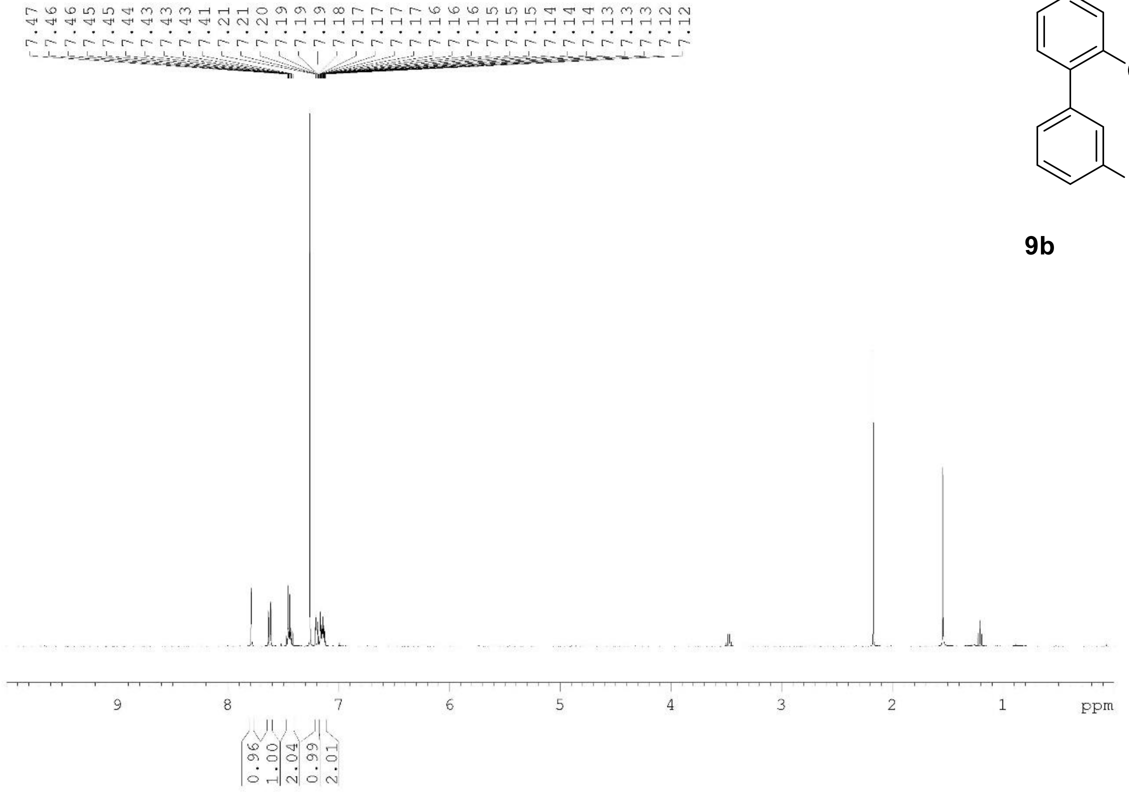

7.47
7.46
7.46
7.45
7.45
7.44
7.43
7.43
7.43
7.41
7.21
7.21
7.20
7.19
7.19
7.19
7.18
7.17
7.17
7.17
7.17
7.16
7.16
7.16
7.15
7.15
7.15
7.14
7.14
7.14
7.13
7.13
7.13
7.12
7.12
9
8
7
6
5
4
3
2
1
ppm
0.96
1.00
2.04
0.99
2.01

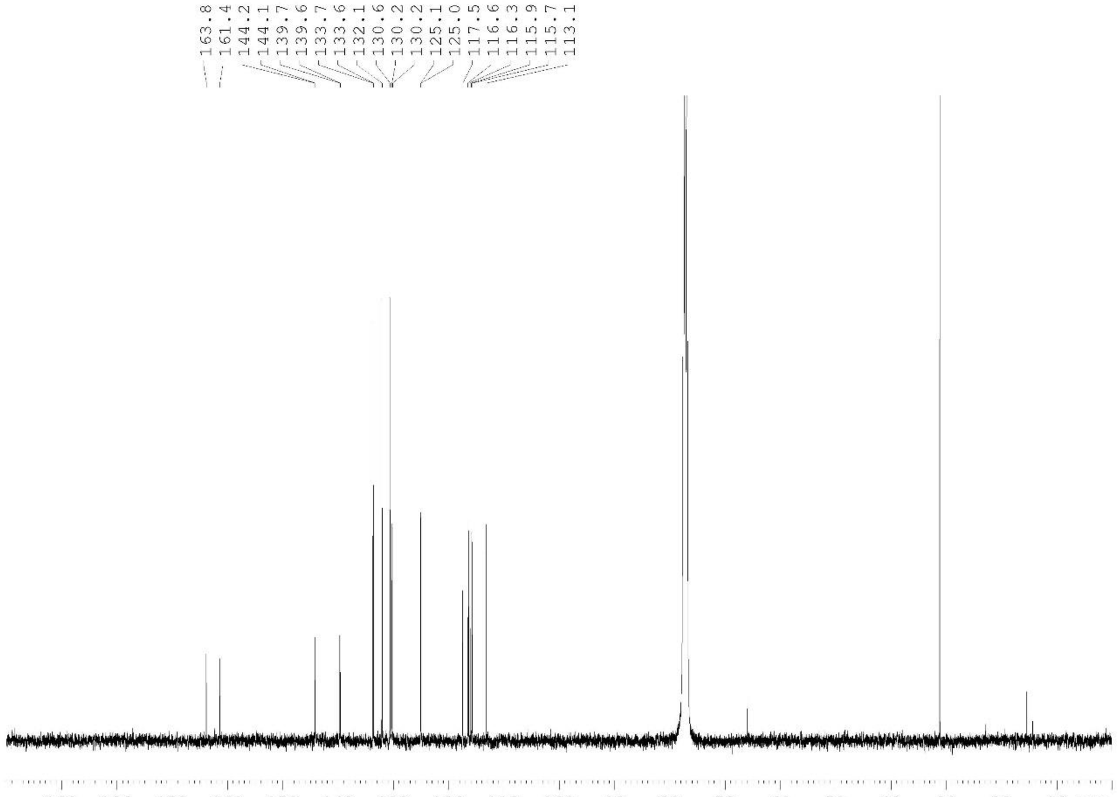

163.8
161.4
144.2
144.1
139.7
139.6
133.7
133.6
132.1
130.6
130.2
130.2
125.1
125.0
117.5
116.6
116.3
115.9
115.7
113.1
190
180
170
160
150
140
130
120
110
100
90
80
70
60
50
40
30
20
10 ppm

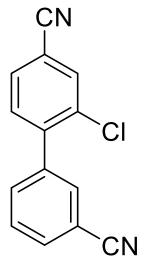
CN
Cl
CN

10b

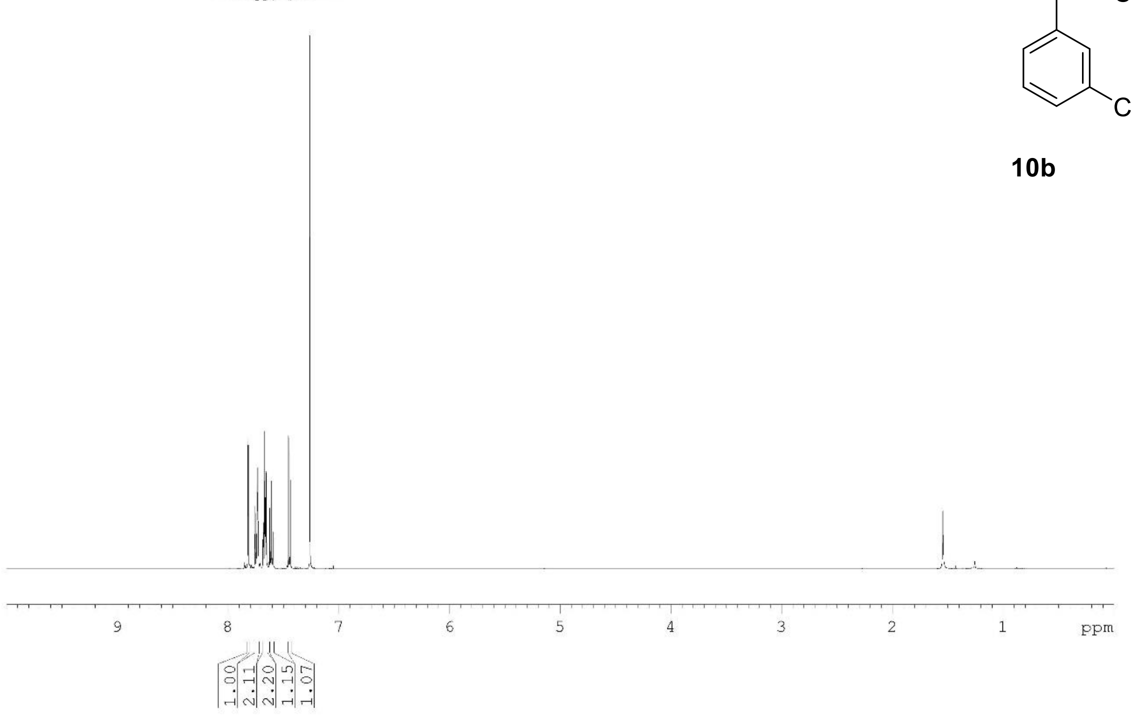
7.75
7.75
7.74
7.74
7.74
7.73
7.73
7.73
7.73
7.73
7.68
7.68
7.67
7.67
7.66
7.66
7.65
7.65
7.62
7.62
7.60
7.60
7.59
7.59
7.45
7.44
9
8
7
6
5
4
3
2
1
ppm
1.00
2.11
2.20
1.15
1.07

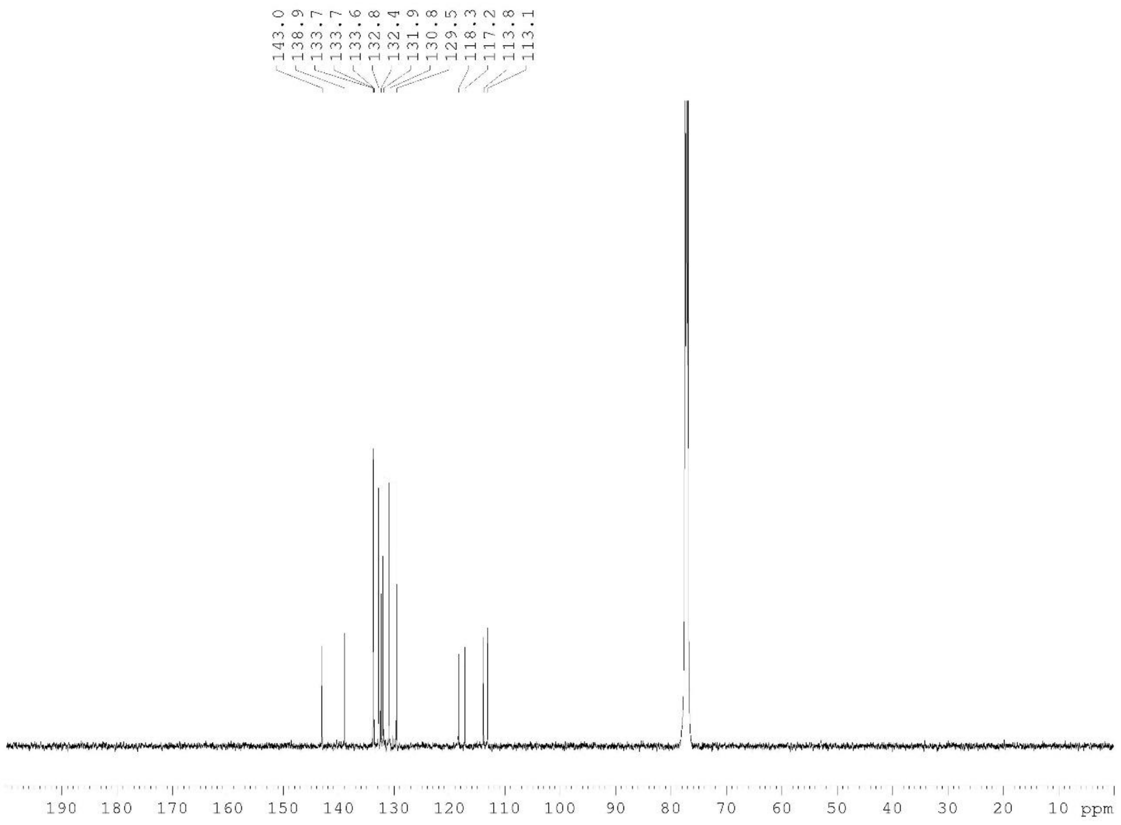
143.0
138.9
133.7
133.6
132.8
132.4
131.9
130.8
129.5
118.3
117.2
113.8
113.1
190
180
170
160
150
140
130
120
110
100
90
80
70
60
50
40
30
20
10 ppm

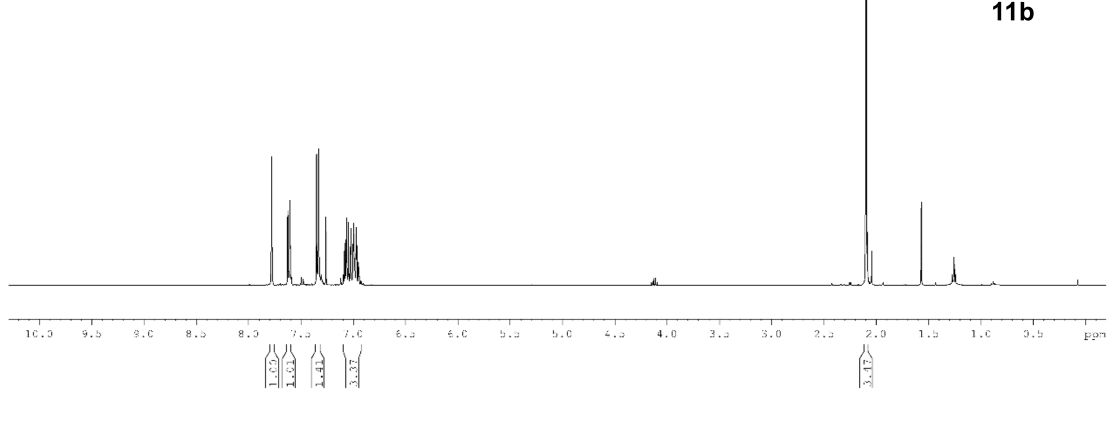

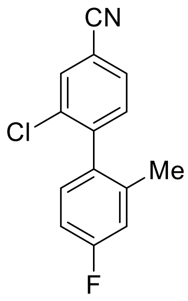

11b

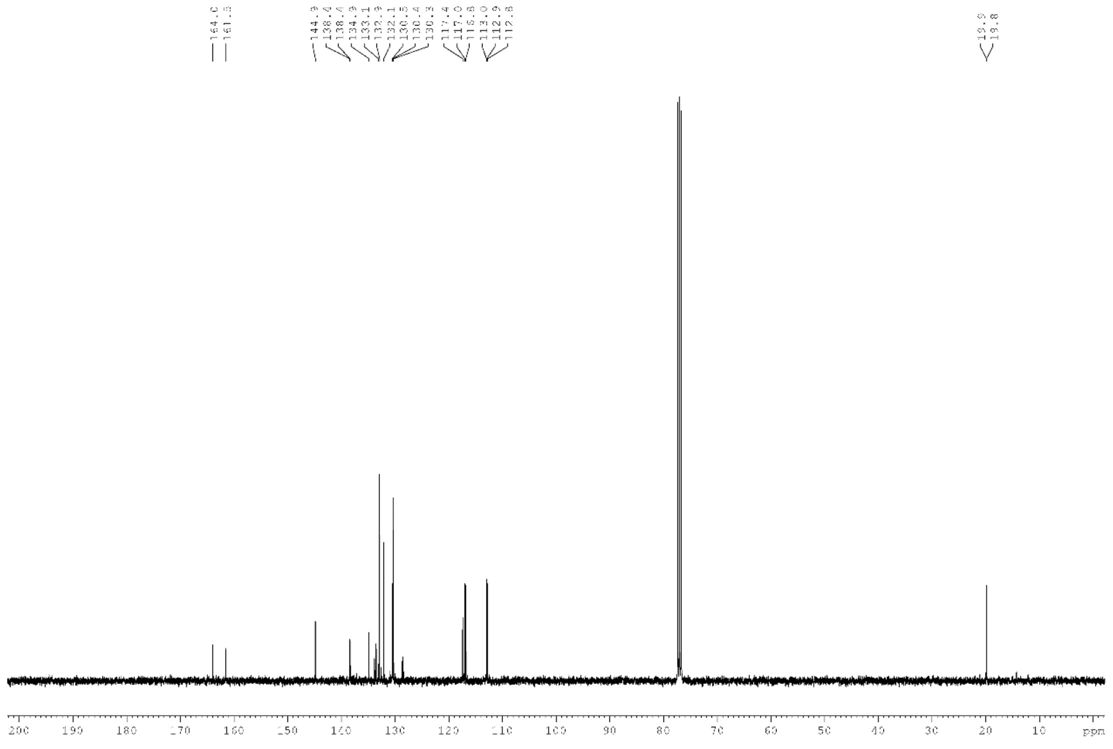

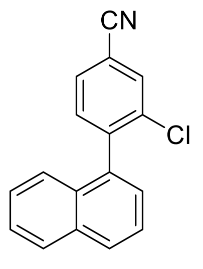
CN
Cl

12b

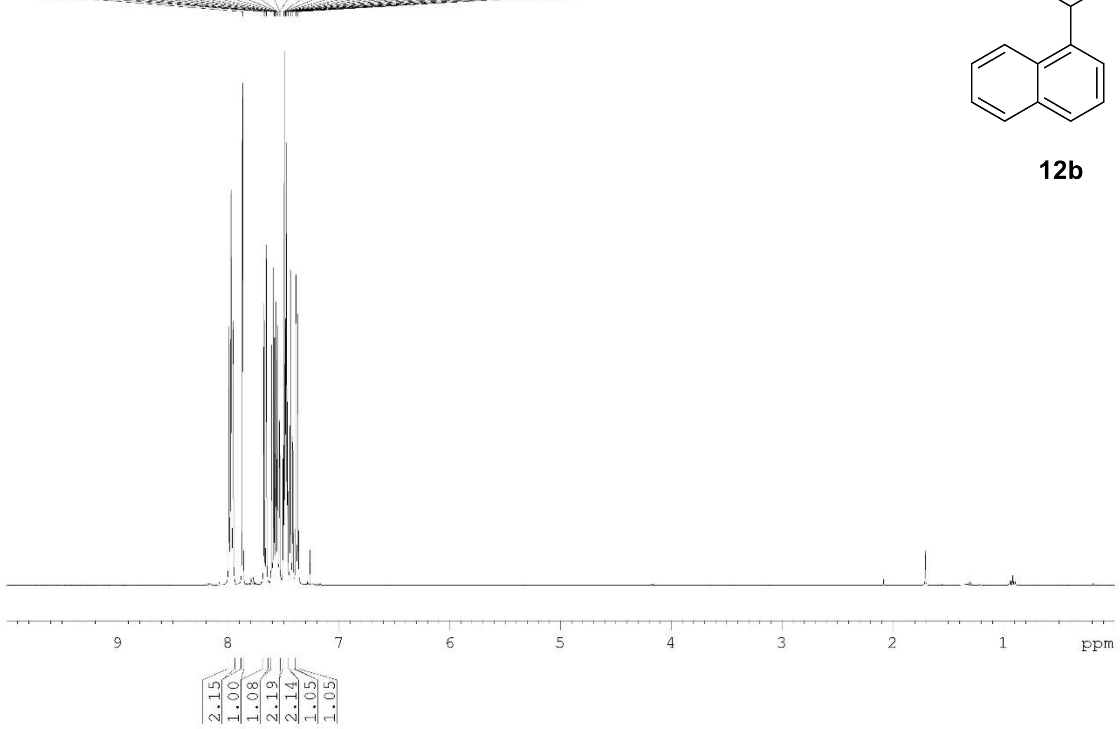
7.87
7.68
7.67
7.66
7.65
7.61
7.59
7.59
7.57
7.57
7.56
7.55
7.55
7.54
7.53
7.50
7.49
7.49
7.48
7.48
7.47
7.46
7.46
7.44
7.42
7.42
7.39
7.38
7.37
7.37
9 8 7 6 5 4 3 2 1 ppm
2.15
1.00
1.08
2.19
2.14
1.05
1.05

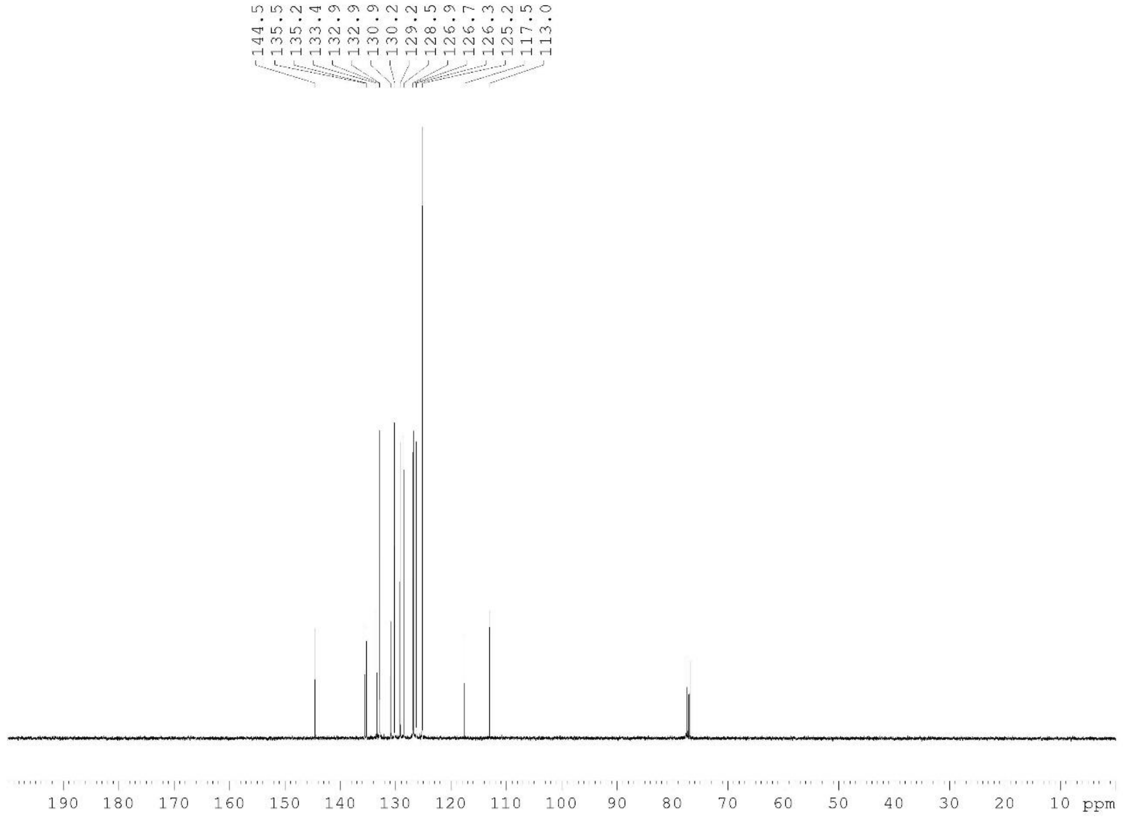
144.5
135.5
135.2
133.4
132.9
132.9
130.9
130.2
129.2
128.5
126.9
126.7
126.3
125.2
117.5
113.0
190 180 170 160 150 140 130 120 110 100 90 80 70 60 50 40 30 20 10 ppm

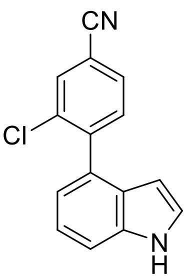
CN
Cl
N
H

13b

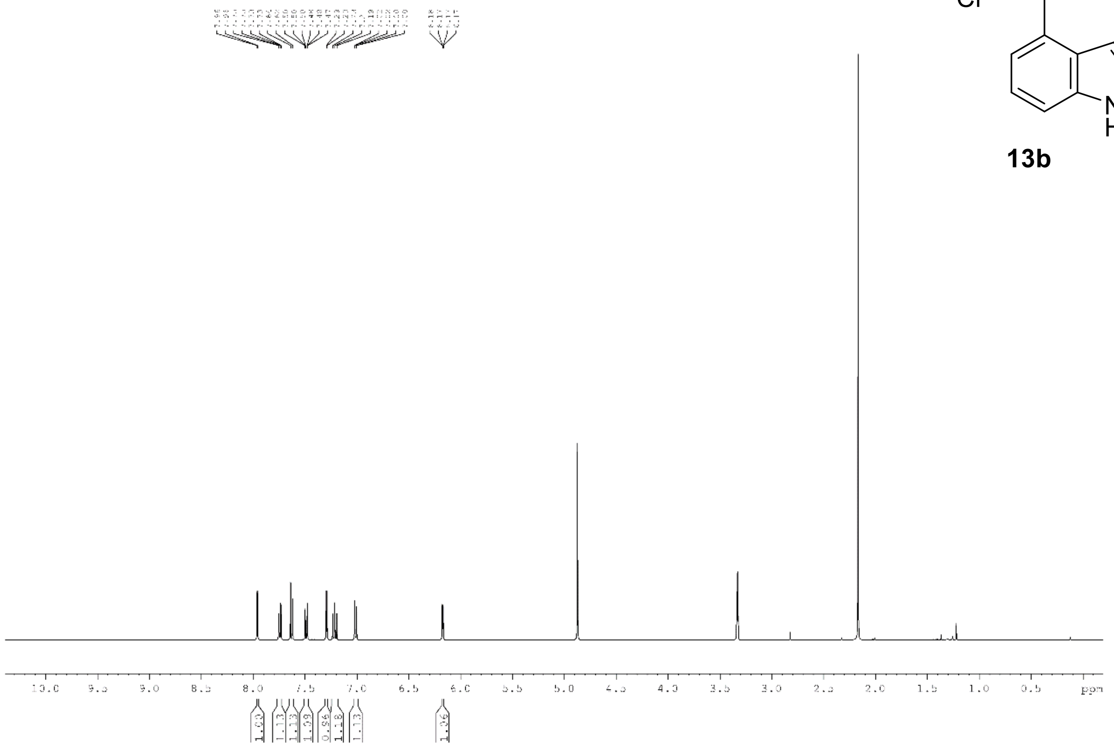
10.0 9.5 9.0 8.5 8.0 7.5 7.0 6.5 6.0 5.5 5.0 4.5 4.0 3.5 3.0 2.5 2.0 1.5 1.0 0.5 ppm

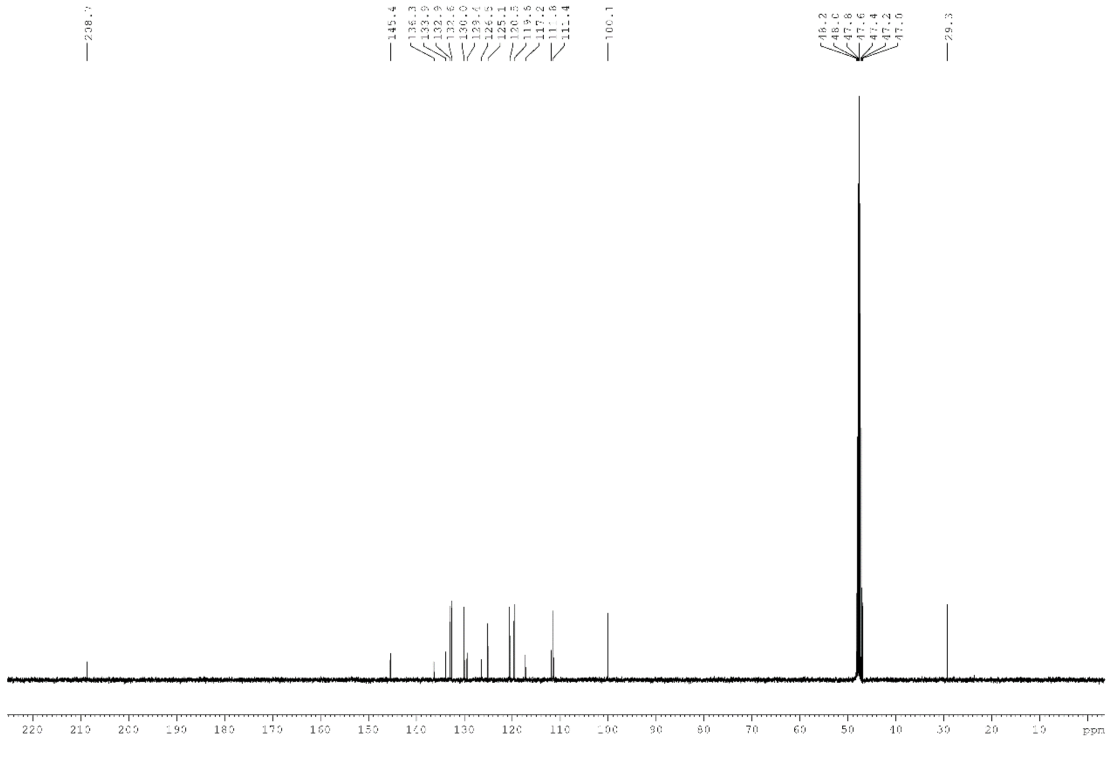
208.7
145.4
136.3
133.9
132.9
132.6
130.0
129.4
126.5
125.1
120.5
119.8
117.2
111.8
111.4
100.1
48.2
48.0
47.8
47.6
47.4
47.2
47.0
29.3
220 210 200 190 180 170 160 150 140 130 120 110 100 90 80 70 60 50 40 30 20 10 ppm

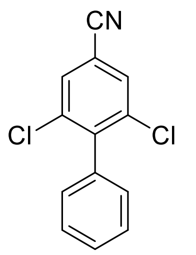

14b

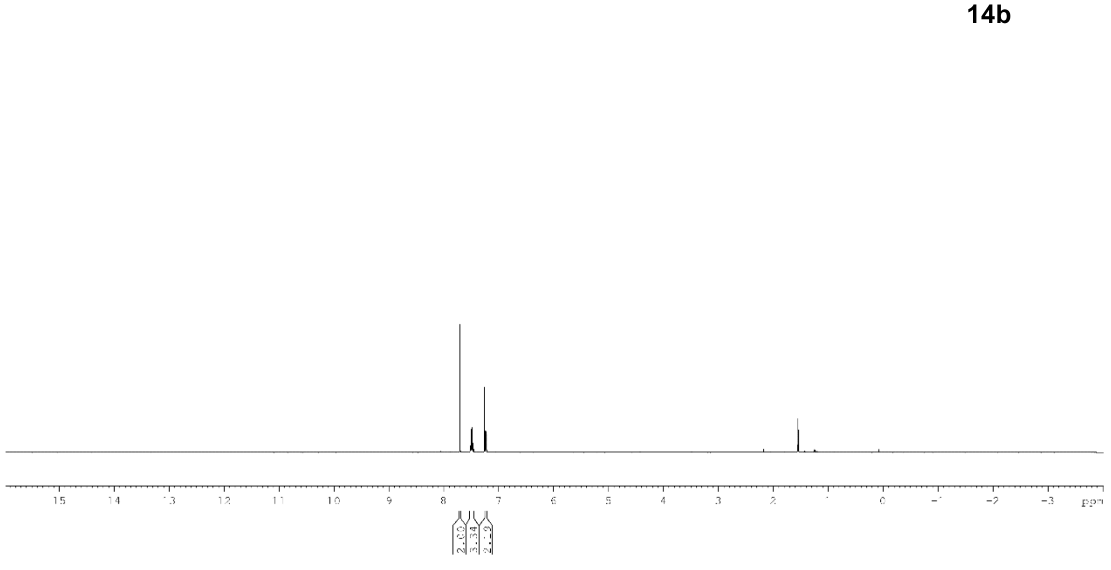

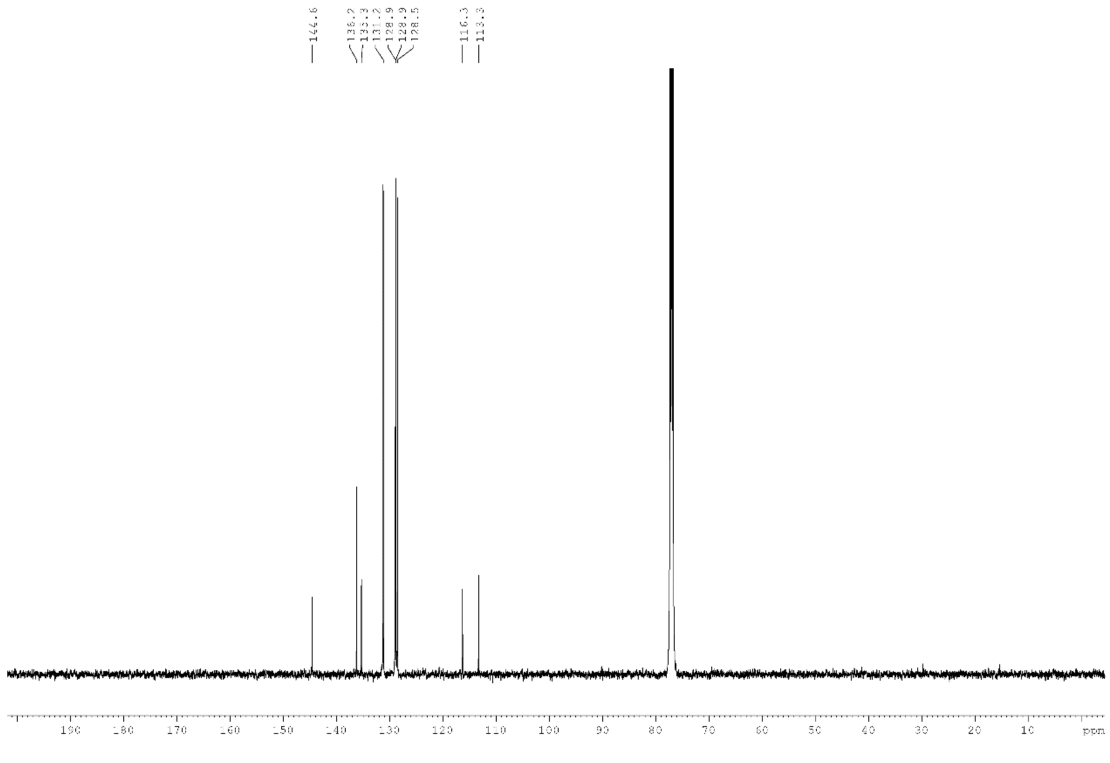

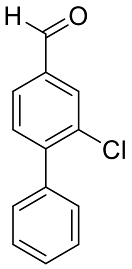
H
O
Cl

16b

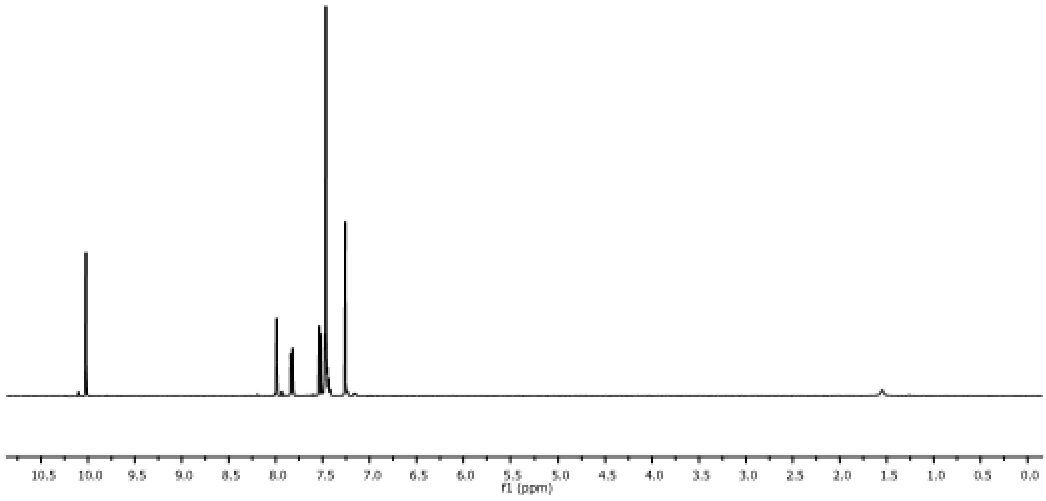
10.5
10.0
9.5
9.0
8.5
8.0
7.5
7.0
6.5
6.0
5.5
5.0
4.5
4.0
3.5
3.0
2.5
2.0
1.5
1.0
0.5
0.0
f1 (ppm)

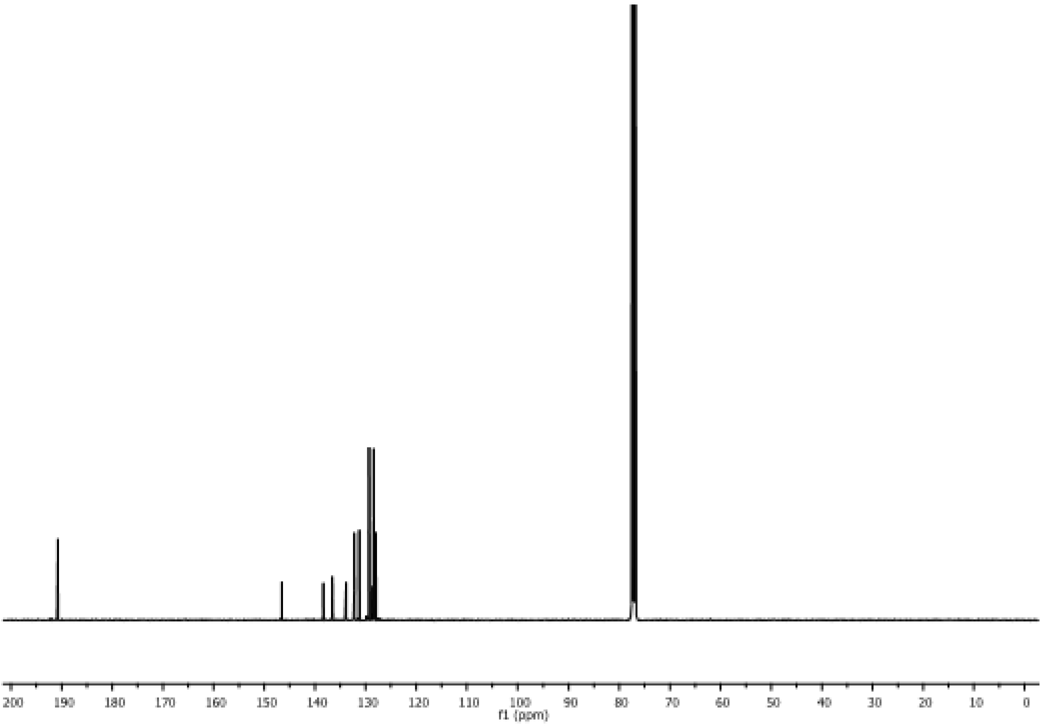
200
190
180
170
160
150
140
130
120
110
100
90
80
70
60
50
40
30
20
10
0
f1 (ppm)

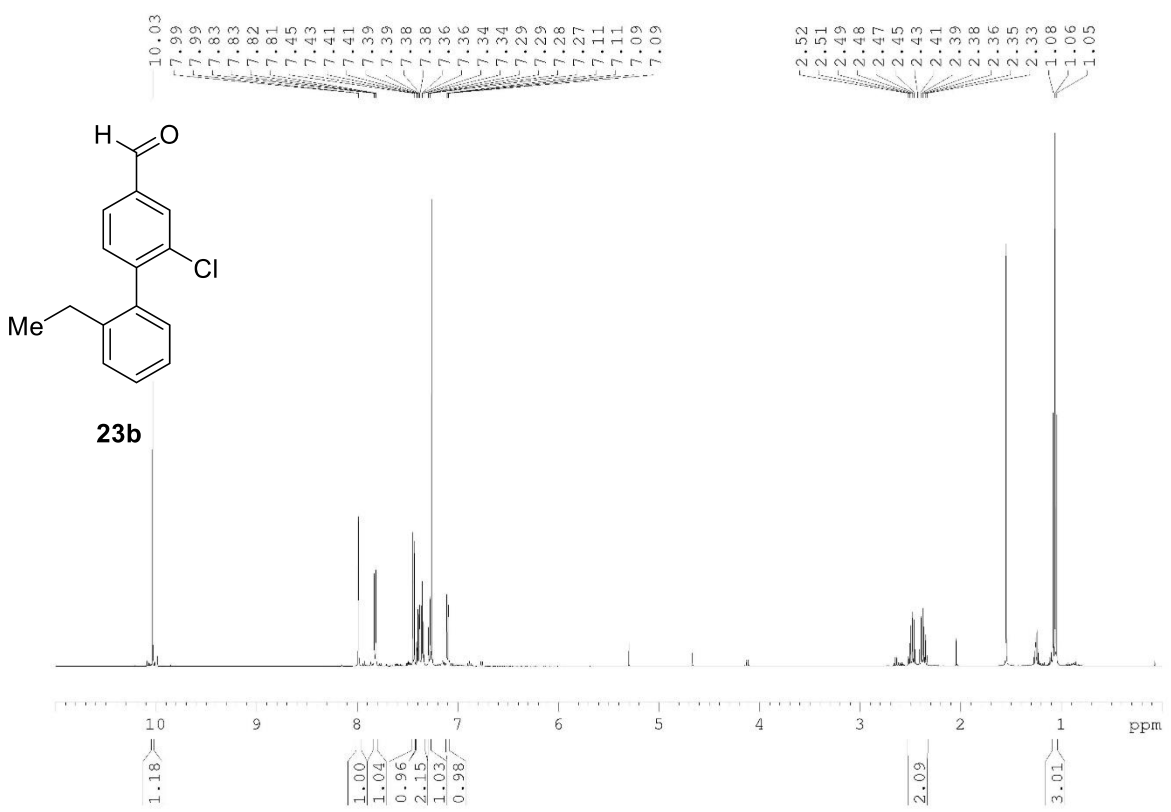
H
O
Cl
Me
10.03
7.99 7.99 7.83 7.83 7.82 7.81 7.45 7.43 7.41 7.41 7.39 7.39 7.38 7.38 7.36 7.36 7.34 7.34 7.29 7.29 7.28 7.27 7.11 7.11 7.09 7.09
2.52 2.51 2.49 2.48 2.47 2.45 2.43 2.41 2.39 2.38 2.36 2.35 2.33
1.08 1.06 1.05
10 9 8 7 6 5 4 3 2 1 ppm
1.18
1.00 1.04 0.96 2.15 1.03 0.98
2.09
3.01

23b

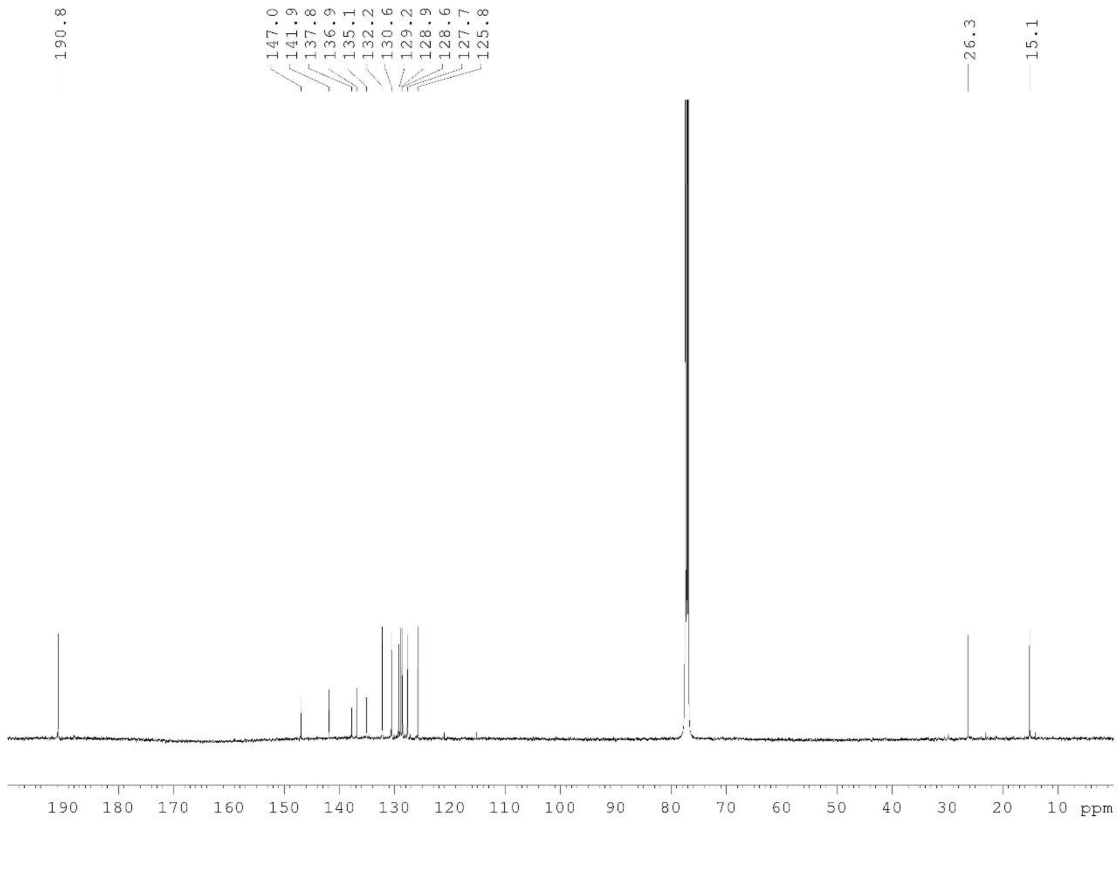
190.8
147.0 141.9 137.8 136.9 135.1 132.2 130.6 129.2 128.9 128.6 127.7 125.8
26.3
15.1
190 180 170 160 150 140 130 120 110 100 90 80 70 60 50 40 30 20 10 ppm

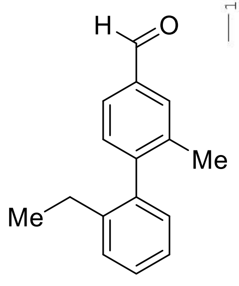
H
O
Me
Me

31b

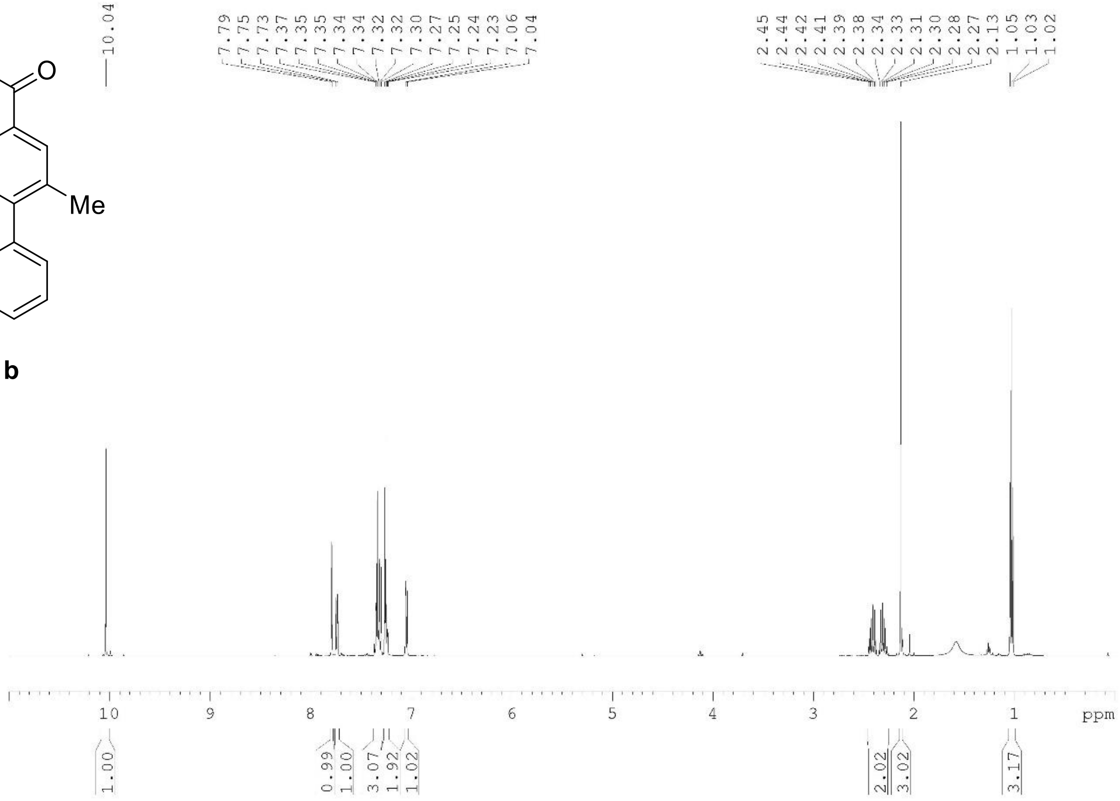
10.04
7.79 7.75 7.73 7.37 7.35 7.35 7.34 7.32 7.32 7.30 7.27 7.25 7.24 7.23 7.06 7.04
2.45 2.44 2.42 2.41 2.39 2.38 2.34 2.33 2.31 2.30 2.28 2.27 2.13 1.05 1.03 1.02
10 9 8 7 6 5 4 3 2 1 ppm
1.00 0.99 1.00 3.07 1.92 1.02 2.02 3.02 3.17

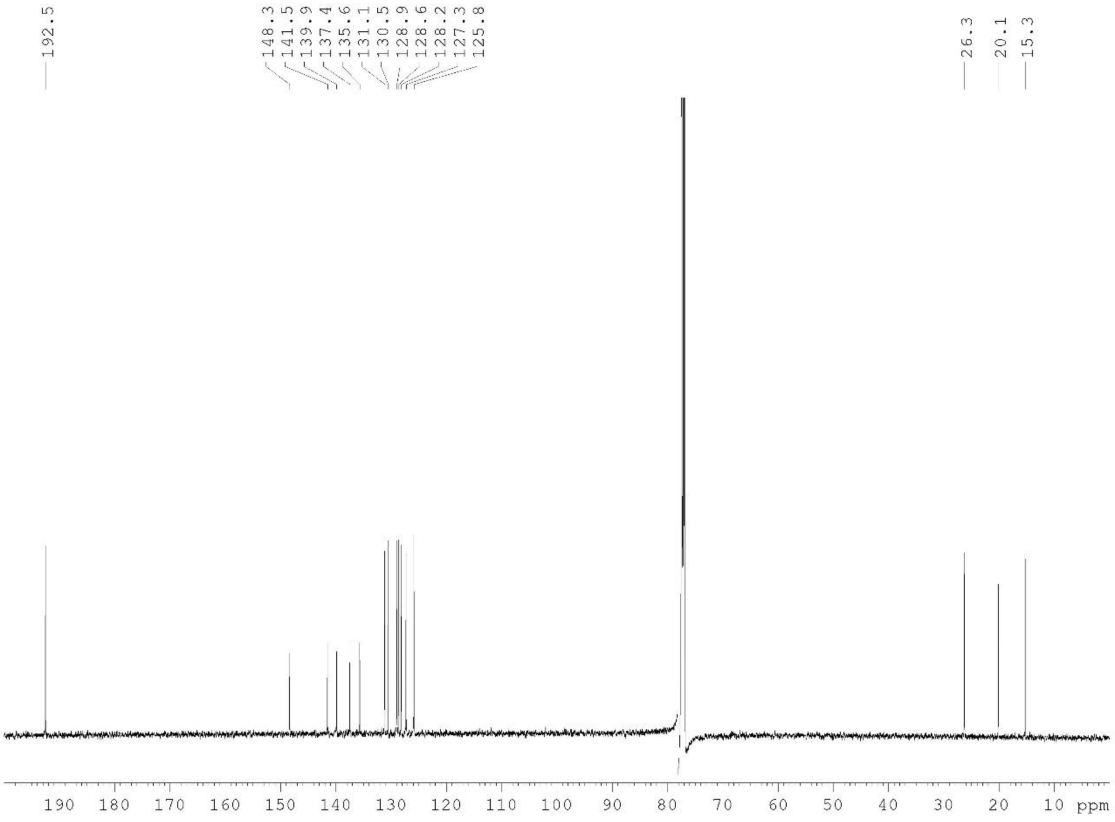
192.5
148.3 141.5 139.9 137.4 135.6 131.1 130.5 128.9 128.6 128.2 127.3 125.8
26.3 20.1 15.3
190 180 170 160 150 140 130 120 110 100 90 80 70 60 50 40 30 20 10 ppm

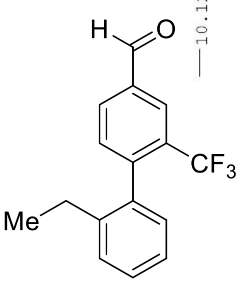
H
O
CF3
Me

32b

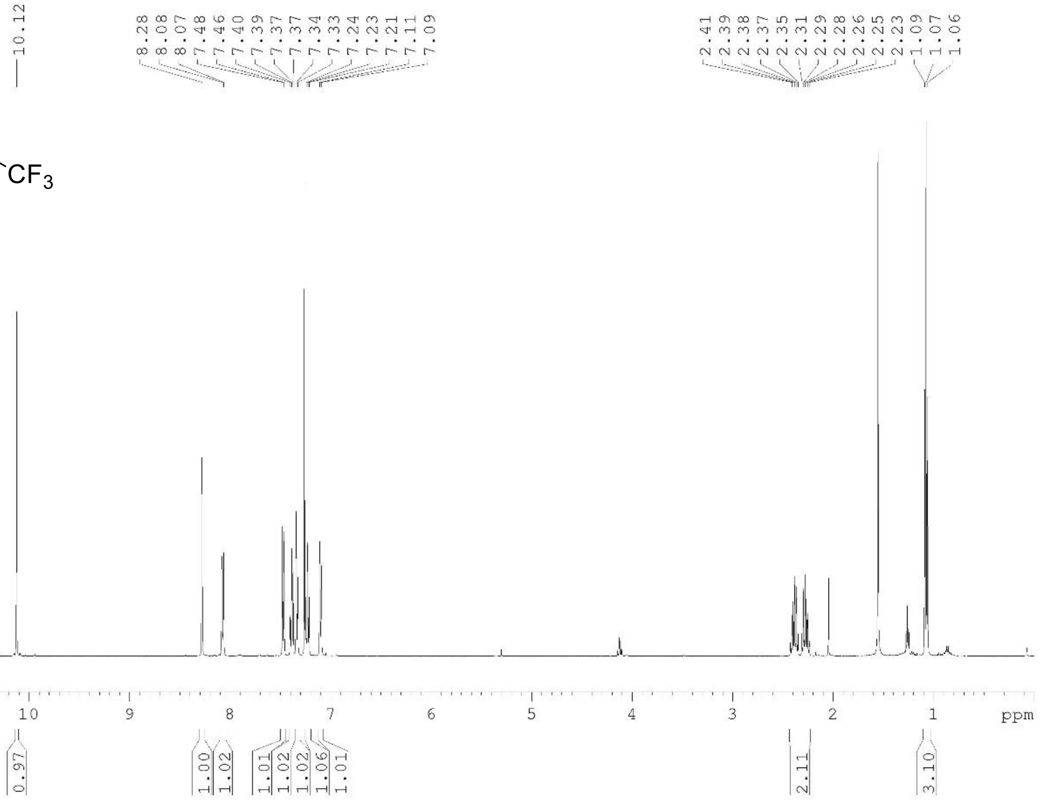
10.12
8.28 8.08 8.07 7.48 7.46 7.40 7.39 7.37 7.37 7.34 7.33 7.24 7.23 7.21 7.11 7.09
2.41 2.39 2.38 2.37 2.35 2.31 2.29 2.28 2.26 2.25 2.23 1.09 1.07 1.06
10 9 8 7 6 5 4 3 2 1 ppm
0.97 1.00 1.02 1.01 1.02 1.02 1.06 1.01 2.11 3.10

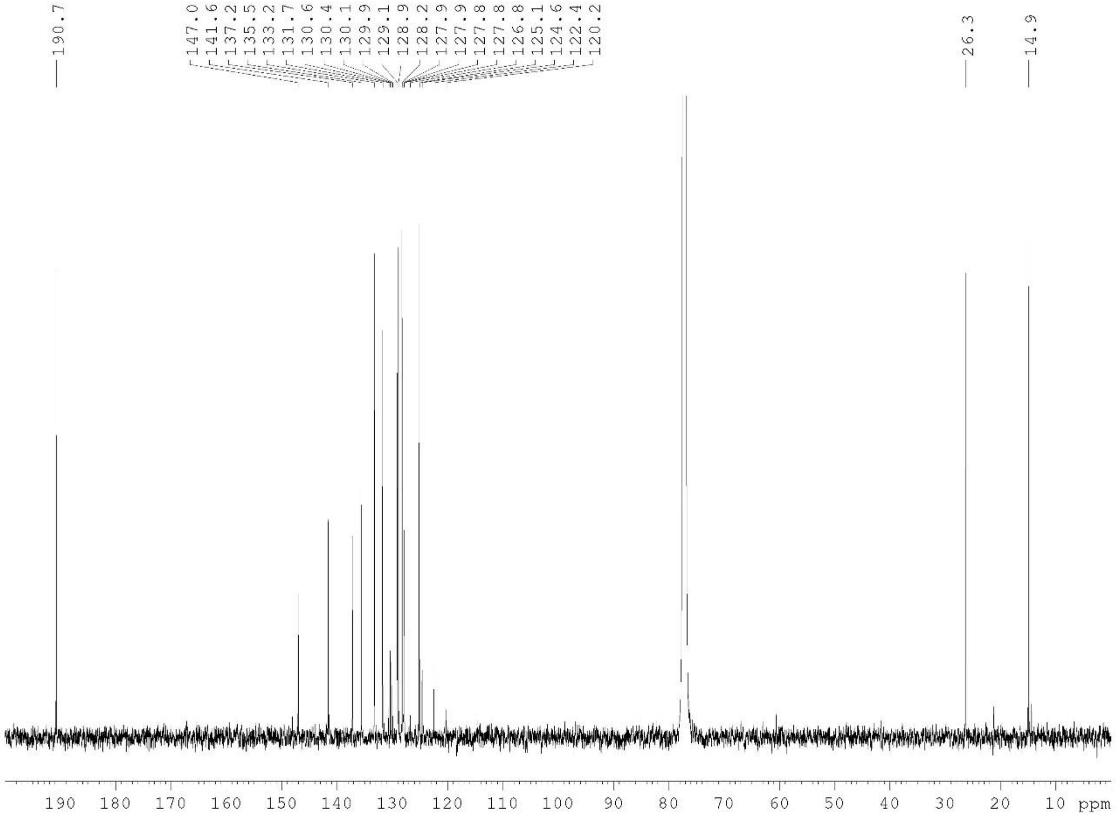
190.7
147.0 141.6 137.2 135.5 133.2 131.7 130.6 130.4 130.1 129.9 129.1 128.9 128.2 127.9 127.9 127.8 127.8 126.8 125.1 124.6 122.4 120.2
26.3
14.9
190 180 170 160 150 140 130 120 110 100 90 80 70 60 50 40 30 20 10 ppm

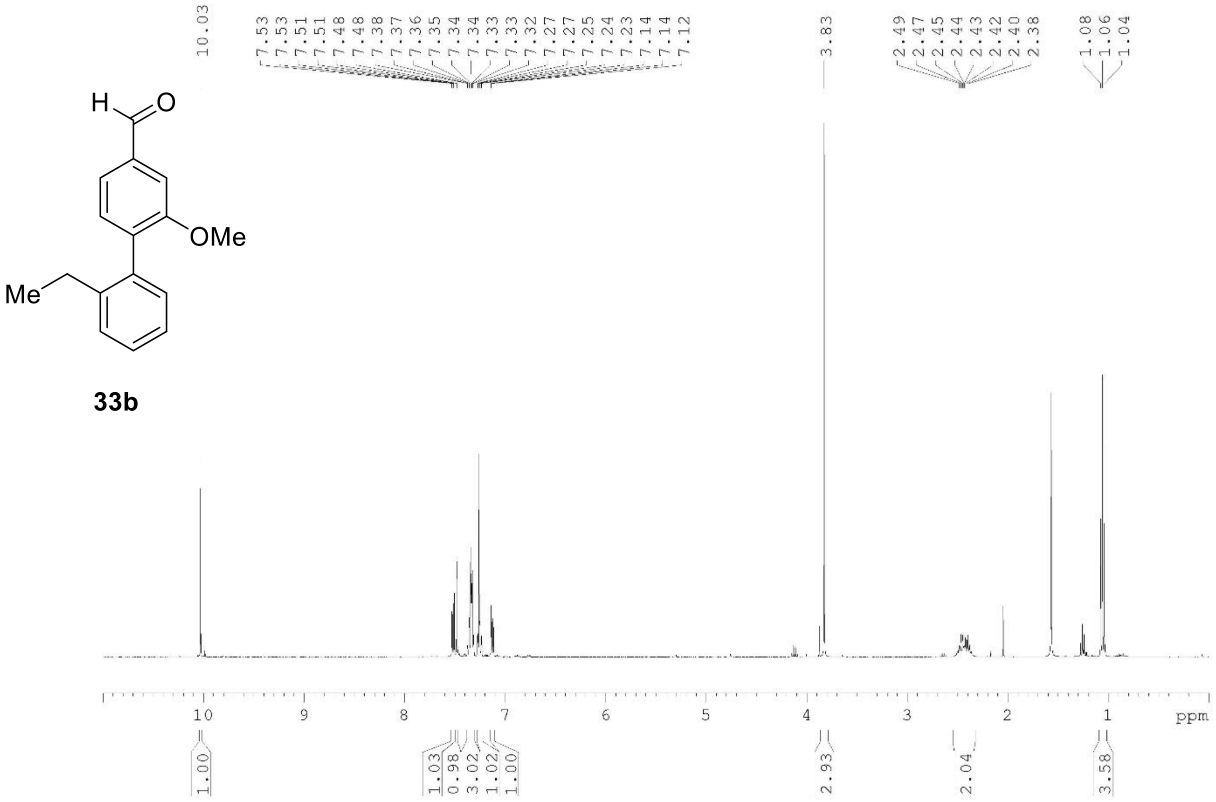
H
O
OMe
Me
33b
10.03
7.53
7.53
7.51
7.51
7.48
7.48
7.38
7.37
7.36
7.35
7.34
7.34
7.33
7.33
7.32
7.27
7.25
7.24
7.23
7.14
7.14
7.12
3.83
2.49
2.47
2.45
2.44
2.43
2.42
2.40
2.38
1.08
1.06
1.04
10 9 8 7 6 5 4 3 2 1 ppm
1.00
1.03
0.98
3.02
1.02
1.00
2.93
2.04
3.58

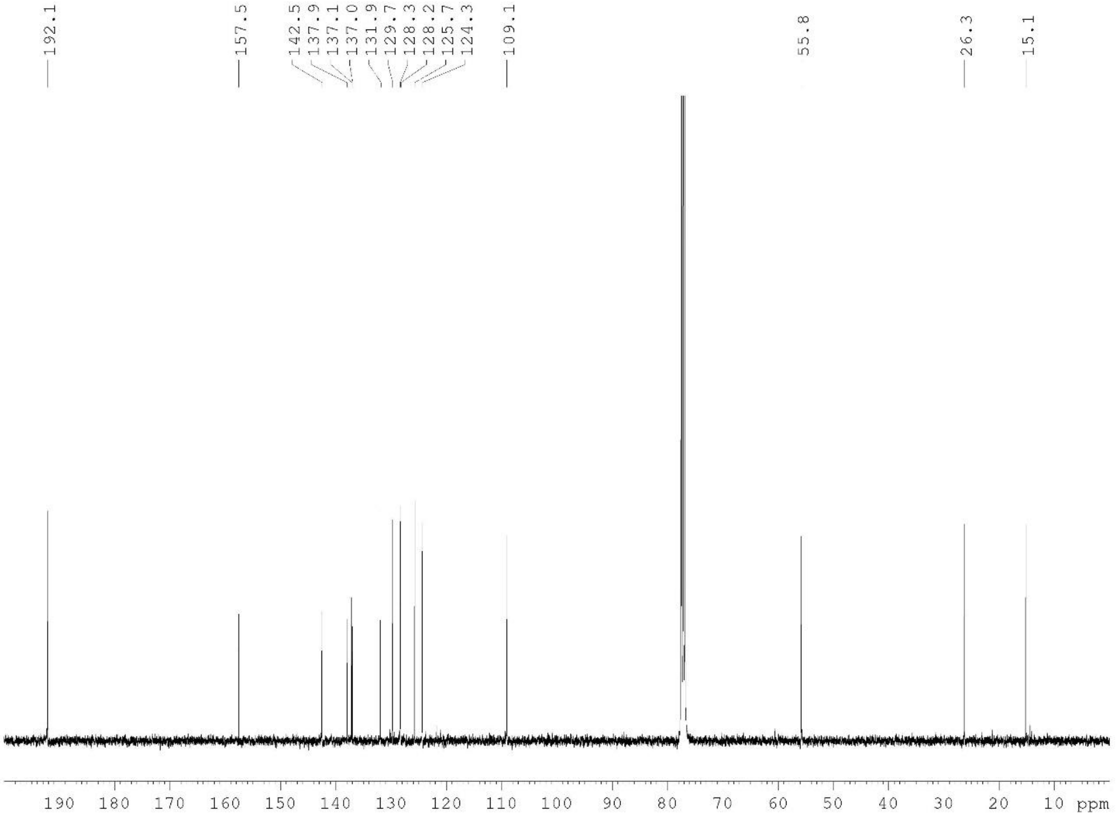
192.1
157.5
142.5
137.9
137.1
137.0
131.9
129.7
128.3
128.2
125.7
124.3
109.1
55.8
26.3
15.1
190 180 170 160 150 140 130 120 110 100 90 80 70 60 50 40 30 20 10 ppm

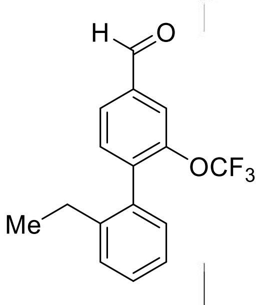

H
O
OCF3
Me

34b

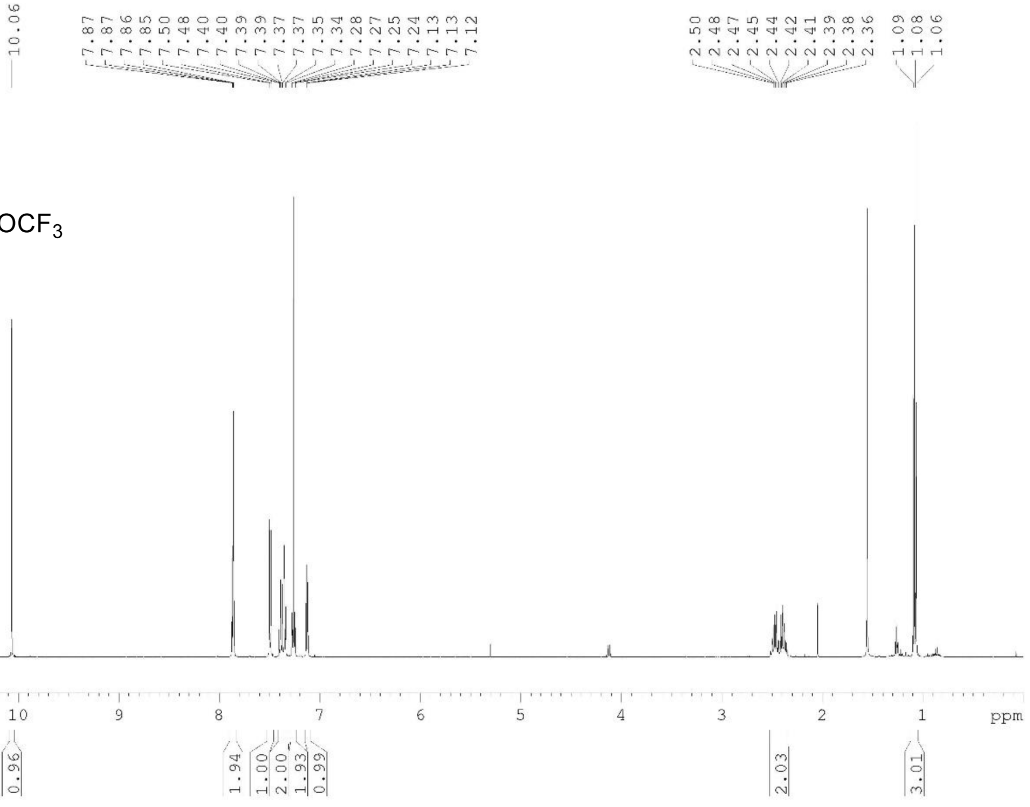

10.06
7.87 7.87 7.86 7.85 7.50 7.48 7.40 7.40 7.39 7.39 7.37 7.37 7.35 7.34 7.28 7.27 7.25 7.24 7.13 7.13 7.12
2.50 2.48 2.47 2.45 2.44 2.42 2.41 2.39 2.38 2.36
1.09 1.08 1.06
10 9 8 7 6 5 4 3 2 1 ppm
0.96
1.94 1.00 2.00 1.93 0.99
2.03
3.01

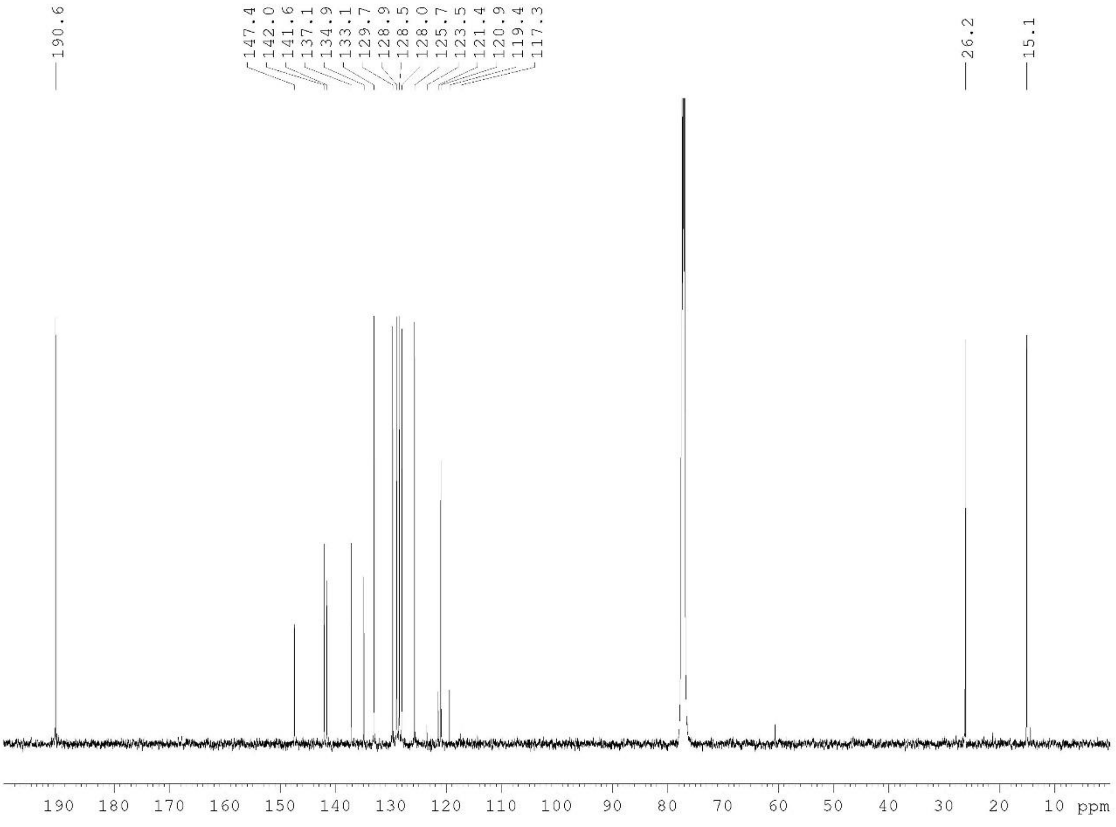

190.6
147.4 142.0 141.6 137.1 134.9 133.1 129.7 128.9 128.5 128.0 125.7 123.5 121.4 120.9 119.4 117.3
26.2
15.1
190 180 170 160 150 140 130 120 110 100 90 80 70 60 50 40 30 20 10 ppm

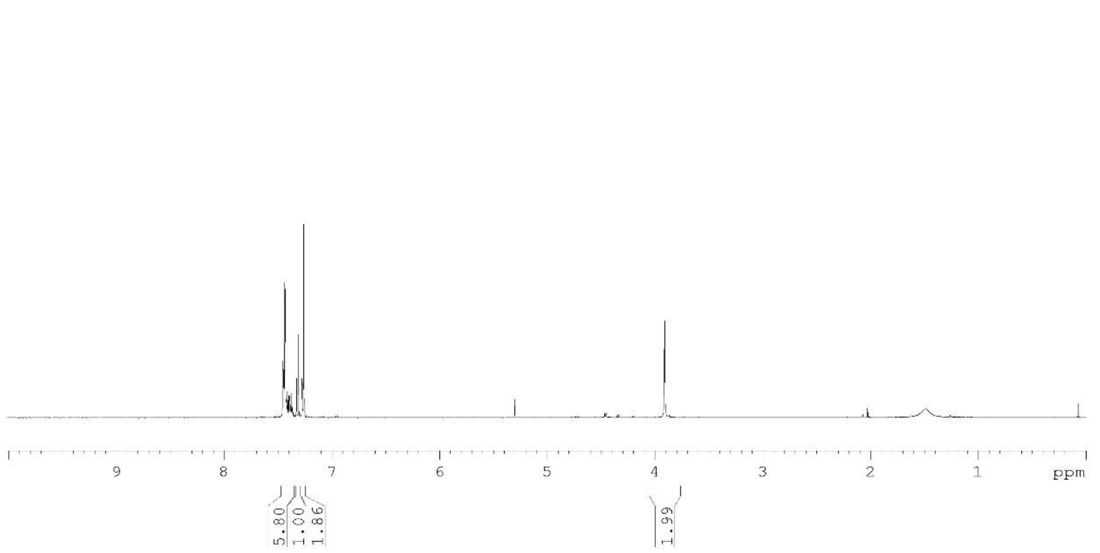
7.45
7.45
7.44
7.44
7.43
7.43
7.42
7.33
7.31
7.28
7.27
7.25
3.91
9
8
7
6
5
4
3
2
1
ppm
5.80
1.00
1.86
1.99

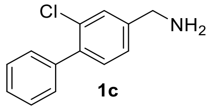
Cl
NH2
1c

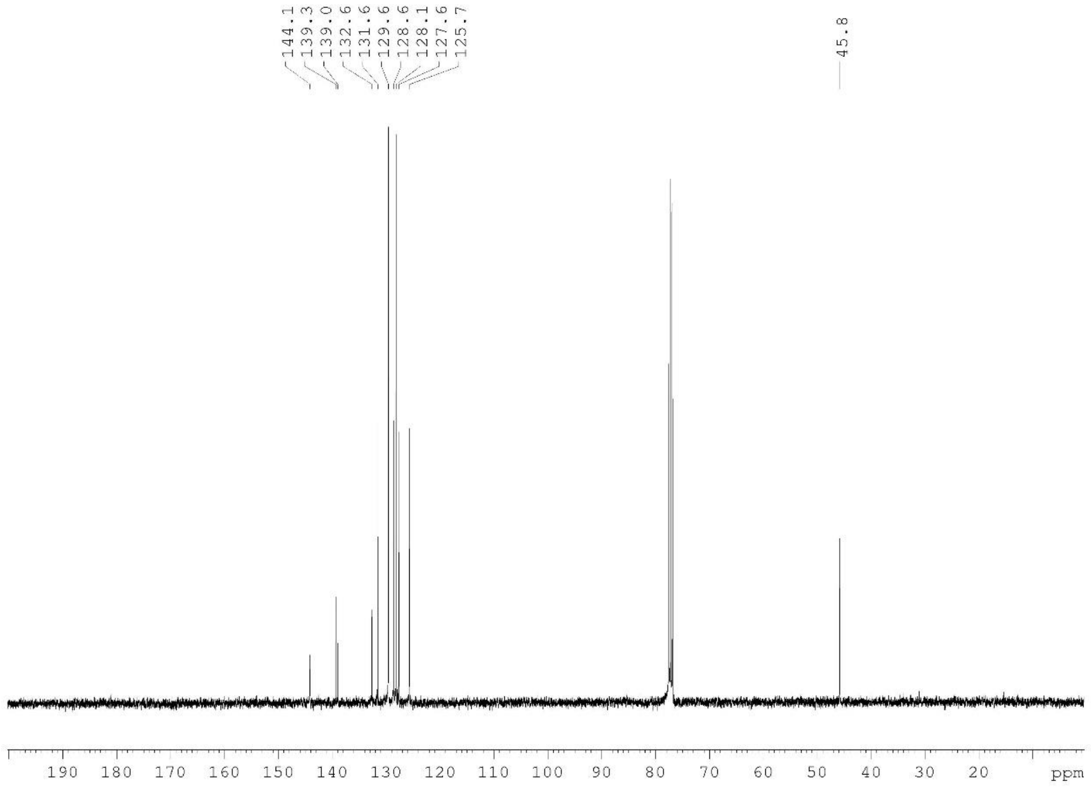
144.1
139.3
139.0
132.6
131.6
129.6
128.6
128.1
127.6
125.7
45.8
190
180
170
160
150
140
130
120
110
100
90
80
70
60
50
40
30
20
ppm

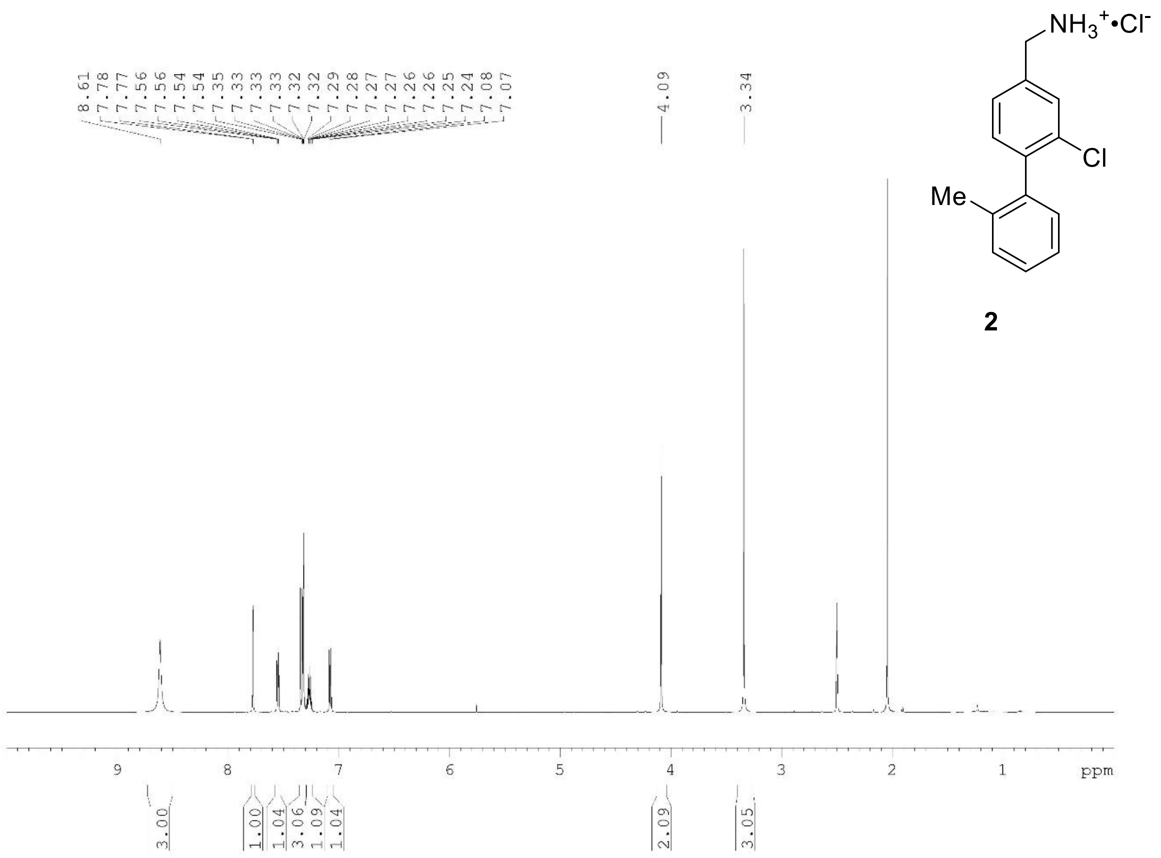
NH3+•Cl-
Cl
Me
2
8.61
7.78
7.77
7.56
7.56
7.54
7.54
7.35
7.33
7.33
7.33
7.32
7.32
7.29
7.28
7.27
7.27
7.26
7.26
7.25
7.24
7.08
7.07
4.09
3.34
9
8
7
6
5
4
3
2
1
ppm
3.00
1.00
1.04
3.06
1.09
1.04
2.09
3.05

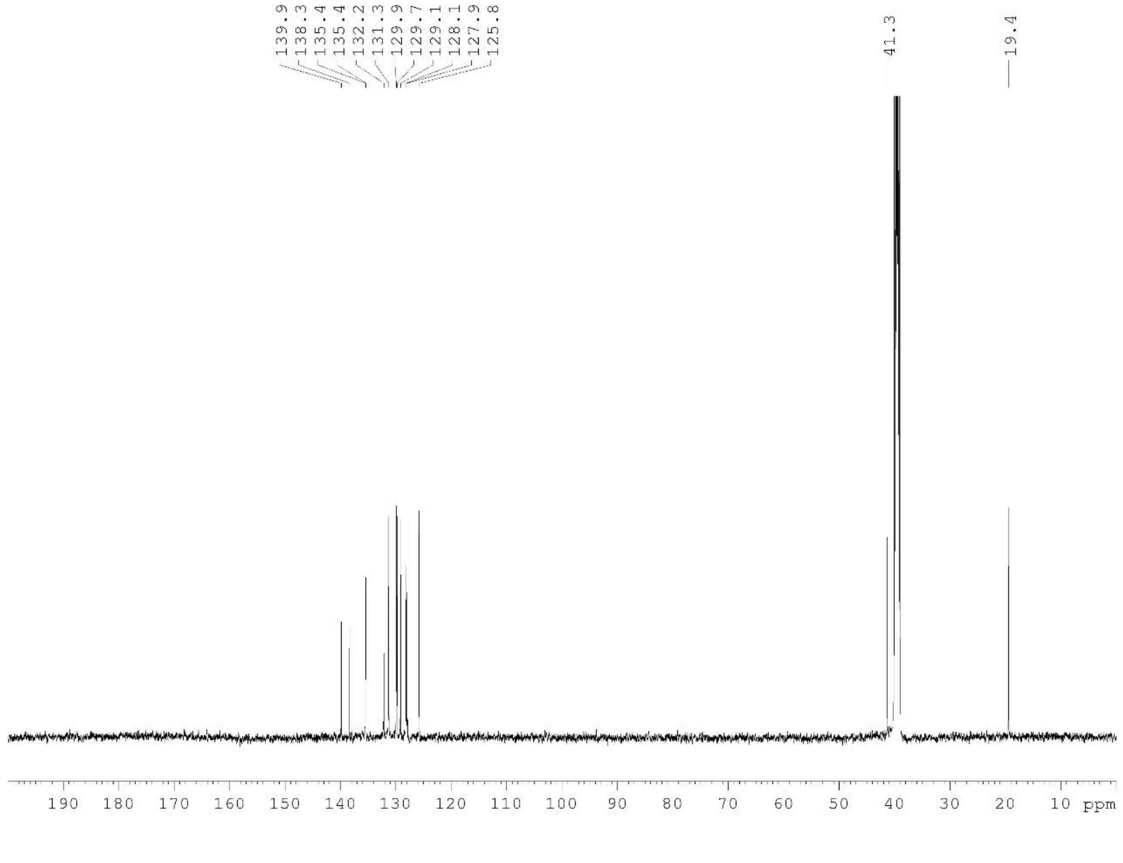
139.9
138.3
135.4
135.4
132.2
131.3
129.9
129.7
129.1
128.1
127.9
125.8
41.3
19.4
190
180
170
160
150
140
130
120
110
100
90
80
70
60
50
40
30
20
10
ppm

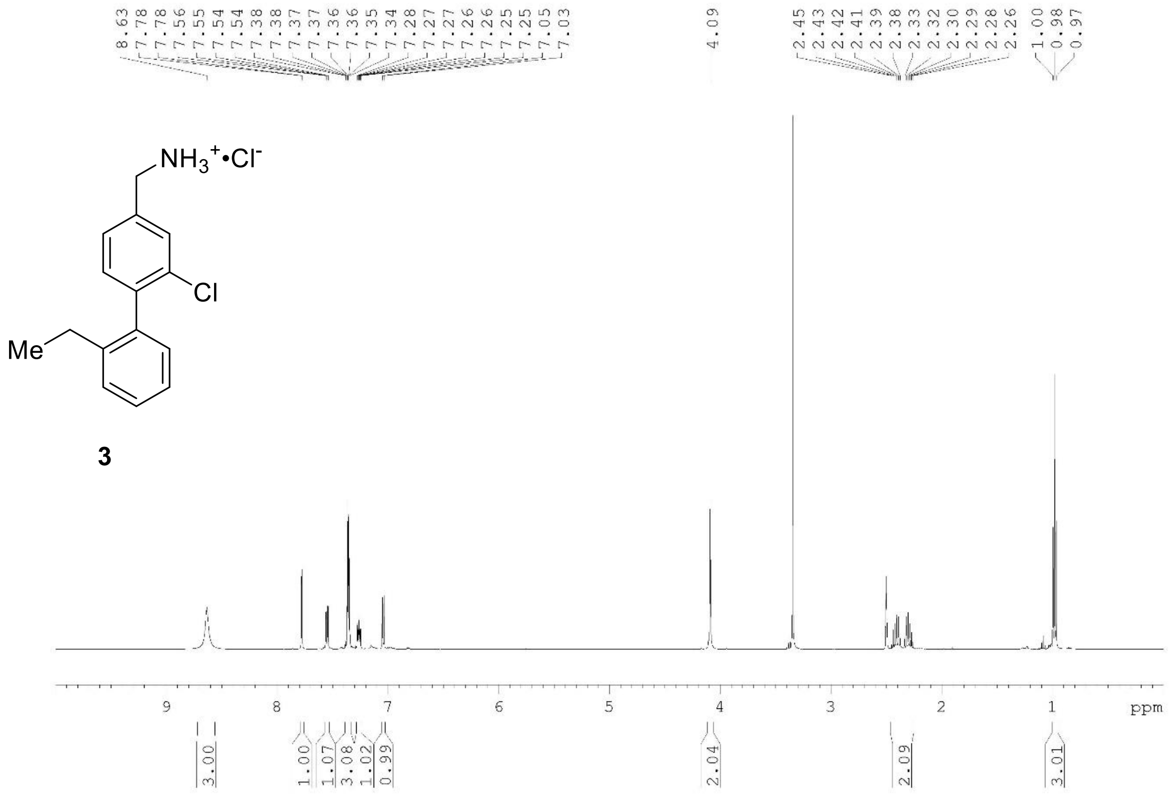
NH3+•Cl-
Cl
Me
3
8.63 7.78 7.78 7.56 7.55 7.54 7.54 7.38 7.38 7.37 7.37 7.36 7.36 7.35 7.34 7.28 7.27 7.27 7.26 7.26 7.25 7.25 7.05 7.03
4.09
2.45 2.43 2.42 2.41 2.39 2.38 2.33 2.32 2.30 2.29 2.28 2.26
1.00 0.98 0.97
9 8 7 6 5 4 3 2 1 ppm
3.00 1.00 1.07 3.08 1.02 0.99 2.04 2.09 3.01

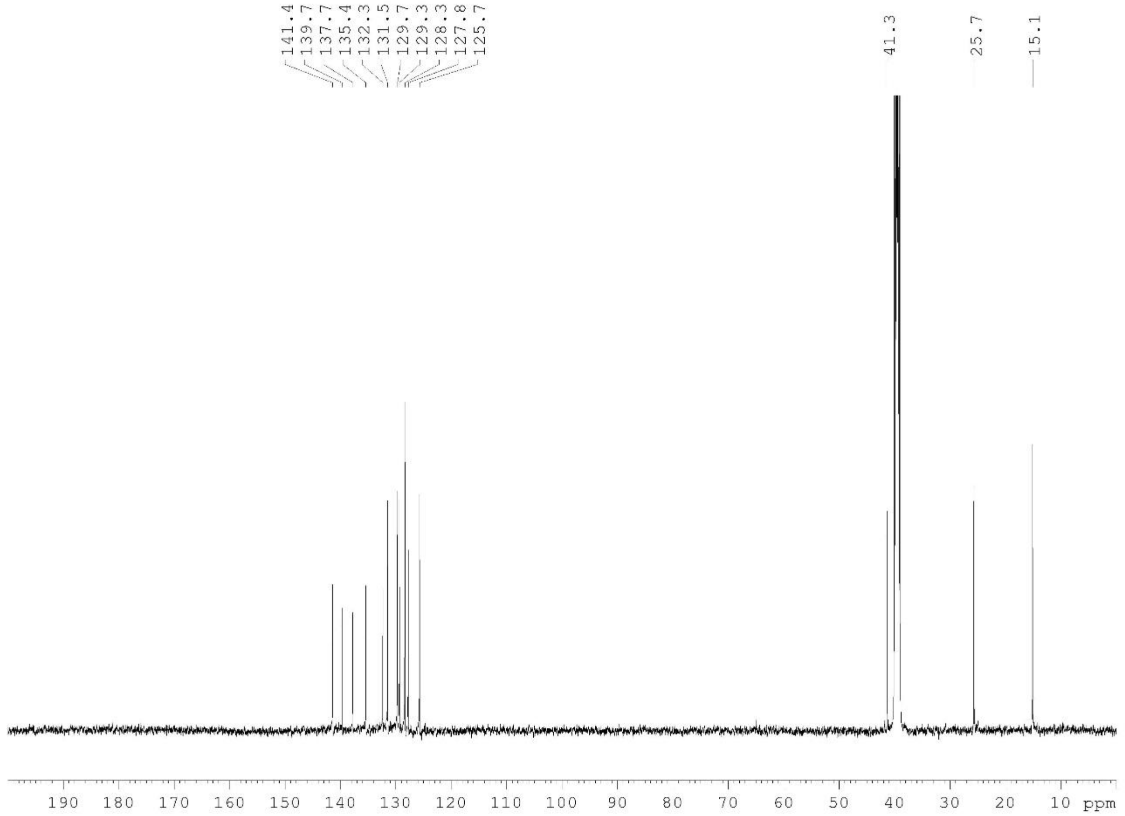
141.4 139.7 137.7 135.4 132.3 131.5 129.7 129.3 128.3 127.8 125.7
41.3
25.7
15.1
190 180 170 160 150 140 130 120 110 100 90 80 70 60 50 40 30 20 10 ppm

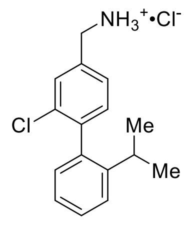

4

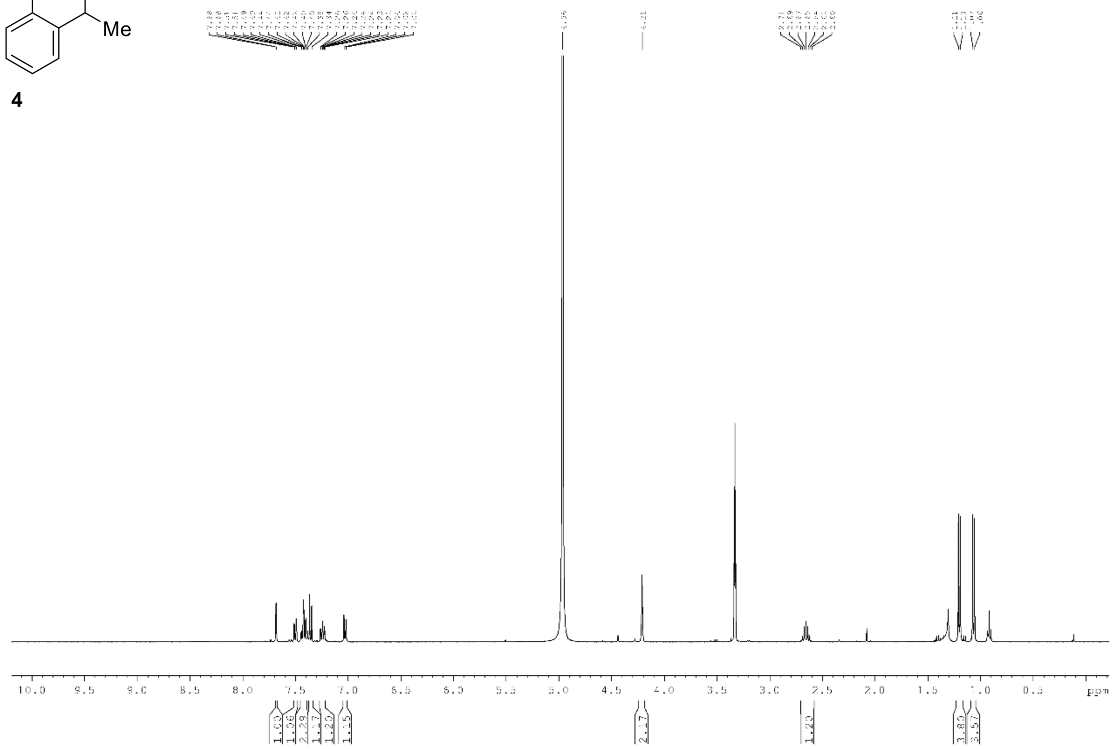

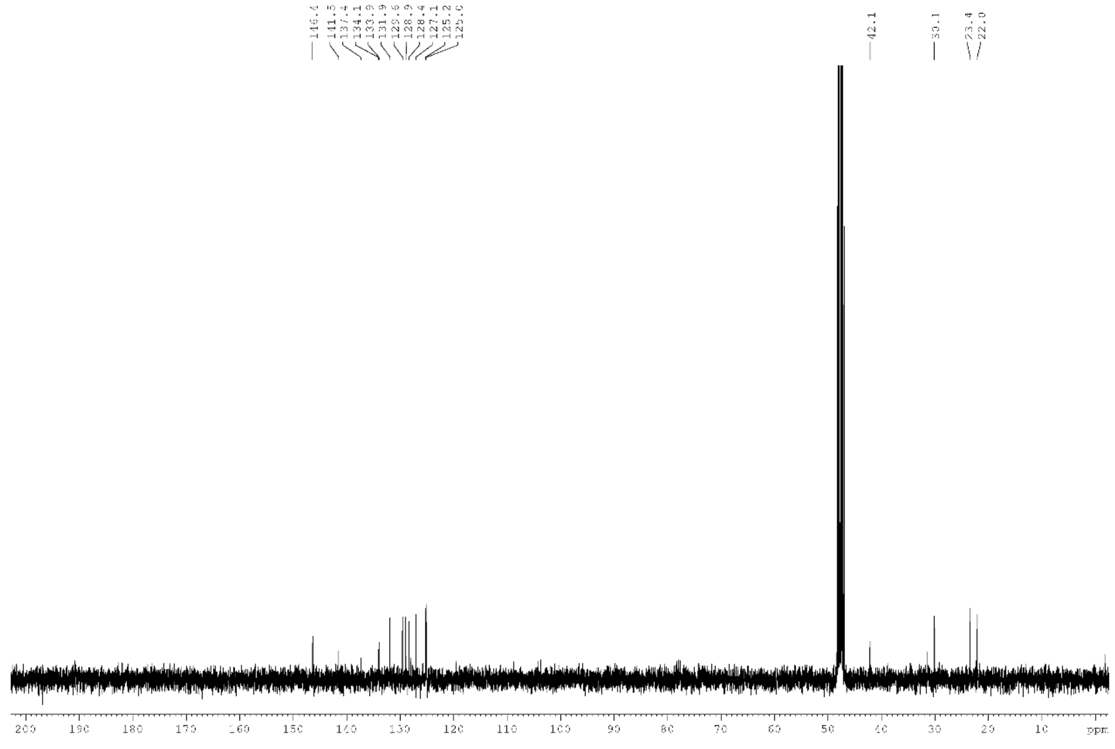

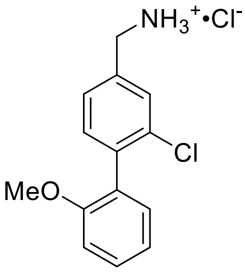
NH3+•Cl-
Cl
MeO

5

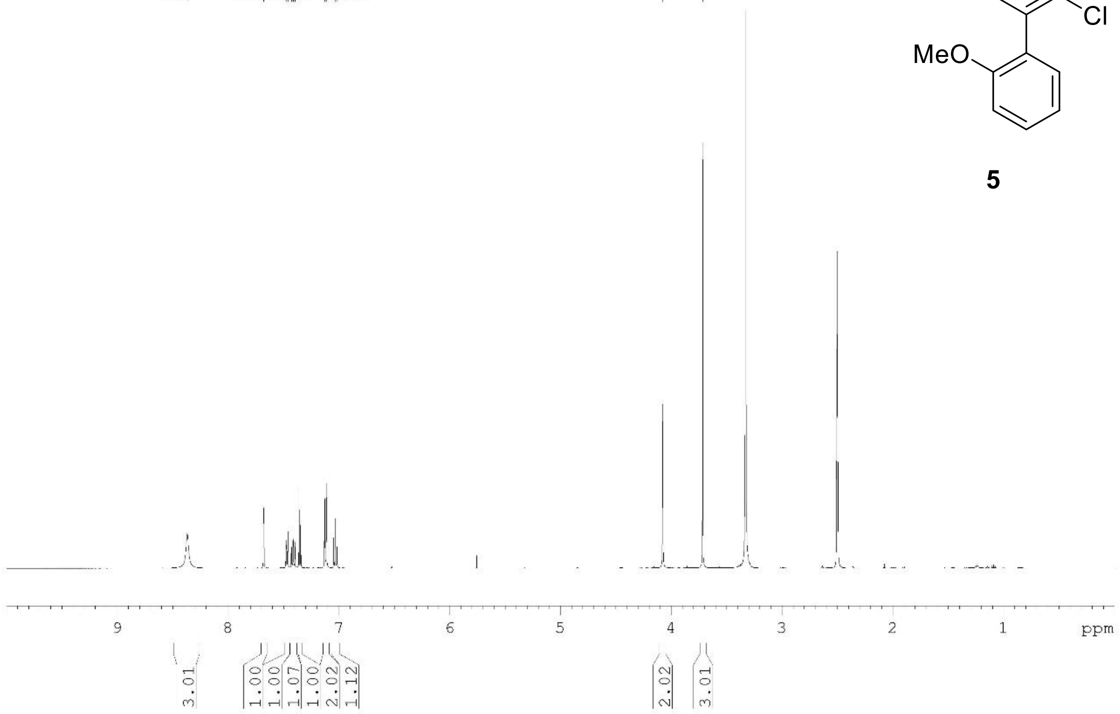
8.37 7.68 7.68 7.48 7.47 7.46 7.46 7.43 7.43 7.41 7.41 7.41 7.40 7.39 7.36 7.34 7.13 7.12 7.11 7.11 7.05 7.04 7.03 7.03 7.02 7.01
4.08
3.72
9 8 7 6 5 4 3 2 1 ppm
3.01
1.00 1.00 1.07 1.00 2.02 1.12
2.02
3.01

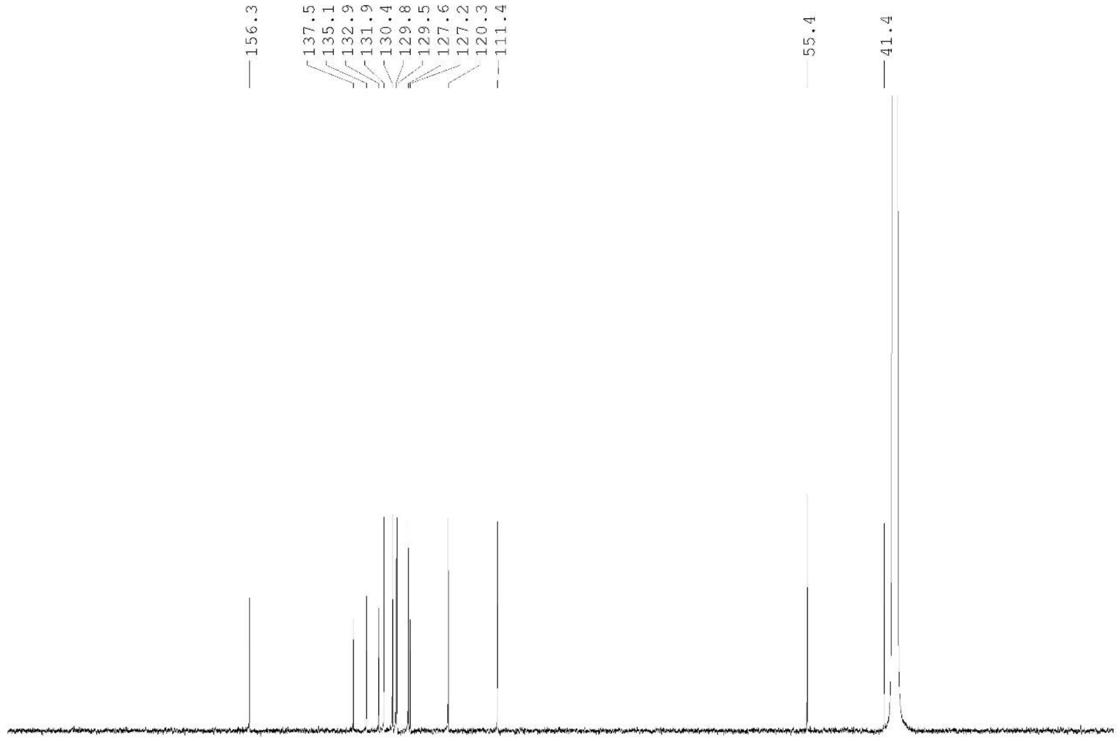
156.3
137.5 135.1 132.9 131.9 130.4 129.8 129.5 127.6 127.2 120.3 111.4
55.4
41.4
190 180 170 160 150 140 130 120 110 100 90 80 70 60 50 40 30 20 10 ppm

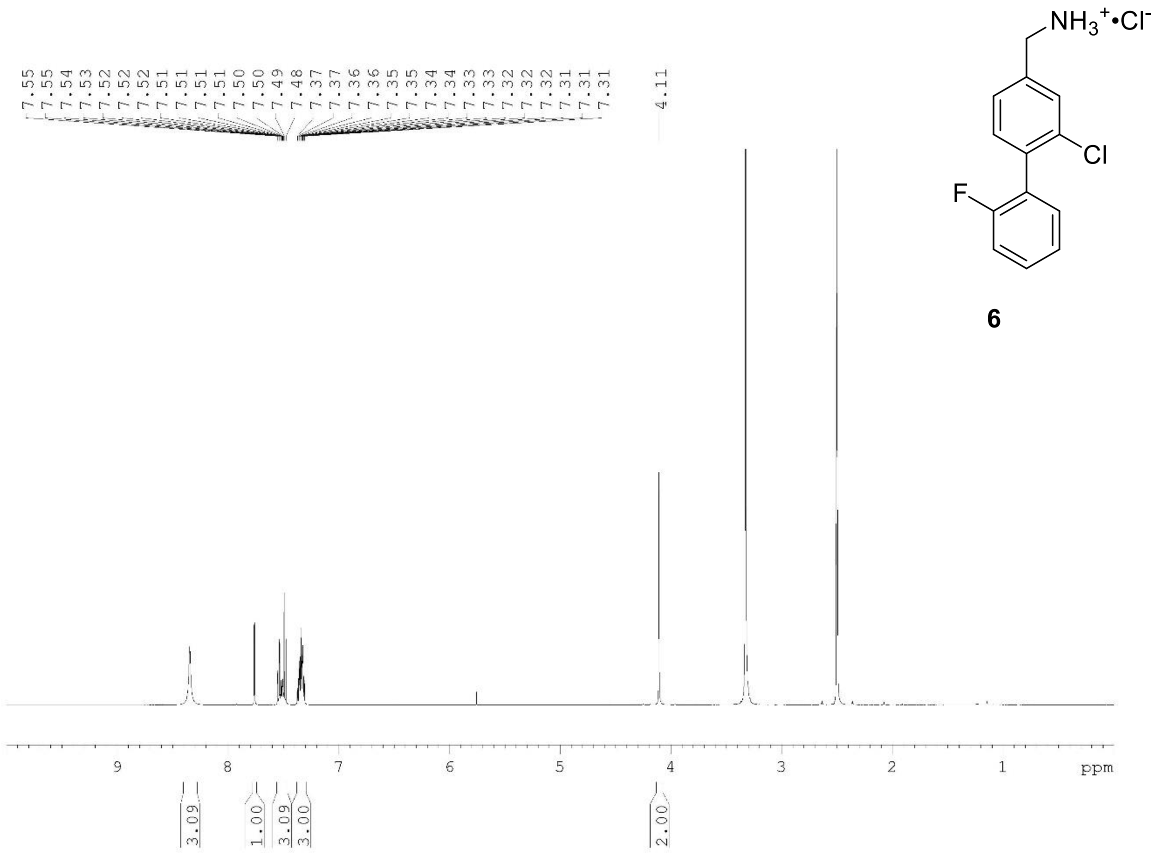

7.55
7.55
7.54
7.53
7.52
7.52
7.52
7.51
7.51
7.51
7.50
7.50
7.49
7.48
7.37
7.37
7.36
7.36
7.35
7.35
7.34
7.34
7.33
7.33
7.32
7.32
7.32
7.31
7.31
7.31
4.11
9
8
7
6
5
4
3
2
1
ppm
3.09
1.00
3.09
3.00
2.00
NH_3^+•Cl^-
Cl
F

6

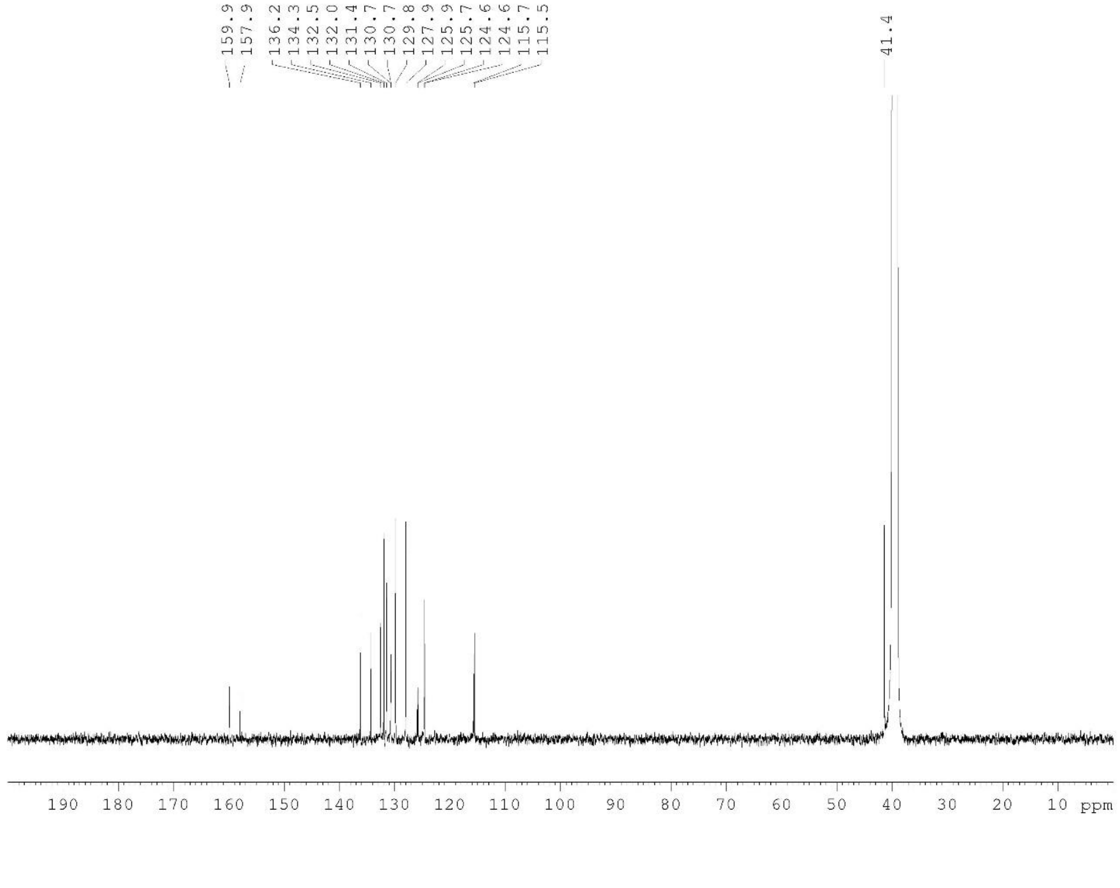

159.9
157.9
136.2
134.3
132.5
132.0
131.4
130.7
130.7
129.8
127.9
125.9
125.7
124.6
124.6
115.7
115.5
41.4
190
180
170
160
150
140
130
120
110
100
90
80
70
60
50
40
30
20
10
ppm

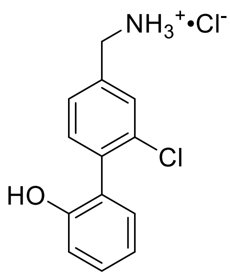
NH3+•Cl-
Cl
HO

7

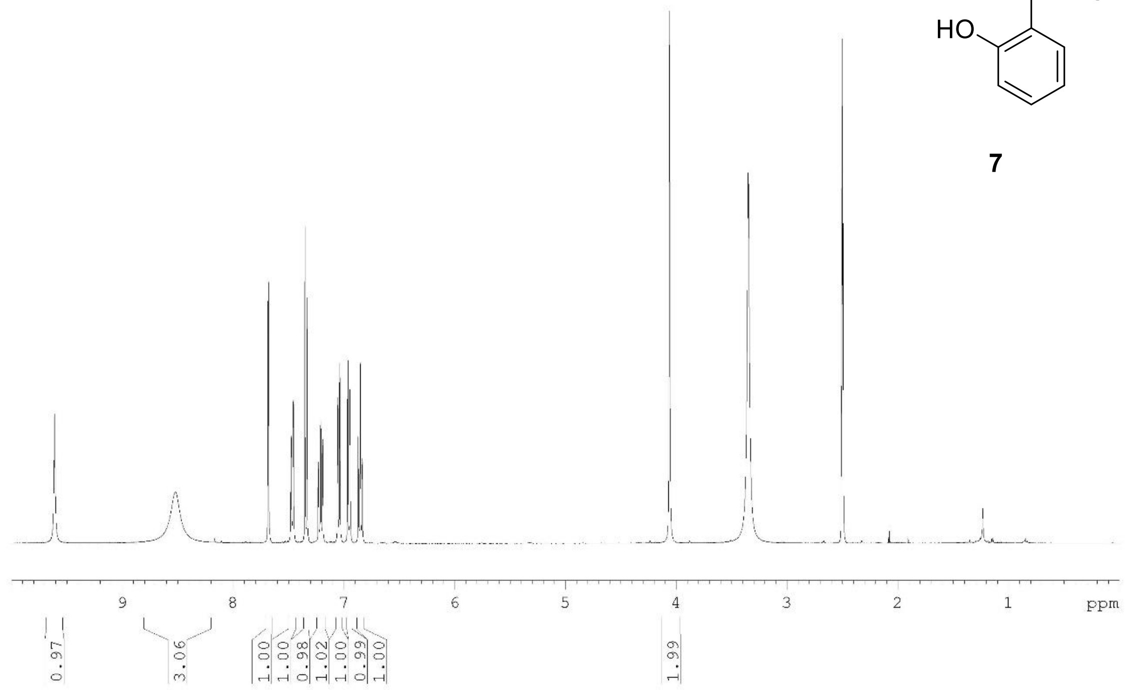
8.52
7.68
7.68
7.48
7.47
7.46
7.45
7.35
7.33
7.23
7.23
7.21
7.21
7.21
7.21
7.19
7.19
7.06
7.05
7.04
7.03
6.97
6.96
6.94
6.94
6.87
6.87
6.85
6.85
6.83
6.83
4.06
ppm
0.97
3.06
1.00
1.00
0.98
1.02
1.00
0.99
1.00
1.99

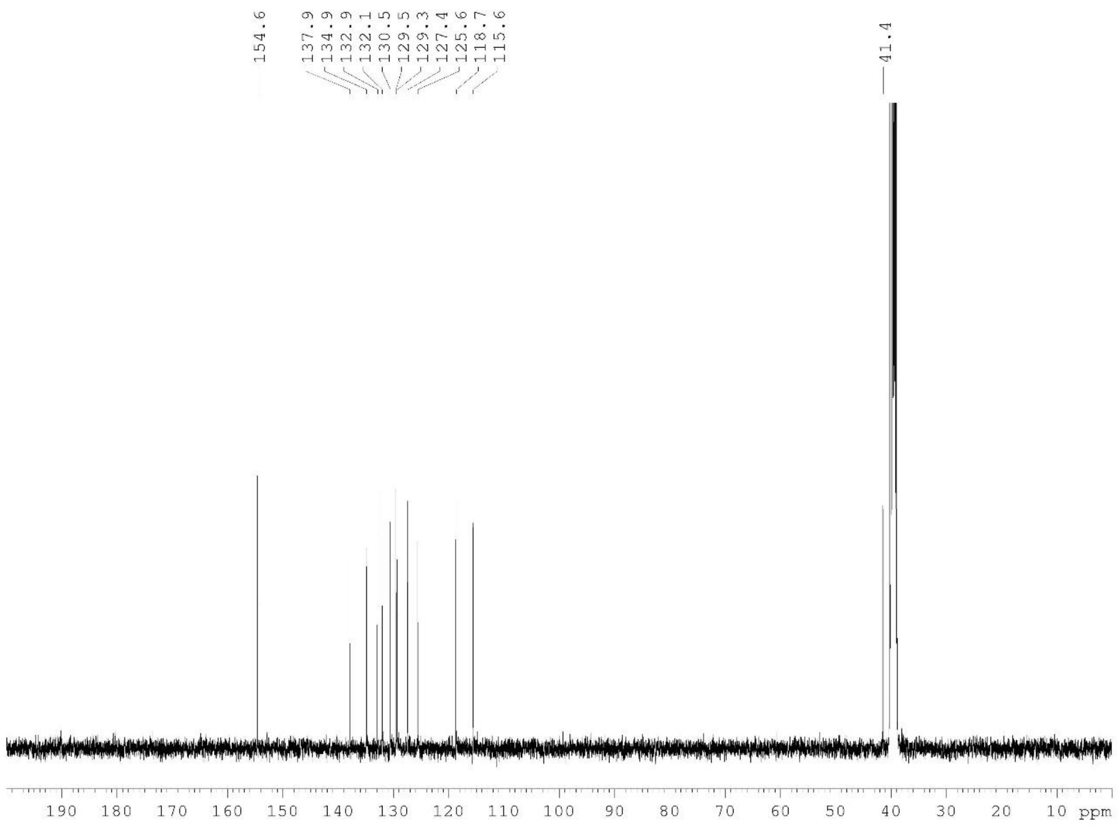
154.6
137.9
134.9
132.9
132.1
130.5
129.5
129.3
127.4
125.6
118.7
115.6
41.4
ppm

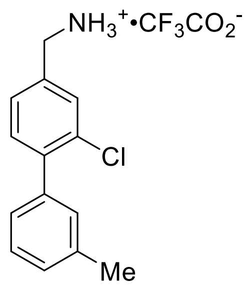
NH3+•CF3CO2-
Cl
Me

8

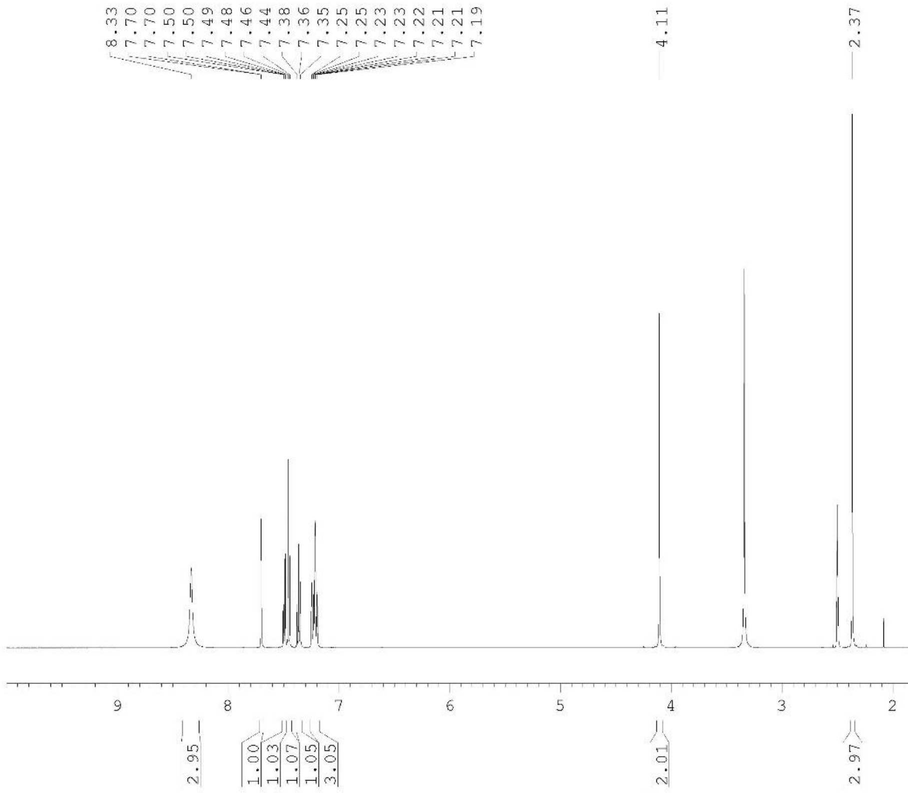
8.33 7.70 7.70 7.50 7.49 7.48 7.46 7.44 7.38 7.36 7.35 7.25 7.25 7.23 7.23 7.22 7.21 7.21 7.19
4.11
2.37
9 8 7 6 5 4 3 2 1 ppm
2.95
1.00 1.03 1.07 1.05 3.05
2.01
2.97

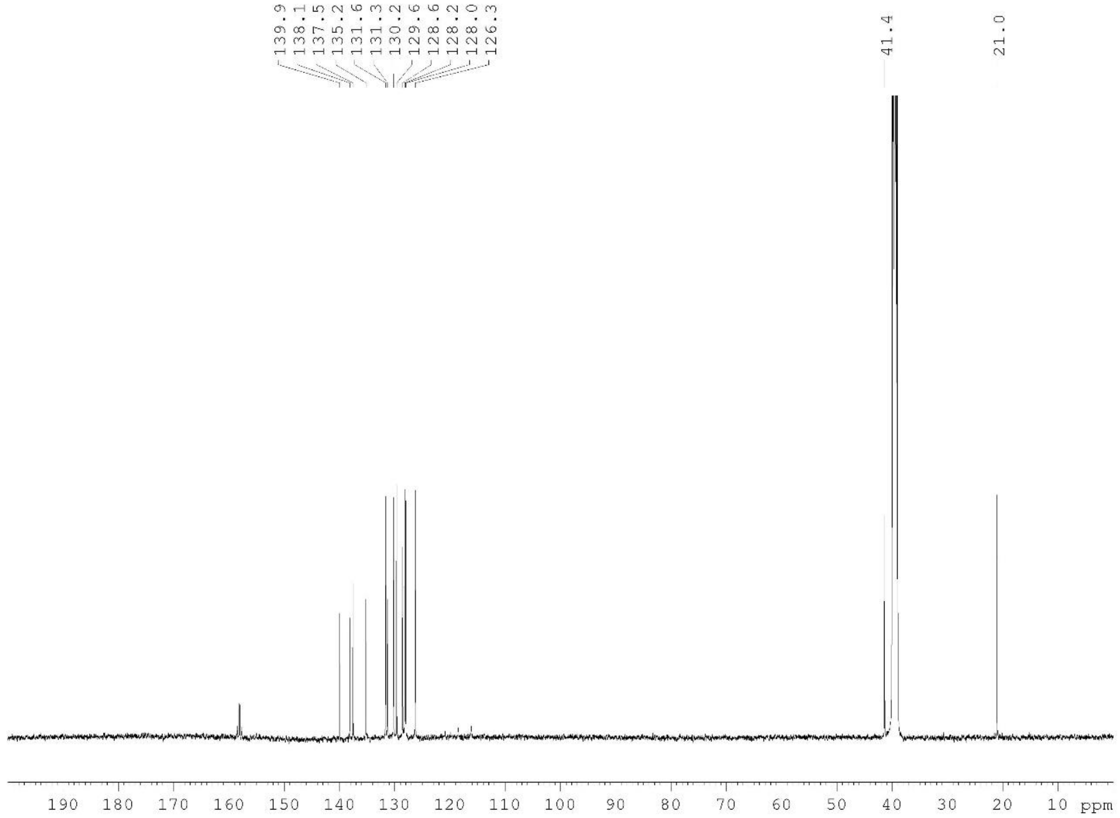
139.9 138.1 137.5 135.2 131.6 131.3 130.2 129.6 128.6 128.2 128.0 126.3
41.4
21.0
190 180 170 160 150 140 130 120 110 100 90 80 70 60 50 40 30 20 10 ppm

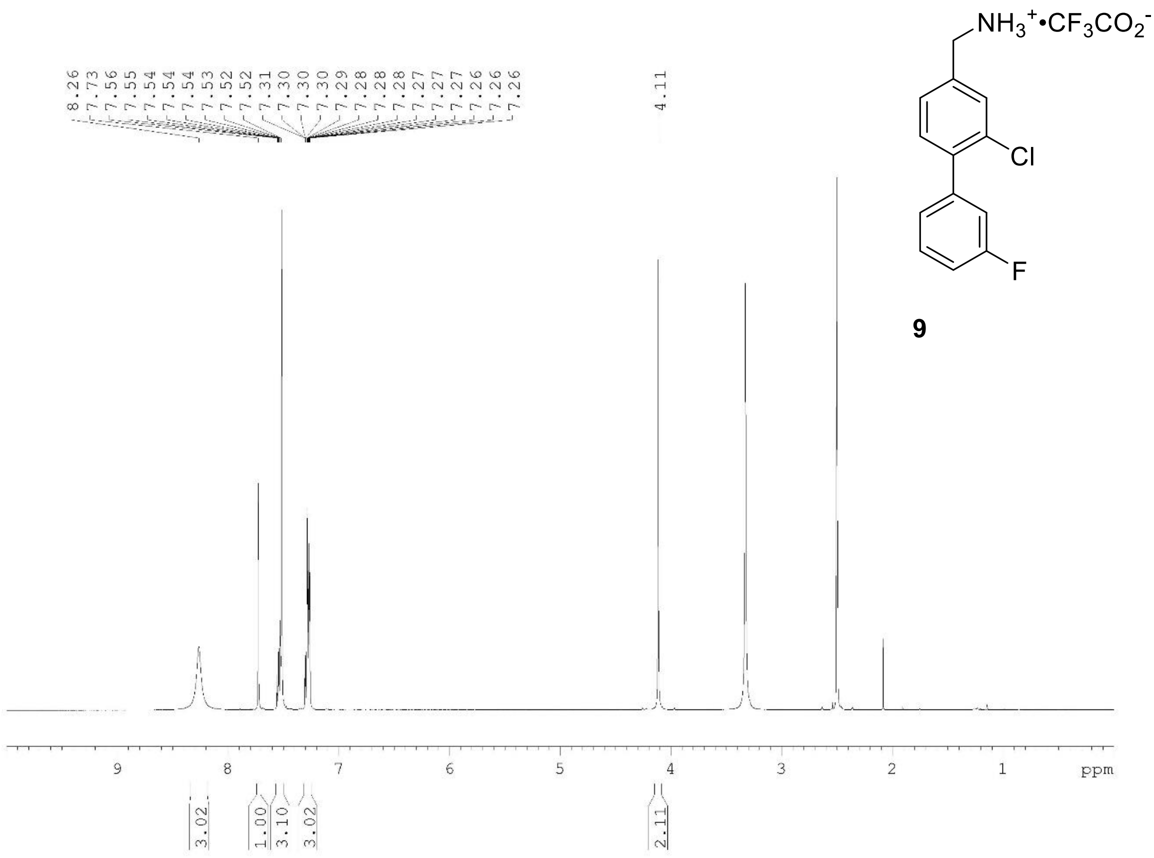
NH3+•CF3CO2-
Cl
F
8.26 7.73 7.56 7.55 7.54 7.54 7.53 7.52 7.52 7.31 7.30 7.30 7.30 7.29 7.28 7.28 7.28 7.27 7.27 7.27 7.26 7.26 7.26
4.11
9 8 7 6 5 4 3 2 1 ppm
3.02 1.00 3.10 3.02 2.11

9

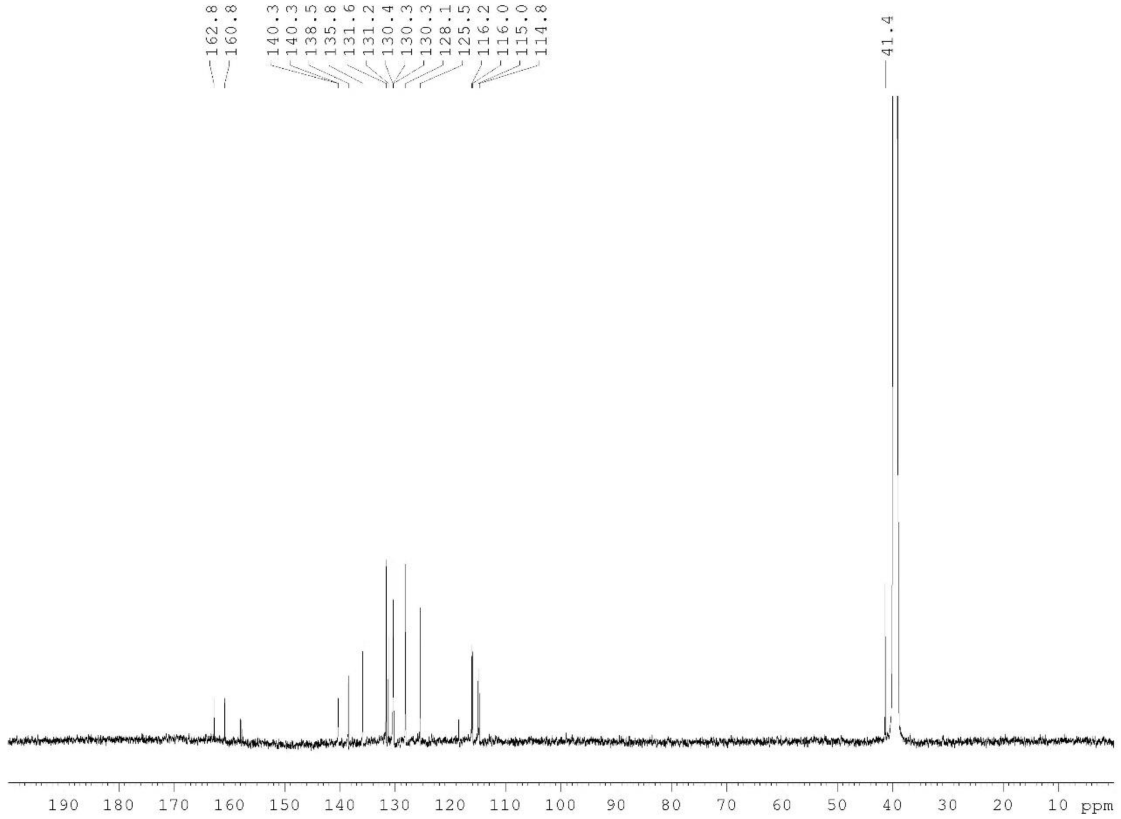
162.8 160.8 140.3 140.3 138.5 135.8 131.6 131.2 130.4 130.3 130.3 128.1 125.5 116.2 116.0 115.0 114.8
41.4
190 180 170 160 150 140 130 120 110 100 90 80 70 60 50 40 30 20 10 ppm

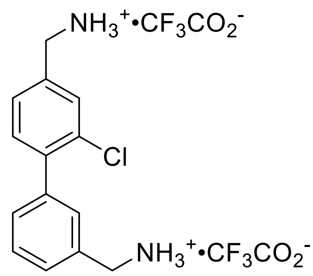
NH3+•CF3CO2-
Cl
NH3+•CF3CO2-

10

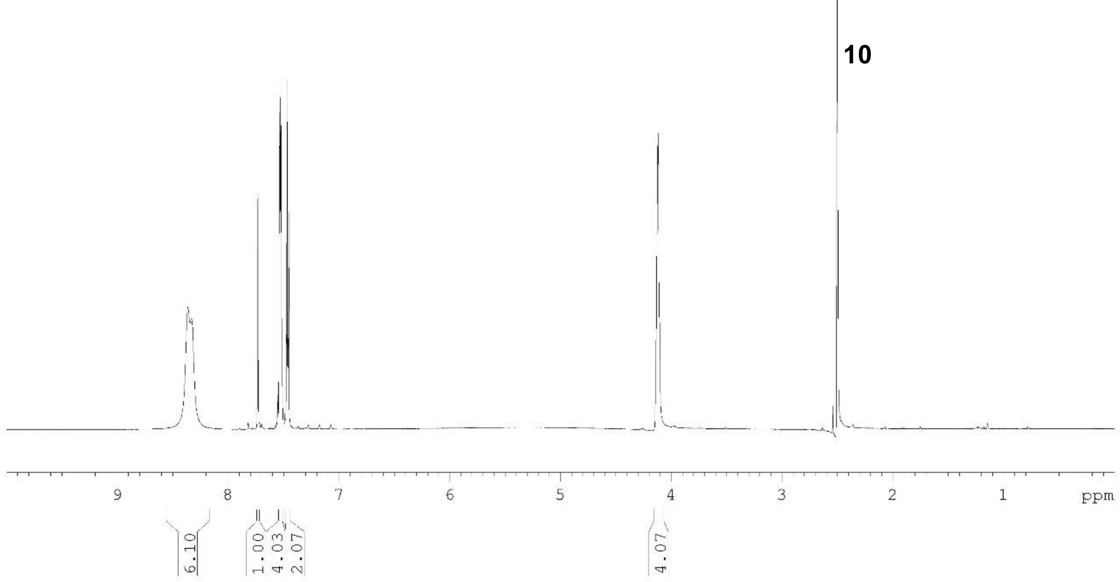
8.36
8.33
7.73
7.73
7.54
7.53
7.53
7.52
7.52
7.52
7.47
7.47
7.46
7.45
4.13
4.12
9
8
7
6
5
4
3
2
1
ppm
6.10
1.00
4.03
2.07
4.07

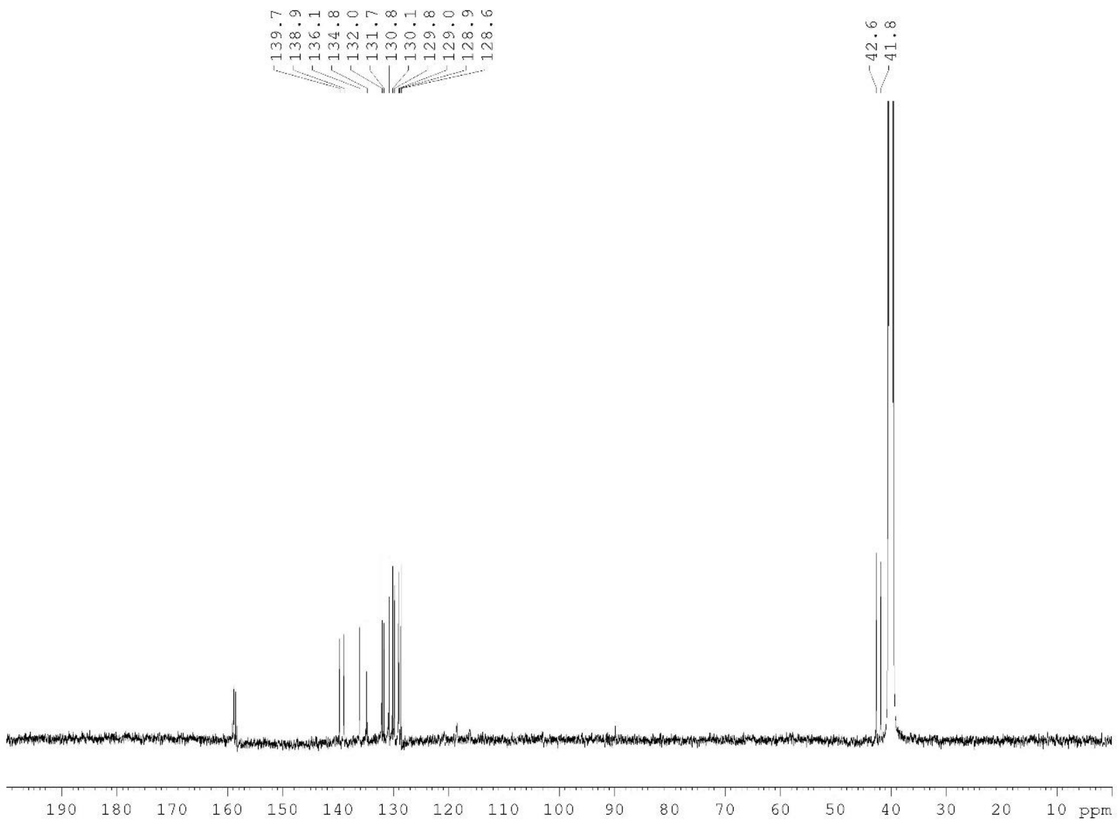
139.7
138.9
136.1
134.8
132.0
131.7
130.8
130.1
129.8
129.0
128.9
128.6
42.6
41.8
190
180
170
160
150
140
130
120
110
100
90
80
70
60
50
40
30
20
10
ppm

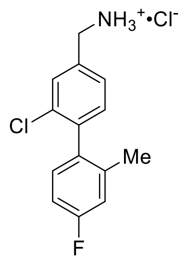
NH3+•Cl-
Cl
Me
F

11

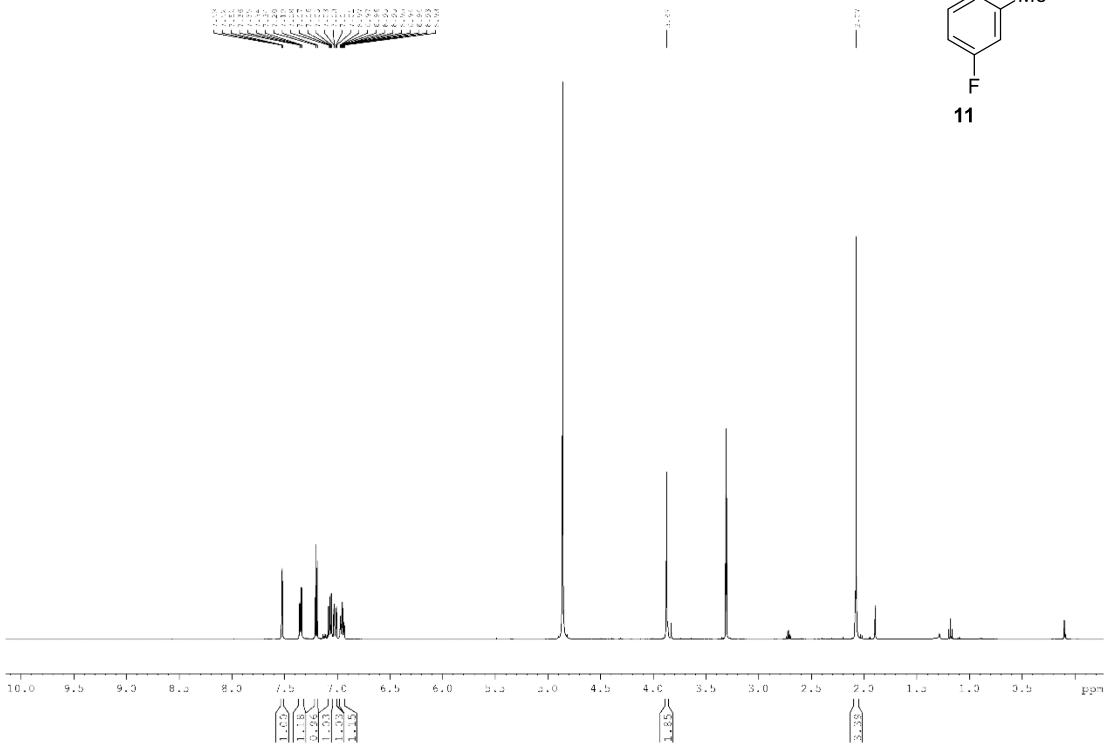
10.0
9.5
9.0
8.0
7.5
7.0
6.5
6.0
4.5
4.0
3.5
3.0
2.5
2.0
1.0
0.5
ppm
1.85

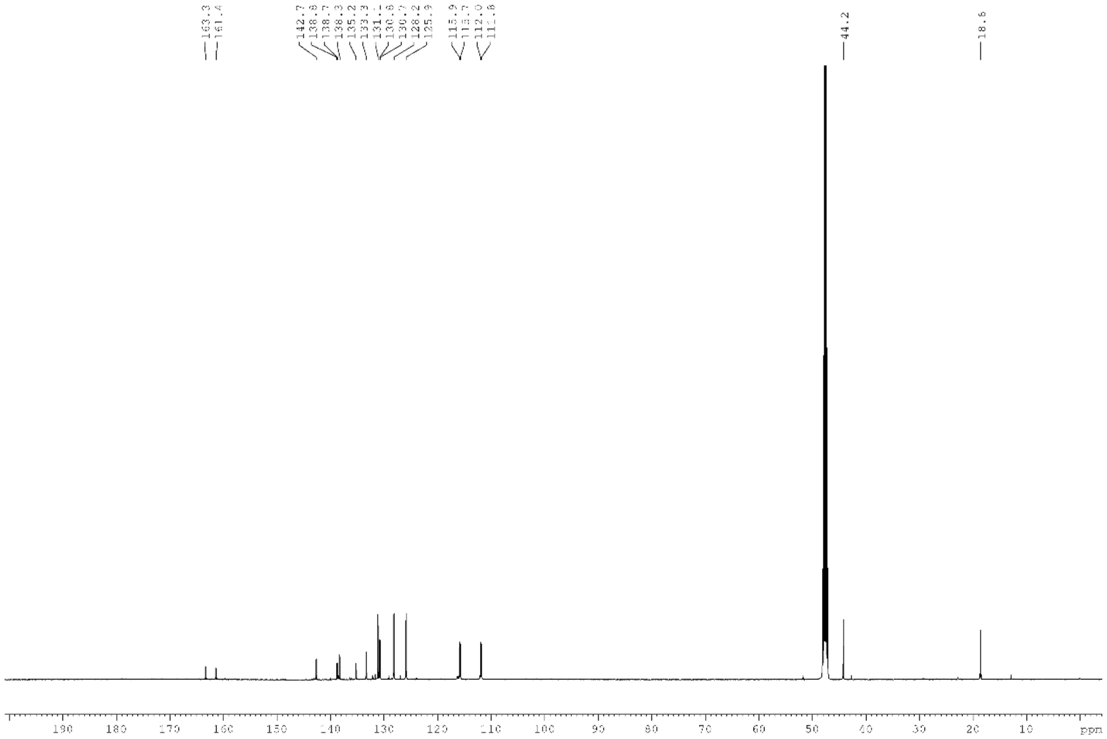
163.3
161.4
142.7
138.8
138.7
138.3
135.2
133.3
130.8
130.7
128.2
125.9
115.9
115.7
112.0
44.2
18.8
190
180
170
160
150
140
130
120
110
100
90
80
70
60
50
40
30
20
10
ppm

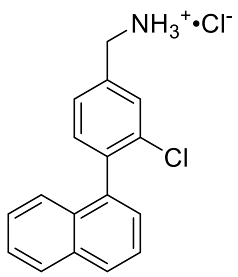
NH3+•Cl-
Cl

12

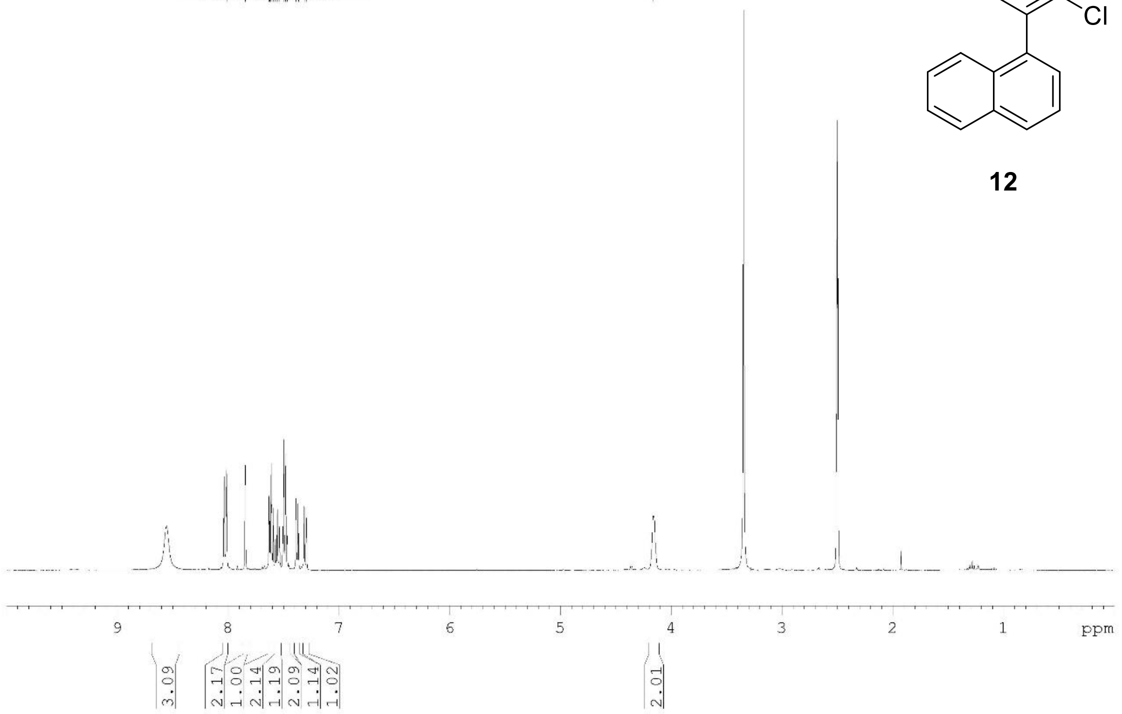
8.01 7.85 7.84 7.63 7.62 7.61 7.60 7.59 7.57 7.57 7.56 7.55 7.54 7.53 7.50 7.50 7.49 7.48 7.48 7.47 7.46 7.39 7.38 7.37 7.37 7.31 7.29
4.16 4.15
9 8 7 6 5 4 3 2 1 ppm
3.09 2.17 1.00 2.14 1.19 2.09 1.14 1.02 2.01

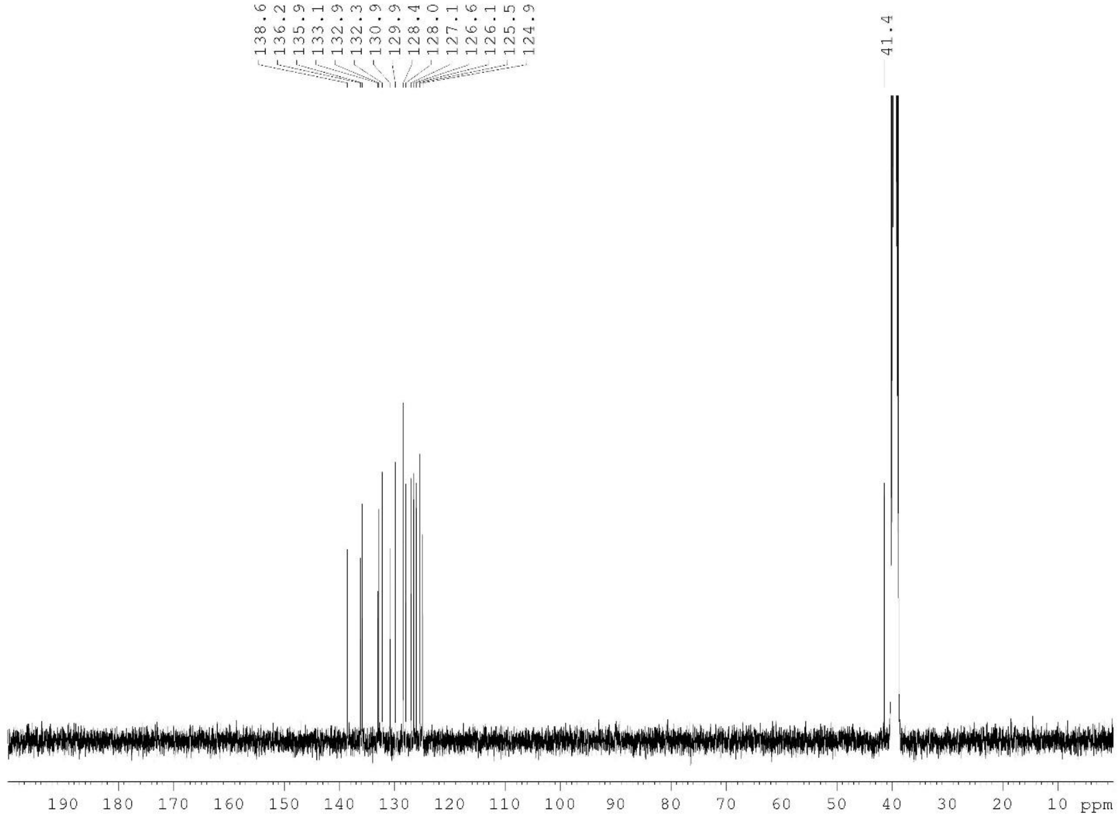
138.6 136.2 135.9 133.1 132.9 132.3 130.9 129.9 128.4 128.0 127.1 126.6 126.1 125.5 124.9
41.4
190 180 170 160 150 140 130 120 110 100 90 80 70 60 50 40 30 20 10 ppm

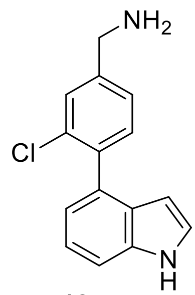
NH$_2$
Cl
N
H

13

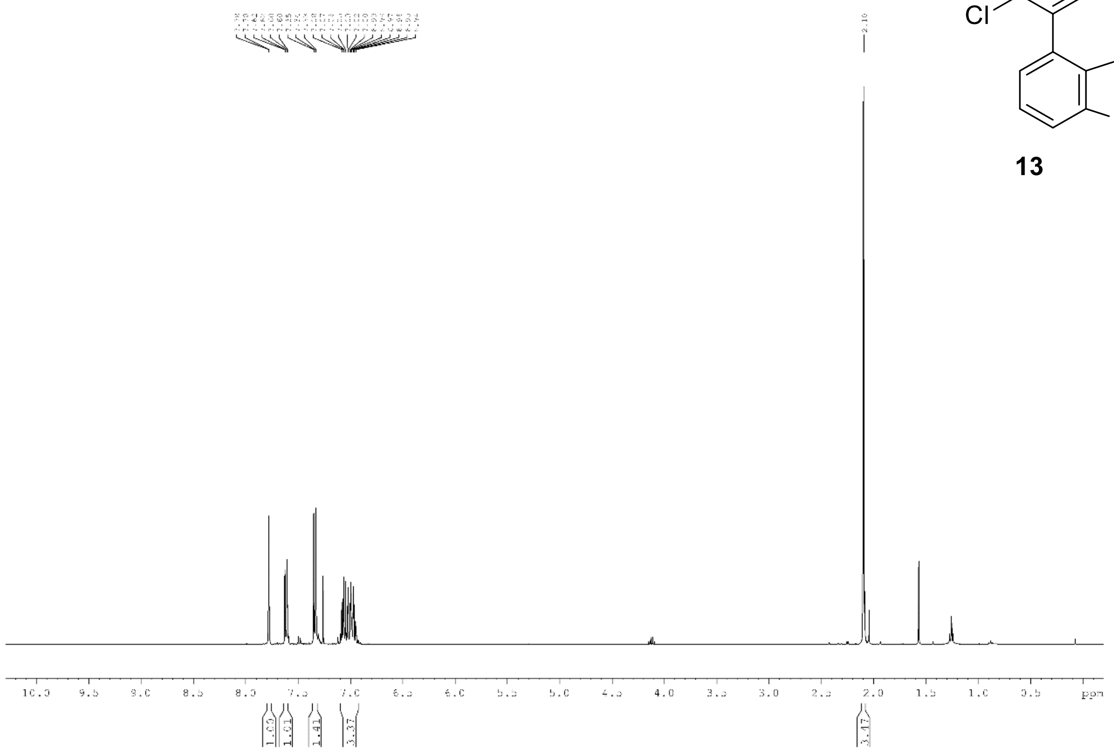
2.10
ppm
3.47

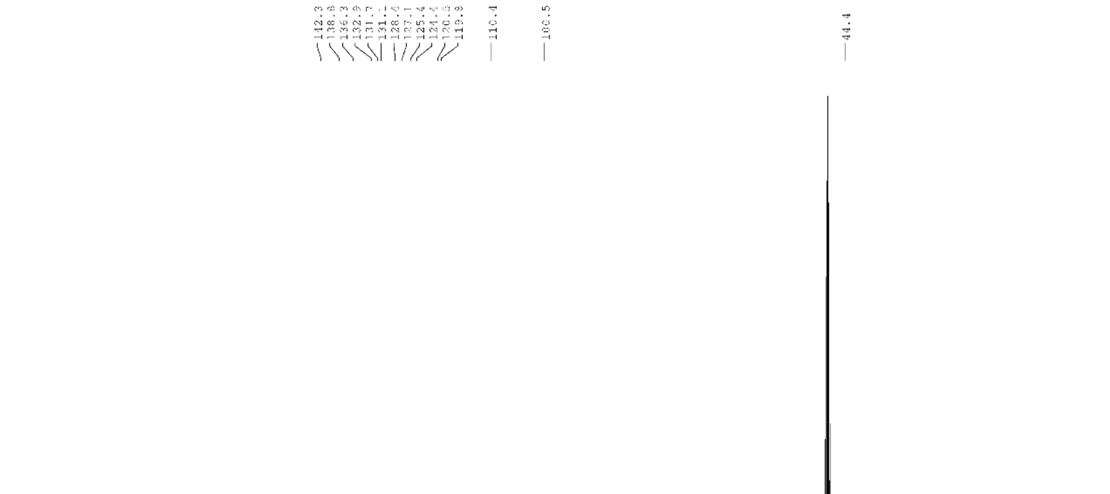
142.3
138.8
136.3
132.9
131.7
131.1
128.4
127.1
125.4
124.4
120.6
119.8
110.4
100.5
44.4
ppm

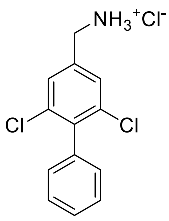

14

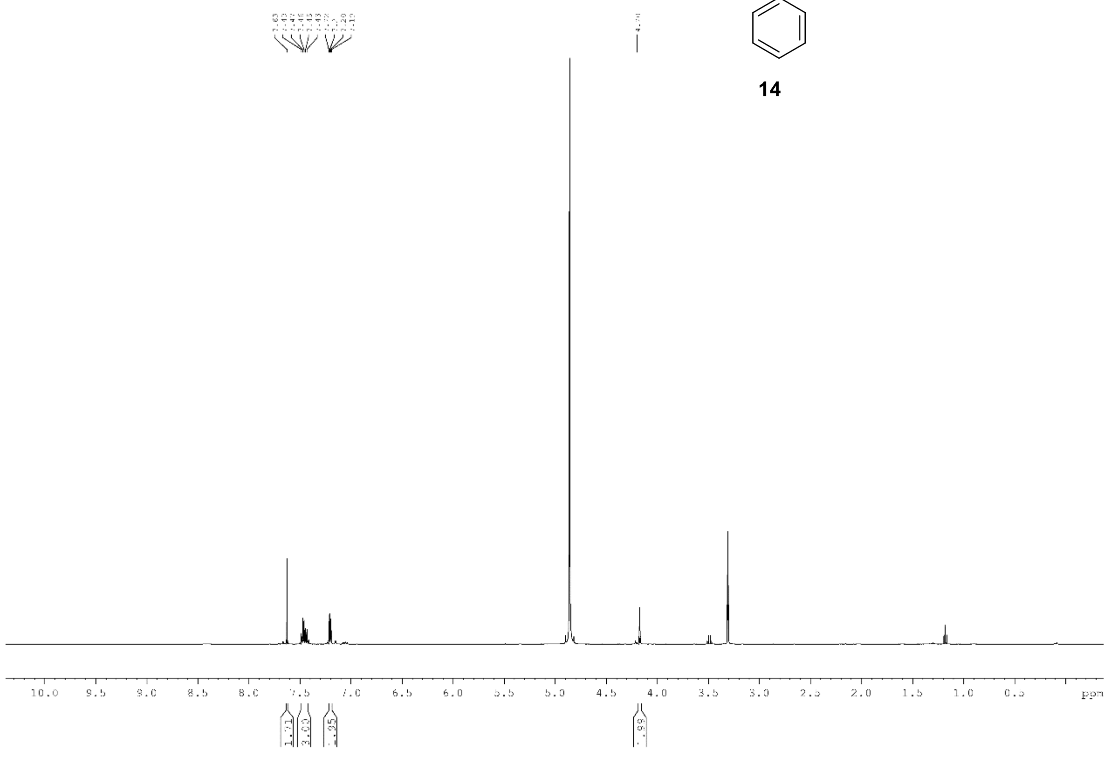

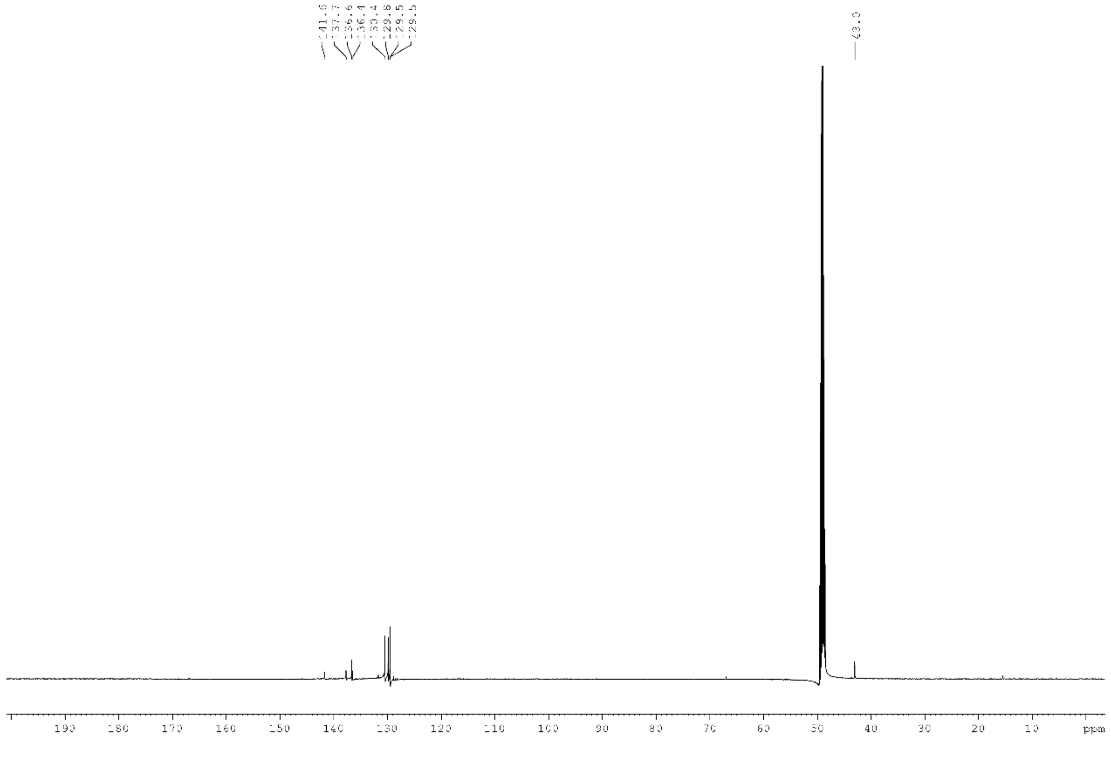

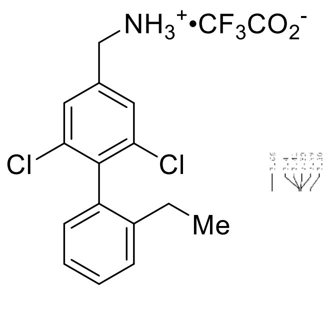
NH3+•CF3CO2-
Cl
Cl
Me

15

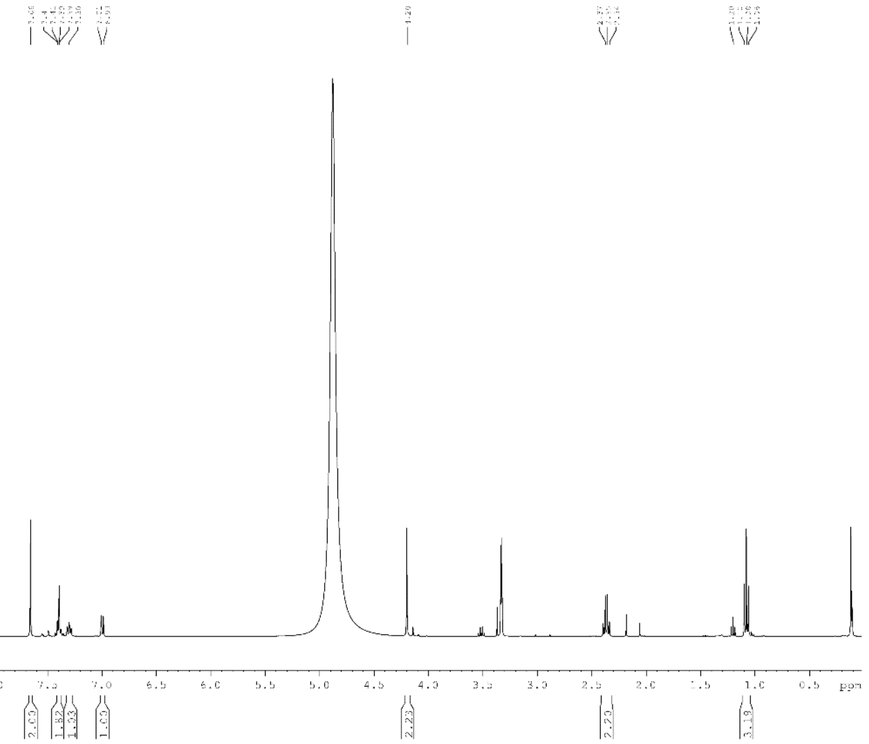
9.5 9.0 8.5 8.0 7.5 7.0 6.5 6.0 5.5 5.0 4.5 4.0 3.5 3.0 2.5 2.0 1.5 1.0 0.5 ppm

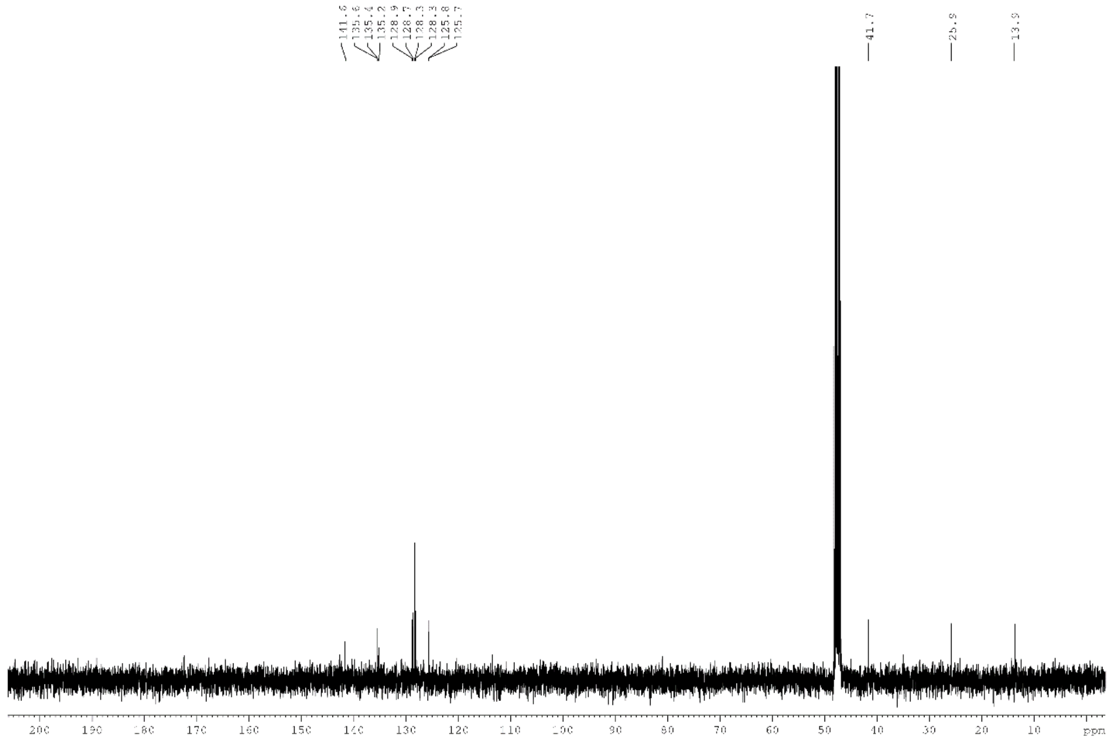
141.6
41.7
25.9
13.9
200 190 180 170 160 150 140 130 120 110 100 90 80 70 60 50 40 30 20 10 ppm

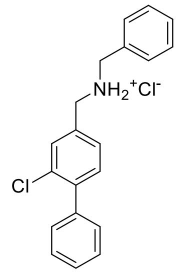
NH2+Cl-
Cl

16

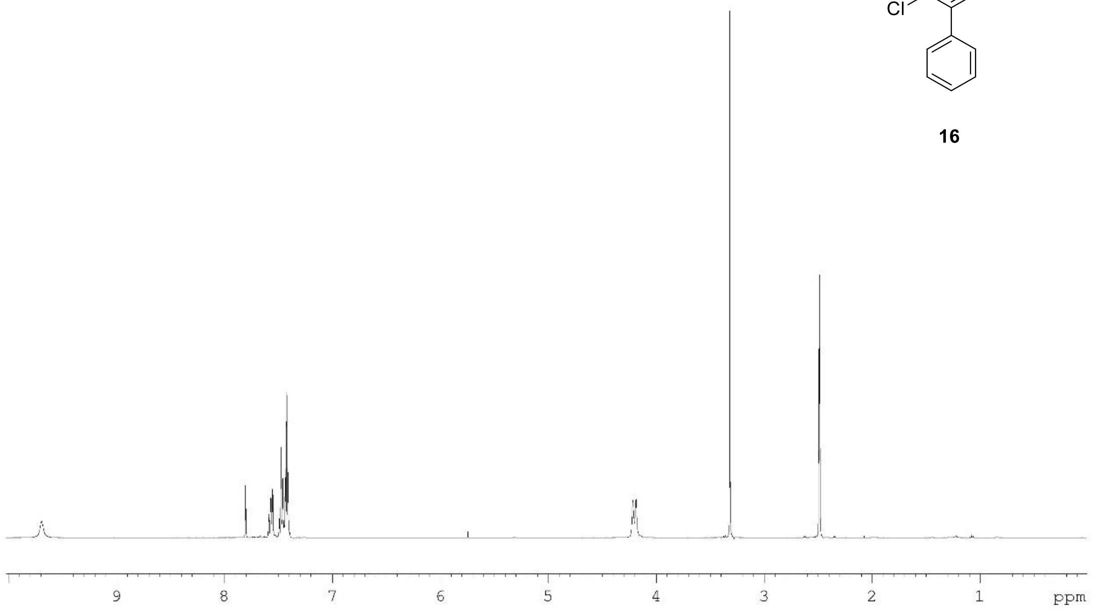
9 8 7 6 5 4 3 2 1 ppm

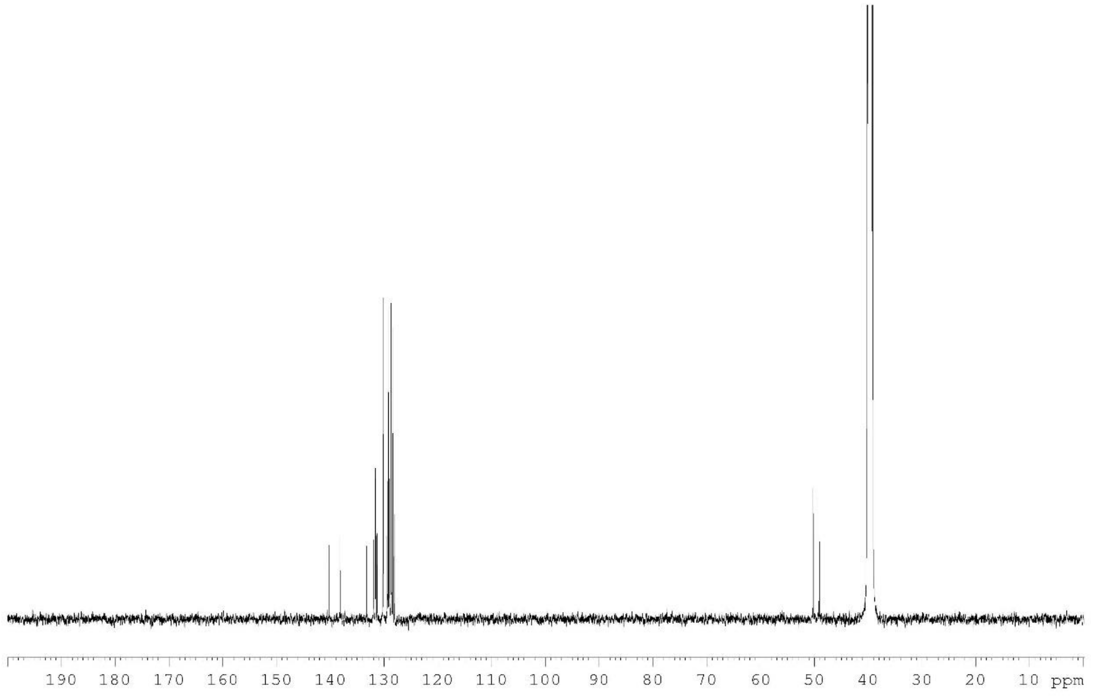
190 180 170 160 150 140 130 120 110 100 90 80 70 60 50 40 30 20 10 ppm

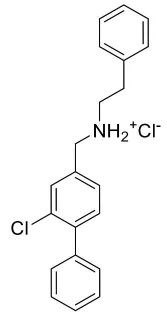
NH2+Cl-
Cl

17

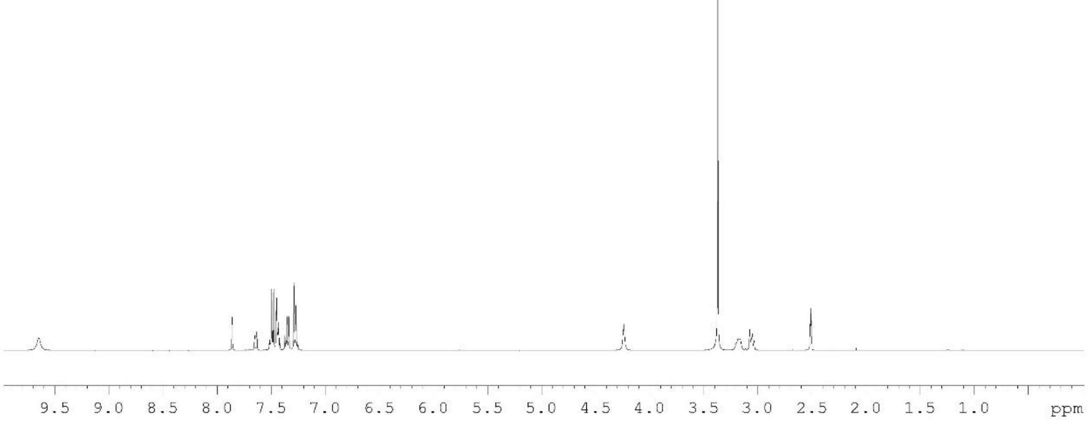
9.5 9.0 8.5 8.0 7.5 7.0 6.5 6.0 5.5 5.0 4.5 4.0 3.5 3.0 2.5 2.0 1.5 1.0 ppm

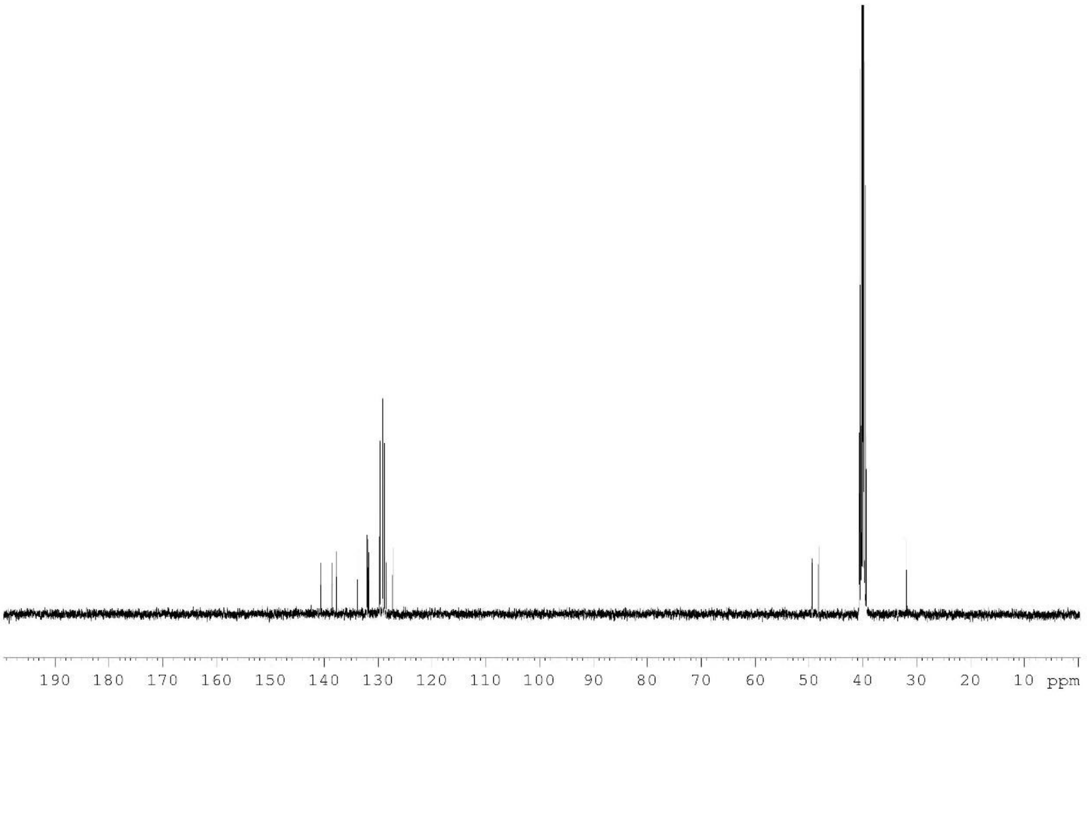
190 180 170 160 150 140 130 120 110 100 90 80 70 60 50 40 30 20 10 ppm

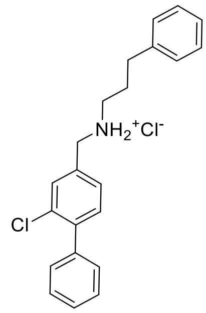
NH2+Cl-
Cl

18

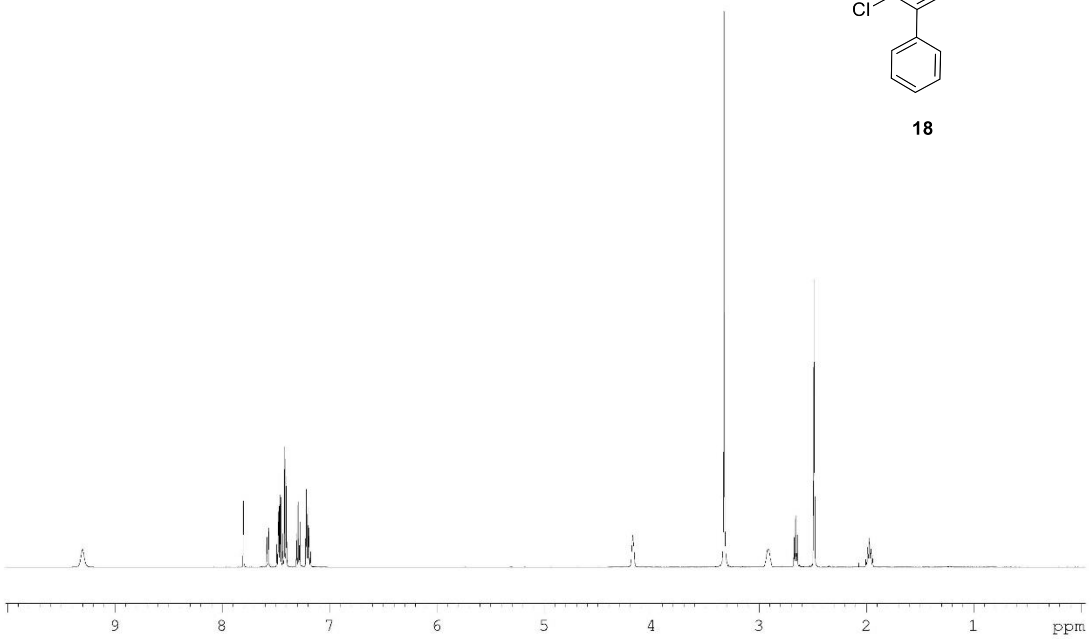
9 8 7 6 5 4 3 2 1 ppm

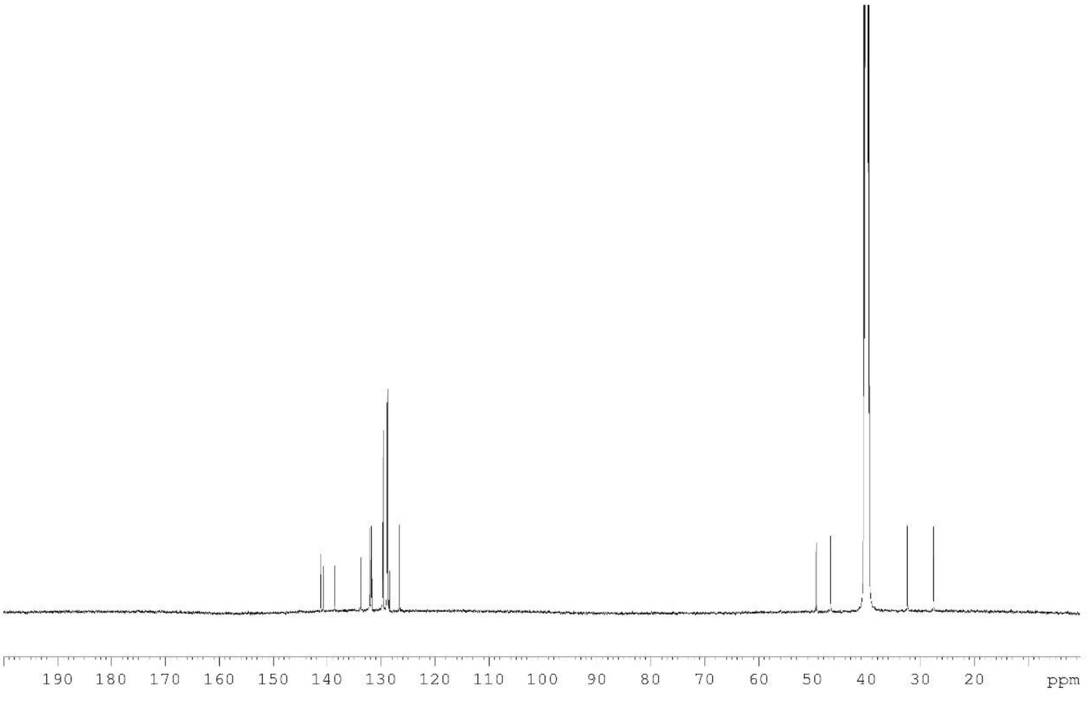
190 180 170 160 150 140 130 120 110 100 90 80 70 60 50 40 30 20 ppm

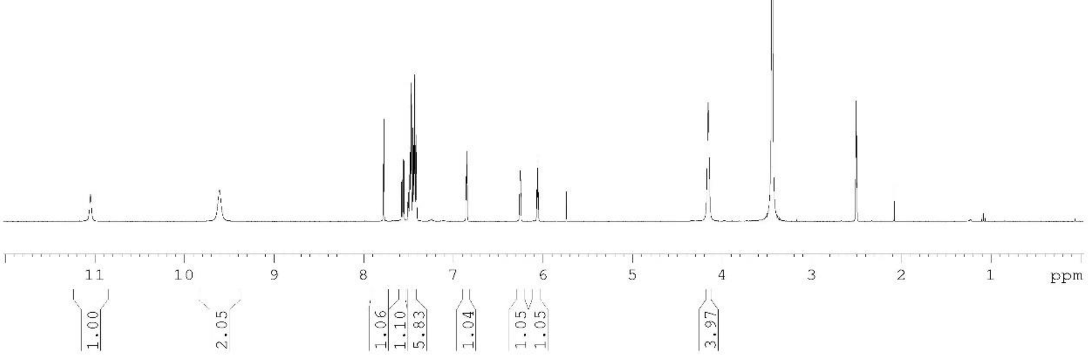
11.05 9.61 7.78 7.77 7.57 7.57 7.55 7.55 7.50 7.50 7.49 7.48 7.48 7.47 7.47 7.47 7.45 7.45 7.44 7.44 7.44 7.43 7.43 7.42 7.42 7.41 7.41 7.41 6.86 6.85 6.85 6.85 6.84 6.84 6.26 6.25 6.24 6.07 6.06 6.05 6.05 5.74 4.15
11 10 9 8 7 6 5 4 3 2 1 ppm
1.00 2.05 1.06 1.10 5.83 1.04 1.05 1.05 3.97

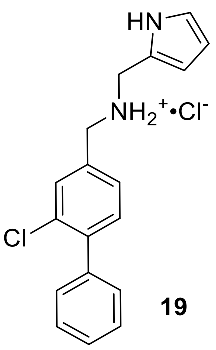
HN
NH2+•Cl-
Cl

19

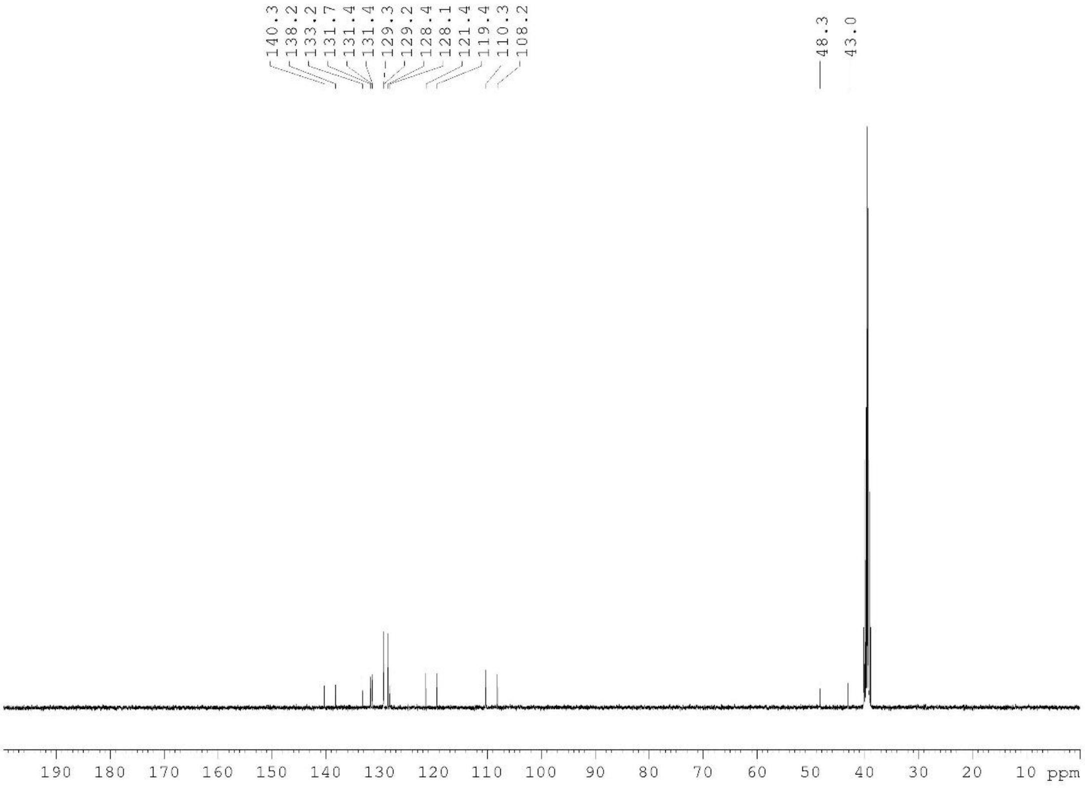
140.3 138.2 133.2 131.7 131.4 131.4 129.3 129.2 128.4 128.1 121.4 119.4 110.3 108.2
48.3 43.0
190 180 170 160 150 140 130 120 110 100 90 80 70 60 50 40 30 20 10 ppm

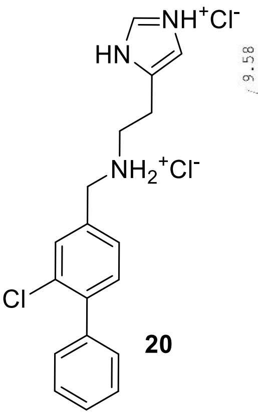

NH+Cl-
HN
NH2+Cl-
Cl

20

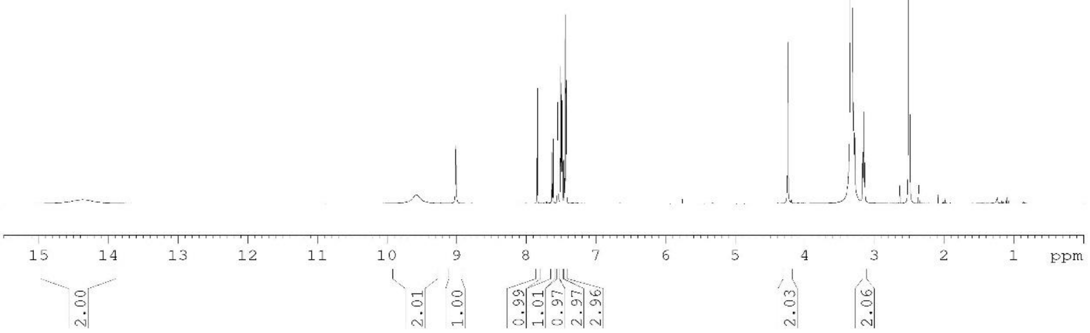

14.36
9.58
9.01
7.84
7.84
7.63
7.63
7.61
7.61
7.55
7.51
7.50
7.49
7.49
7.48
7.45
7.45
7.44
7.43
7.42
7.42
4.24
3.17
3.15
15 14 13 12 11 10 9 8 7 6 5 4 3 2 1 ppm
2.00
2.01
1.00
0.99
1.01
0.97
2.97
2.96
2.03
2.06

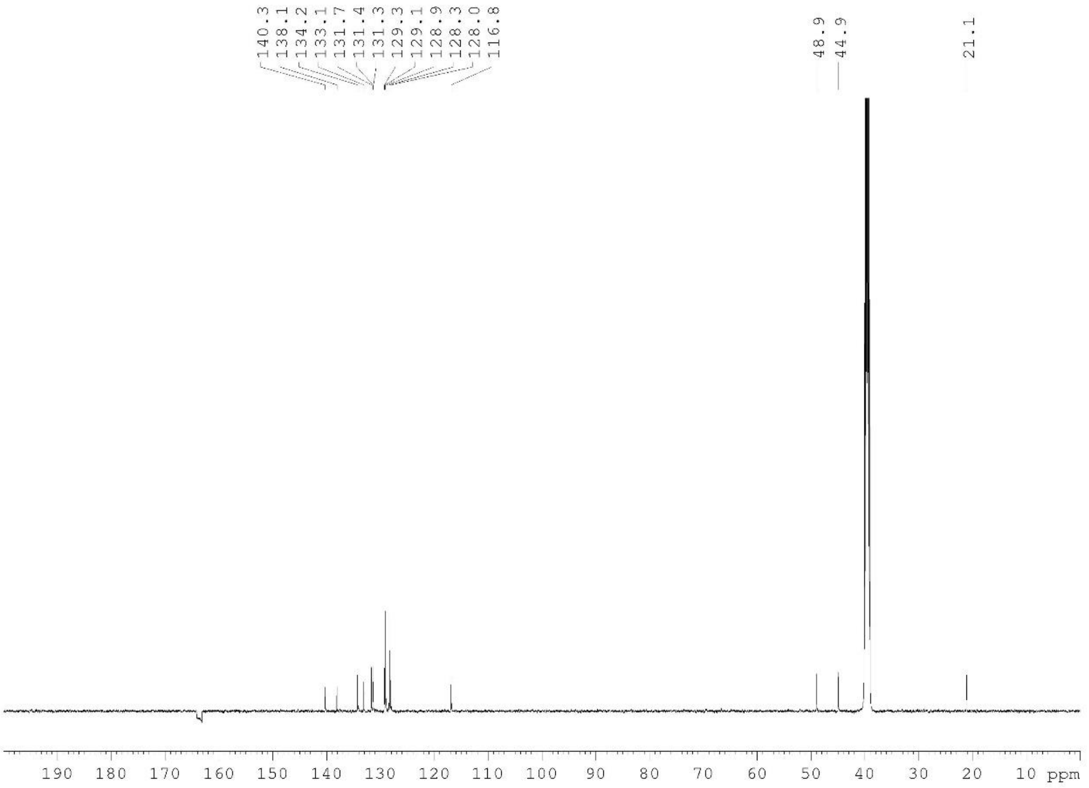

140.3
138.1
134.2
133.1
131.7
131.4
131.3
129.3
129.1
128.9
128.3
128.0
116.8
48.9
44.9
21.1
190 180 170 160 150 140 130 120 110 100 90 80 70 60 50 40 30 20 10 ppm

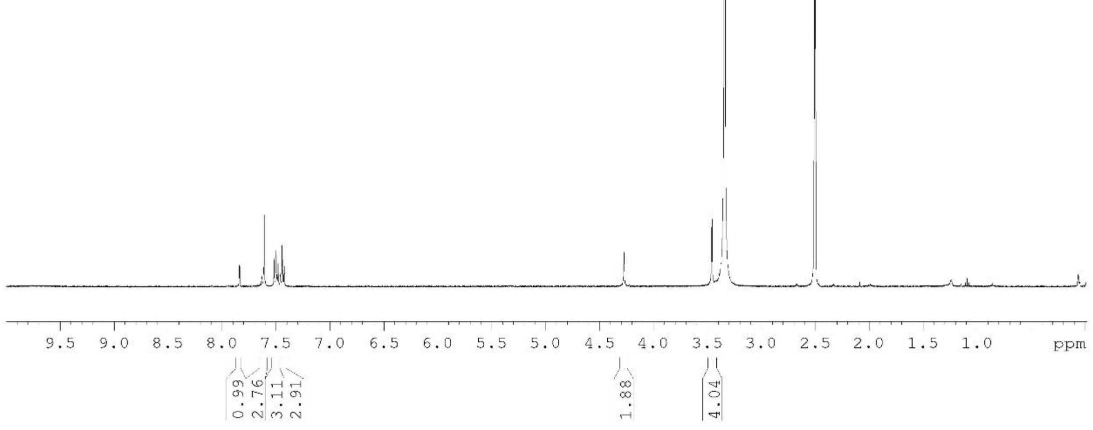
7.84 7.83 7.63 7.63 7.61 7.61 7.52 7.50 7.48 7.44 7.44 7.44 7.42
4.27
3.46
9.5 9.0 8.5 8.0 7.5 7.0 6.5 6.0 5.5 5.0 4.5 4.0 3.5 3.0 2.5 2.0 1.5 1.0 ppm
0.99 2.76 3.11 2.91
1.88
4.04

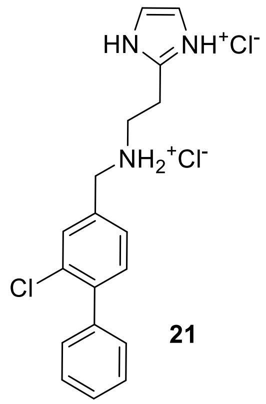
HN
NH+Cl-
NH2+Cl-
Cl

21

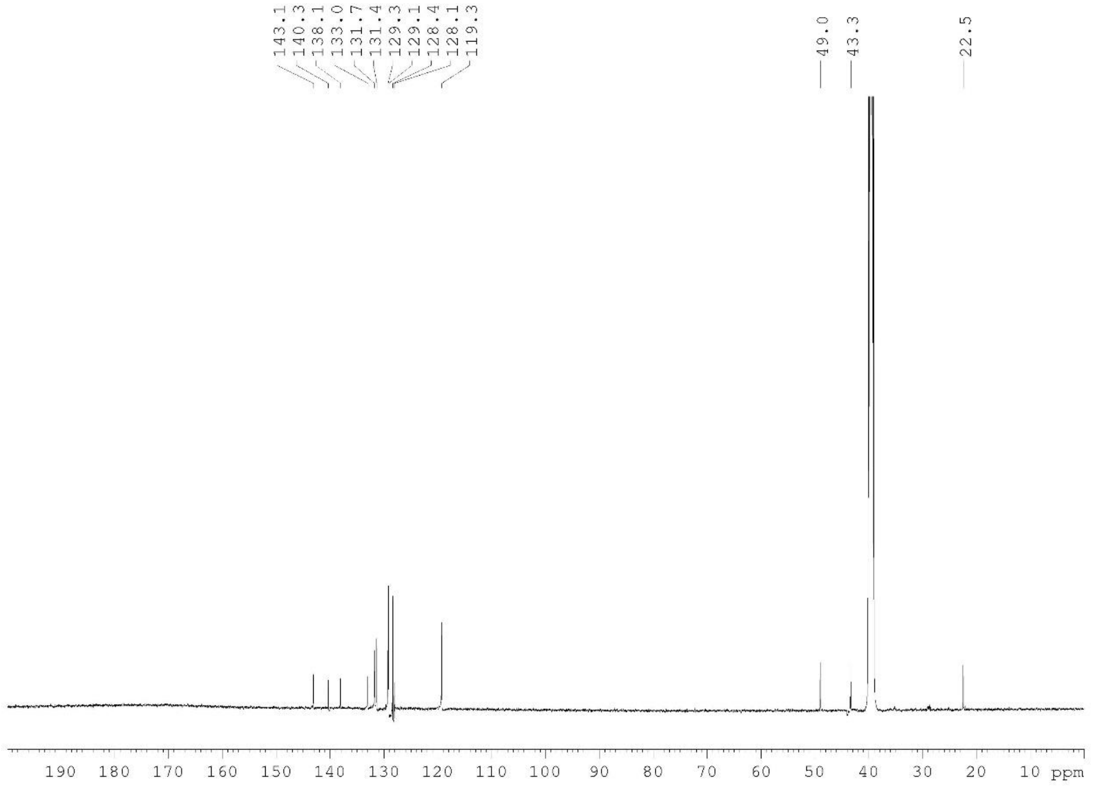
143.1 140.3 138.1 133.0 131.7 131.4 129.3 129.1 128.4 128.1 119.3
49.0
43.3
22.5
190 180 170 160 150 140 130 120 110 100 90 80 70 60 50 40 30 20 10 ppm

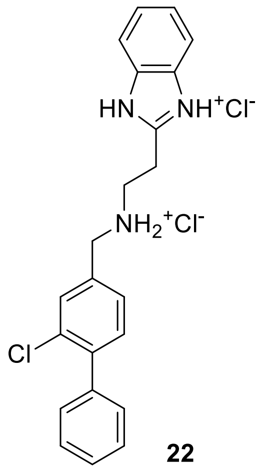
HN
NH+Cl-
NH2+Cl-
Cl

22

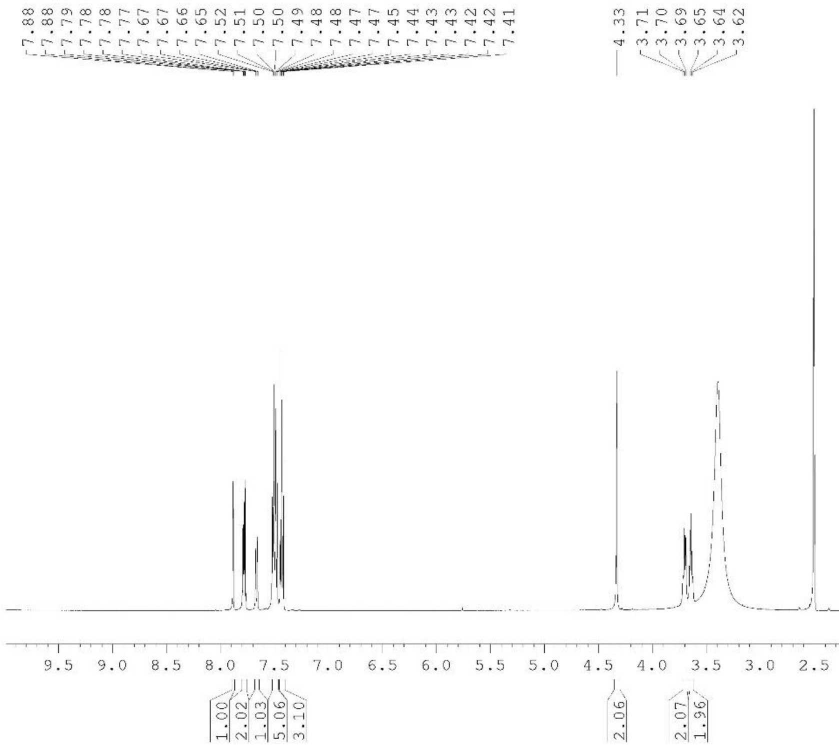
7.88 7.88 7.79 7.78 7.78 7.77 7.67 7.67 7.66 7.65 7.52 7.51 7.50 7.50 7.49 7.48 7.48 7.47 7.47 7.45 7.44 7.43 7.43 7.42 7.42 7.41
4.33 3.71 3.70 3.69 3.65 3.64 3.62
9.5 9.0 8.5 8.0 7.5 7.0 6.5 6.0 5.5 5.0 4.5 4.0 3.5 3.0 2.5 2.0 1.5 1.0 ppm
1.00 2.02 1.03 5.06 3.10 2.06 2.07 1.96

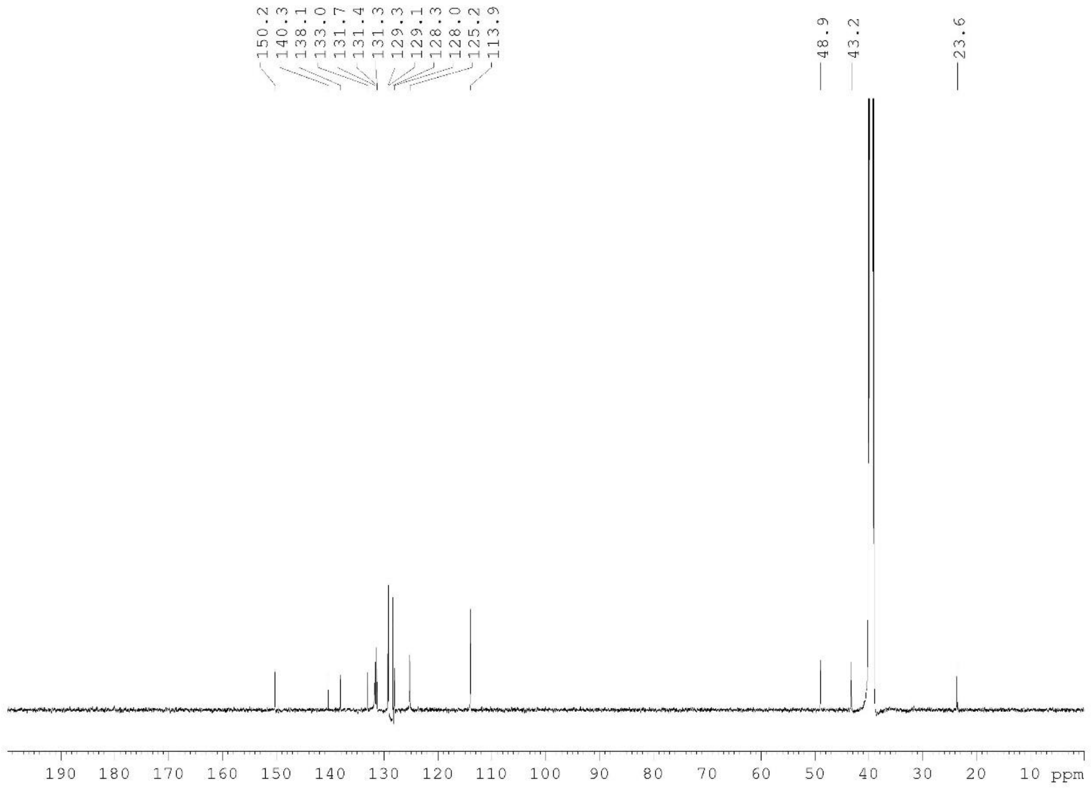
150.2 140.3 138.1 133.0 131.7 131.4 131.3 129.3 129.1 128.3 128.0 125.2 113.9
48.9 43.2 23.6
190 180 170 160 150 140 130 120 110 100 90 80 70 60 50 40 30 20 10 ppm

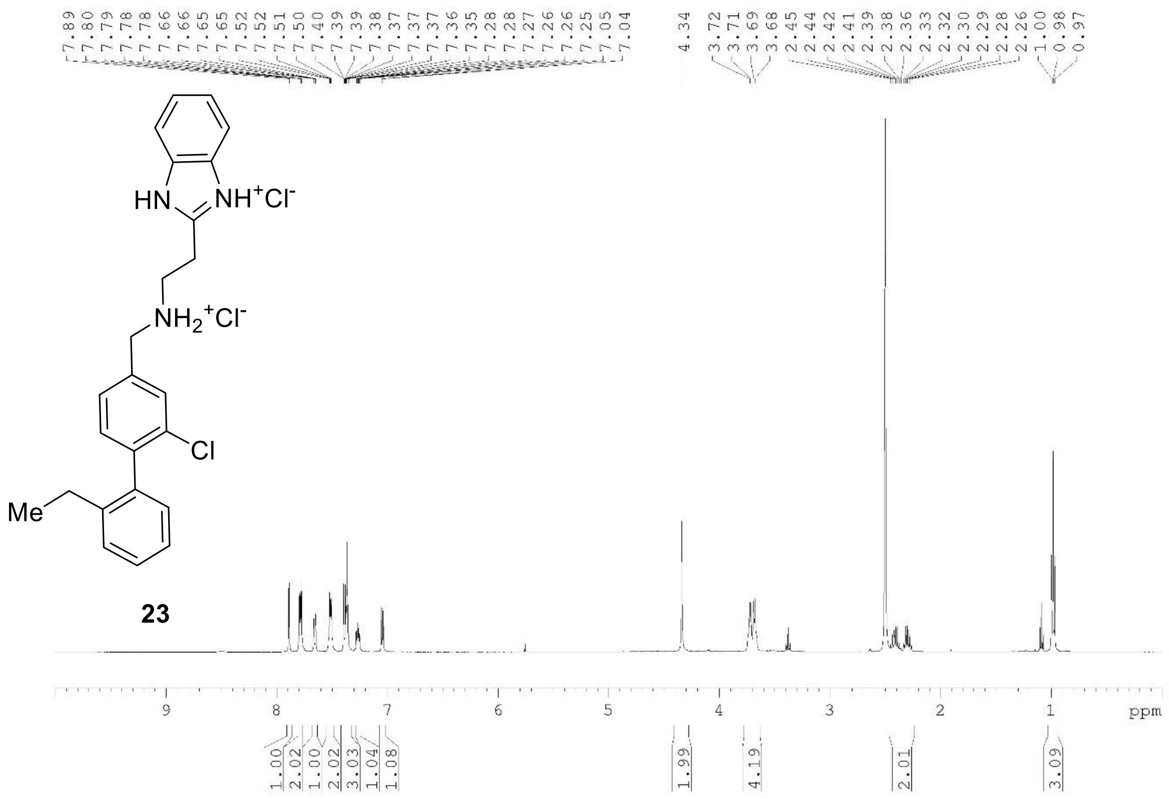
7.89 7.80 7.79 7.78 7.78 7.66 7.66 7.65 7.65 7.52 7.52 7.51 7.50 7.40 7.39 7.39 7.38 7.37 7.37 7.37 7.36 7.35 7.28 7.28 7.27 7.26 7.26 7.25 7.05 7.04
4.34 3.72 3.71 3.69 3.68 2.45 2.44 2.42 2.41 2.39 2.38 2.36 2.33 2.32 2.30 2.29 2.28 2.26 1.00 0.98 0.97
HN
NH+Cl-
NH2+Cl-
Cl
Me
23
9 8 7 6 5 4 3 2 1 ppm
1.00 2.02 1.00 2.02 3.03 1.04 1.08 1.99 4.19 2.01 3.09

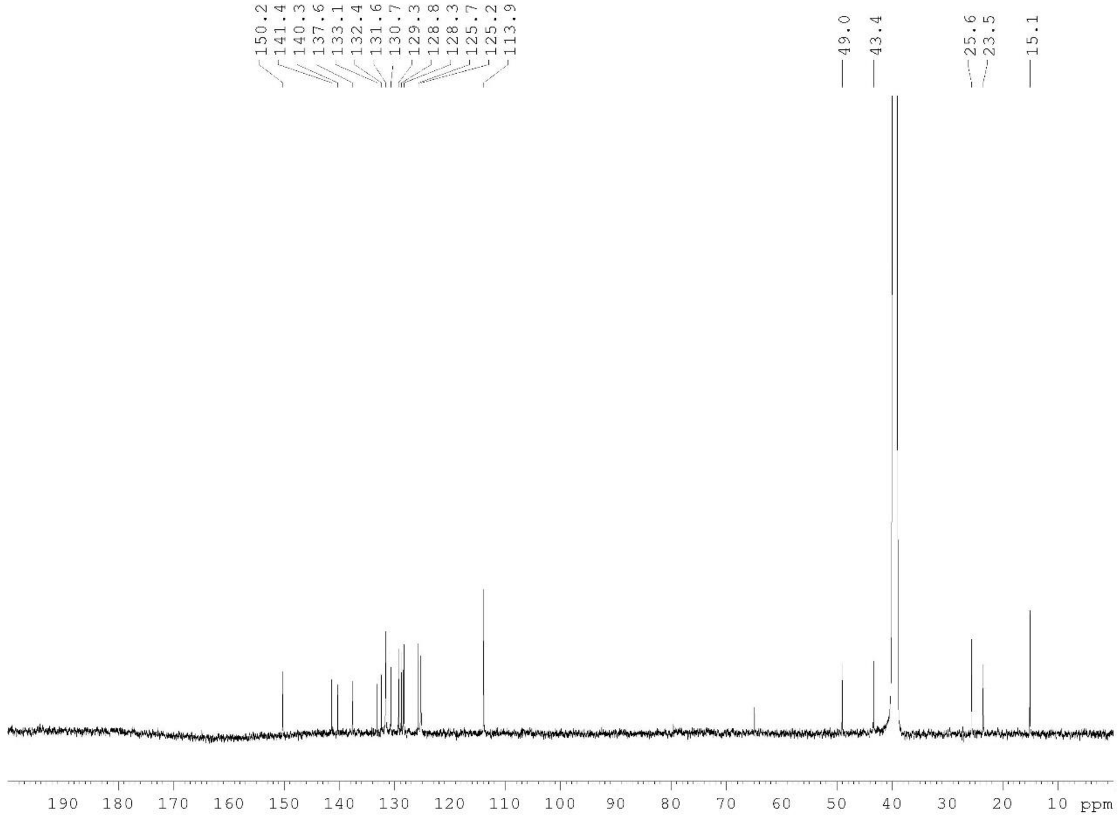
150.2 141.4 140.3 137.6 133.1 132.4 131.6 130.7 129.3 128.8 128.3 125.7 125.2 113.9
49.0 43.4 25.6 23.5 15.1
190 180 170 160 150 140 130 120 110 100 90 80 70 60 50 40 30 20 10 ppm

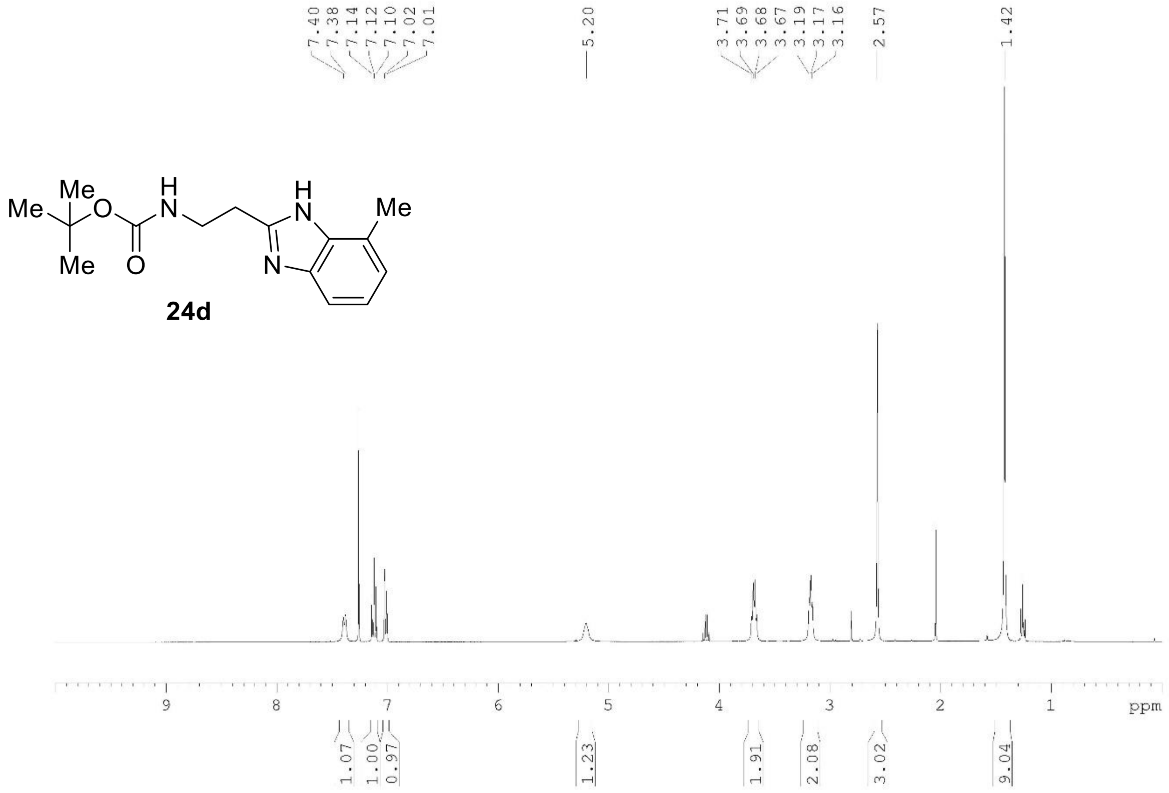
7.40
7.38
7.14
7.12
7.10
7.02
7.01
5.20
3.71
3.69
3.68
3.67
3.19
3.17
3.16
2.57
1.42
Me
Me
O
H
N
H
N
Me
Me
O
N
24d
9 8 7 6 5 4 3 2 1 ppm
1.07
1.00
0.97
1.23
1.91
2.08
3.02
9.04

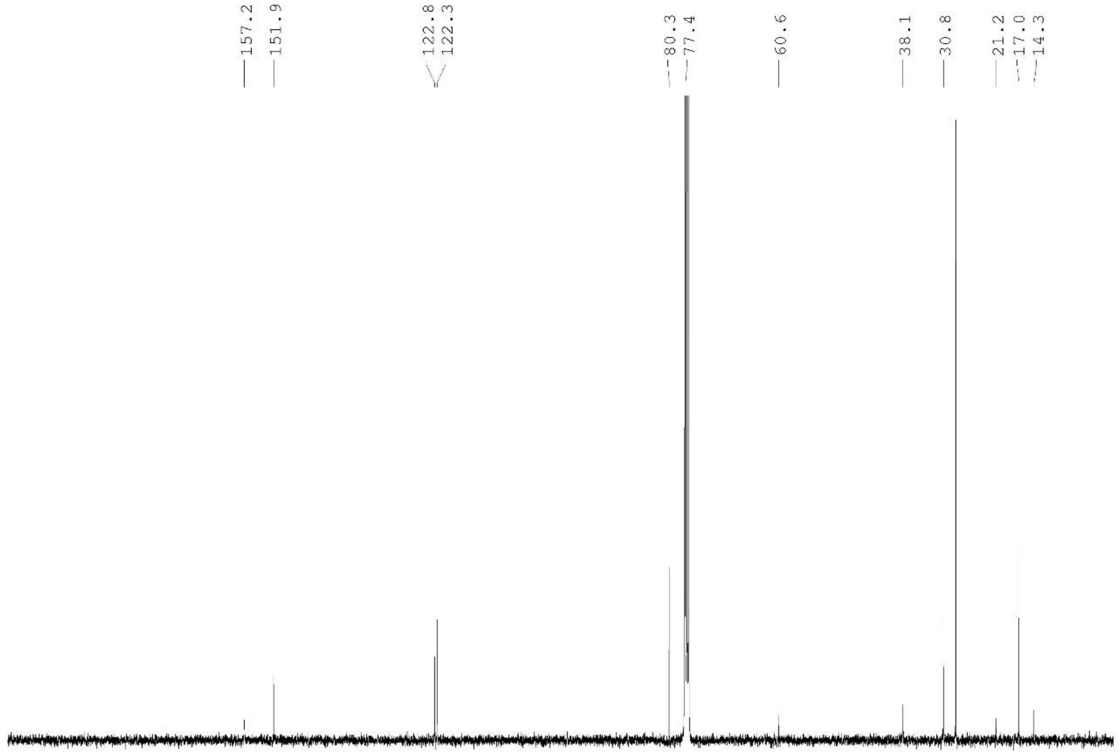
157.2
151.9
122.8
122.3
80.3
77.4
60.6
38.1
30.8
21.2
17.0
14.3
190 180 170 160 150 140 130 120 110 100 90 80 70 60 50 40 30 20 10 ppm

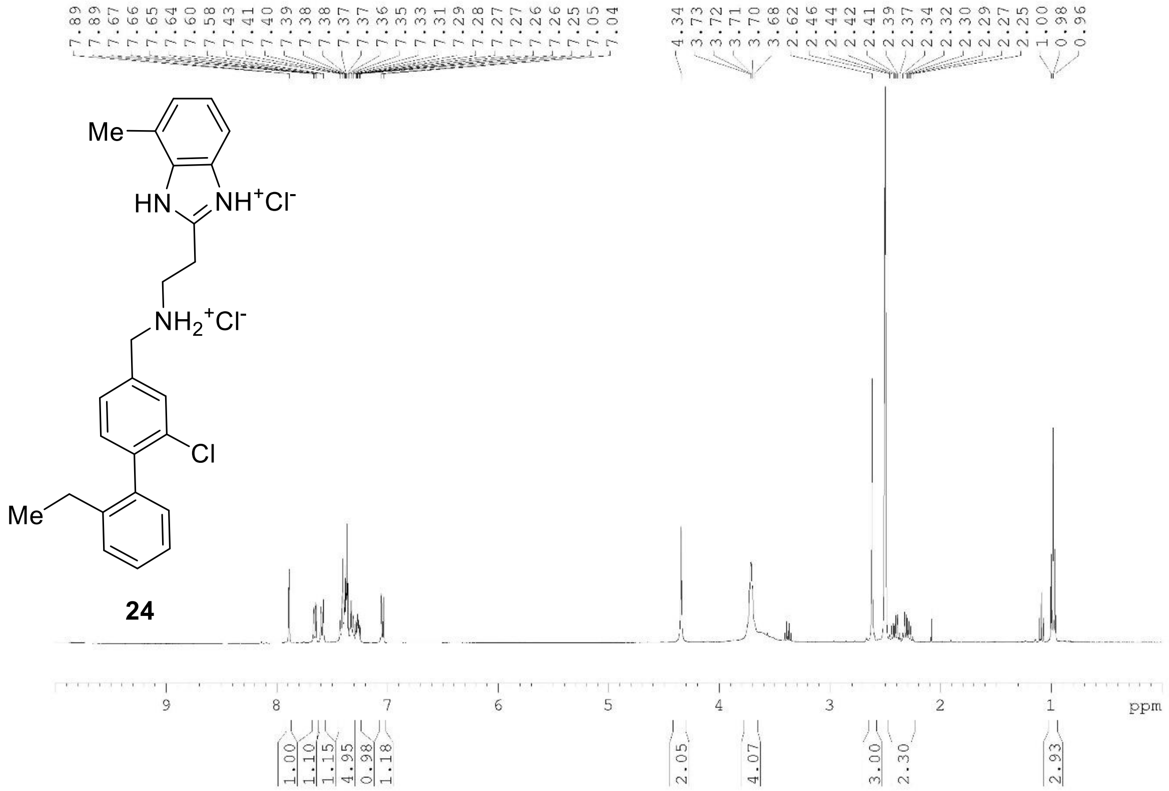
7.89 7.89 7.67 7.66 7.65 7.64 7.60 7.58 7.43 7.41 7.40 7.39 7.38 7.38 7.37 7.37 7.36 7.35 7.33 7.31 7.29 7.28 7.27 7.27 7.26 7.26 7.25 7.05 7.04
4.34 3.73 3.72 3.71 3.70 3.68 2.62 2.46 2.44 2.42 2.41 2.39 2.37 2.34 2.32 2.30 2.29 2.27 2.25 1.00 0.98 0.96
Me
HN
NH+Cl-
NH2+Cl-
Cl
Me
24
9 8 7 6 5 4 3 2 1 ppm
1.00 1.10 1.15 4.95 0.98 1.18 2.05 4.07 3.00 2.30 2.93

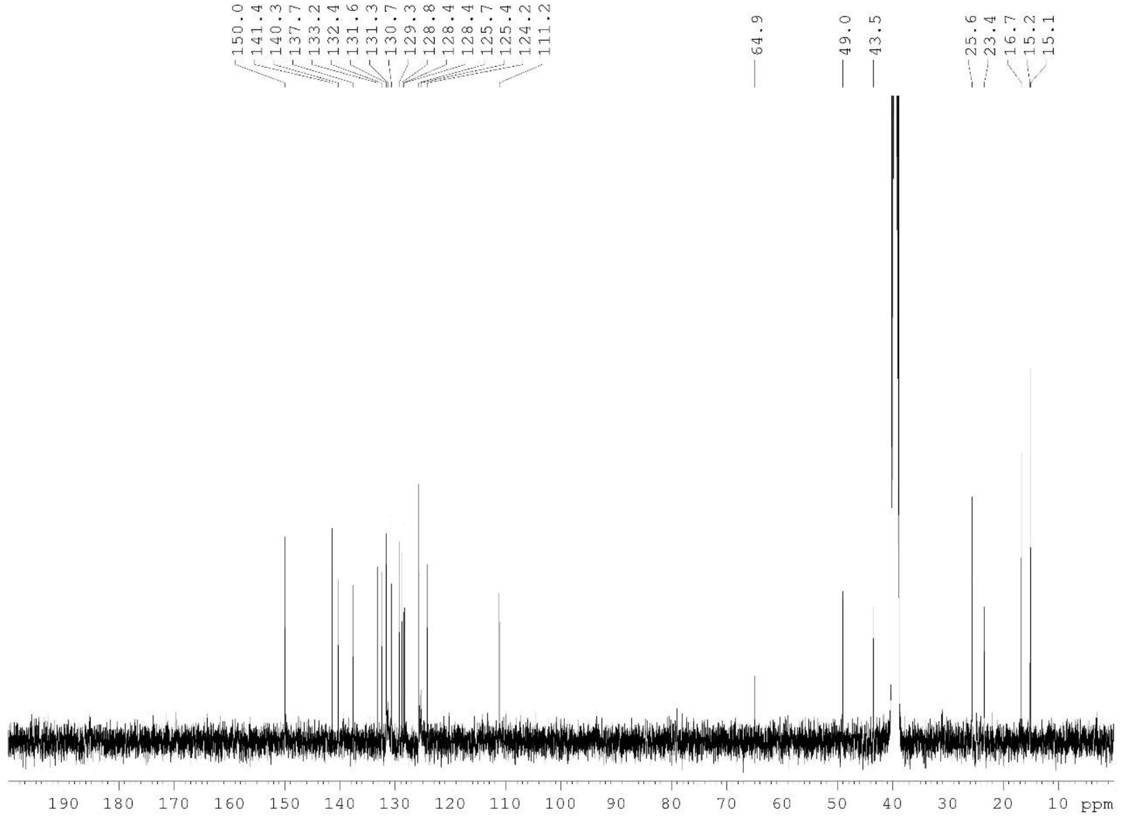
150.0 141.4 140.3 137.7 133.2 132.4 131.6 131.3 130.7 129.3 128.8 128.4 125.7 125.4 124.2 111.2
64.9
49.0
43.5
25.6 23.4 16.7 15.2 15.1
190 180 170 160 150 140 130 120 110 100 90 80 70 60 50 40 30 20 10 ppm

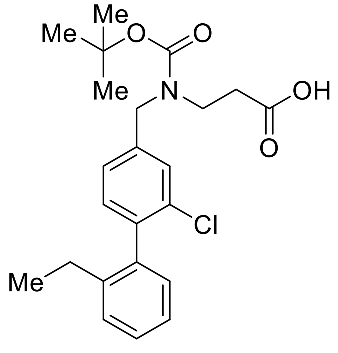
Me
Me
O
O
Me
N
OH
O
Cl
Me

25e

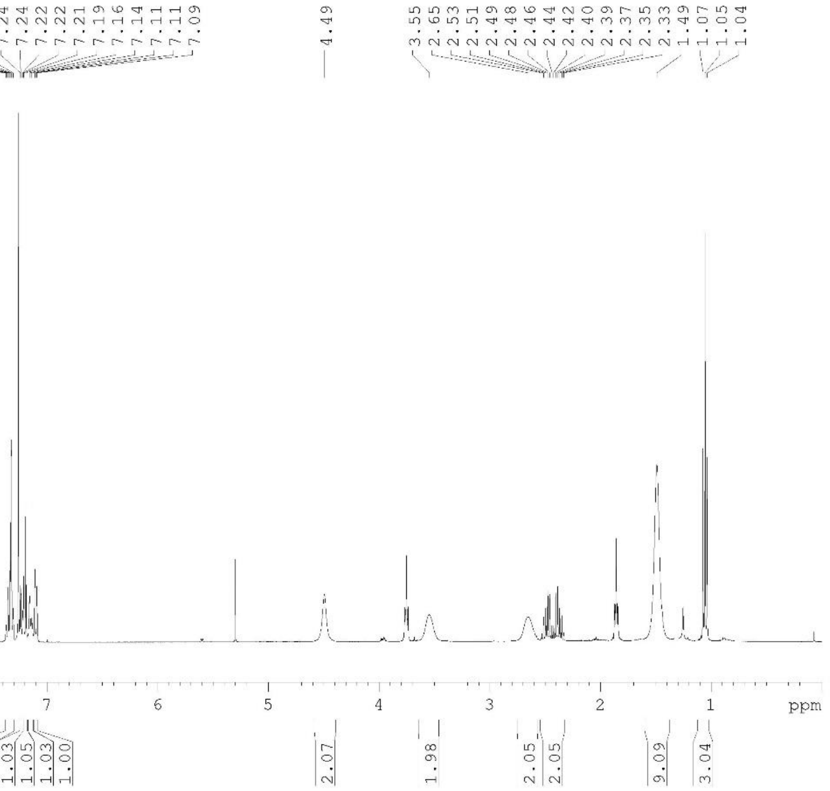
7.37 7.37 7.35 7.35 7.34 7.33 7.32 7.31 7.24 7.24 7.22 7.22 7.21 7.19 7.16 7.14 7.11 7.11 7.09
4.49
3.55 2.65 2.53 2.51 2.49 2.48 2.46 2.44 2.42 2.40 2.39 2.37 2.35 2.33 1.49 1.07 1.05 1.04
9 8 7 6 5 4 3 2 1 ppm
2.90 1.03 1.05 1.03 1.00
2.07
1.98
2.05 2.05
9.09 3.04

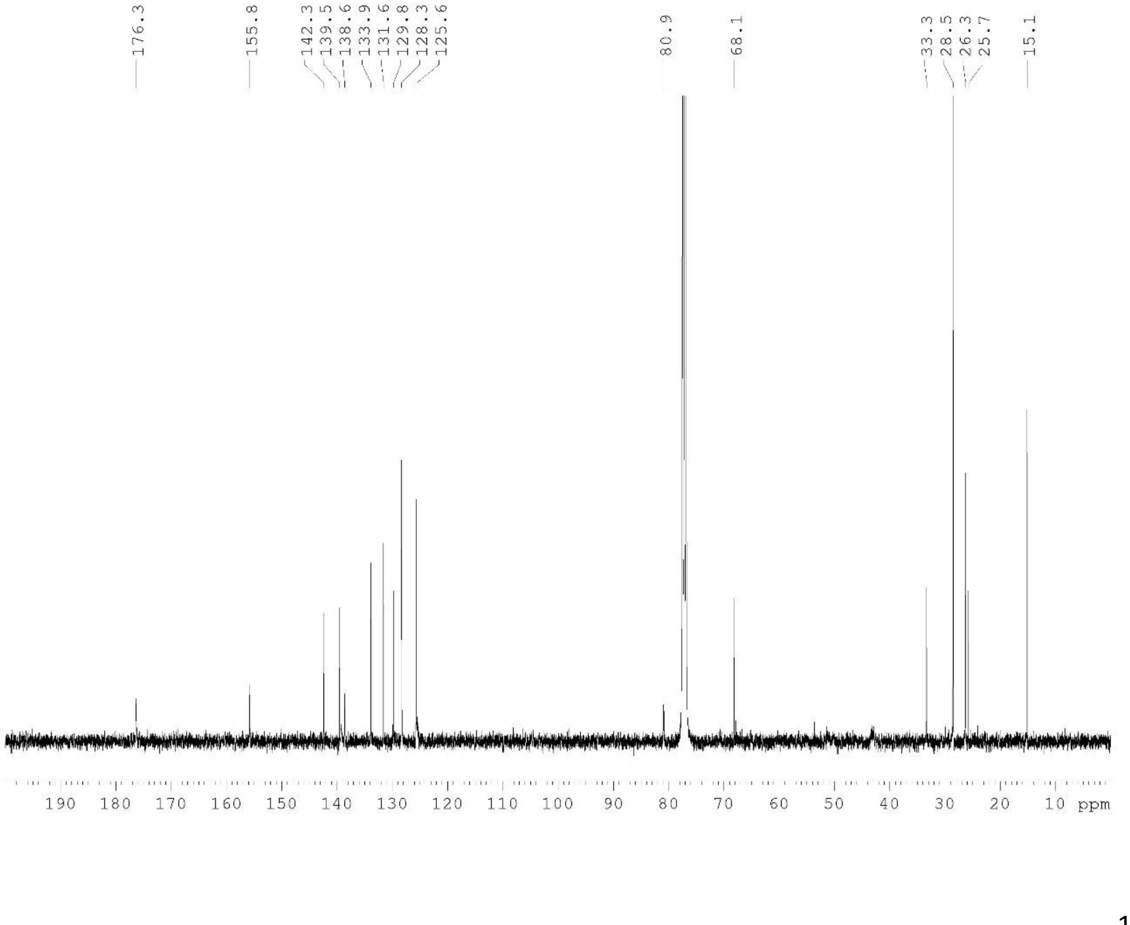
176.3
155.8
142.3 139.5 138.6 133.9 131.6 129.8 128.3 125.6
80.9
68.1
33.3 28.5 26.3 25.7
15.1
190 180 170 160 150 140 130 120 110 100 90 80 70 60 50 40 30 20 10 ppm

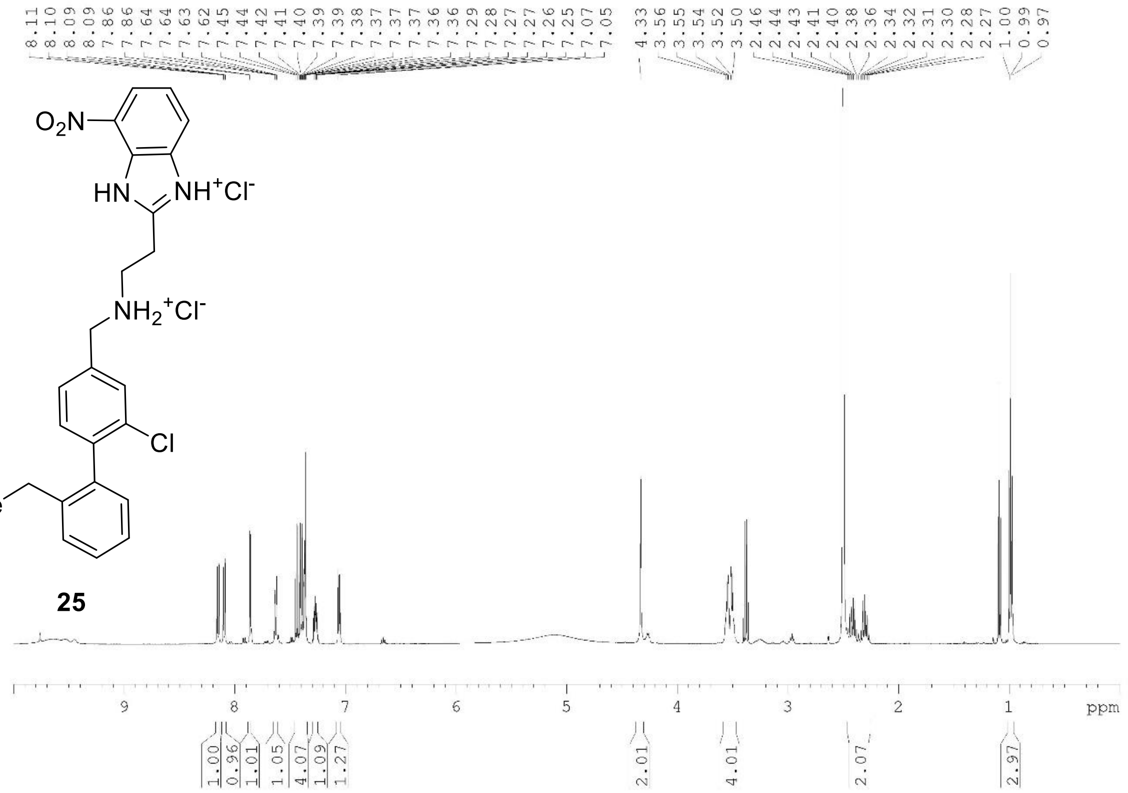
O2N
HN
NH+Cl-
NH2+Cl-
Cl
Me
25
8.11 8.10 8.09 8.09 7.86 7.86 7.64 7.63 7.62 7.45 7.44 7.42 7.41 7.40 7.39 7.39 7.38 7.37 7.37 7.37 7.36 7.36 7.29 7.28 7.27 7.27 7.26 7.25 7.07 7.05 4.33 3.56 3.55 3.54 3.52 3.50 2.46 2.44 2.43 2.41 2.40 2.38 2.36 2.34 2.32 2.31 2.30 2.28 2.27 1.00 0.99 0.97
9 8 7 6 5 4 3 2 1 ppm
1.00 0.96 1.01 1.05 4.07 1.09 1.27 2.01 4.01 2.07 2.97

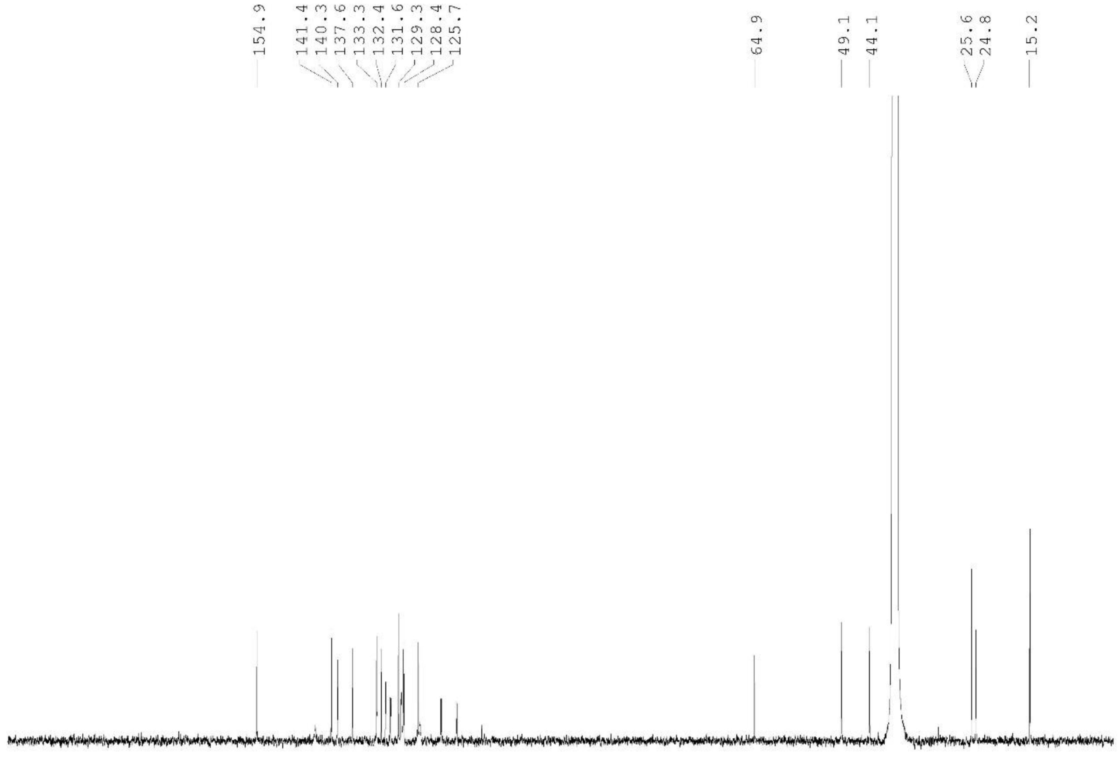
154.9 141.4 140.3 137.6 133.3 132.4 131.6 129.3 128.4 125.7 64.9 49.1 44.1 25.6 24.8 15.2
190 180 170 160 150 140 130 120 110 100 90 80 70 60 50 40 30 20 10 ppm

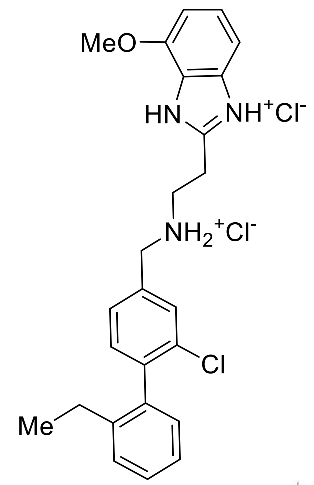
MeO
HN
NH+Cl-
NH2+Cl-
Cl
Me

26

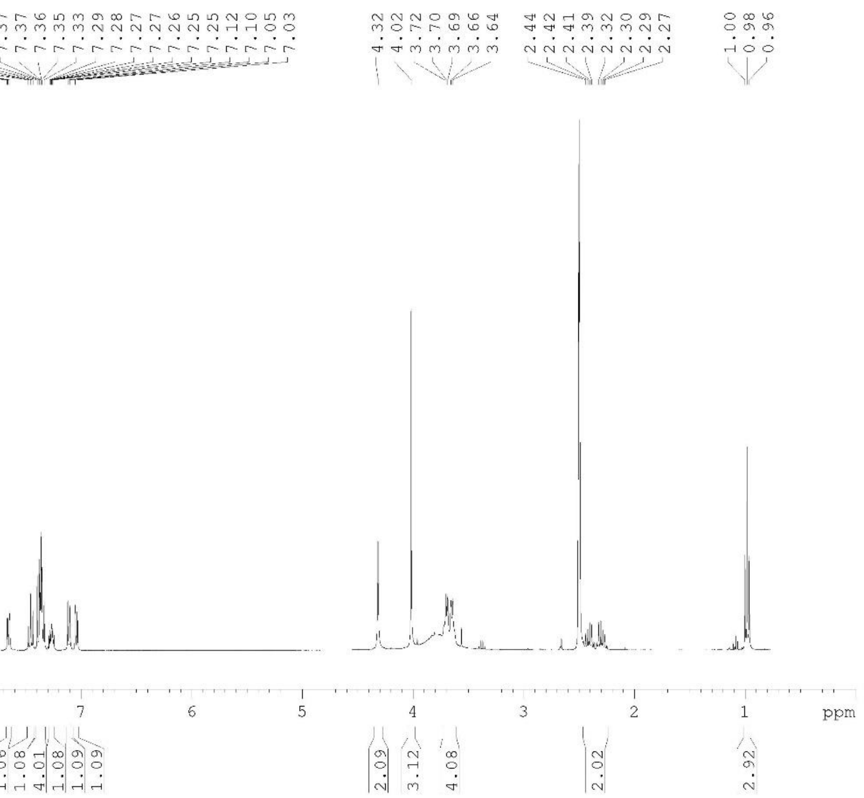
7.89 7.89 7.67 7.66 7.65 7.64 7.48 7.46 7.44 7.40 7.38 7.37 7.37 7.36 7.35 7.33 7.29 7.28 7.27 7.27 7.26 7.25 7.25 7.22 7.12 7.10 7.05 7.03
4.32 4.02 3.72 3.70 3.69 3.66 3.64
2.44 2.42 2.41 2.39 2.32 2.30 2.29 2.27
1.00 0.98 0.96
9 8 7 6 5 4 3 2 1 ppm
1.00 1.06 1.08 4.01 1.08 1.09 1.09
2.09 3.12 4.08
2.02
2.92

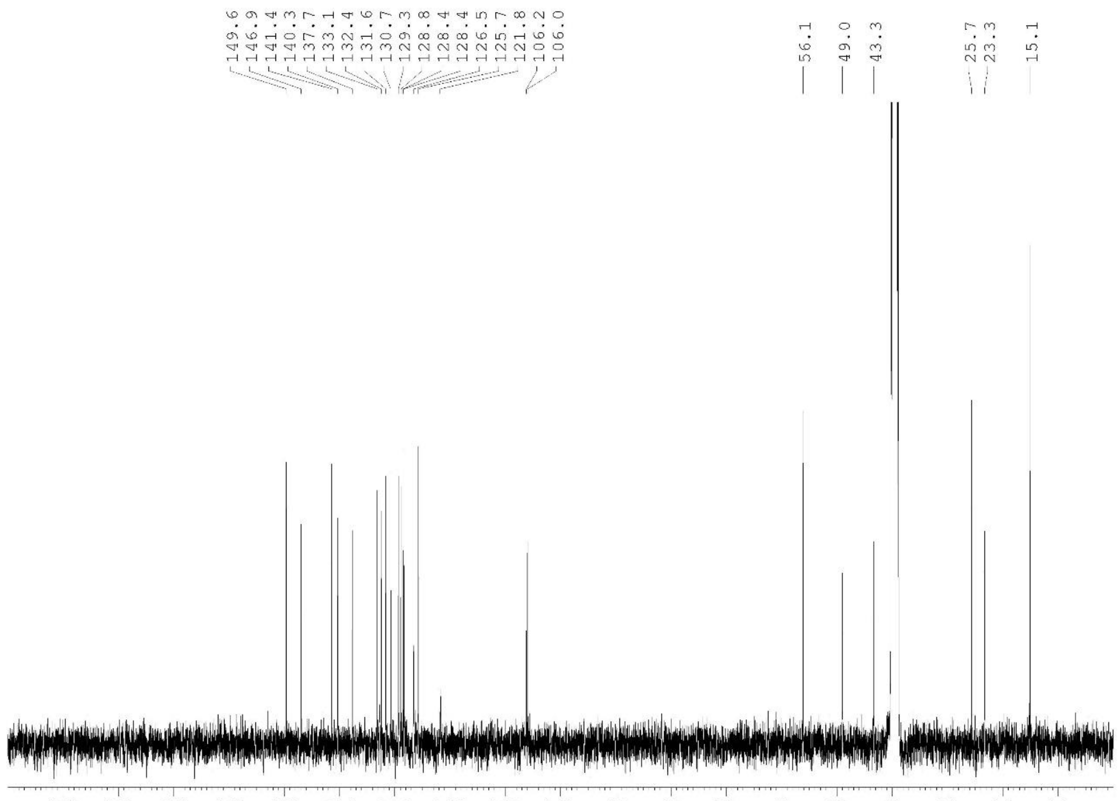
149.6 146.9 141.4 140.3 137.7 133.1 132.4 131.6 130.7 129.3 128.8 128.4 128.4 126.5 125.7 121.8 106.2 106.0
56.1 49.0 43.3
25.7 23.3 15.1
190 180 170 160 150 140 130 120 110 100 90 80 70 60 50 40 30 20 10 ppm

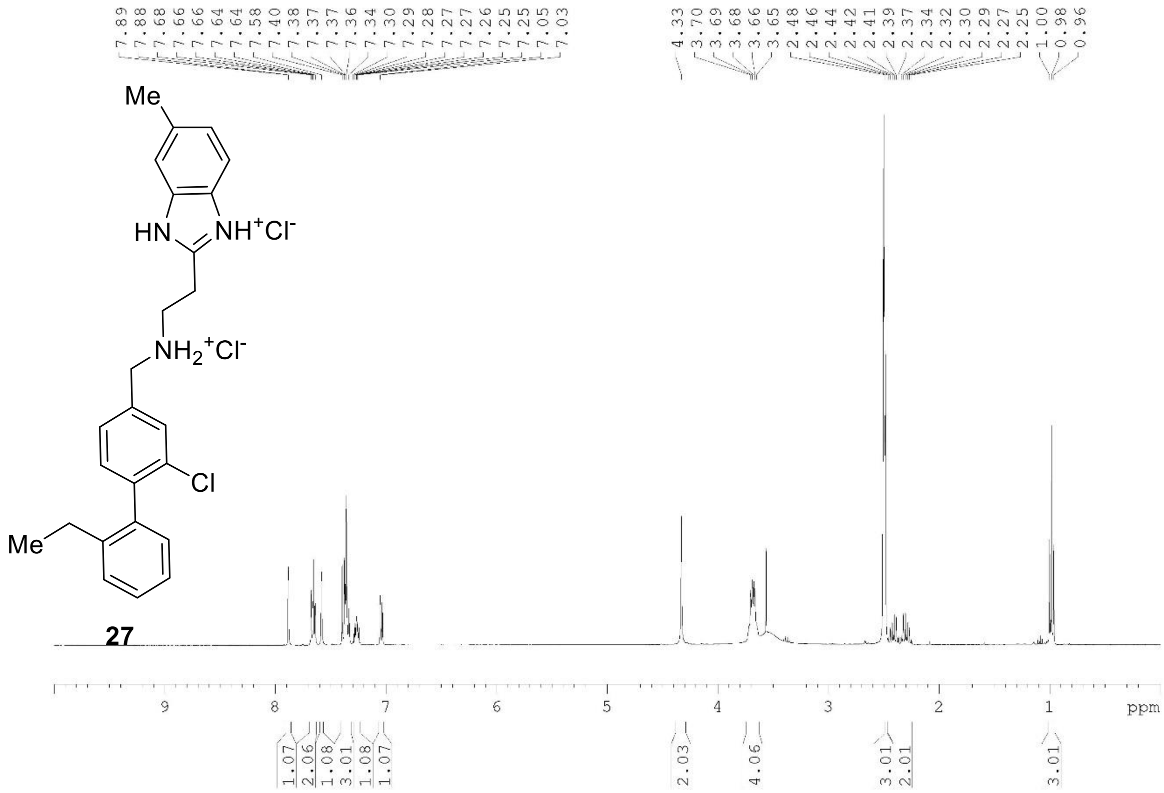
Me
HN
NH+Cl-
NH2+Cl-
Cl
Me
27
7.89 7.88 7.68 7.66 7.66 7.64 7.58 7.40 7.38 7.37 7.37 7.36 7.34 7.30 7.29 7.28 7.27 7.27 7.26 7.25 7.25 7.05 7.03
4.33 3.70 3.69 3.68 3.66 3.65 2.48 2.46 2.44 2.42 2.41 2.39 2.37 2.34 2.32 2.30 2.29 2.27 2.25 1.00 0.98 0.96
9 8 7 6 5 4 3 2 1 ppm
1.07 2.06 1.08 3.01 1.08 1.07 2.03 4.06 3.01 2.01 3.01

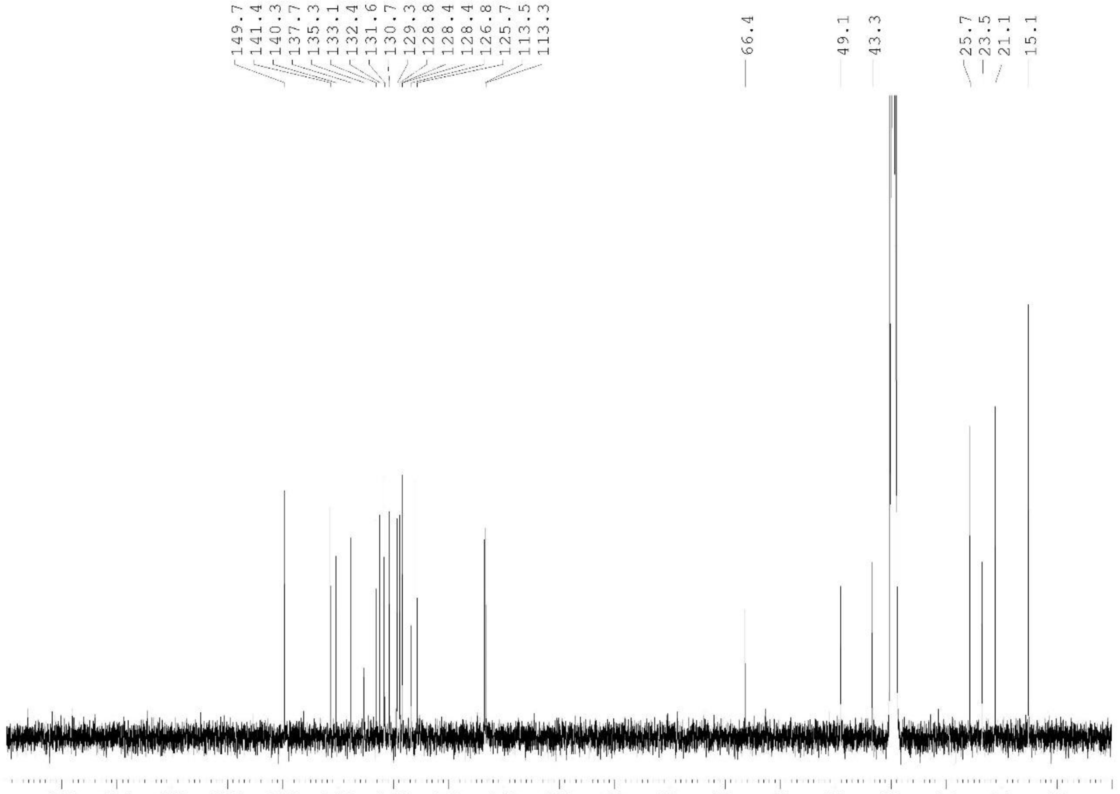
149.7 141.4 140.3 137.7 135.3 133.1 132.4 131.6 130.7 129.3 128.8 128.4 126.8 125.7 113.5 113.3
66.4
49.1
43.3
25.7 23.5 21.1 15.1
190 180 170 160 150 140 130 120 110 100 90 80 70 60 50 40 30 20 10 ppm

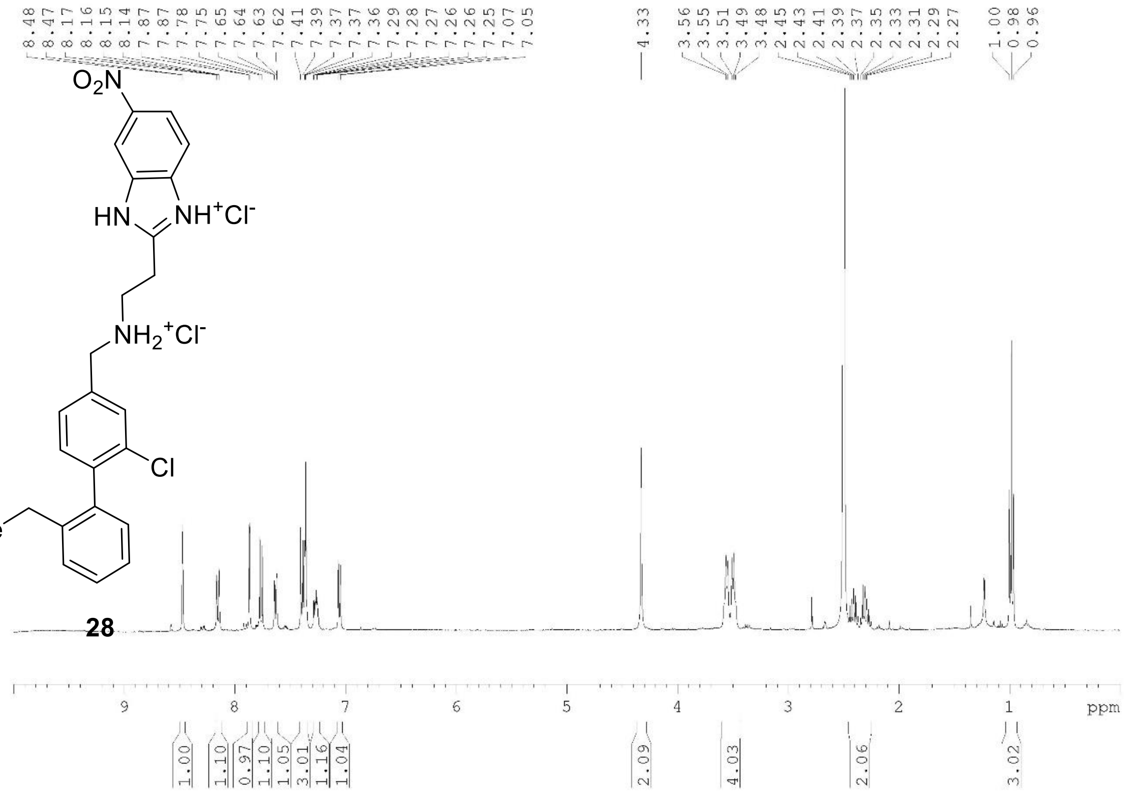
8.48 8.47 8.17 8.16 8.15 8.14 7.87 7.87 7.78 7.75 7.65 7.64 7.63 7.62 7.41 7.39 7.37 7.37 7.36 7.29 7.28 7.27 7.26 7.25 7.07 7.05
4.33
3.56 3.55 3.51 3.49 3.48 2.45 2.43 2.41 2.39 2.37 2.35 2.33 2.31 2.29 2.27
1.00 0.98 0.96
O2N
HN
NH+Cl-
NH2+Cl-
Cl
Me
28
9 8 7 6 5 4 3 2 1 ppm
1.00 1.10 0.97 1.10 1.05 3.01 1.16 1.04 2.09 4.03 2.06 3.02

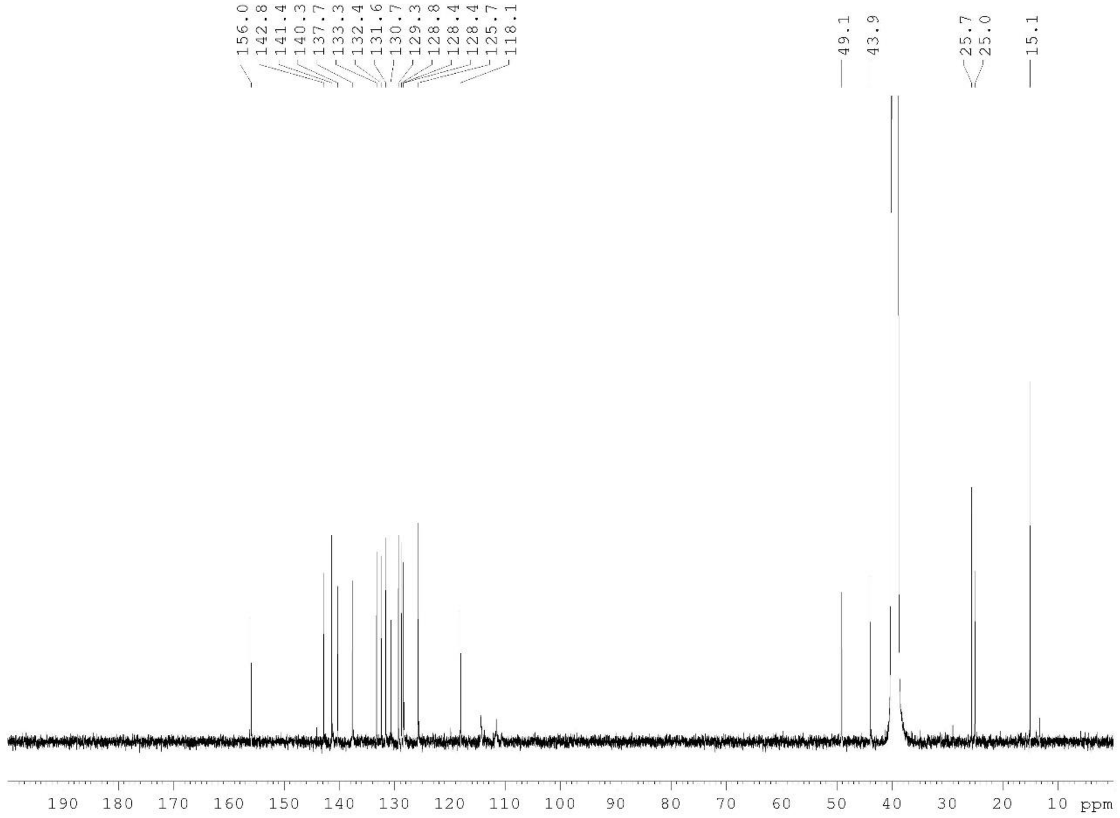
156.0 142.8 141.4 140.3 137.7 133.3 132.4 131.6 130.7 129.3 128.8 128.4 128.4 125.7 118.1
49.1 43.9
25.7 25.0
15.1
190 180 170 160 150 140 130 120 110 100 90 80 70 60 50 40 30 20 10 ppm

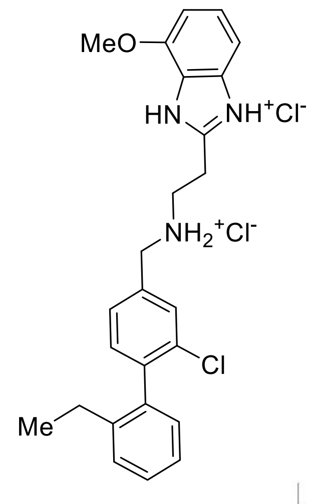
MeO
HN
NH+Cl-
NH2+Cl-
Cl
Me

29

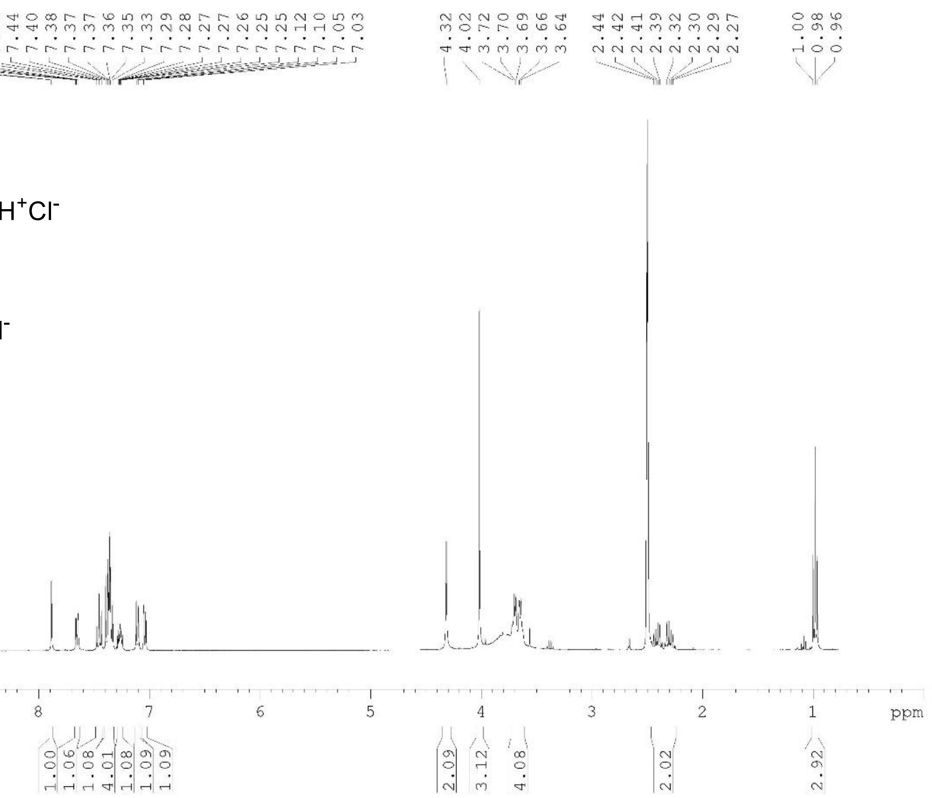
7.89 7.89 7.67 7.66 7.65 7.64 7.48 7.46 7.44 7.40 7.38 7.37 7.37 7.36 7.35 7.33 7.29 7.28 7.27 7.27 7.26 7.25 7.25 7.12 7.10 7.05 7.03
4.32 4.02 3.72 3.70 3.69 3.66 3.64
2.44 2.42 2.41 2.39 2.32 2.30 2.29 2.27
1.00 0.98 0.96
9 8 7 6 5 4 3 2 1 ppm
1.00 1.06 1.08 4.01 1.08 1.09 1.09
2.09 3.12 4.08
2.02
2.92

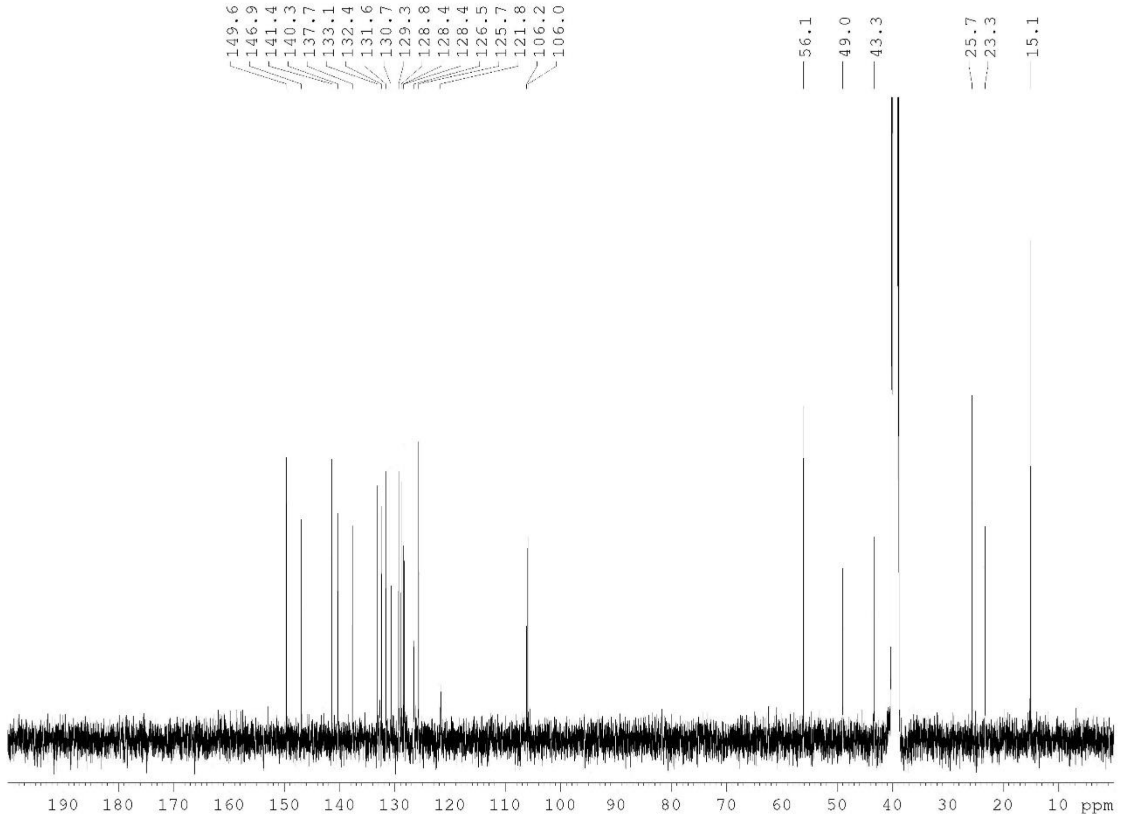
149.6 146.9 141.4 140.3 137.7 133.1 132.4 131.6 130.7 129.3 128.8 128.4 128.4 126.5 125.7 121.8 106.2 106.0
56.1
49.0
43.3
25.7 23.3
15.1
190 180 170 160 150 140 130 120 110 100 90 80 70 60 50 40 30 20 10 ppm

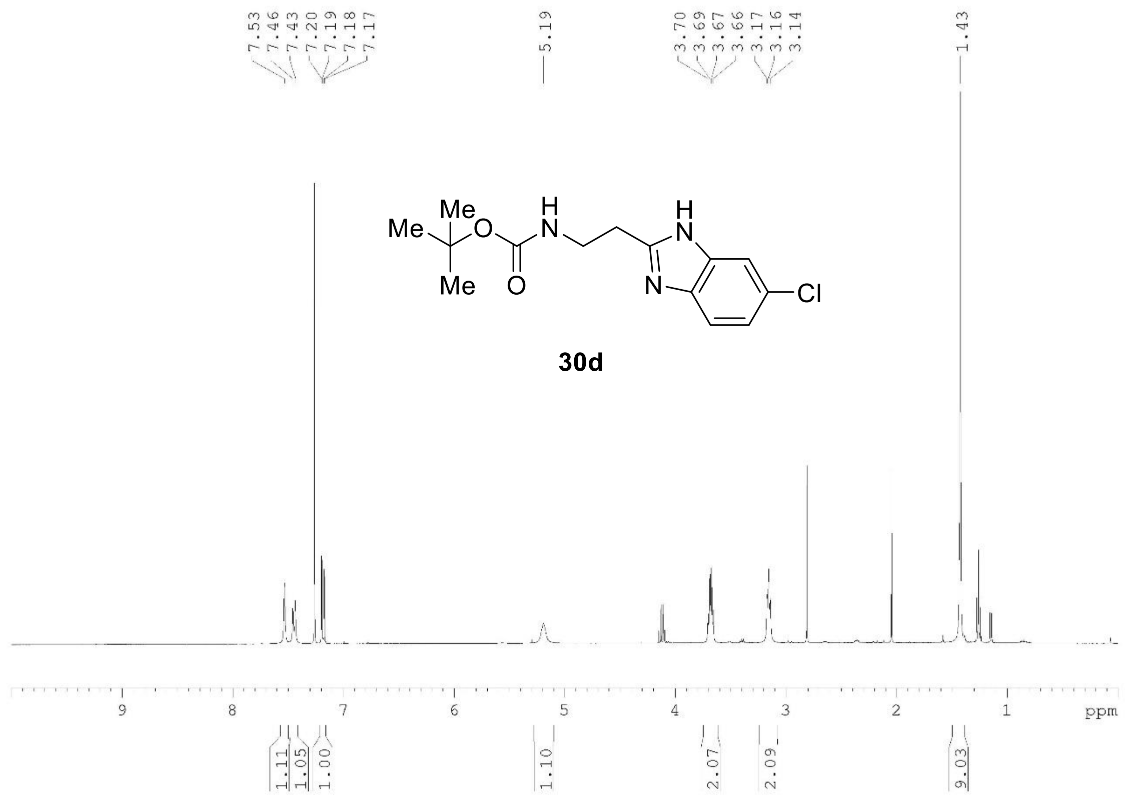
Me
Me
Me
O
O
H
N
H
N
N
Cl
30d
7.53
7.46
7.43
7.20
7.19
7.18
7.17
5.19
3.70
3.69
3.67
3.66
3.17
3.16
3.14
1.43
9 8 7 6 5 4 3 2 1 ppm
1.11
1.05
1.00
1.10
2.07
2.09
9.03

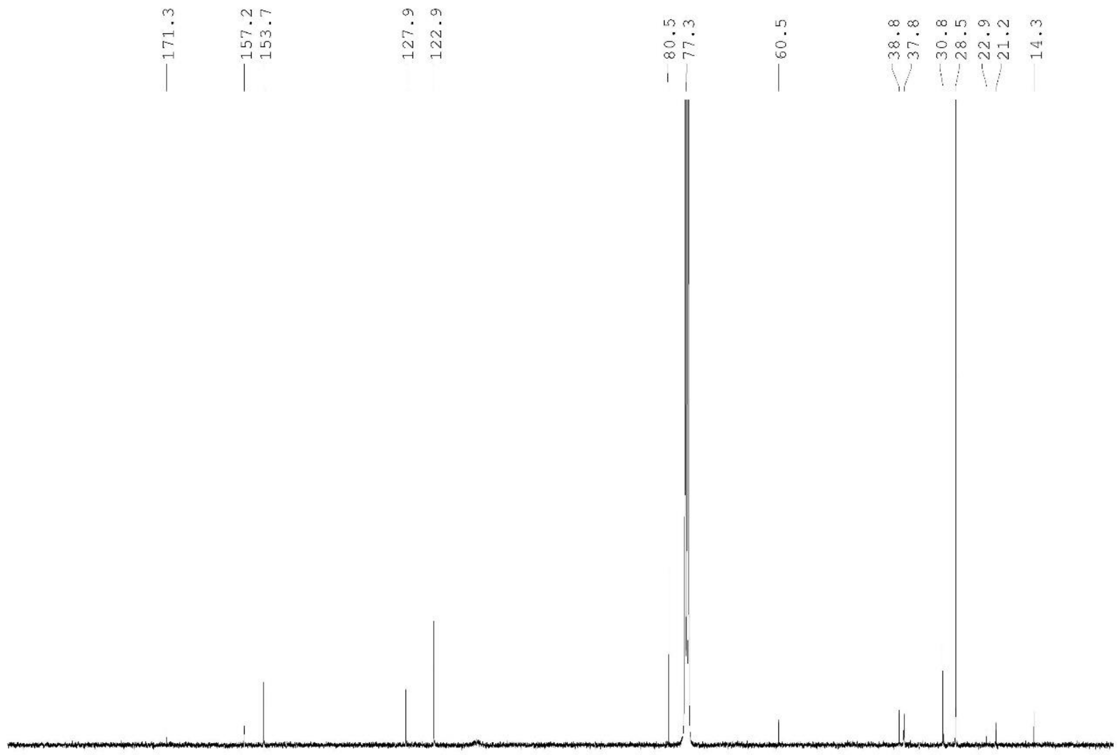
171.3
157.2
153.7
127.9
122.9
80.5
77.3
60.5
38.8
37.8
30.8
28.5
22.9
21.2
14.3
190 180 170 160 150 140 130 120 110 100 90 80 70 60 50 40 30 20 10 ppm

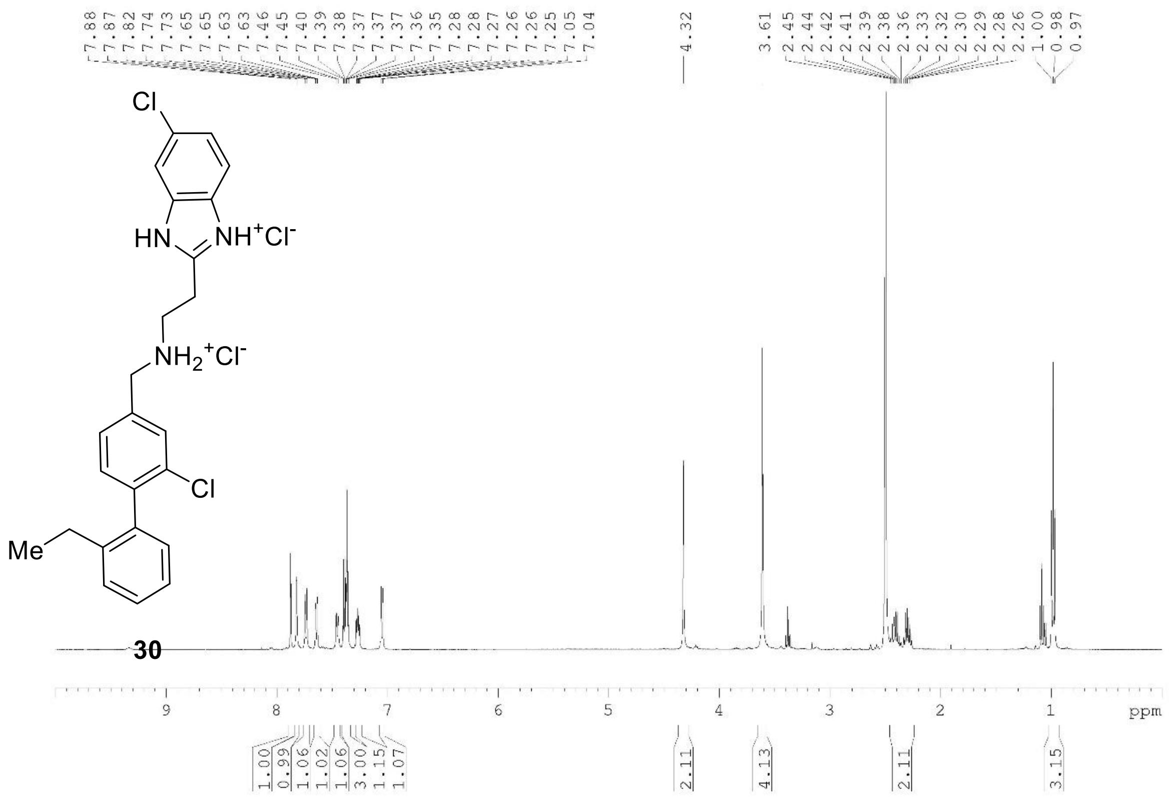

30

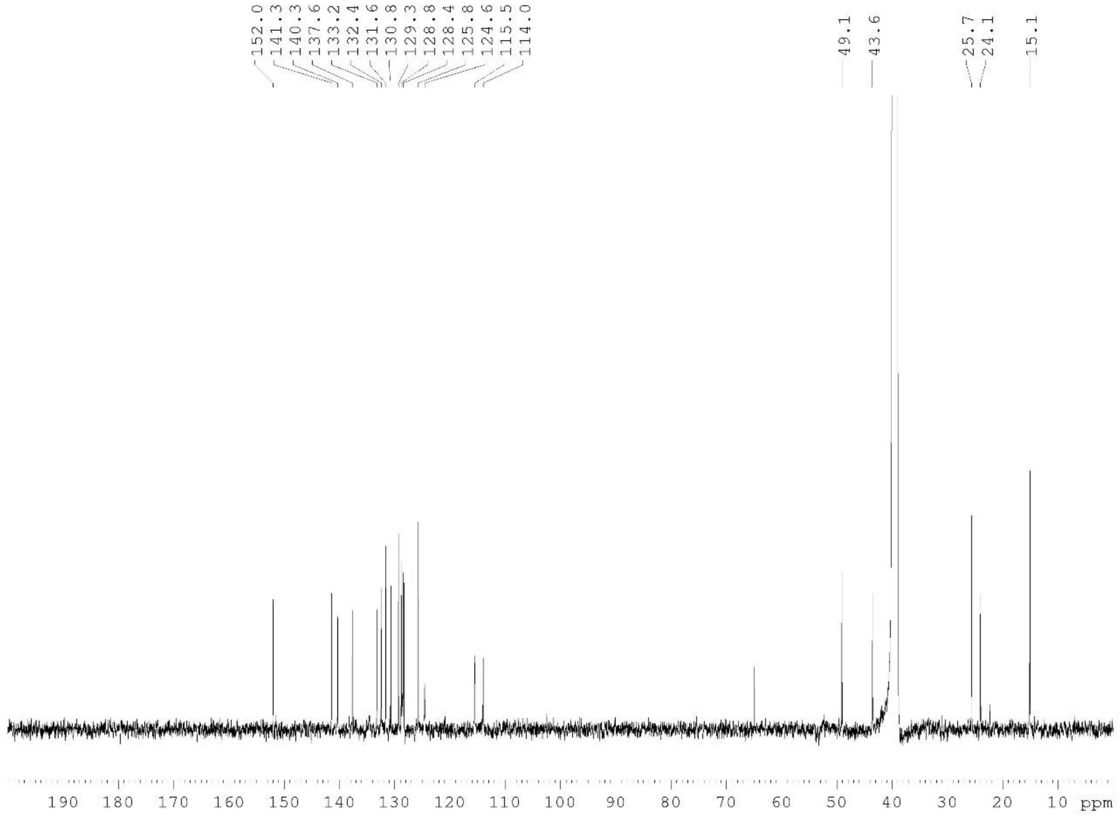

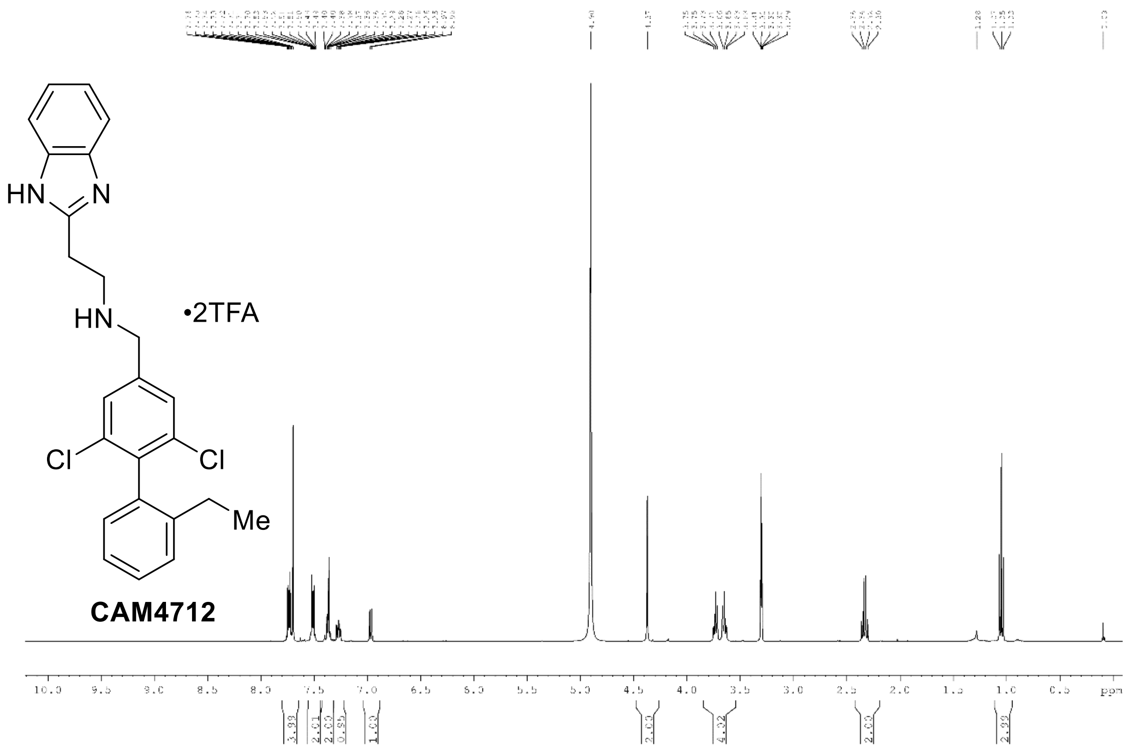
HN
N
HN
•2TFA
Cl
Cl
Me
CAM4712

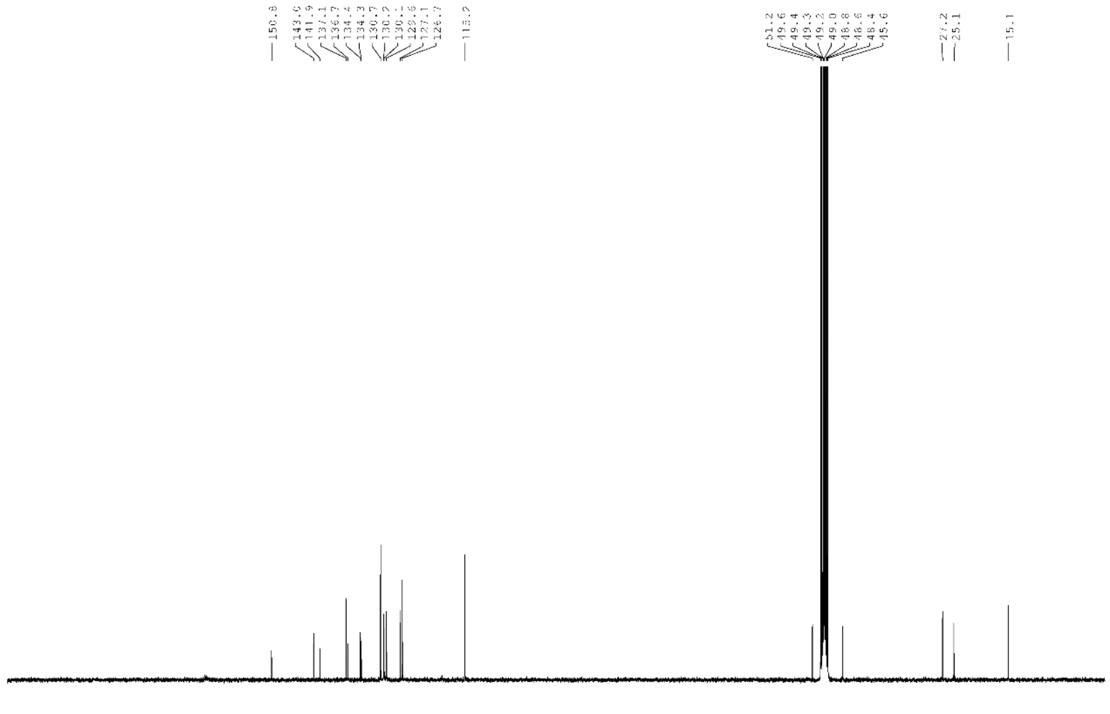

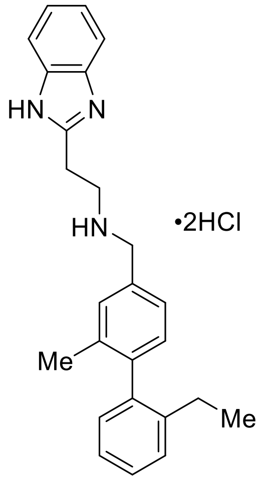
HN
N
HN
•2HCl
Me
Me

31

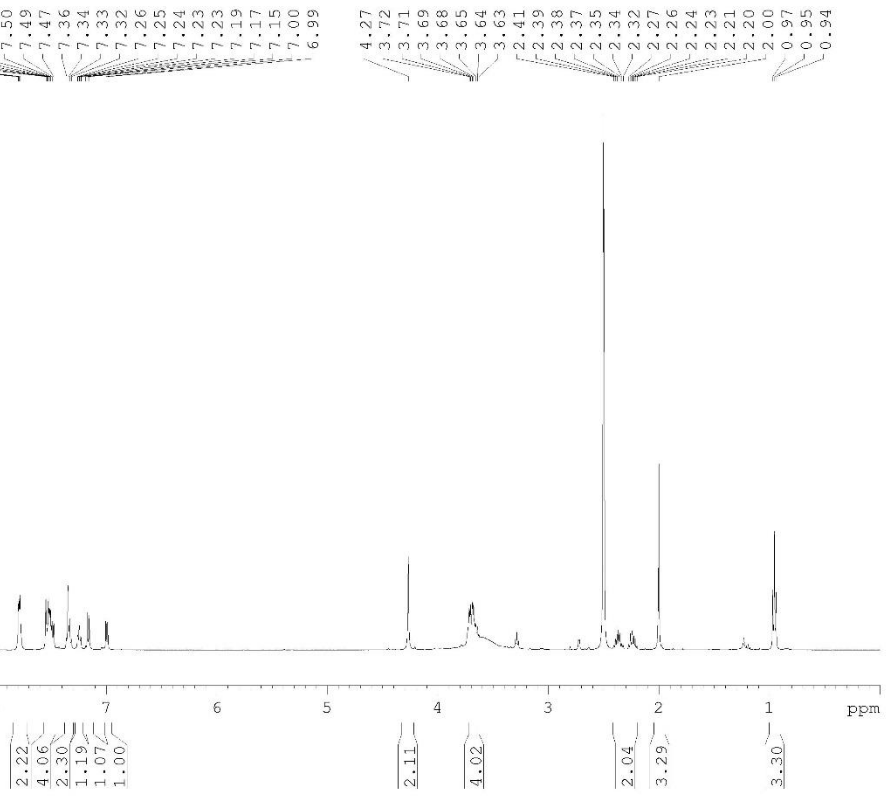
7.79 7.79 7.78 7.78 7.55 7.53 7.53 7.52 7.52 7.51 7.50 7.49 7.47 7.36 7.34 7.33 7.32 7.26 7.25 7.24 7.23 7.23 7.19 7.17 7.15 7.00 6.99
4.27 3.72 3.71 3.69 3.68 3.65 3.64 3.63 2.41 2.39 2.38 2.37 2.35 2.34 2.32 2.27 2.26 2.24 2.23 2.21 2.20 2.00 0.97 0.95 0.94
9 8 7 6 5 4 3 2 1 ppm
2.22 4.06 2.30 1.19 1.07 1.00
2.11
4.02
2.04 3.29
3.30

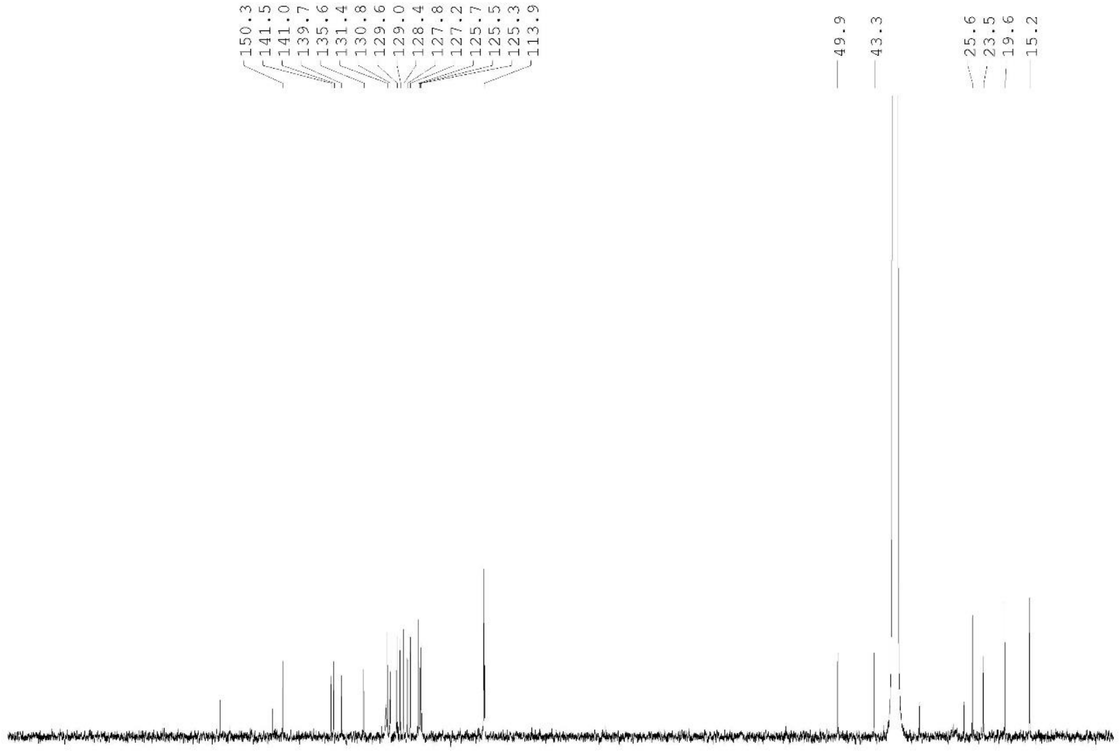
150.3 141.5 141.0 139.7 135.6 131.4 130.8 129.6 129.0 128.4 127.8 127.2 125.7 125.5 125.3 113.9
49.9 43.3
25.6 23.5 19.6 15.2
190 180 170 160 150 140 130 120 110 100 90 80 70 60 50 40 30 20 10 ppm

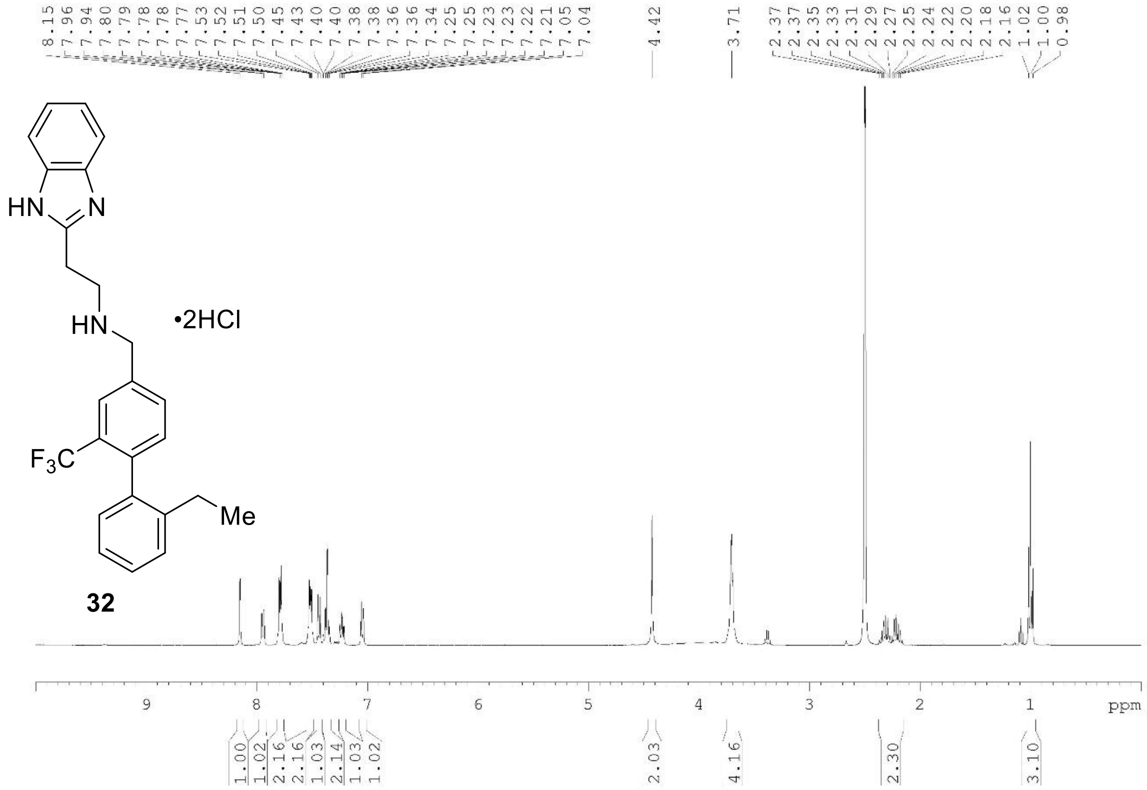
HN
N
HN
•2HCl
F_3C
Me
32
8.15 7.96 7.94 7.80 7.79 7.78 7.78 7.77 7.53 7.52 7.51 7.50 7.45 7.43 7.40 7.40 7.38 7.38 7.36 7.36 7.34 7.25 7.25 7.23 7.23 7.22 7.21 7.05 7.04
4.42
3.71
2.37 2.37 2.35 2.33 2.31 2.29 2.27 2.25 2.24 2.22 2.20 2.18 2.16 1.02 1.00 0.98
9 8 7 6 5 4 3 2 1 ppm
1.00 1.02 2.16 2.16 1.03 2.14 1.03 1.02
2.03
4.16
2.30
3.10

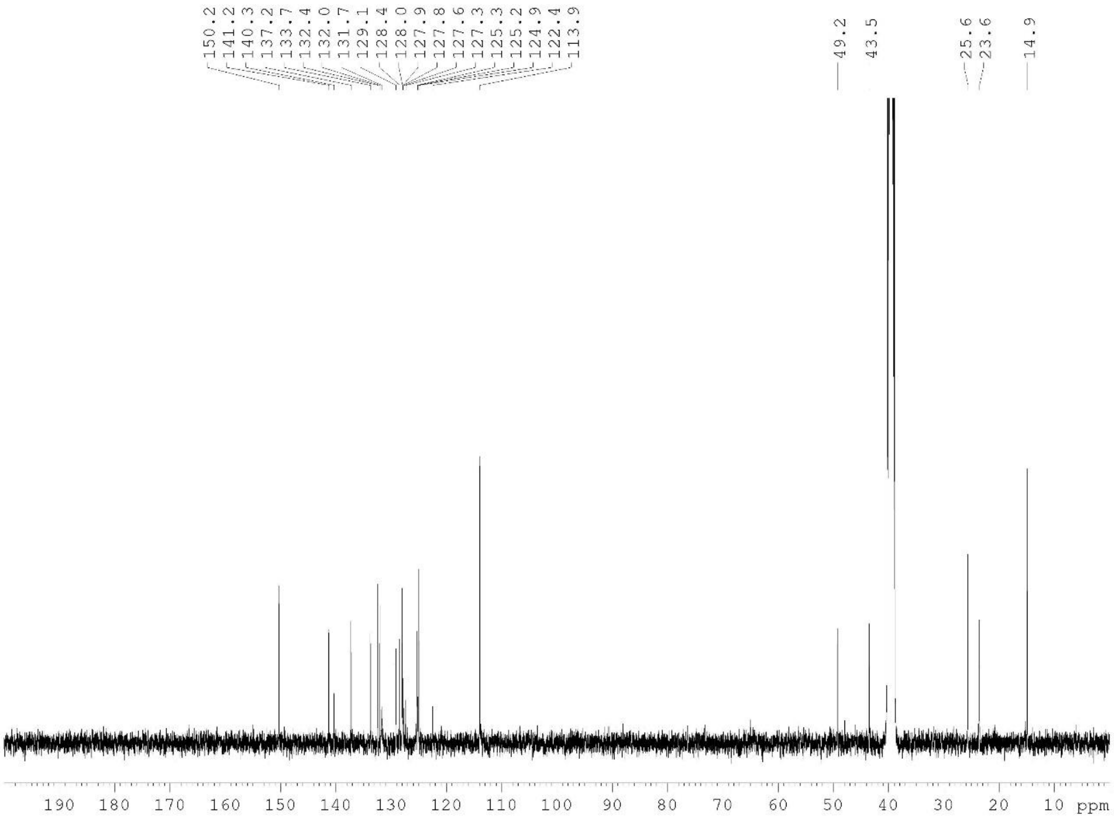
150.2 141.2 140.3 137.2 133.7 132.4 132.0 131.7 129.1 128.4 128.0 127.9 127.8 127.6 127.3 125.3 125.2 124.9 122.4 113.9
49.2
43.5
25.6
23.6
14.9
190 180 170 160 150 140 130 120 110 100 90 80 70 60 50 40 30 20 10 ppm

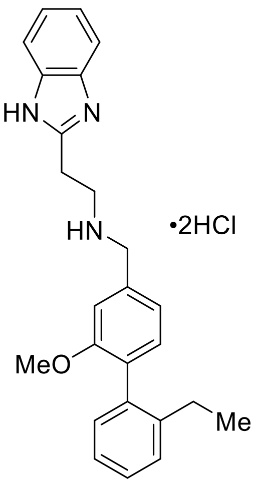
HN
N
HN
•2HCl
MeO
Me

33

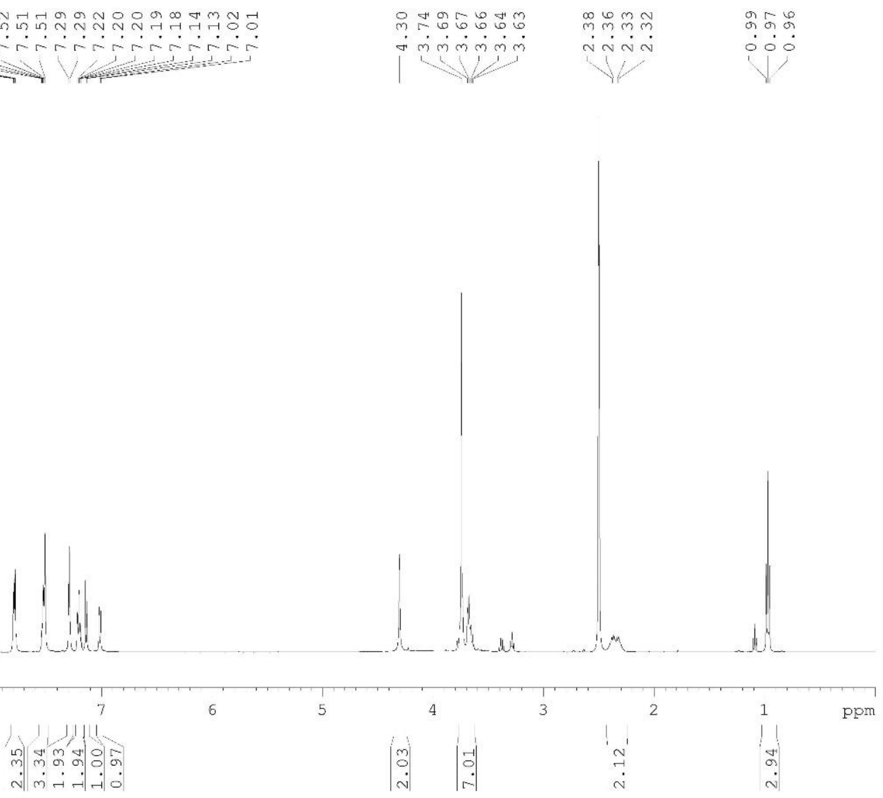
7.80
7.79
7.78
7.78
7.54
7.53
7.53
7.52
7.51
7.51
7.29
7.29
7.22
7.20
7.20
7.19
7.18
7.14
7.13
7.02
7.01
4.30
3.74
3.69
3.67
3.66
3.64
3.63
2.38
2.36
2.33
2.32
0.99
0.97
0.96
9
8
7
6
5
4
3
2
1
ppm
2.35
3.34
1.93
1.94
1.00
0.97
2.03
7.01
2.12
2.94

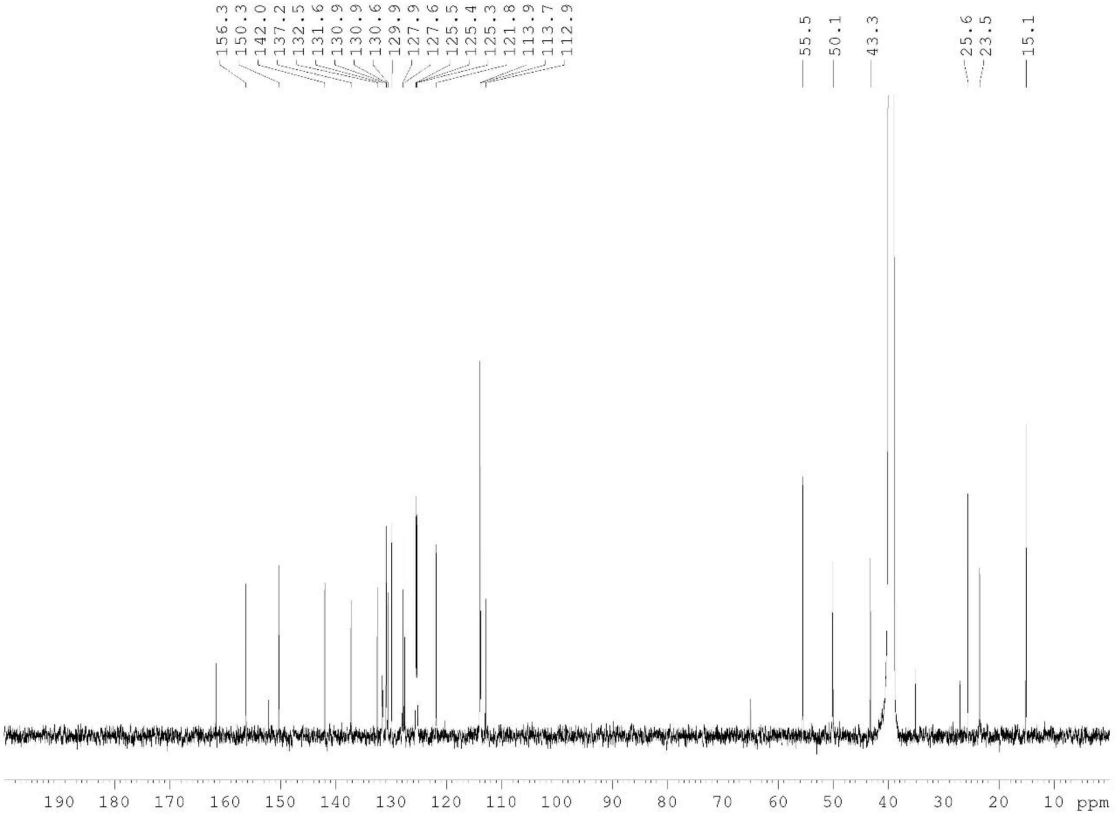
156.3
150.3
142.0
137.2
132.5
131.6
130.9
130.9
130.6
129.9
127.9
127.6
127.5
125.5
125.4
125.3
121.8
113.9
113.7
112.9
55.5
50.1
43.3
25.6
23.5
15.1
190
180
170
160
150
140
130
120
110
100
90
80
70
60
50
40
30
20
10
ppm

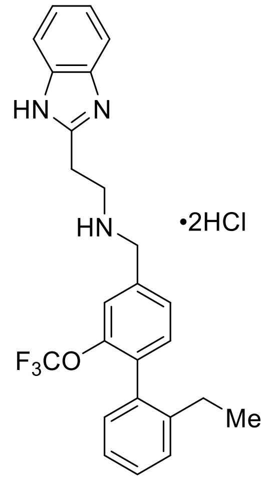
HN
N
HN
•2HCl
F3CO
Me

34

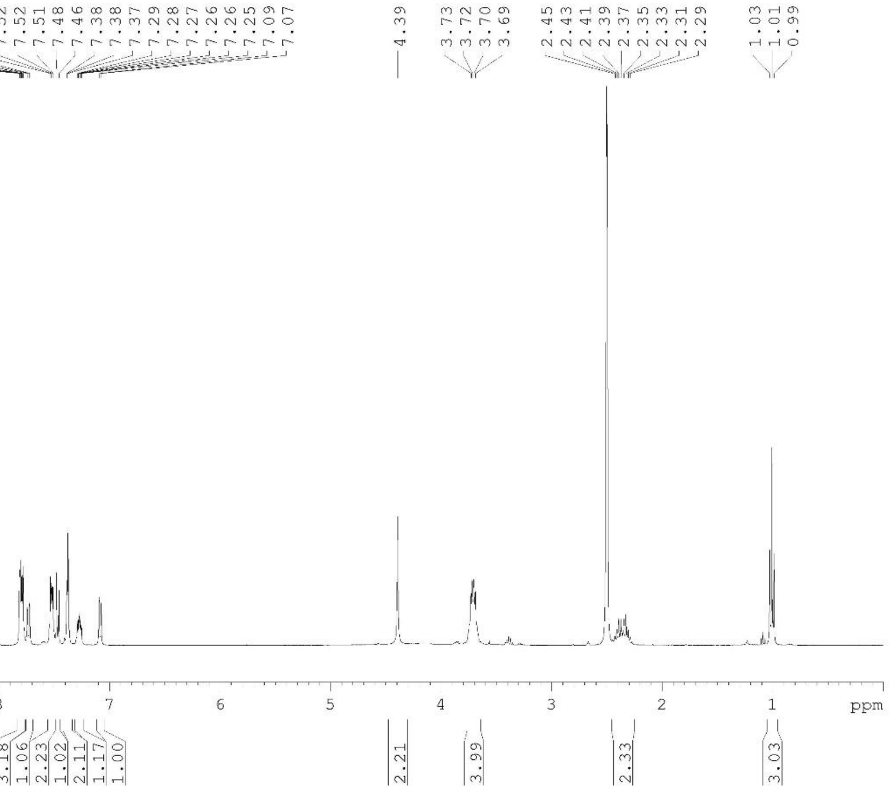
7.81 7.80 7.80 7.79 7.78 7.74 7.72 7.72 7.53 7.52 7.52 7.51 7.48 7.46 7.38 7.38 7.37 7.29 7.28 7.27 7.26 7.26 7.25 7.09 7.07
4.39
3.73 3.72 3.70 3.69
2.45 2.43 2.41 2.39 2.37 2.35 2.33 2.31 2.29
1.03 1.01 0.99
9 8 7 6 5 4 3 2 1 ppm
3.18 1.06 2.23 1.02 2.11 1.17 1.00
2.21
3.99
2.33
3.03

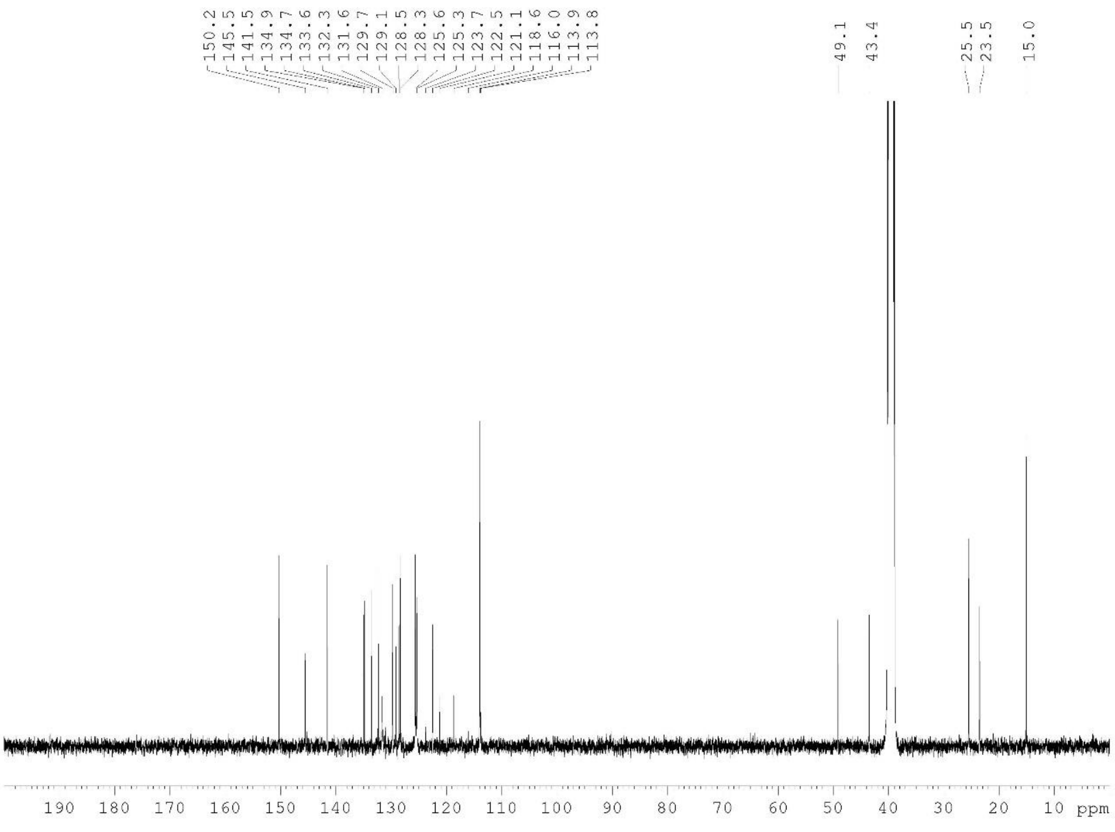
150.2 145.5 141.5 134.9 134.7 133.6 132.3 131.6 129.7 129.1 128.5 128.3 125.6 125.3 123.7 122.5 121.1 118.6 116.0 113.9 113.8
49.1
43.4
25.5 23.5
15.0
190 180 170 160 150 140 130 120 110 100 90 80 70 60 50 40 30 20 10 ppm

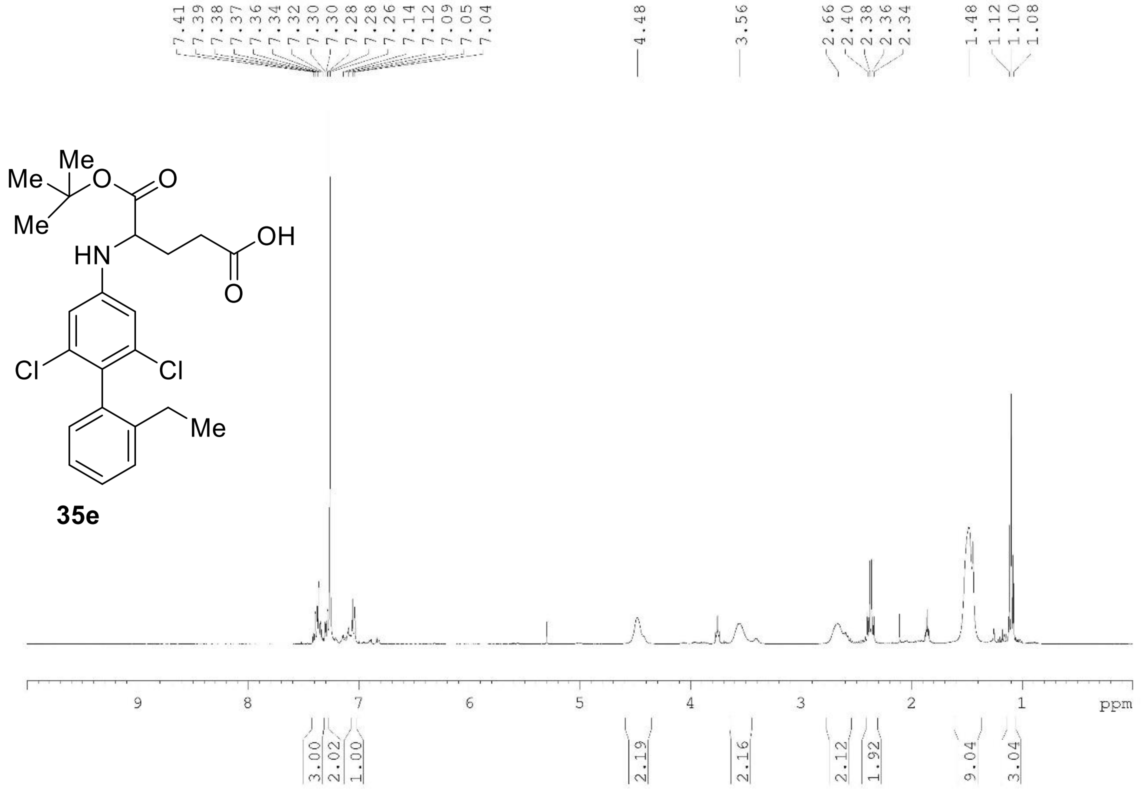
Me
Me
O
O
Me
HN
OH
O
Cl
Cl
Me

35e

7.41 7.39 7.38 7.37 7.36 7.34 7.32 7.30 7.30 7.28 7.28 7.26 7.14 7.12 7.09 7.05 7.04
4.48
3.56
2.66 2.40 2.38 2.36 2.34
1.48 1.12 1.10 1.08
9 8 7 6 5 4 3 2 1 ppm
3.00 2.02 1.00
2.19
2.16
2.12 1.92
9.04 3.04

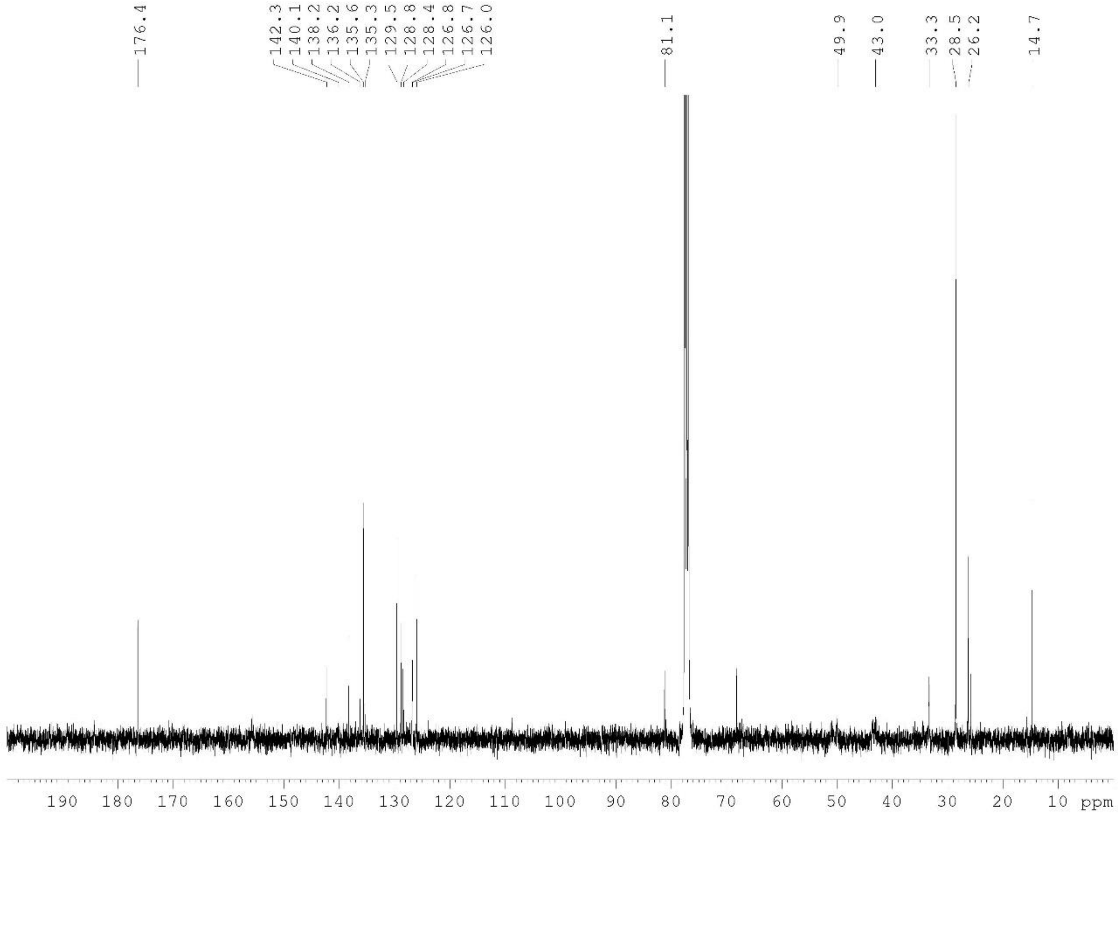
176.4
142.3 140.1 138.2 136.2 135.6 135.3 129.5 128.8 128.4 126.8 126.7 126.0
81.1
49.9
43.0
33.3 28.5 26.2
14.7
190 180 170 160 150 140 130 120 110 100 90 80 70 60 50 40 30 20 10 ppm

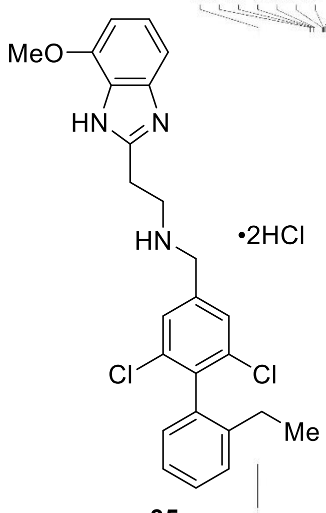

35

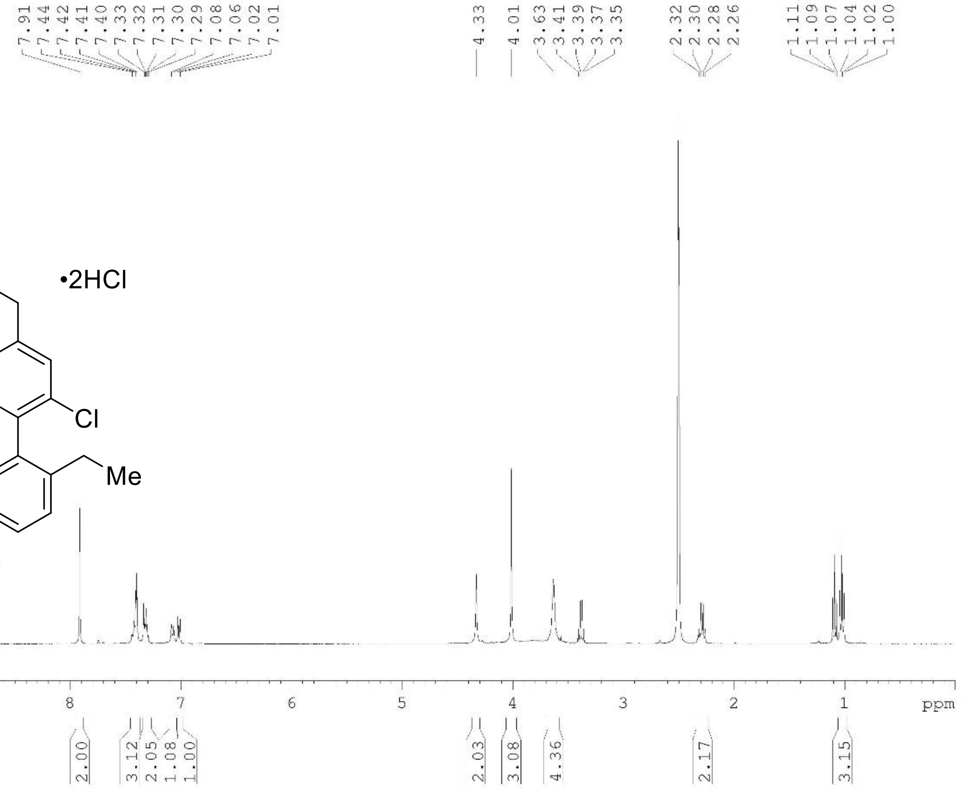

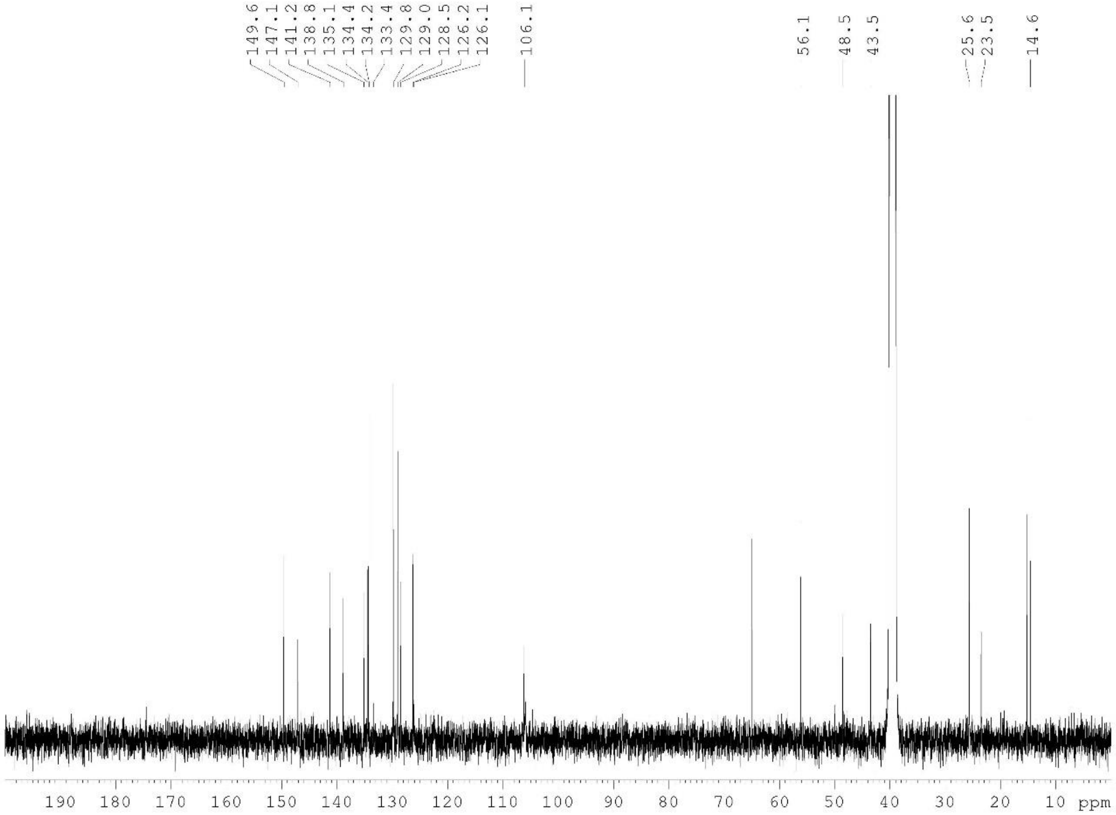

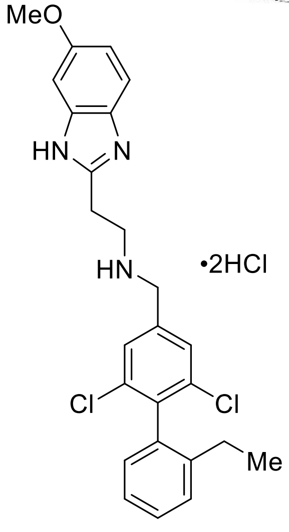
MeO
HN
N
HN
•2HCl
Cl
Cl
Me

36

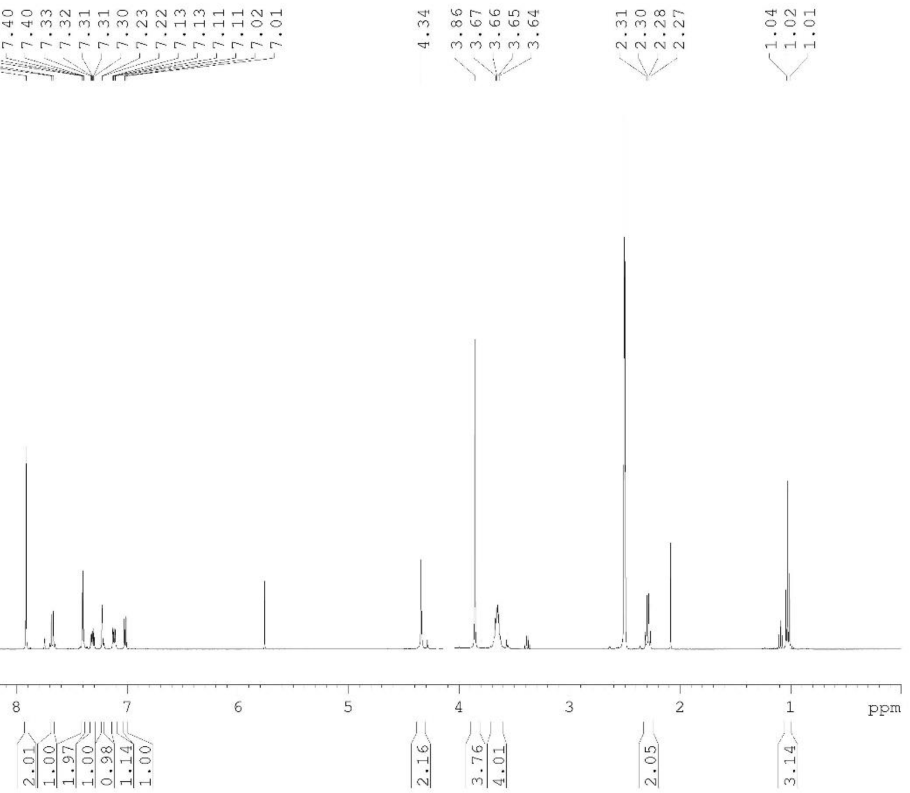
7.91 7.69 7.67 7.41 7.41 7.40 7.40 7.33 7.32 7.31 7.31 7.30 7.23 7.22 7.13 7.13 7.11 7.11 7.02 7.01
4.34 3.86 3.67 3.66 3.65 3.64
2.31 2.30 2.28 2.27
1.04 1.02 1.01
9 8 7 6 5 4 3 2 1 ppm
2.01 1.00 1.97 1.00 0.98 1.14 1.00
2.16
3.76 4.01
2.05
3.14

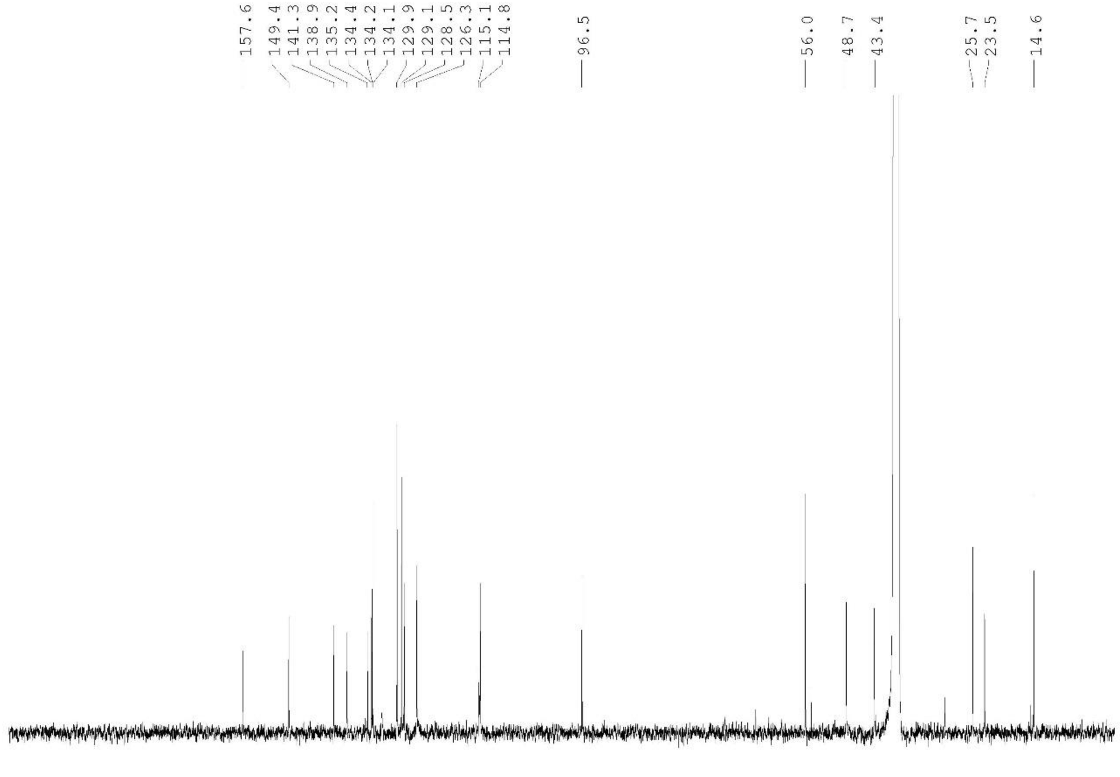
157.6
149.4 141.3 138.9 135.2 134.4 134.2 134.1 129.9 129.1 128.5 126.3 115.1 114.8
96.5
56.0 48.7 43.4
25.7 23.5
14.6
190 180 170 160 150 140 130 120 110 100 90 80 70 60 50 40 30 20 10 ppm

TABLE S1

Compound	Kd (µM)	Enthalpy (cal/mol)	Entropy (cal/mol/deg)
**2**	41	-5054	3.11
**3**	17	-2784	12.5
**4**	205	-1846	-23.2
**5**	244	-1184	-23.2
**6**	234	-1253	-25.4
**7**	375	-5671	-3.34
**11**	105	-6977	-5.21
**12**	172	-8425	-11
**14**	12	-3333	9.78
**15**	6.5	-6361	11.8
**CAM4712**	8.6	-4090	9.46
**CAM4712** in competition with **CAM4066**	4.0	-11710	n/a*
**CAM4712** in competition with **15**	5.0	-3420	n/a*
**CAM4712** in competition with **CX4945**	3.0	-5900	n/a*

*The model for competitive binding used with origin did not give the entropy therefore none is reported.

TABLE S2

Crystallisation and ligand soaking conditions

**PDB code**	5ORH	5ORJ	5OS7	5OQU	5ORK	5OSL	5OUL
Ligand	2	3	4	5	6	7	9
Ligand code	**A4N**	**A4Q**	**A8Q**	**A4B**	**A4T**	**A9K**	**AWE**
Construct	FP10	FP10	FP10	FP10	FP10	KA	KA
Crystallisation:	A	A	A	A	A	B	B
Cryo-cooling:	10 mM **2** in D	10 mM **3** in D	10 mM **4** in D	10 mM **5** in D	10 mM **6** in D	10 mM **7** in C	10 mM **9** in C
Soaking:	10 mM **2** in D, 16h	10 mM **3** in D, 16 h	10 mM **4** in D, 16h	10 mM **5** in D, 16h	10 mM **6** in D, 16h	10 mM **7** in C, 16h	10 mM **9** in C, 16h

**PDB code**	5OS8	5OTR	5OTZ	6EII	5OT6	5OUE	5OUM	5OUU
Ligand	11	14	15	18	19	20	21	22
Ligand code	**J27**	**AU8**	**AUT**	**B5W**	**AJK**	**AVZ**	**AVK**	**C84**
Construct	KA	KA	KA	FP10	FP10	FP10	FP10	FP10
Crystallisation:	B	B	B	A	A	A	A	A
Cryo-cooling:	10 mM **11** in C	10 mM **14** in C	10 mM **15** in C	10 mM **18** in D	10 mM **19** in D	10 mM **20** in D	10 mM **21** in D	10 mM **22** in D
Soaking:	10 mM **11** in C, 16h	10 mM **14** in C, 16 h	10 mM **15** in C, 16 h	10 mM **18** in D, 16 h	10 mM **19** in D, 16 h	10 mM **20** in D, 16 h	10 mM **21** in D, 16 h	10 mM **22** in D, 16 h

**PDB code**	5OSZ	5OT5	50TD	5OTH	5OTI	5OTL	5OTO	5OYF
Ligand	23	24	25	26	27	29	30	31
Ligand code	**AHK**	**AWK**	**AOW**	**AQ8**	**AOK**	**AQT**	**AQW**	**B4Q**
Construct	KA	FP10	FP10	FP10	KA	FP10	FP10	KA
Crystallisation:	B	A	A	A	B	A	A	B
Cryo-cooling:	10 mM **23** in C	10 mM **24** in D	10 mM **25** in D	10 mM **26** in D	10 mM **27** in C	10 mM **29** in D	10 mM **30** in D	10 mM **31** in C
Soaking:	10 mM **23** in C, 16 h	10 mM **24** in D, 16 h	10 mM **25** in D, 16 h	10 mM **26** in D, 16 h	10 mM **27** in C, 16 h	10 mM **29** in D, 16 h	10 mM **30** in D, 16 h	10 mM **31** in C, 16 h

**PDB code**	6EHU	5OTQ	5OTY	6EHK
Ligand	32	33	CAM4712	CAM4712, 37
Ligand code	**B5E**	**AUH**	**AUW**	**AUW, 54G**
Construct	KA	KA	KA	KA
Crystallisation:	B	B	B	B
Cryo-cooling:	10 mM **32** in C	10 mM **33** in C	10 mM **CAM4712** in C	10 mM **4712** and 10 mM 37 in D
Soaking:	10 mM **32** in C, 16h	10 mM **33** in C, 16 h	10 mM **CAM4712** in C, 16 h	10 mM **4712** and 10 mM 37 in D, 16 h

Condition A: 107mM Mes pH 6.5, 29% glycerol ethoxylate, 1 M ammonium acetate

Condition B: 112.5mM Mes pH 6.5, 35% glycerol ethoxylate, 180 mM ammonium acetate

Condition C:	107mM Mes pH 6.5, 29% glycerol ethoxylate, 1 M ammonium acetate, 5% DMSO
Condition D:	107mM Mes pH 6.5, 29% glycerol ethoxylate, 1 M ammonium acetate, 30% DMSO

TABLE S3

Compound	2	3	4
Construct	CK2A_FP10	CK2A_FP10	CK2A_FP10
Crystallisation conditions	Condition A	Condition A	Condition A
**Data collection and processing:**			
Beamline	DIAMOND BEAMLINE I04	DIAMOND BEAMLINE I04	DIAMOND BEAMLINE I24
Wavelength (Å)	0.9795	0.9795	0.9686
Resolution range (Å)	83.33 - 1.75 (1.753 - 1.748)	166.8- 1.99 (1.994 - 1.988)	55.54 - 1.66 (1.700 - 1.660)
Space group	C 2 2 21	C 2 2 21	C 2 2 21
Cell (a b c) (Å)	64.52 67.97 333.33	64.79 68.60 333.66	64.74 68.84 333.26
Cell (alpha beta gamma) (°)	90.00 90.00 90.00	90.00 90.00 90.00	90.00 90.00 90.00
Total reflections	485802 (4103)	337321 (3421)	687678 (44116)
Unique reflections	74809 (707)	51855 (522)	88560 (6484)
Multiplicity	6.5 (5.8)	6.5 (6.6)	7.8 (6.8)
Completeness (%)	100.0 (100.0)	100.0 (100.0)	100.0 (100.0)
Mean I/sigma(I)	18.1 (2.1)	17.2 (2.1)	8.1 (1.5)
R-merge	0.052 (0.79)	0.056 (1.13)	0.130 (1.88)
R-pim	0.022 (0.36)	0.024 (0.47)	0.050 (0.77)
CC-half	0.999 (0.82)	0.999 (0.79)	0.994 (0.52)
**Refinement:**			
R-factor / Rfree	0.208 / 0.227	0.200 / 0.221	0.221 / 0.247
Number of total atoms	5924	5815	6053
atoms for ligands	76	146	107
atoms for waters	314	144	405
Number of polymer residues	649	649	651
Average/Wilson B-factor ($Å^2$)	39.5 / 31.0	62.6 / 42.1	25.5 / 18.1
B-factor for ligands ($Å^2$)	44.8	95.9	33.6
B-factor for solvent ($Å^2$)	44.6	55.6	29.6
RMS bonds (Å)	0.011	0.01	0.01
RMS bond angles (Å)	0.96	0.94	0.94
RMS dihedral angles (°)	3.04	2.96	3.18
Crystallisation conditions	Condition A	Condition A	Condition A
PDB code	5ORH	5ORJ	5OS7

Crystallisation and cryo conditions

A: 107mM Mes pH 6.5, 29% glycerol ethoxylate, 1 M ammonium acetate

B: 112.5mM Mes pH 6.5, 35% glycerol ethoxylate, 180 mM ammonium acetate

5	6	7	9
CK2A_FP10	CK2A_FP10	CK2A_KA	CK2A_KA
Condition A	Condition A	Condition B	Condition B
DIAMOND BEAMLINE I04	DIAMOND BEAMLINE I04	BRUKER AXS MICROSTAR	DIAMOND BEAMLINE I03
0.9795	0.9795	1.54	0.9762
166.40 - 2.32 (2.331 - 2.324)	166.04 - 2.14 (2.151 - 2.143)	36.27 - 1.95 (1.980 - 1.950)	58.59 - 1.34 (1.370 - 1.340)
C 2 2 21	C 2 2 21	P 1 21 1	P 1 21 1
64.67 67.92 332.80	64.98 68.74 332.07	58.61 46.40 62.76	58.70 46.08 63.37
90.00 90.00 90.00	90.00 90.00 90.00	90.00 112.11 90.00	90.00 112.39 90.00
209930 (2051)	272382 (2885)	327090 (-)	270297 (19436)
32253 (321)	41363 (433)	23058 (-)	69349 (5042)
6.5 (6.4)	6.6 (6.7)	14.1 (5.4)	3.9 (3.9)
100.0 (100.0)	99.8 (100.0)	99.5 (90.9)	98.6 (97.1)
18.4 (2.3)	18.4 (2.0)	17.6 (2.0)	8.1 (1.7)
0.054 (0.84)	0.054 (1.05)	0.121 (0.58)	0.070 (0.91)
0.023 (0.36)	0.023 (0.44)	- (-)	0.039 (0.53)
1.000 (0.89)	0.999 (0.76)	- (-)	0.996 (0.43)
0.220 / 0.244	0.216 / 0.243	0.163 / 0.211	0.198 / 0.222
5671	5735	3124	3199
130	78	90	143
15	150	234	250
648	647	328	327
73.5 / 59.8	63.0 / 49.5	20.4 / 19.0	25.1 / 16.2
90.8	71.6	23.8	49
51.3	57	26.7	33.3
0.01	0.01	0.01	0.01
1	0.96	0.96	0.95
2.79	2.94	3.24	3.51
Condition A	Condition A	Condition B	Condition B
5OQU	5ORK	5OSL	5OUL

11	14	15	18
CK2A_KA	CK2A_KA	CK2A_KA	CK2A_FP10
Condition B	Condition B	Condition B	Condition A
BRUKER AXS MICROSTAR	BRUKER AXS MICROSTAR	DIAMOND BEAMLINE I03	DIAMOND BEAMLINE I02
1.54	1.54	0.9762	0.9174
36.29 - 1.55 (1.570 - 1.550)	36.20 - 1.52 (1.540 - 1.520)	54.22 - 1.46 (1.500 - 1.460)	111.05 - 1.94 (1.941 - 1.935)
P 1 21 1	P 1 21 1	P 1 21 1	C 2 2 21
58.78 46.22 63.37	58.89 46.19 62.92	58.72 46.22 63.33	65.92 66.23 333.16
90.00 112.41 90.00	90.00 112.16 90.00	90.00 112.58 90.00	90.00 90.00 90.00
508764 (-)	464005 (-)	176660 (12101)	307997 (2720)
45884 (-)	48157 (-)	54630 (4026)	53830 (562)
10.5 (5.9)	9.6 (3.1)	3.2 (3.0)	5.7 (4.8)
100.0 (100.0)	99.5 (94.2)	99.9 (99.5)	97.8 (97.9)
15.3 (2.1)	21.4 (1.9)	6.7 (1.3)	19.5 (2.1)
0.086 (0.57)	0.057 (0.45)	0.083 (0.90)	0.045 (0.53)
- (-)	- (-)	0.054 (0.62)	0.020 (0.25)
- (-)	- (-)	0.994 (0.51)	0.999 (0.87)
0.176 / 0.201	0.179 / 0.202	0.182 / 0.211	0.208 / 0.234
3146	3205	3135	5873
99	75	43	108
244	307	264	212
327	327	327	652
20.8 / 16.5	19.3 / 15.5	22.4 / 15.8	45.2 / 35.0
40.1	29.6	28.4	58.7
28.4	27.1	30.3	45.1
0.01	0.01	0.01	0.01
0.92	0.96	0.97	0.96
3.5	3.57	3.35	2.78
Condition B	Condition B	Condition B	Condition A
5OS8	5OTR	5OTZ	6EII

19	20	21	22
CK2A_FP10	CK2A_FP10	CK2A_FP10	CK2A_FP10
Condition A	Condition A	Condition A	Condition A
DIAMOND BEAMLINE I02	DIAMOND BEAMLINE I04	DIAMOND BEAMLINE I04	DIAMOND BEAMLINE I03
0.9795	0.9323	0.9795	0.9762
167.64 - 1.94 (1.946 - 1.940)	166.61 - 2.01 (2.018 - 2.012)	46.11 - 2.05 (2.100 - 2.050)	55.33 - 1.81 (1.818 - 1.812)
C 2 2 21	C 2 2 21	C 2 2 21	C 2 2 21
64.84 68.09 335.28	64.36 68.40 333.21	64.24 67.57 332.33	64.83 67.95 331.97
90.00 90.00 90.00	90.00 90.00 90.00	90.00 90.00 90.00	90.00 90.00 90.00
721975 (7517)	393152 (3480)	299152 (16741)	490138 (4805)
55709 (605)	48568 (484)	45145 (3195)	67273 (634)
13.0 (12.4)	8.1 (7.2)	6.6 (5.2)	7.3 (7.6)
100.0 (99.8)	98.1 (99.0)	98.0 (95.6)	100.0 (100.0)
20.2 (2.3)	15.1 (2.2)	8.9 (1.3)	16.4 (2.2)
0.108 (1.49)	0.092 (0.70)	0.114 (0.96)	0.064 (1.06)
0.032 (0.44)	0.035 (0.28)	0.045 (0.41)	0.025 (0.41)
0.995 (0.80)	0.998 (0.90)	0.997 (0.61)	0.999 (0.90)
0.219 / 0.241	0.195 / 0.220	0.220 / 0.248	0.186 / 0.203
5837	5945	5757	5920
50	85	34	105
234	298	187	269
653	654	652	649
53.8 / 37.6	47.1 / 31.2	57.2 / 38.2	49.2 / 32.5
77.5	58.3	58	57.2
48.6	43.7	49.4	49.7
0.01	0.01	0.01	0.01
0.95	0.96	1.01	0.93
2.71	3.02	2.87	3.09
Condition A	Condition A	Condition A	Condition A
5OT6	5OUE	5OUM	5OUU

23	24	25	26
CK2A_KA	CK2A_FP10	CK2A_FP10	CK2A_FP10
Condition B	Condition A	Condition A	Condition A
BRUKER AXS MICROSTAR	DIAMOND BEAMLINE I04	DIAMOND BEAMLINE I02	DIAMOND BEAMLINE I04
1.54	0.9799	0.9282	0.9795
36.14 - 2.00 (2.030 - 2.000)	34.10 - 1.63 (1.670 - 1.630)	168.75 - 1.57 (1.610 - 1.570)	55.59 - 1.69 (1.730 - 1.690)
P 1 21 1	C 2 2 21	C 2 2 21	C 2 2 21
59.62 45.49 63.78	64.89 68.19 332.67	65.59 69.25 337.50	64.91 67.35 333.54
90.00 111.08 90.00	90.00 90.00 90.00	90.00 90.00 90.00	90.00 90.00 90.00
408067 (-)	523656 (15636)	855719 (57471)	515129 (37986)
21831 (-)	91089 (5958)	107777 (7882)	82464 (6056)
15.4 (9.6)	5.7 (2.6)	7.9 (7.3)	6.2 (6.3)
99.9 (100.0)	98.4 (88.9)	100.0 (100.0)	100.0 (100.0)
12.2 (1.5)	21.5 (1.2)	9.8 (1.4)	8.2 (1.1)
0.174 (0.75)	0.036 (0.81)	0.082 (1.36)	0.086 (1.50)
- (-)	0.016 (0.56)	0.031 (0.54)	0.037 (0.65)
- (-)	1.000 (0.59)	0.998 (0.65)	0.997 (0.72)
0.191 / 0.241	0.215 / 0.239	0.248 / 0.261	0.240 / 0.265
3096	6035	6092	5897
138	128	126	120
168	309	406	240
327	651	651	650
26.1 / 22.8	43.5 / 30.0	38.6 / 23.9	49.4 / 28.1
32.1	47.9	45.3	47.3
30.3	43.5	39	41.6
0.01	0.01	0.01	0.01
0.99	0.92	0.9	0.98
3.07	3.06	2.77	3.15
Condition B	Condition A	Condition A	Condition A
5OSZ	5OT5	50TD	5OTH

27	29	30	31
CK2A_KA	CK2A_FP10	CK2A_FP10	CK2A_KA
Condition B	Condition A	Condition A	Condition B
DIAMOND BEAMLINE I04	DIAMOND BEAMLINE I04	DIAMOND BEAMLINE I02	DIAMOND BEAMLINE I03
0.9795	0.9795	0.9282	0.9762
53.62 - 1.59 (1.630 - 1.590)	166.45 - 1.57 (1.610 - 1.570)	168.60 - 1.51 (1.550 - 1.510)	55.50 - 1.54 (1.580 - 1.540)
P 1 21 1	C 2 2 21	C 2 2 21	P 1 21 1
57.60 46.03 63.20	64.66 67.83 332.90	65.48 69.54 337.21	59.62 45.82 63.45
90.00 111.42 90.00	90.00 90.00 90.00	90.00 90.00 90.00	90.00 111.42 90.00
130674 (6954)	659167 (46113)	943839 (59587)	149545 (11024)
41521 (2931)	101780 (7330)	121077 (8873)	46364 (3345)
3.1 (2.4)	6.5 (6.3)	7.8 (6.7)	3.2 (3.3)
99.6 (96.7)	99.3 (97.8)	99.9 (99.7)	98.1 (96.7)
11.8 (1.2)	12.1 (1.4)	10.0 (1.1)	8.4 (1.3)
0.039 (0.71)	0.055 (0.99)	0.071 (1.50)	0.060 (0.83)
0.025 (0.54)	0.024 (0.42)	0.027 (0.62)	0.040 (0.53)
0.998 (0.53)	0.997 (0.79)	0.999 (0.60)	0.994 (0.67)
0.190 / 0.232	0.225 / 0.244	0.239 / 0.253	0.185 / 0.216
3078	5941	6094	3086
70	132	125	80
200	296	404	201
327	646	651	327
33.8 / 26.0	43.4 / 24.1	38.3 / 22.8	30.4 / 20.7
42.3	49.3	47.6	33.6
37.6	37.4	38.8	34.5
0.01	0.01	0.01	0.01
0.97	0.95	0.91	0.94
3.22	3.16	2.81	3.12
Condition B	Condition A	Condition A	Condition B
5OTI	5OTL	5OTO	5OYF

32	33	CAM4712	CAM4712 + 37
CK2A_KA	CK2A_KA	CK2A_KA	CK2A_KA
Condition B	Condition B	Condition B	Condition A
DIAMOND BEAMLINE I03	DIAMOND BEAMLINE I03	DIAMOND BEAMLINE I03	DIAMOND BEAMLINE I03
0.9762	0.9762	0.9763	0.9762
56.38 - 1.95 (2.000 - 1.950)	59.04 - 1.38 (1.420 - 1.380)	53.80 - 1.48 (1.520 - 1.480)	36.30 - 1.40 (1.440 - 1.400)
P 1 21 1	P 1 21 1	P 1 21 1	P 1 21 1
71.81 46.48 101.35	59.90 44.98 63.47	58.11 46.22 63.60	58.29 46.29 63.05
90.00 94.47 90.00	90.00 111.53 90.00	90.00 112.21 90.00	90.00 111.93 90.00
159064 (11688)	190559 (7222)	165800 (11661)	198321 (14625)
48721 (3571)	63430 (3835)	51587 (3778)	61116 (4497)
3.3 (3.3)	3.0 (1.9)	3.2 (3.1)	3.2 (3.3)
99.3 (99.8)	97.9 (80.7)	98.8 (98.6)	99.3 (99.7)
10.7 (1.8)	8.8 (1.0)	10.3 (1.5)	11.1 (1.4)
0.068 (0.75)	0.049 (0.53)	0.062 (0.81)	0.056 (0.85)
0.044 (0.49)	0.032 (0.46)	0.040 (0.54)	0.036 (0.55)
0.998 (0.52)	0.998 (0.66)	0.998 (0.50)	0.998 (0.49)
0.189 / 0.218	0.183 / 0.198	0.169 / 0.192	0.178 / 0.194
6048	3145	3057	3051
182	53	48	48
262	249	186	206
654	327	327	327
40.8 / 34.7	28.0 / 18.7	27.3 / 21.5	24.6 / 20.0
43.9	34.3	33	30
43.7	33.1	32.6	30.1
0.01	0.01	0.01	0.01
0.98	1.01	0.99	0.97
3.15	3.39	3.41	3.51
Condition B	Condition B	Condition B	Condition A
6EHU	5OTQ	5OTY	6EHK

Crystallisation and cryo conditions

A: 107mM Mes pH 6.5, 29% glycerol ethoxylate, 1 M ammonium acetate

B: 112.5mM Mes pH 6.5, 35% glycerol ethoxylate, 180 mM ammonium acetate
